# Chromosome-level genome assembly and methylome profile yield insights for the conservation of endangered loggerhead sea turtles

**DOI:** 10.1093/gigascience/giaf054

**Published:** 2025-06-06

**Authors:** Eugenie C Yen, James D Gilbert, Alice Balard, Albert Taxonera, Kirsten Fairweather, Heather L Ford, Doko-Miles J Thorburn, Stephen J Rossiter, José M Martín-Durán, Christophe Eizaguirre

**Affiliations:** School of Biological and Behavioural Sciences, Queen Mary University of London, London, E1 4DQ, UK; School of Biological and Behavioural Sciences, Queen Mary University of London, London, E1 4DQ, UK; School of Biological and Behavioural Sciences, Queen Mary University of London, London, E1 4DQ, UK; Project Biodiversity, Mercado Municipal, Local 22, Santa Maria, Ilha do Sal, 4111, Cabo Verde; Project Biodiversity, Mercado Municipal, Local 22, Santa Maria, Ilha do Sal, 4111, Cabo Verde; School of Geography, Queen Mary University of London, London, E1 4NS, UK; School of Biological and Behavioural Sciences, Queen Mary University of London, London, E1 4DQ, UK; School of Biological and Behavioural Sciences, Queen Mary University of London, London, E1 4DQ, UK; School of Biological and Behavioural Sciences, Queen Mary University of London, London, E1 4DQ, UK; School of Biological and Behavioural Sciences, Queen Mary University of London, London, E1 4DQ, UK

**Keywords:** reference genome, loggerhead sea turtle, endangered species, epigenomics, DNA methylation, temperature-dependent sex determination, Oxford Nanopore Technology

## Abstract

**Background:**

Characterizing genetic and epigenetic diversity is crucial for assessing the adaptive potential of threatened populations and species in the face of climate change. Sea turtles are particularly vulnerable due to their temperature-dependent sex determination (TSD) system, which heightens the risk of extreme sex ratio bias and extinction under future climate scenarios. High-quality genomic and epigenomic resources will therefore support conservation efforts for these endangered flagship species with such plastic traits.

**Findings:**

We generated a chromosome-level genome assembly for the loggerhead sea turtle (*Caretta caretta*) from the globally important Cabo Verde rookery. Using Oxford Nanopore Technology (ONT) and Illumina reads followed by homology-guided scaffolding to the same species, we achieved a contiguous (N50: 129.7 Mbp) and complete (BUSCO: 97.1%) assembly, with 98.9% of the genome scaffolded into 28 chromosomes and 33,887 annotated genes. We also extracted the blood methylome profile from our ONT reads, which was confirmed to be representative of the reference population via whole-genome bisulfite sequencing of 10 additional loggerheads from the same population. Applying our novel resources, we revealed high conservation of synteny between sea turtle species, reconstructed population size fluctuations in line with major climatic events, and identified microchromosomes as key regions for monitoring genetic diversity and epigenetic flexibility. Isolating 199 TSD-linked genes, we further built a large network of functional protein associations and blood-based methylation patterns.

**Conclusions:**

We present a high-quality loggerhead sea turtle genome and methylome from the globally significant East Atlantic population. By leveraging ONT sequencing, we generate genomic and epigenomic resources simultaneously and showcase the potential of this approach for driving molecular insights for conservation of endangered sea turtles.

## Background

With biodiversity declining at an alarming rate [1], genomic tools are increasingly being deployed to inform conservation management strategies for endangered species [2]. Characterizing genetic diversity [3, 4], inbreeding [5], demographic history [6], and locally adapted genomic regions [7] can all provide insights into the adaptive potential of populations and species [8]. Conservation epigenomics has recently gained momentum, driven by technological advancements and falling sequencing costs [9]. This is a promising framework as it offers an additional layer of molecular information that can directly link individuals to their environment [10]. For instance, quantifying epigenetic variation can aid in assessing a population’s capacity for adaptive plastic responses or provide biomarkers that reflect individual health and environmental exposure [9, 11, 12]. In particular, DNA methylation—the addition of a methyl group to cytosine residues to regulate gene expression—is the best-described epigenetic modification in nonmodel species to date [13].

There are 7 species of sea turtles, with 6 classified as Vulnerable to Critically Endangered and 1 as Data Deficient by the IUCN Red List [14]. Beyond threats such as bycatch, poaching, and coastal development [15], sea turtles are climate-vulnerable because of their poikilothermic physiology and temperature-dependent sex determination (TSD) system, where higher incubation temperatures induce female development [16]. As theoretical studies predict a significant shift toward female-biased primary sex ratios and subsequent population collapse by 2100 [17, 18], it is essential to assess whether sea turtles can sustain viable sex ratios via adaptive responses [19, 20]. Although their capacity for genetic evolution is constrained by long generation times and small effective population sizes [21], plastic responses via epigenetic mechanisms could offer alternative pathways, especially as they already play a role in TSD regulation [22–24]. A lack of high-quality reference genomes previously hindered such molecular insights in sea turtles. However, this situation is changing, with chromosome-level genome assemblies released at the time of writing for the green sea turtle (*Chelonia mydas*, NCBI TaxID: 8469) [25], leatherback sea turtle (*Dermochelys coriacea*, NCBI TaxID: 27794) [25], hawksbill sea turtle (*Eretmochelys imbricata*, NCBI TaxID: 27787) [26], and a loggerhead sea turtle (*Caretta caretta*, NCBI TaxID: 8467) [27]. The latter was sequenced by the Canada BioGenome Project (CBP) and represents individuals from the Adriatic Sea. Yet, given the well-known influence of reference bias on downstream population-based analyses, continuing to build genome resources remains crucial [28].

**Figure 1:**
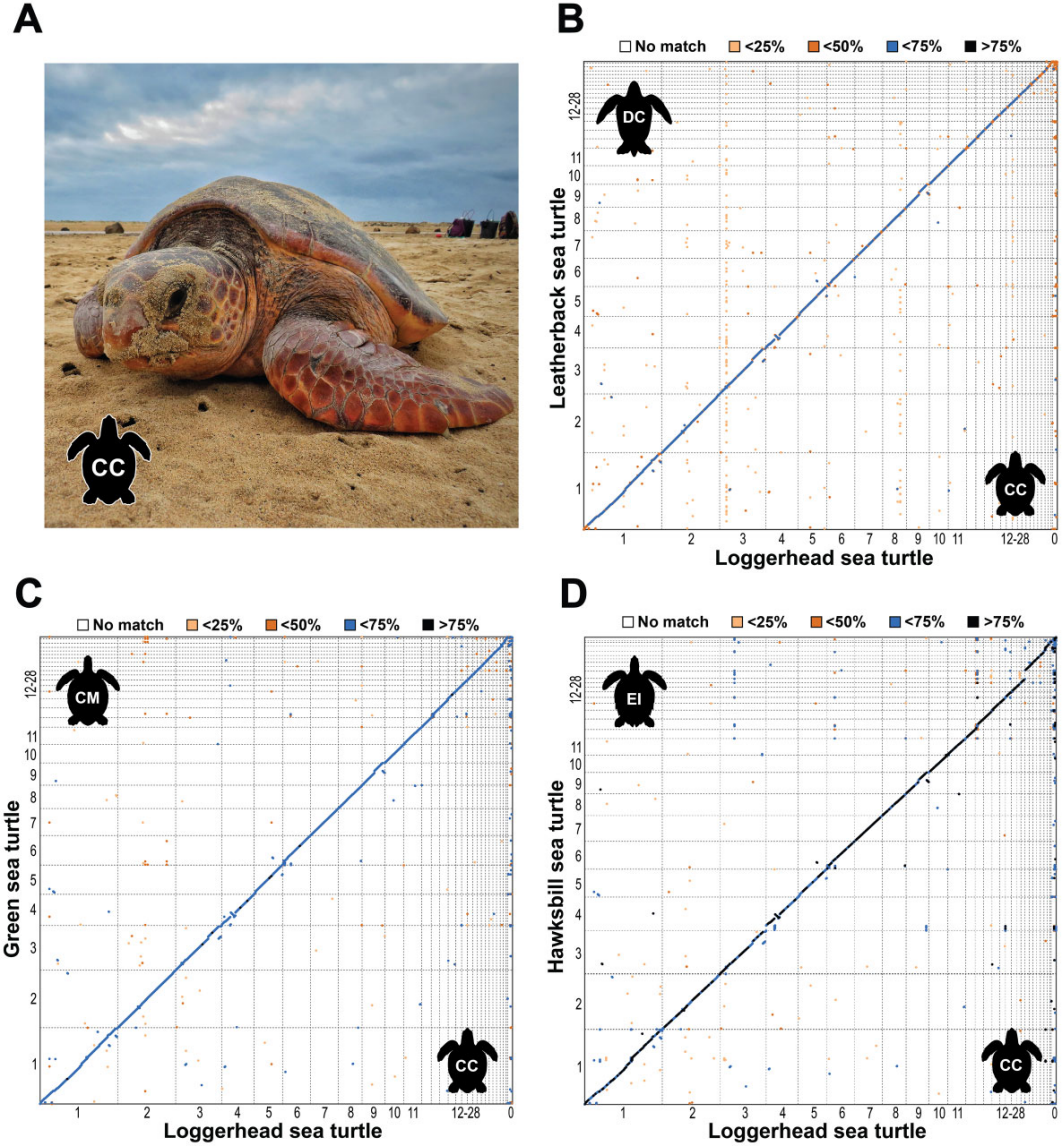
A chromosome-level genome assembly for loggerhead sea turtles from the East Atlantic nesting aggregation. (A) A loggerhead sea turtle (*Caretta caretta*; CC) nesting in Cabo Verde on Sal Island, our reference population. Photo credit: Project Biodiversity. (B–D) Whole-genome alignment dot plots of our loggerhead sea turtle assembly against publicly available chromosome-level assemblies for the (B) leatherback sea turtle (*Dermochelys coriacea*; DC; top 100,000 alignments shown), (C) green sea turtle (*Chelonia mydas*; CM), and (D) hawksbill sea turtle (*Eretmochelys imbricata*; EI). Axes show chromosome number (1–11: macrochromosomes, 12–28: microchromosomes, 0: unplaced contigs), and colors represent alignment sequence identity (%).

Here, we present a chromosome-level assembly for a loggerhead sea turtle from the Cabo Verde (East Atlantic) nesting aggregation (Fig. 1A, Population IUCN Red List Status: Endangered), which is now the largest worldwide for this species [29]. The population is composed of genetically distinct nesting groups maintained by strong female philopatry across the archipelago [30]. Our population-specific assembly complements the existing loggerhead turtle genome “GSC_CCare_1.0” [27] by eliminating reference bias for genomic studies of the globally important East Atlantic rookery, while providing a high-quality loggerhead genome for comparative studies. In addition, we present the first methylome profile derived from Oxford Nanopore Technology (ONT) reads for a sea turtle species, which we compared against methylomes of 10 loggerheads from the same population obtained via whole-genome bisulfite sequencing (WGBS). We next applied our novel resources to describe genome-wide synteny, demographic history, and genomic properties of our target genome. Lastly, we described the chromosomal locations of 199 TSD-linked genes, then created a map of their methylation status and predicted functional associations, providing a useful resource for future epigenetic studies of these endangered TSD species.

## Methods

### Reference sample collection

On 31 August 2020, we sampled blood from a wild female loggerhead turtle (ID: SLK063, Permit: 013/DNA/2020) that nested on Sal Island of the Cabo Verde Archipelago. The nesting season extends between late June and October at this rookery. The sampling site (16.62123, −22.92972) was on Algodoeiro Beach, which consists of 800 m of sandy coastline. Blood was collected from the dorsal cervical sinus with a 40-mm, 21-gauge needle and a 5-mL syringe following oviposition [31]. A Passive Integrated Transponder tag was added to the front right flipper for identification [30]. The sample was stored in a lithium heparin tube and then centrifuged for 1 minute at 3,000 rpm to separate plasma and blood cells. Samples were stored at −18°C during the field season, then at −80°C following transport to Queen Mary University of London (London, UK).

### DNA extraction, sequencing, and quality control

Genomic DNA was extracted from the nucleated blood cells using a Genomic-Tips 100 G Kit (Qiagen). For ONT sequencing, libraries were constructed using an SQK-LSK109 Ligation Sequencing Kit, and sequencing was conducted on the PromethION 24 platform with a FLO-PRO002 (R9.4.1 chemistry) flow cell (Oxford Nanopore Technologies) with a benchmarked sequencing error rate of ∼7.16% [32]. Base calling was performed via Guppy v.4.0.11 in high-accuracy mode [33]. This generated 11,643,721 reads (50,075,750,398 bp, ∼23.3× sequencing depth) with an N50 of 8,230 bp. All downstream bioinformatic steps were conducted on the Apocrita High Performance Computing Cluster [34]. Adapters were trimmed with PoreChop v.0.2.4 [35], and reads were filtered for a Phred score >Q8 [36] and length >500 bp with NanoFilt v.2.6.0 [37]. This passed 8,288,359 reads (40,371,088,741 bp, 80.6%, ∼18.8× sequencing depth), with an N50 of 8,518 bp.

We also generated Illumina sequencing data for assembly polishing. Libraries were constructed by fragmenting DNA via sonification, end polishing, A-tailing and adapter ligation, PCR amplification with P5 and indexed P7 oligos, and purification with the AMPure XP system. Sequencing was performed with 150-bp paired-end reads on the NovaSeq 6000 platform (Illumina) (RRID:SCR_016387). This gave 1,050,248,476 reads (157,537,271,400 bp, ∼73.3× sequencing depth). Haploid genome size, heterozygosity, and repeat content were estimated via GenomeScope (RRID:SCR_017014) (Supplementary Fig. S1) [38]. Reads were trimmed for adapters and filtered for a Phred score >Q20 with TrimGalore v.0.6.5 (RRID:SCR_011847) [39], passing 1,050,248,476 reads (156,382,436,354 bp, 99.3%, ∼72.9× sequencing depth).

### *De novo* assembly

ONT reads were assembled using Flye v.2.8.3 (RRID:SCR_017016) [40] in “–asm-coverage 40” mode. A polished consensus sequence was produced using Medaka v.1.3.3 with the “r941_prom_high_g4011” model [41]. Using our Illumina reads from the same sample, 2 rounds of error polishing were performed with Pilon v.1.24 [42]. The contamination level was assessed via BlobTools v.1.1.1 [43] with Diamond BLASTx v.2.0.11(RRID:SCR_001653) [44], comparing against the 2021_03 release of the UniProt reference proteomes database [45]. The assembly was haploidized using Purge_Dups v.1.2.5 [46] to give a contig-level assembly “CarCar_QM_v1.21.12.” In addition, we assembled and annotated the mitochondrial genome “CarCar_QM_v1.21.12”_Mito’ from our Illumina data with MitoZ v.3.4 (see Supplementary Text S1 for extended methods) [47].

### Homology-guided scaffolding

We used the chromosome-level Adriatic loggerhead turtle assembly “GSC_CCare_1.0” produced by the CBP for homology-guided scaffolding [27]. Unplaced contigs were removed with SAMtools v.1.9 (RRID:SCR_002105) [48], and then the remaining 28 chromosomal scaffolds served as a reference for homology-based scaffolding into chromosomes using RagTag v.2.1.0 [49]. We supply 2 versions of our assembly: (i) “CarCar_QM_v1.21.12_Sc” with 28 chromosomes and unplaced contigs/scaffolds and (ii) “CarCar_QM_v1.21.12_Sc_Chr0,” where unplaced contigs/scaffolds were concatenated into a single scaffold “SLK063_ragtag_chr0” with 100 bp of Ns as gap padding. Note this artificial chromosome was created as an option to facilitate certain computational analyses by reducing assembly fragmentation and does not represent true positional information.

### Assembly quality assessment

Contiguity was evaluated with QUAST v.5.0.2 (RRID:SCR_001228) [50]. Completeness was assessed with BUSCO scores, using BUSCO v.5.1.2 (RRID:SCR_015008) [51] in genome mode against the “sauropsida_odb10” database (*n* = 7,480 BUSCOs). The *k*-mer–based assessments were performed by comparing *k*-mers from our Illumina reads against the assembly with parameter *K* = 21. A *k*-mer spectrum was produced using KAT v.2.4.1 [52], and an assembly consensus quality value (QV) was calculated with Merqury v.1.3 [53]. Contiguity and completeness comparisons were also conducted against all assemblies currently available for sea turtles: (i) “CarCar_GSC_CCare_1.0” produced by the CBP for the Adriatic loggerhead turtle [27]; (ii) “rDerCor1.pri.v4” for the leatherback turtle and (iii) “rCheMyd1.pri.v2” for the green turtle, both produced by the Vertebrate Genomes Project (VGP) [25]; (iv) “ASM3001250v1” produced by an independent group for the hawksbill turtle [26]; and (v) “CheMyd_1.0,” which was the first draft assembly for the green turtle [54].

### Genome annotation

We performed genome annotation for our chromosome-level assembly “CarCar_QM_v1.21.12_Sc.” A repeat library was built using RepeatModeler v.2.0.4 (RRID:SCR_015027) in “LTRStruct” mode to discover long terminal repeats [55]. To exclude potential gene families, the repeat library was compared against the proteome of the “rCheMyd1.pri.v2” green turtle annotation [25] via Diamond BLASTp v.2.0.11 (RRID:SCR_001010) [44]. Transposable elements (TEs) were classified using TEclass [56], and then the curated repeat library was used for annotation via RepeatMasker v.4.1.4 (RRID:SCR_012954) [57].

For gene annotation, paired-end RNA sequencing (RNA-seq) reads (*n* = 746,132,735, Supplementary Table S1) from 24 loggerhead turtles across 3 life stages (hatchling, juvenile, and adult), 4 tissue types (blood, gonad, brain, and heart), and both sexes were mined from the Sequence Read Archive [58–61]. Reads were trimmed with Trimmomatic v.0.36 (RRID:SCR_011848) [62], mapped with STAR v.2.7.10a [63], and sorted with SAMtools v.1.9 [48]. Alignments were then supplied to BRAKER1 for gene prediction [64]. Note that species-specific training was attempted, but it resulted in a poorer annotation. Pretrained parameters for the chicken (*Gallus gallus domesticus*) were hence applied as the most related species available. Concurrently, gene prediction followed the Mikado pipeline v.2.2.4 [65] with whole-transcriptome and transcript-based hints. Transcriptomes included a publicly available blood transcriptome produced from 8 loggerhead turtles across 3 life stages [61] and a second transcriptome we assembled *de novo* using Trinity v.2.14 [66] with publicly available RNA-seq data (Supplementary Table S1) from gonad, brain, and heart tissue of 3 hatchlings [60]. Transcriptomes were mapped to our assembly via GMAP v.2021–12-17 [67], with a 99.56% and 99.98% alignment rate, respectively. From our STAR alignments, intron junctions were curated with Portcullis v.1.2.3 [68], and open reading frames were calculated using TransDecoder v.5.5.0 [69]. All evidence types were subsequently supplied for gene prediction by Mikado.

The BRAKER and Mikado gene sets were merged using the PASA pipeline v.2.5.2 (RRID:SCR_014656) [70] with 3 rounds of comparison. Finally, the gene set was filtered and standardized with AGAT v.0.9.1 [71], and in-frame stop codons were removed with gffread v.0.12.7 in “-V -H” mode [72]. To assess completeness, BUSCO v.5.1.2 [51] was run on the longest isoforms in protein mode against the “sauropsida_odb10” database (*n* = 7,480 BUSCOs). Homology-based functional information was assigned via Diamond BLASTp v.2.0.11 [44] against the SwissProt database v.2022_03_02 [45]. Gene Ontology (GO) terms were added using InterProScan 5 v.5.60–92.0 with HMMER databases: Gene3D-4.3.0, PANTHER-17.0, Pfam-35.0, PIRSR-2021, SFLD-4, SUPERFAMILY-1.75, and TIGRFAM-15.0 [73]. All functional annotations were attached via MAKER v. 2.31.9 (RRID:SCR_005309) [74].

### ONT methylation call and comparison with population-level WGBS methylation calls

From our ONT reads, we called 5-methylcytosine (5mC) and 5-hydroxymethylcytosine (5hmC) modifications in the CpG (5'-C-phosphate-G-3) context via Guppy v.6.5.7 [33] in configuration mode “dna_r9.4.1_450bps_modbases_5hmc_5mc_cg_hac_prom.” We focused on CpGs as this is the main methylation context in vertebrates [75]. Since methylation occurs symmetrically at CpGs in vertebrates [75], calls were de-stranded per CpG and then converted to bedMethyl files using Modkit v.0.1.9 mpileup [76].

To evaluate whether our ONT-derived methylation call was comparable to population-level methylation profiles obtained via a gold-standard methylation sequencing method, we generated WGBS-derived methylomes of 10 nesting adult female loggerhead turtles from the same population and locality as the reference individual (Supplementary Table S2), using the same blood sampling protocol. Genomic DNA was extracted with a DNeasy Blood and Tissue Kit (Qiagen). DNBseq libraries were constructed and sequenced with 100-bp paired-end reads on an MGI DNBSEQ platform (BGI), generating 132,192,522 ± 40,360 (SD) reads per sample (Supplementary Table S3). Extended methods for methylation calling are available in Supplementary Text S2. Briefly, alignment and methylation calling were performed via Bismark v.0.22.1 [77] with 79.1% ± 3.37% (SD) mapping efficiency. CpGs were de-stranded using the “merge_CpG.py” script [78], resulting in a sequencing depth of 9.2× ± 0.33× (SD) (Supplementary Table S3). Using the R package methylKit v.1.24.0 [79], CpGs were excluded if they had a sequencing depth lower than 8× to match the filtering threshold applied to the ONT dataset or within the 99.9th percentile to account for PCR bias [80]. Finally, CpGs were retained if they were covered in >75% of individuals. For each CpG, methylation value (%) was calculated per individual with methylKit’s “percMethylation” function.

To compare ONT and WGBS methylation calls, 5mC and 5hmC modifications were first merged for the ONT dataset because WGBS cannot distinguish between them. As methylation patterns are known to differ between genomic feature types [81], we assigned CpG locations across 4 categories (promoter, exon, intron, and intergenic regions; extended methods in Supplementary Text S3) with the R packages genomation v.1.30.0 [82] and GenomicRanges v.1.50.2 [83]. Intergenic regions were considered gene-associated if they lay within 10 kbp (kilo base pairs) from the nearest transcription start site (TSS) [84]. Prior to conducting statistical tests, we verified that the data met underlying assumptions for parametric testing by assessing the normality and homoscedasticity of residuals. When data violated assumptions, appropriate nonparametric alternatives were used. Linear models were used to test whether (i) methylation value at gene-associated CpGs and (ii) mean methylation across genes from the ONT methylome were correlated with those from the WGBS methylomes, with an interaction by gene-associated feature type. Pearson correlation coefficients were calculated *post hoc* to characterize the correlation between ONT and WGBS methylomes separately per feature type. All correlations were performed against each of the 10 WGBS samples individually, as well as against the mean methylation value per CpG/gene across all 10 samples. This “average population WGBS methylome” was chosen to better represent the population-level methylation profile over individual-level noise. Finally, a χ^2^ test was used to evaluate whether the frequency of highly methylated (>70%) CpGs across the whole genome was distributed differently over feature type categories between the ONT methylome and the “average population WGBS methylome” [85]. All statistical analyses were conducted in R v.4.2.2 [86], and all plots were produced with the R package ggplot2 v.3.4.2 [87].

### Genome-wide synteny between sea turtle species

To investigate genome-level synteny among different sea turtle species, we mapped our loggerhead turtle assembly against the chromosome-level green [25], leatherback [25], and hawksbill [26] turtle assemblies. Genomes were aligned using minimap2 v.2.18-r1015 with parameter “-f 0.02” [88], and dot plots were produced in D-GENIES v.1.5.0 [89].

### Demographic history

We performed pairwise sequentially Markovian coalescent (PSMC) analysis [90] to reconstruct the effective population size (Ne) of the Cabo Verdean loggerhead turtle population (East Atlantic), using our Illumina reads for the reference individual SLK063 [91]. To extend insights across the Atlantic Ocean, we repeated this analysis with publicly available Illumina reads for a loggerhead from a Brazilian (Bahia, West Atlantic) population (BioSample: SAMN20502673, SRA Run: SRR15328383) [92]. Reads were aligned via BWA-MEM v.0.7.17 [93], with a mapping rate of 99.8% for SLK063 and 98.7% for SAMN20502673. Alignments were sorted with SAMtools v.1.9 [48], and duplicates were tagged with Picard MarkDuplicates v.2.26.9 [94]. Variant calling and consensus building were conducted in BCFtools v.1.19 with a base and mapping filter of >Q30 [95]. Sites between a third and twice the mean sequencing depth (SLK063: ∼51.7×, SAMN20502673: ∼22.5×) were retained [25]. PSMC v.0.6.5 was run on the 11 macrochromosomes (1.73 Gbp, 80.8% of total assembly) with parameters “-N25 -t15 -r5 -b -p "4+25*2+4+6” [25] and 100 bootstraps. PSMC was also run on microchromosomes to verify that patterns were similar (Supplementary Fig. S2). Outputs were scaled with a mutation rate of 1.2^–8^ [25] and a generation time of 45 years. This was calculated by adding the age of maturity to half the reproductive longevity [92], where the age of maturity was estimated as ∼30 years in Cabo Verde using the length-at-age relationship [96]. Global mean surface temperature anomaly was plotted relative to preindustrial times with climate data inferred from marine sediments [97, 98].

### Genome properties

We used our Illumina data for the reference individual to estimate genome-wide heterozygosity [99]. Reads were aligned via BWA-MEM v.0.7.17 with a mapping rate of 99.6% [93] and sorted with SAMtools v.1.9 [48], and then PCR duplicates were tagged with Picard MarkDuplicates v.2.26.9 [94]. Variants were called including monomorphic sites with GATK v.4.2.6.1 [100] HaplotypeCaller in “-ERC BP_RESOLUTION” mode, followed by genotyping via GenotypeGVCFs with expected heterozygosity set to 0.00179, as estimated by GenomeScope (Supplementary Fig. S1). Unused alternate alleles were removed, and sites between a third and twice the mean sequencing depth (∼56.1×) were retained [25]. Other filters applied were quality by depth >2.0, root mean square mapping quality >50.0, mapping quality rank sum test >−12.5, read position rank sum test >−8.0, Fisher strand bias <60.0, and strand odds ratio <3.0 [100]. Heterozygosity was computed in nonoverlapping 100-kbp windows with the “popgenWindows.py” script in “-indHet” mode [101].

Next, we summarized a selection of genetic and methylation properties per chromosome in our reference assembly to explore differences between the 11 macrochromosomes and 17 microchromosomes of the loggerhead genome [102]. Genetic properties included heterozygosity (%), gene density (number of genes by chromosome length), CpG density (number of CpG sites by chromosome length), and repeat content (%). Methylation properties included mean methylation and proportion of highly methylated (>70%) CpGs. Nonparametric Wilcoxon rank-sum tests were used to investigate median differences between chromosome types per property, since Shapiro–Wilk tests indicated nonnormally distributed data. To examine relationships between properties, separate linear models were implemented to test whether different properties were correlated, with an interaction by chromosome type to test if relationships differed between macro- and microchromosomes.

### TSD-linked genes: identification and synteny between sea turtle species

To identify TSD-linked genes in our loggerhead turtle genome (see Supplementary Text S4 for extended methods), we used a list of 223 genes compiled by Bentley et al. [25]. These genes have documented links to TSD, primarily from studies of freshwater turtles and alligators. Following manual curation of this list, we identified the longest isoform orthologs of the “rCheMyd1.pri.v2” green turtle sequences [25] in our loggerhead turtle genome via BLASTn v.2.11.0 [103]. These were manually verified by integrating BLAST output and gene name information. For loggerhead genes that matched sequences on 2 chromosomes, both locations were retained if they were syntenic with the green turtle’s genes, under the assumption of a conserved duplication between these sea turtle species. If 1 sequence was syntenic and the other not, the nonsyntenic sequence was removed under the conservative assumption of assembly error. This retained 199 unique TSD-linked genes across 202 loci for downstream analyses. The chromosomal locations of TSD-linked genes in our loggerhead turtle genome were visualized and compared against the leatherback, green, and hawksbill turtle genomes with the R package Circlize v.0.4.16 [104]. All 199 TSD-linked genes in the loggerhead turtle genome annotation were present in the green and leatherback turtle genome annotations, while 192 TSD-linked genes were present in the less complete hawksbill turtle genome annotation for comparison.

### TSD-linked genes: comparison of methylation patterns

We examined whether methylation differs between TSD-linked and non-TSD-linked genes in the loggerhead ONT blood methylome. For the non-TSD-linked gene set, we used single-copy orthologs representing evolutionarily conserved genes in sea turtles. These were identified with OrthoFinder v.2.5.4 [105] in nucleotide mode between our loggerhead turtle genome, as well as the green [25], leatherback [25], and hawksbill [26] turtle genomes, excluding TSD-linked genes. Overall, 11,211 single-copy orthologs were covered in our loggerhead methylome and included in this analysis. We tested whether methylation differs between gene categories. To satisfy statistical assumptions, 1,000 random subsamples of 199 orthogroups were generated for comparison against the 199 TSD-linked genes. For each subsample, a linear model was used to test if mean methylation was associated with gene category, with an interaction by feature type. A quasi-Poisson generalized linear model was used to test whether the highly methylated CpG count was associated with gene category in an interaction by feature type, with an offset of total CpG count.

### TSD-linked genes: a functional association map

We built a functional association network for TSD-linked genes with the STRING v.12.0 database [106]. Protein sequences from the VGP green turtle [25] were available on STRING and used as query sequences. Proteins were retained if they matched the name of the target gene or had a sequence homology match >80%. This left 191 proteins, which were searched with the following options: full STRING network, 0.4 confidence, and 5% false discovery rate stringency. The Markov clustering algorithm was used to identify clusters in the network with inflation parameter 2.2 [106]. Promoter methylation status from our reference loggerhead turtle’s blood methylome was also annotated onto the protein network per TSD-linked gene, with 3 categories based on the bimodal distribution observed: high (>70%), low (<30%), and intermediate (30%–70%) methylation. A χ^2^ test was used to investigate if frequencies of promoter methylation categories differed between the 5 largest clusters.

## Results and Discussion

### Genomic resources generated

#### Genome assembly

By combining long ONT and short Illumina read sequencing, we produced a contig-level assembly (Table 1) with a total size of 2.146 Gbp, 1,799 contigs, and an N50 of 5.51 Mbp. A blob plot confirmed minimal contamination, with 99.2% of contigs mapping to Chordata and the remainder yielding no taxonomic hits (Supplementary Fig. S3). We also assembled and annotated the mitochondrial genome from our Illumina reads, consisting of a circular 16,574-bp contig with 37 genes (Supplementary Text S1, Supplementary Fig. S4). Following homology-guided scaffolding against the same species [27], 98.9% (2.123 Gbp) of the assembly was placed into 28 chromosomal scaffolds, with 698 unplaced contigs (22.7 Mbp; 1.06% of assembly). This elevated our assembly to chromosome-level contiguity with an N50 of 129.73 Mbp, comparable to the best assemblies available for sea turtle species (Table 1). With a BUSCO score of 97.1% (single copy: 96.2%, duplicated: 0.9%, fragmented: 0.4%, missing: 2.5%), our assembly is the most complete among sea turtles after the “rCheMyd1.pri.v2” green turtle genome [25] and the most complete loggerhead genome to date (Table 1, Supplementary Table S4 for BUSCO summaries). Quality is further supported by a QV score of Q41.1 (>99.99% assembly accuracy) and a *k*-mer spectrum indicating successful haploidization (Supplementary Fig. S5). Overall, these comparisons demonstrate that our East Atlantic loggerhead turtle assembly is a high-quality contribution to sea turtle genome resources.

**Table 1:** Comparison of assembly quality across sea turtle species. “CarCar_QM_v1.21.12” is our contig-level and “CarCar_QM_v1.21.12_Sc” is our chromosome-level loggerhead sea turtle (*Caretta caretta*) assembly representing the East Atlantic population. “CarCar_GSC_CCare_1.0” is the chromosome-level loggerhead sea turtle assembly representing the Adriatic Sea population [27]. “rCheMyd1.pri.v2” and “rDerCor1.pri.v4” are chromosome-level green (*Chelonia mydas*) and leatherback (*Dermochelys coriacea*) sea turtle assemblies, respectively [25]. “CheMyd_1.0” is the first, draft green sea turtle assembly [54]. “ASM3001250v1” is the chromosome-level hawksbill sea turtle (*Eretmochelys imbricata*) assembly [26]. BUSCO scores (full summaries in Supplementary Table S4) were calculated against the Sauropsida gene set (*n* = 7,480) with BUSCO v.5.1.2 [49].

Assembly	Size (Gb)	Total scaffold/contig count	Longest scaffold/contig (Mbp)	N50 (Mbp)	N50 count	GC content (%)	Complete BUSCOs (%)
**CarCar_QM_v1.21.12**	2.146	1,799	24.43	5.51	118	43.94	97.1
(Loggerhead sea turtle, contig)							
**CarCar_QM_v1.21.12_Sc**	2.146	726	352.82	129.73	5	43.94	97.1
(Loggerhead sea turtle, scaffolded)							
**CarCar_GSC_CCare_1.0**	2.134	2,008	345.74	130.96	5	44.03	96.1
(Loggerhead sea turtle, scaffolded)							
**rDerCor1.pri.v4**	2.165	41	354.45	137.57	5	43.35	96.3
(Leatherback sea turtle, scaffolded)							
**rCheMyd1.pri.v2**	2.134	93	348.27	134.43	5	44.01	97.2
(Green sea turtle, scaffolded)							
**CheMyd_1.0**	2.132	140,023	22.92	4.07	149	43.43	95.9
(Green sea turtle, contig)							
**ASM3001250v1**	2.296	208	367.35	137.21	5	44.13	97.1
(Hawksbill sea turtle, scaffolded)							

#### Genome annotation

We identified and masked 919 Mbp (42.8% of assembly) of repetitive elements (see Supplementary Table S5 for element type breakdown). A total of 33,887 protein-coding genes were annotated with a mean gene length of 51.0 kbp, of which 27,817 genes (82.1%) were functionally annotated via homology and 19,966 genes (58.9%) were assigned GO terms (see Supplementary Table S6 for full gene annotation statistics). Our annotation had a BUSCO completeness score of 95.4% (single copy: 94.4%, duplicated: 1.0%, fragmented: 1.1%, missing: 3.5%), which is of intermediate completeness between existing chromosome-level annotations for sea turtles (see Supplementary Table S7 for full BUSCO summaries). Future improvements could involve optimizing species-specific tuning and manual curation steps through collaborations with annotation experts [107].

#### Methylation call

By calling methylation from our ONT reads, we provide an additional layer of molecular information to facilitate epigenomic insights for loggerhead sea turtle conservation via minimally invasive blood sampling. Out of 26,449,075 CpGs in total, 22,327,230 CpGs had only 5mC modifications, 120,983 CpGs had only 5hmC modifications, and 2,986,762 CpGs had a combination of both (Table 2). The mean genome-wide methylation level was 76.0%, and the proportion of highly methylated (>70%) CpGs was 74.1% for the ONT methylome. We also assessed whether our reference individual’s ONT methylome was comparable to 10 additional methylomes of adult female nesting loggerheads from the same population. These were sequenced via WGBS, as this is the current gold standard for base-resolution methylation analysis [108]. Averaged across all 10 individuals, the mean genome-wide methylation level was 75.5% ± 0.75% (SD), and the proportion of highly methylated (>70%) CpGs was 75.2% ± 1.58% (SD) (Supplementary Table S3). Methylation estimates were therefore consistent between the reference ONT and population-level WGBS methylation calls, as well as a reported genome-wide methylation level of ∼70% across Testudines [75]. Highly methylated CpGs were similarly distributed across feature types between the ONT and “average population WGBS methylome” (χ^2^ = 0.0387, *P* = 0.998, Supplementary Fig. S6).

**Table 2:** ONT methylation call statistics for the reference individual

ONT methylation call statistics	
Total CpGs	26,449,075
Total CpGs with 5mC modifications only	22,327,230 (84.4%)
Total CpGs with 5hmC modifications only	120,983 (0.46%)
Total CpGs with 5mC and 5hmC modifications	2,986,762 (11.3%)
Total unmethylated CpGs	1,014,100 (3.83%)
Mean/median sequencing depth per CpG	17.0/16.0

**Figure 2:**
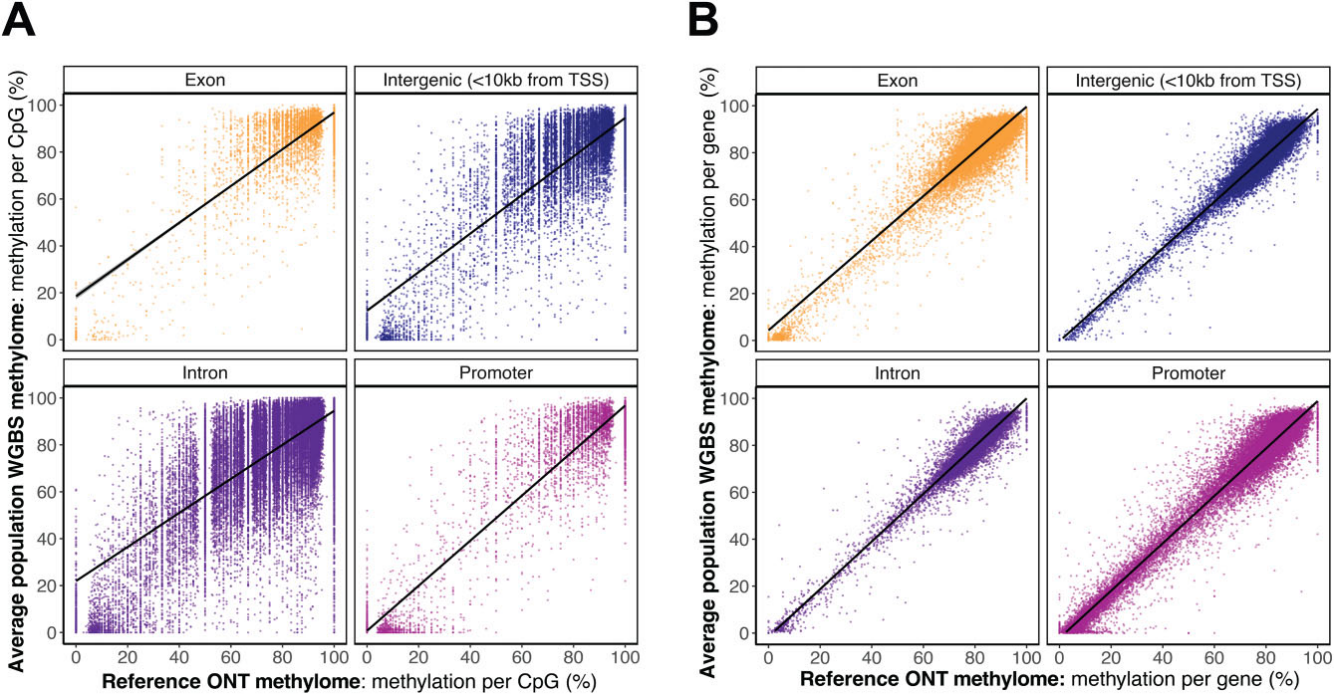
Comparison of the ONT methylome with population-level WGBS methylomes. Correlation of methylation value (%) between the reference individual’s ONT-derived methylome and the “average population WGBS methylome,” which represents the mean methylation of 10 additional loggerhead sea turtles sequenced from the same population via WGBS. Correlations are split by gene feature type (exons, introns, promoters, and gene-associated intergenic regions <10 kbp from the TSS), plotted for (A) methylation per gene-associated CpG for a random subsample of 100,000 out of 9,341,292 gene-associated CpGs covered in both datasets and (B) mean methylation per gene by feature type across 25,161 genes covered in both datasets.

At 9,341,292 gene-associated CpGs covered by both datasets, per-CpG methylation values between the ONT and “average population WGBS methylome” exhibited an interaction by feature type (*F*_1, 9,303,195_ = 33,969, *P* < 0.0001, Fig. 2A), due to different positive correlations for each feature type (exons: *r*(486,723) = 0.81, *P* < 0.0001; introns: *r*(6,895,343) = 0.75, *P* < 0.0001; promoters: *r*(499,018) = 0.95, *P* < 0.0001; intergenic: *r*(1,422,111) = 0.84, *P* < 0.0001). When focusing on mean methylation per gene at 25,161 genes covered in both datasets, we also found an interaction by feature type (*F*_1, 84,631_ = 210.3, *P* < 0.0001; Fig. 2B), with positive correlations and higher correlation coefficients than the CpG-based analysis (exons: *r*(20,348) = 0.93, *P* < 0.0001; introns: *r*(18,968) = 0.96, *P* < 0.0001; promoters: *r*(23,771) = 0.98, *P* < 0.0001; intergenic: *r*(21,544) = 0.95, *P* < 0.0001). This result is expected given the smoothening of individual-level noise on the per-gene level analysis versus the per-site level analysis [109]. Note that results were comparable when testing the ONT methylome against each of the 10 WGBS methylomes separately (Supplementary Tables S8–S9).

These results suggest that our ONT-derived methylome is representative of genome-wide methylation profiles for nesting female loggerheads from the East Atlantic population. Previous benchmarking studies via paired ONT and WGBS sequencing further demonstrate ONT as a robust alternative approach for methylation analysis [110–113], particularly following the arrival of R10.4 flow cell chemistry with <1% sequencing error rates [114]. By measuring real-time ionic current fluctuations, ONT enables the simultaneous acquisition of genomic and methylation sequencing data from native DNA [115, 116]. This maximizes molecular insights while removing technical biases introduced by amplification and bisulfite conversion processes. Unlike WGBS, ONT can also distinguish between base modification types that have different regulatory implications, such as 5mC and 5hmC [117]. Together, these properties support ONT as a cost-effective and powerful sequencing method for conservation epigenomic studies.

### Application of genomic resources

#### Genome-wide synteny between sea turtle species

The loggerhead turtle genome displayed high conservation of synteny against the leatherback (Fig. 1B), green (Fig. 1C), and hawksbill turtle genomes (Fig. 1D). Possible inversions were detected on chromosomes 4 and 9 of the loggerhead turtle genome against all other species, with future validation required to determine whether these represent true loggerhead-specific rearrangements or assembly artifacts. Sequence similarity between pairwise alignments (Supplementary Table S10) was highest between the loggerhead and hawksbill turtle genomes (>50% identical: 97.4%, >75% identical: 16.4%), followed by the green turtle genome (>50% identical: >90.1%, >75% identical: 0.01%), and lastly the leatherback turtle genome (>50% identical: 3.37%, >75% identical: 0%). This is coherent with phylogenetic expectations: within the Cheloniidae family, loggerheads turtles diverged from hawksbill turtles ∼20 million years ago (Mya) and from green turtles ∼40 Mya, compared to the deeper split of ∼75 Mya from leatherback turtles in the Dermochelyidae family [92]. Overall, our results extend the observation of high genomic stability within the sea turtle lineage [25].

#### Demographic history

**Figure 3:**
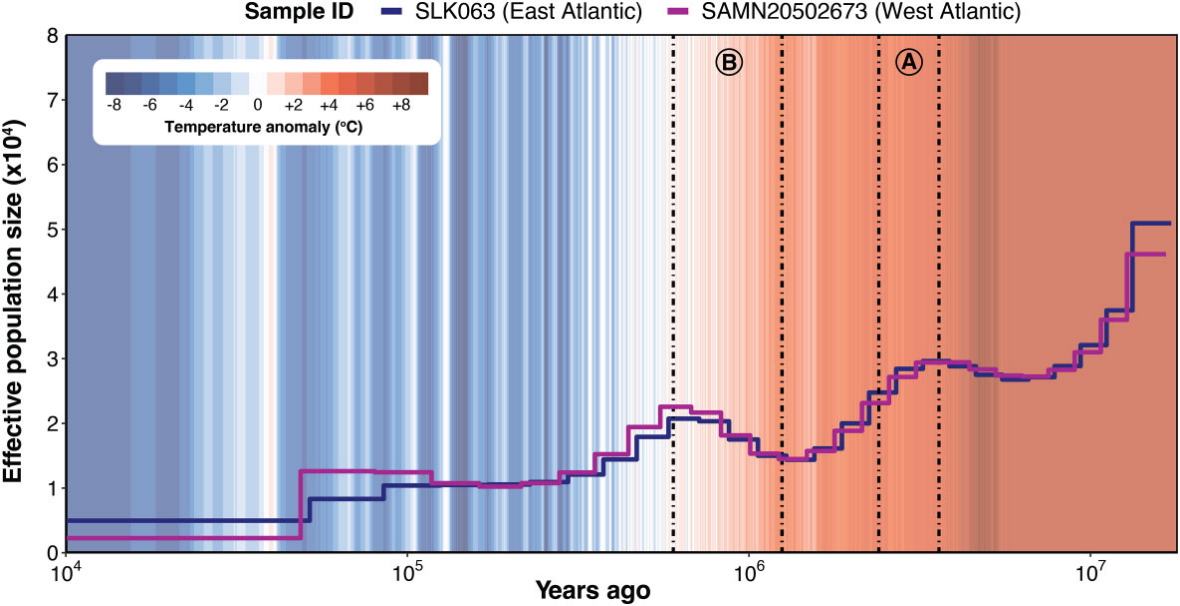
Demographic history reconstruction for loggerhead sea turtle populations across the East and West Atlantic Ocean. The East Atlantic population is in Cabo Verde (SLK063, blue line), and the West Atlantic population is in Bahia State, Brazil (SAMN20502673, purple line) [92]. Effective population size (Ne, ×10^4^) was reconstructed using PSMC over ∼17 Mya (log-scaled). Marine sediments were used to infer the global mean surface temperature anomaly (red and blue climate stripes) compared to preindustrial times [97, 98]. Letters designate geoclimatic events of interest: (A) Northern Hemisphere glaciation (∼3.3 to 2.4 Mya). (B) Mid-Pleistocene Transition (∼1.25 to 0.60 Mya).

We used PSMC to reconstruct the effective population size (Ne) of loggerheads from East Atlantic (ID: SLK063, Cabo Verde) and West Atlantic (ID: SAMN20502673, Bahia State, Brazil) [92] populations. An overall decline in Ne was detected across ∼17 million years of reconstruction (Fig. 3). In line with contemporary estimates, the East Atlantic population had a higher Ne (∼5,000) than the West Atlantic population (∼2,000) near present times, although the West Atlantic population had higher Ne over the populations’ histories. Interestingly, Ne fluctuations were similar in timing and amplitude for both populations. This suggests that major climatic and oceanic processes affecting the entire Atlantic Ocean, rather than region-specific events, were the primary drivers of loggerhead turtle demographic changes. Specifically, a population contraction occurred during the onset and intensification of Northern Hemisphere glaciation (∼3.3–2.4 Mya), as global temperatures and atmospheric carbon dioxide dropped and ice sheets expanded [118, 119]. This period of cooling, coupled with the migration of high productivity centers from polar to equatorial regions, likely reduced habitable zones for loggerhead turtles, as reflected in the relatively rapid decrease in Ne during this interval. During the mid-Pleistocene Transition (∼1.25–0.60 Mya), prolonged and intensified glacial intervals resulted in glacial cooling and expansion of ice sheets [120]. At that time, productivity centers shifted from equatorial regions toward subpolar regions and were increasingly variable on glacial-interglacial time scales [121, 122]. These shifts may coincide with Ne expansion in loggerhead turtles, which also matches their proposed migration history in the Atlantic Ocean [123]. Altogether, our new loggerhead turtle genome enabled us to trace population-level dynamics, emphasizing the importance of climate and niche availability for sea turtles. Such information can inform predictive models of demographic responses to current anthropogenic climate change.

#### Genome properties

**Figure 4:**
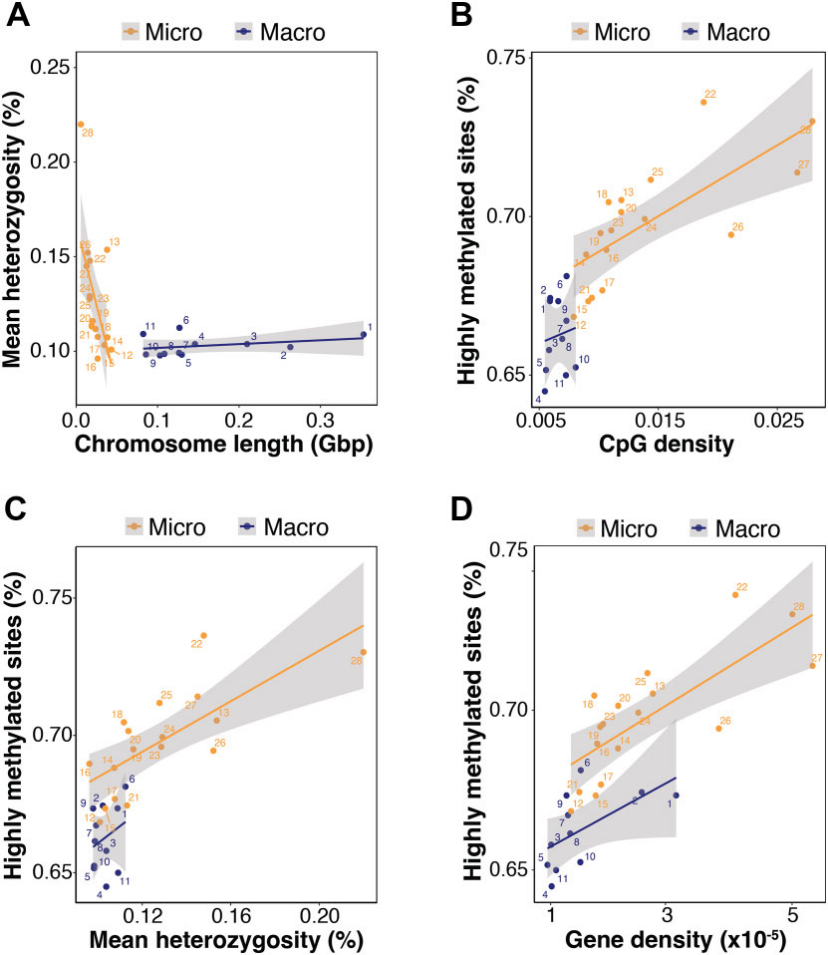
Comparison of chromosome-level characteristics. Relationships between genome properties in macrochromosomes (dark blue) versus microchromosomes (yellow) of the loggerhead turtle genome. (A) Mean heterozygosity (%) exhibits an interaction by chromosome length (Gbp) and type, with a negative correlation in microchromosomes but no correlation in macrochromosomes. (B) The proportion of highly methylated (>70%) CpGs (%) is higher overall for microchromosomes and positively correlates with CpG density. A similar relationship is observed between highly methylated CpGs (%) against (C) mean heterozygosity (%) and (D) gene density (total genes by chromosome length ×10^−5^).

Genome-wide heterozygosity was estimated as ∼0.12% for our loggerhead turtle genome, in alignment with ∼0.11% reported for the Adriatic loggerhead turtle genome [27]. This places the genomic diversity of loggerhead turtles at approximately 4 times that of leatherback turtles (∼0.0029%), less than half of green turtles (∼0.25%), and about a third of hawksbill turtles (∼0.33%) [25, 26]. Genome-wide heterozygosity patterns further varied among the 28 chromosomes, with microchromosomes being more heterozygous than macrochromosomes (W = 29, *P* = 0.002; Supplementary Fig. S7A). Heterozygosity was predicted by an interaction between chromosome length and type (length × type: *F*_1, 24_ = 12.3, *P* = 0.002; Fig. 4A), with a negative correlation in microchromosomes (*F*_1, 15_ = 7.88, *P* = 0.013) but none detected for macrochromosomes (*F*_1, 9_ = 1.09, *P* = 0.324). Microchromosomes were also more gene-dense (W = 28, *P* = 0.001; Supplementary Fig. S7B), GC-rich (W = 1, *P* < 0.0001; Supplementary Fig. S7C), CpG-dense (W = 1, *P* < 0.0001; Supplementary Fig. S7D), and less repeat-rich overall (W = 166, *P* = 0.0003; Supplementary Fig. S7E). This is consistent with patterns described in other sea turtles [25] and wider vertebrates with both macro- and microchromosomes [124, 125].

We next performed comparisons to address the knowledge gap of methylation differences between macro- and microchromosomes. From our ONT-derived blood methylome, mean methylation was similar between chromosome types (W = 54, *P* = 0.07; Supplementary Fig. S7F), but microchromosomes had a greater proportion of highly methylated CpGs (W = 54, *P* < 0.0001; Supplementary Fig. S7G). This likely stems from higher CpG density on microchromosomes providing more methylatable sites [126], as supported by a strong, positive correlation (CpG density: *F*_1, 24_ = 56.5, *P* < 0.0001; type: *F*_1, 24_ = 8.67, *P* = 0.007; Fig. 4B) without an interaction by chromosome type (CpG density × type: *F*_1, 24_ = 0.08, *P* = 0.896). This is functionally interesting as previous studies have shown that highly methylated CpG sites are often regulated more dynamically than lowly methylated CpG sites [127]. Correlations without interactions were also observed between the proportion of highly methylated CpGs against heterozygosity (heterozygosity: *F*_1, 24_ = 50.30, *P* < 0.0001; type: *F*_1, 24_ = 17.03, *P* = 0.0004; heterozygosity × type: *F*_1, 24_ = 0.04, *P* = 0.84; Fig. 4C) and gene density (gene density: *F*_1, 24_ = 65.89, *P* < 0.0001; type: *F*_1, 24_ = 8.67, *P* = 0.007; gene density × type: *F*_1, 24_ = 0.048, *P* = 0.83; Fig. 4D), suggesting relationships between methylation and those genetic properties are shaped by evolutionary forces acting consistently across chromosome types. There was no correlation between the proportion of highly methylated CpGs and repeat content (*F*_1, 24_ = 1.64, *P* = 0.21).

In addition to microchromosomes offering a greater combination of genetic variants via independent assortment, our results highlight the unique genetic properties of vertebrate microchromosomes and add novel insights into their methylation potential. With higher heterozygosity, gene density, and regulatory opportunities via CpG methylation, microchromosomes could serve as hotspots for both adaptive evolution and plastic responses to environmental change. As such, microchromosomes represent promising targets for monitoring and preserving functional genetic and epigenetic diversity in sea turtles and other vertebrates [128].

#### TSD-linked genes: synteny between sea turtle species

**Figure 5:**
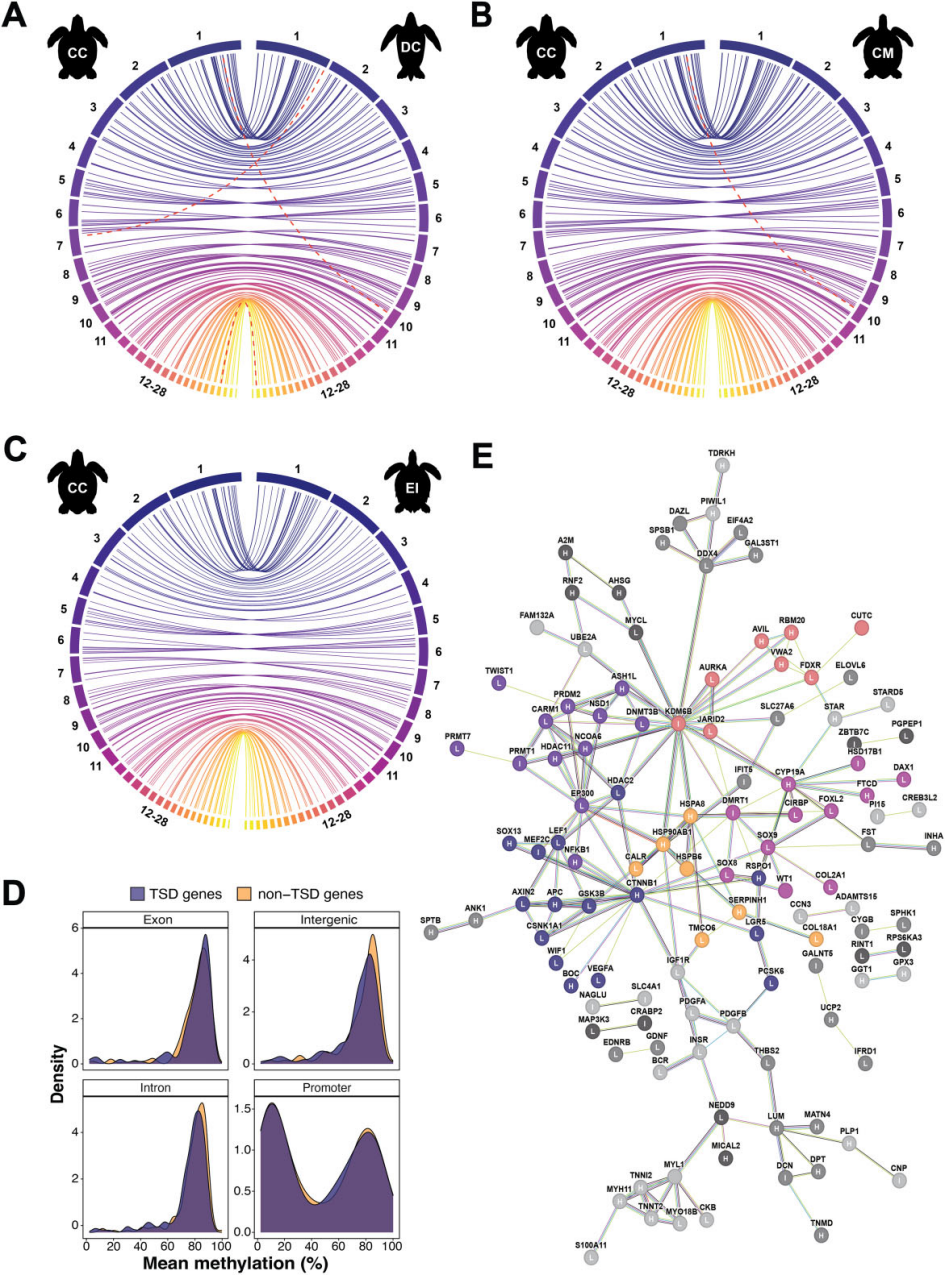
Molecular insights into TSD-linked genes: synteny, methylation, and protein functional associations. (A, B, C) Chromosomal locations of 199 TSD-linked genes across 201 loci in our loggerhead sea turtle assembly against the (A) leatherback (DC), (B) green (CM), and (C) hawksbill (EI) chromosome-level sea turtle assemblies. Chromosomes of the loggerhead assembly are plotted on the left, with colors representing the 28 chromosomes. Dashed red lines indicate genes that mapped to different locations between species (loggerhead versus green: EP300, loggerhead versus leatherback: EP300, CIRBP, IFIT5). (D) Density plots of mean methylation (%) per gene by feature type between 199 TSD-linked genes (purple) and a random subset of 199 out of 11,211 non-TSD-linked genes (yellow), identified as single-copy ortholog between sea turtle species (yellow). (E) Predicted functional association network of proteins coded by TSD-linked genes, created with STRING [107]. In total, 119 genes with at least 1 connection are shown (*n* = 28 clusters total). Nodes represent gene IDs and edges represent associations. Edge colors represent different evidence types used for association prediction. Nongray node colos represent the 5 largest functional clusters identified via Markov clustering. Letters on nodes represent methylation status of gene promoters in the reference individual methylome. Categories are based on the bimodal methylation distribution observed in Fig. 5D: H: highly methylated (>70%), L: lowly methylated (<30%), I: intermediate methylation (30%–70%). Sixty-seven promoters (33.8%) were hypermethylated, 96 (48%) were hypomethylated, and 36 (18.2%) had intermediate methylation.

As TSD species, loggerhead turtles are particularly vulnerable to climate change, with strongly female-biased sex ratios and population collapse predicted by the end of the century [20]. To gain molecular insights into this system, we compared the chromosomal locations of 199 TSD-linked genes present in our loggerhead turtle genome against other available sea turtle genomes. Consistent with Bentley et al. [25], most TSD-linked genes were single-copy and reside on equivalent chromosomes across sea turtle species (Fig. 5A–C). Two genes were syntenic between all species except the leatherback turtle (Fig. 5A): CIRBP (cold-inducible RNA-binding protein; loggerhead/green: chromosome 25; leatherback: chromosome 27) and IFIT5 (interferon induced protein with tetratricopeptide repeats 5; loggerhead/green: chromosome 7; leatherback: chromosome 1). Only the EP300 (E1A binding protein p300) gene was not syntenic between loggerheads and greens (Fig. 5B). Although it mapped to chromosome 1 in all species, a duplicate on chromosome 10 was found in the green and leatherback genomes. All TSD-linked genes in the loggerhead turtle genome were syntenic with those present in the hawksbill turtle genome (Fig. 5C).

As rearrangements and copy number variants can alter gene regulation [129], the 3 identified genes may contribute to interspecies differences in the TSD response curve and should be investigated. Nevertheless, structural variants seem rare in sea turtles overall, unlike in some other Testudines [130, 131]. Finer-scale genetic variation may instead be more important, particularly within species. For example, a single-nucleotide polymorphism on the CIRBP gene, which we found to be nonsyntenic against leatherbacks, influences TSD in snapping turtles (*Chelydra serpentina*) and exhibits varying allele frequencies with latitude [132]. It is thus crucial to continue characterizing genetic variants that contribute to the adaptive potential of the TSD mechanism, to evaluate whether different sea turtle species and populations can evolve to mitigate sex ratio skew as climate change progresses.

#### TSD-linked genes: methylation patterns

Given epigenetic mechanisms are involved in TSD regulation, we next provide broad insights into the methylation profile of 199 TSD-linked genes from our reference ONT blood methylome. Methylation distributions were similar between TSD-linked and non-TSD-linked genes comprising 11,211 single-copy orthologs identified between sea turtle species (Fig. 5D, Supplementary Fig. S8). This was supported by performing linear models comparing the 199 TSD-linked genes against 1,000 random subsets of 199 non-TSD-linked genes, which revealed feature type as the sole determinant of both the mean methylation level (Supplementary Fig. S9) and the proportion of highly methylated CpG per gene (Supplementary Fig. S10). Exons, introns, and gene-associated intergenic space (<10 kbp from TSS) were mostly hypermethylated, with a peak centered on ∼80% (Fig. 5D, Supplementary Fig. S8). In contrast, methylation was bimodally distributed for promoters, with peaks of low (∼10%) and high (∼80%) methylation. These distributions are consistent with those described across mammalian vertebrates [75, 133, 134].

Overall, we provide a genome-wide description of blood-based methylation patterns across gene feature types to guide future study design for sea turtles. Blood is a valuable tissue for conservation monitoring, particularly in reptiles with nucleated erythrocytes, as it can be collected via minimally invasive sampling yet still report on an individual’s developmental status [135–137], health [138], and environmental exposure [139, 140]. For example, a nonlethal method for sexing sea turtle hatchlings is urgently required to assess nest sex ratios as global temperatures continue to rise [20]. As expected, we did not uncover obvious differences between TSD-linked versus non-TSD-linked genes from blood tissue of an adult female loggerhead’s genome, likely given the broad scale of comparison and the timing and tissue specificity of epigenetic regulation [22, 24]. Future studies could therefore employ methylome-wide discovery scans [141] to identify blood-based biomarkers of sex in sea turtle hatchlings, as performed for American alligators (*Alligator mississippiensis*), another TSD reptilian species [142].

#### TSD-linked genes: a functional association map

Using the STRING database [106], we built a network of functional associations between proteins coded by TSD-linked genes (Fig. 5E). In total, 119 TSD-linked genes had at least 1 connection identified via Markov clustering (*n* = 28 clusters total), and the 5 largest clusters encompassed 54 TSD-linked genes (45.4%). Members of the biggest cluster (*n* = 15, dark blue) were mainly linked to Wnt signaling. Connections were centered on CTNNB1 (β-catenin), which is critical for female determination in vertebrates [143, 144]. The next largest cluster (*n* = 12, dark purple) was primarily composed of epigenetic regulators. The most connected protein was EP300 (E1A binding protein p300), a histone acetyltransferase that controls cell proliferation, with germ cell numbers proposed to influence TSD in a freshwater turtle [145]. The third cluster (*n* = 11, mauve) contained genes with well-described roles in TSD and steroid hormone signaling [139]. SOX9 (SRY-related HMG box gene 9), a key regulator of male differentiation [146, 147, 148], was most connected. Although the functional nature of the fourth cluster (*n* = 8, pink) was less obvious, the most connected protein was the histone demethylase KDM6B (lysine demethylase 6B), which is causally linked to temperature-dependent male determination in freshwater turtles [23]. The final cluster contains heat shock proteins and chaperones (*n* = 7, yellow), which could play a role in temperature sensing and transduction during TSD [149]. These protein clusters may already suggest patterns surrounding TSD in sea turtles. Sensory systems associated with heat shock responses could incorporate environmental inputs that link with epigenetic regulators to alter gene expression and cell proliferation, as well as subsequently activate known endocrine systems [150]. With growing genomic tools for sea turtles, this hypothesis will need to be tested.

Given the prevalence of epigenetic regulators in the functional network, we overlaid promoter methylation status of TSD-linked genes from our reference loggerhead turtle’s blood methylome (Fig. 5E). In total, 67 genes (33.8%) were highly (>70%) methylated, 96 genes (48.0%) were lowly (<30%) methylated, and 36 genes (18.2%) had intermediate methylation. The proportions of each promoter methylation category differed among the 5 largest functional clusters (χ^2^ = 41.697, *P* < 0.0001; Supplementary Table S11). The lowest methylated clusters involved Wnt signaling genes (dark blue; high: 33.3%, intermediate: 6.7%, low: 60%) and the well-described TSD-linked/hormone signaling genes (mauve; high: 20%, intermediate: 20%, low: 60%). No clusters were hypermethylated, as the heat shock cluster (yellow) contained the largest proportion of highly methylated genes, with an equal split between highly and lowly methylated genes. With promoter methylation classically linked to transcriptional repression [81], we may have captured female-specific methylation signatures on a broad gene network scale. Overall, this network serves to facilitate future investigations into the relationships between TSD-linked gene function, connectivity, and blood methylation status to aid sex biomarker discovery in sea turtles.

## Conclusions

To support conservation efforts of threatened loggerhead sea turtles, as well as all sea turtles and TSD species in general, we present a chromosome-level genome representing the globally significant East Atlantic nesting group. Our reference assembly will enhance genomic inferences for this population and contribute a new genome for comparative studies within and between species. Moreover, we anticipate our ONT-derived blood methylome profile can guide future epigenetic study design. To demonstrate their potential, we applied these novel resources to yield a variety of insights relevant to the conservation of sea turtles. At the population level, we emphasize the role of climate change and niche availability on effective population size fluctuations. On the chromosome level, we recommend microchromosomes as special regions for monitoring functional genetic and epigenetic diversity. Finally, we bring gene-level insights into the TSD cascade of sea turtles, highlighting 3 TSD-linked genes with potential interspecies rearrangements and providing a map of functional associations and blood-based methylation status for TSD-linked genes in an adult nesting female. By simultaneously generating genome and methylome resources to provide molecular insights in loggerhead sea turtles, our study showcases the application of this dual framework for informing the conservation of an endangered flagship species.

## Supplementary Material

giaf054_Supplemental_File

giaf054_Authors_Response_To_Reviewer_Comments_original_submission

giaf054_GIGA-D-24-00386_original_submission

giaf054_GIGA-D-24-00386_Revision_1

giaf054_Reviewer_1_Report_original_submission Zhongduo Wang -- 10/8/2024

giaf054_Reviewer_2_Report_original_submission Victor Quesada -- 10/9/2024

giaf054_Reviewer_3_Report_original_submission F. Gözde Çilingir -- 10/12/2024

giaf054_Reviewer_3_Report_Revision_1 F. Gözde Çilingir -- 3/10/2025

## Data Availability

All sequencing data and the annotated genome assembly “CarCar_QM_v1.21.12_Sc” are available under study PRJEB79015 on the European Nucleotide Archive (ENA). Supporting scripts are deposited in our GitHub repository [151]. Additional genome assembly, annotation, and methylome files mentioned in this article are available on *GigaScience* repository, GigaDB, alongside supporting data analyses [152].

## Additional Files

**Supplementary Table S1**. Publicly available RNA-seq reads mined for genome annotation. Reads were downloaded from the NCBI Sequence Read Archive (Leinonen, Sugawara, and Shumway, 2011). These comprised 746,132,735 reads from 24 loggerheads across 3 life stages (hatchling, juvenile, and adult), 4 tissue types (blood, gonad, brain, and heart), and both sexes.

**Supplementary Table S2**. Metadata for 10 nesting loggerheads sampled for WGBS.

**Supplementary Table S3**. WGBS summary statistics for 10 nesting loggerheads. Alignment, de-duplication, and methylation calling were performed with Bismark v.0.22.1 (Krueger and Andrews, 2011).

**Supplementary Table S4**. Full BUSCO summary for genome assemblies across sea turtle species. BUSCO scores were calculated against the Sauropsida gene set (*n* = 7,480) with BUSCO v.5.1.2 in genome mode (Simão et al., 2015).

**Supplementary Table S5**. Repetitive element summary statistics. Statistics generated by RepeatMasker v.4.1.4 (Smit, Hubley, and Green, 2022).

**Supplementary Table S6**. Gene annotation summary statistics. Annotation statistics were computed with AGAT v.0.9.1 (Dainat, 2022).

**Supplementary Table S7**. Full BUSCO summary for genome annotations across sea turtle species. BUSCO scores were calculated on the longest gene isoforms against the Sauropsida gene set (*n* = 7,480) with BUSCO v.5.1.2 in protein mode (Simão et al., 2015).

**Supplementary Table S8**. Linear model results for the interaction term per individual WGBS methylome. ANOVA results for the interaction term of the linear model: lm(ONT methylation value ∼ WGBS methylation value * Feature type), performed separately for each of the 10 WGBS methylomes. This analysis compared methylation values at gene-associated CpGs (top) and mean methylation per gene (bottom).

**Supplementary Table S9**. Pearson correlation test results per individual WGBS methylome. Results from a Pearson pairwise correlation test comparing methylation values between the reference ONT methylome and each of the 10 WGBS methylomes separately. Correlations were performed separately for each feature type (exons, introns, promoters, gene-associated intergenic), given an interaction by feature type was found to significant in Supplementary Table S8. This analysis compared methylation values at gene-associated CpGs (top) and mean methylation per gene (bottom).

**Supplementary Table S10**. Summary of whole-genome alignments between the loggerhead sea turtle and other sea turtle species. Alignments and statistics produced by D-GENIES v.1.5.0 (Cabanettes and Klopp, 2018).

**Supplementary Table S11**. Proportions of promoter methylation categories in the top 5 functional clusters. Methylation status of TSD-linked gene promoters in the reference individual methylome (i.e., blood of a nesting female) was assigned to high (>70%), intermediate (30%–70%), or low (<30%) categories based on the bimodal distribution observed (Fig. 5D). The proportion of gene promoters falling into these 3 categories is provided for the 5 largest functional clusters identified via Markov clustering from our STRING functional association network created for TSD-linked genes (Fig. 5E).

**Supplementary Text S1**. Extended methods and results for mitochondrial genome assembly and annotation. We assembled and annotated the mitochondrial genome from our Illumina data. To extract a read set enriched for mitochondrial sequence, reads were mapped via BWA-MEM v.0.7.17 (Li, 2013) against a published loggerhead mitochondrial assembly (Drosopoulou et al., 2012). Mapped reads were inputted to MitoZ v.3.4 (Meng et al., 2019) with the Megahit assembler. Our assembly consisted of a circular 16,574-bp contig with 37 genes (13 protein coding genes, 22 tRNA genes, and 2 rRNA genes, Supplementary Fig. S4). This assembly was 98.9% identical to a loggerhead mitochondrial genome from a Greek population (Drosopoulou et al., 2012). Divergence likely reflects genetic structure between the North Atlantic and Mediterranean nesting groups, driven by female philopatry in sea turtles (Baltazar-Soares et al., 2020; Tolve et al., 2023). To assign the reference individual to a mitochondrial haplogroup, control region haplotype sequences found in the Cabo Verde nesting group (Baltazar-Soares et al., 2020) were downloaded from NCBI GenBank (Sayers et al., 2021) and aligned against our assembly via BLASTn v.2.7.1+ (Altschul et al., 1990). The control region was 99.9% similar to the CC-A1.4 haplotype (GenBank ID: EU179439.1). The reference individual is therefore a member of Haplogroup I (CC-A1), the oldest and commonest loggerhead lineage in Cabo Verde (Baltazar-Soares et al., 2020).

**Supplementary Text S2**. Extended methods for methylation calling from WGBS data of 10 loggerheads. Raw WGBS reads were trimmed for remaining adapters and filtered for a mean Phred score >Q20 using cutadapt v.2.10 (Martin, 2011). Using Bismark v.0.22.1 with default options (Krueger and Andrews, 2011), trimmed reads were aligned against our reference assembly, giving a mean mapping efficiency of 79.1% ± 3.37% (SD) per sample (Supplementary Table S3). Alignments were de-duplicated with Bismark in paired-end mode, followed by merging and sorting with samtools v.1.9 (Li et al., 2009). Methylation calling was then performed in Bismark. Percentage methylation totaled across CHG and CHH sites were calculated to obtain an estimate of bisulfite conversion efficiency (Laine et al., 2023). This gave a mean of 99.39% ± 0.047% (SD), indicating high conversion efficiency (Supplementary Table S3). To improve coverage and minimize pseudo-replication, we further de-stranded adjacent cytosines per CpG site using the “merge_CpG.py” script (Cristofari, 2023), as methylation occurs symmetrically at CpG sites in vertebrates (Klughammer et al., 2023). On average, this resulted in 25,485,557 ± 35,209 (SD) CpG sites per sample, with a de-stranded coverage of 9.2× ± 0.33× (SD) (Supplementary Table S3). Methylation calls at CpG sites were further processed in RStudio v.4.2.2 (R Core Team, 2021) with the methylKit package v.1.24.0 (Akalin et al., 2012). CpG sites were excluded if they had a coverage lower than 8× to match the filtering threshold applied to the ONT methylome or if they were within the 99.9th percentile to account for PCR bias (Wreczycka et al., 2017). Coverage was then normalized between samples using methylKit’s “normalizeCoverage” function. As a final filtering step, we retained CpG sites that were covered in at least 75% of individuals, leaving 24,299,151 CpG sites for downstream analyses. For each CpG site, percentage methylation was calculated per individual with methylKit’s “percMethylation” function, and then the mean across all individuals was calculated per CpG site to provide an average WGBS methylome representative of the population.

**Supplementary Text S3**. Extended methods for annotating gene feature types. To annotate the feature type upon which CpG sites reside, we used the R packages genomation v.1.30.0 (Akalin et al., 2015) and GenomicRanges v.1.50.2 (Lawrence et al., 2013) alongside our reference genome. Promoter regions were defined as 1,500 bp upstream and up to 500 bp downstream of a transcriptional start site (TSS; Heckwolf et al., 2020). All CpG sites were assigned to 1 of 4 feature types using genomation’s “annotateWithGeneParts” function, in the following order of precedence when features overlapped: promoter, exon, intron, or intergenic region. To attach functional gene information to CpG sites, those in genic regions (i.e., located on a promoter, intron, or exon) were associated to a gene using the “findOverlaps” function of GenomicRanges. DMS in intergenic regions were associated with a gene using genomation’s “getAssociationWithTSS” function, if they were less than 10 kb away from the nearest TSS (Heckwolf et al., 2020).

**Supplementary Text S4**. Extended methods for identification and curation of TSD-linked genes. Bentley et al. (2023) compiled the most comprehensive list to date of 223 genes with documented links to temperature-dependent sex determination (TSD) pathways. First, we manually curated this list by removing 11 genes that were not found in either the green or leatherback genomes, consolidating GLU and NAGLU, which referred to the same gene, and correcting the gene name “ST6GALC2” to “ST6GAL2.” We further replaced 4 sequences that did not map to the corresponding gene: the A2M sequence was replaced with XM_037912788.2 for the green turtle, the PDGFA sequence was replaced with XR_003565502.3 in the green turtle and XR_005295469.2 in the leatherback turtle, the PDGFB sequence was replaced with XM_037881931.2 in the green turtle and XM_038387707.2 in the leatherback turtle, and ST6GAL2 was replaced with XM_007054611.4 in the green turtle and XM_043509904.1 in the leatherback turtle. Using our curated list of TSD-linked genes, orthologs were identified in our loggerhead assembly via BLASTn v.2.11.0 with parameters “-evalue 1e-30” and “-perc_identity 70” (Altschul et al., 1990) against gene sequences from the VGP green turtle annotation, which is more closely related to the loggerhead turtle than the leatherback turtle. To verify that true orthologs were selected, a manual curation step integrating BLAST homology and gene name information was conducted. This involved looking up the gene name for every BLAST hit in our loggerhead functional annotation and retaining those that matched the query gene name. We took the top BLAST hit if there was no match to query gene name, due to genes having multiple aliases or missing annotation information. For loggerhead genes that matched sequences in 2 chromosomal locations, hits were retained if they were both syntenic or neither syntenic with the other species. If 1 sequence was syntenic and the other was not, the nonsyntenic sequence was removed under the conservative assumption of it being an assembly or ortholog identification error. This left 201 unique TSD-linked genes in our loggerhead genome for downstream analyses (5 genes annotated in 2 locations; 206 total loci).

**Supplementary Fig. S1**. GenomeScope profile for our loggerhead genome. Produced using GenomeScope (Vurture et al., 2017) on *k*-mers derived from our raw, Illumina reads, with parameter *k* = 21. Estimated haploid genome size is 2.05 Gbp, heterozygosity is 0.179%, and repeat fraction is 20.4%. These metrics were used to help choose downstream parameters.

**Supplementary Fig. S2**. Auxiliary PSMC tests with 100 bootstraps. PSMC (Li and Durbin, 2011) run from the 11 macrochromosomes of the loggerhead genome (80.8% of assembly) for (A) SLK063 (Cabo Verde, East Atlantic) and (B) SAMN20502673 (Brazil, West Atlantic). PSMC was also run on the 17 microchromosomes (19.2% of assembly) for (C) SLK063 and (D) SAMN20502673, confirming overall patterns were comparable with macrochromosomes.

**Supplementary Fig. S3**. GC-coverage blob-plot to evaluate assembly contamination. Outputted by BlobTools v.1.1.1 (Laetsch and Blaxter, 2017) with our contig-level assembly. Scaffolds are colored by phylum, and blob size represents scaffold length. Frequency histograms are plotted along the side of each axis. Lower bar charts show the proportion of scaffolds that were mapped to each phylum. Of the 99.24% of scaffolds that successfully mapped to our assembly, 98.66% of scaffolds mapped to Chordata (blue), and 0.58% produced no hit. This confirms minimal taxonomic contamination from other phyla in our assembly.

**Supplementary Fig. S4**. Mitochondrial genome assembly and annotation. Length: 16,574 bp, GC content: 38.75%, total number of genes: 37 (protein coding genes: 13, tRNA genes: 22, rRNA genes: 2). The inner purple ring visualizes coverage. Mitochondrial assembly, annotation, and visualization were performed with MitoZ v.3.4 (Meng et al., 2019).

**Supplementary Fig. S5**. The *k*-mer spectrum plot of our loggerhead assembly. Produced using KAT v.2.4.1 (Mapleson et al., 2017), showing the frequency of *k*-mers in the assembly versus frequency of *k*-mers (i.e., sequencing coverage) in the raw Illumina reads. The first, black peak corresponds to *k*-mers present in the raw reads but missing from the assembly due to sequencing errors. The second, smaller peak corresponds to *k*-mers from heterozygous regions, and the third peak corresponds to *k*-mers from homozygous regions. This plot confirms that our assembly is well haploidized.

**Supplementary Fig. S6**. Genome-wide distribution of highly methylated (>70%) CpGs across feature types by site. The outer ring shows the distribution called from the ONT methylome of the reference individual (*n* = 19,606,231 CpGs), with 3.74% on exons (yellow), 24.64% on introns (dark purple), 2.90% on promoters (mauve), and 49.89% being intergenic (dark blue). The inner ring shows the distribution called from the average of 10 WGBS methylomes (*n* = 17,950,597 CpGs), with 3.96% on exons, 24.96% on introns, 2.78% on promoters, and 49.91% being intergenic. Highly methylated CpGs are similarly distributed across feature types between sequencing methods over the entire genome (χ^2^ = 0.0387, *P* = 0.998).

**Supplementary Fig. S7**. Comparison of macro- and microchromosome properties. Boxplots comparing genetic and epigenetic properties per chromosome, for the 11 macrochromosomes (dark blue) and 17 microchromosomes (yellow) of the loggerhead genome. Comparisons for (A) heterozygosity (%), (B) gene density (total genes over chromosome length, ×10^−5^), (C) GC content (%), (D) CpG density (total CpGs over chromosome length), (E) methylation (%), (F) proportion of highly methylated (>70%) CpGs (%), and (G) mean repeat content (%). Microchromosomes are more heterozygous (W = 29, *P* = 0.002), gene-dense (W = 28, *P* = 0.001), GC-rich, and CpG-dense (W = 1, *P* < 0.0001); possess a larger proportion of highly methylated CpGs (W = 54, *P* < 0.0001); and are less repeat-rich (W = 166, *P* = 0.0003), with the notable exception of chromosome 28, which has the highest repeat content overall.

**Supplementary Fig. S8**. Violin plot of mean methylation per gene. For TSD-linked genes (*n* = 199; purple) versus non-TSD-linked genes (*n* = 11,560 single-copy orthologs between sea turtle species; yellow).

**Supplementary Fig. S9**. *P* value histogram from comparing mean methylation between TSD-linked genes and non-TSD-linked genes. A linear model was used to test if mean methylation differed between 199 TSD-linked genes and 1,000 random subsets of 199 non-TSD-linked genes, sampled from 11,560 single-copy orthologs shared between sea turtle species. Histograms are plotted for the *P* values per iteration (*n* = 1,000), for each term in the ANOVA results table: gene category (TSD-linked vs. non-TSD-linked, dark blue), genomic feature type (purple), and their interaction (gene category × feature type, yellow). The dashed line represents *P* = 0.05. In total, 161 (16.1%) tests passed *P* < 0.05 for gene category, 1,000 (100%) tests passed for feature type, and 28 (0.28%) tests passed for the interaction term.

**Supplementary Fig. S10**. *P* value histogram from comparing the proportion of highly methylated CpGs between TSD-linked genes and non-TSD-linked genes. A quasi-Poisson generalized linear model was used to test if the count of highly methylated CpGs differed between 199 TSD-linked genes and 1,000 random subsets of 199 non-TSD-linked genes, sampled from 11,560 single-copy orthologs shared between sea turtle species. An offset of total CpG count was included in the model. Histograms are plotted for the *P* values per iteration (*n* = 1,000), for each term in the ANOVA results table: gene category (TSD-linked vs. non-TSD-linked, dark blue), genomic feature type (purple), and their interaction (gene category × feature type, yellow). The dashed line represents *P* = 0.05. In total, 171 (17.1%) tests passed *P* < 0.05 for gene category, 1,000 (100%) tests passed for feature type, and 152 (1.52%) tests passed for the interaction term.

## List of Abbreviations

5mC: 5-methylcytosine; 5hmC: 5-hydroxymethylcytosine; bp: base pairs; CBP: Canada BioGenome Project; CpG: 5′-C-phosphate-G-3, ENA: European Nucleotide Archive; Gbp: giga base pairs; kbp: kilo base pairs; Mbp: mega base pairs; Mya: million years ago; Ne: effective population size; ONT: Oxford Nanopore Technology; PSMC: pairwise sequentially Markovian coalescent; TSS: transcription start site; VGP: Vertebrate Genomes Project; WGBS: whole-genome bisulfite sequencing.

## References

[1] Ceballos G, Ehrlich PR, Barnosky AD, et al. Accelerated modern human–induced species losses: entering the sixth mass extinction. Sci Adv. 2015;1:e1400253. 10.1126/sciadv.1400253. 26601195 PMC4640606

[2] Theissinger K, Fernandes C, Formenti G, et al. How genomics can help biodiversity conservation. Trends Genet. 2023;39:545–59. 10.1016/j.tig.2023.01.005. 36801111

[3] Coates DJ, Byrne M, Moritz C. Genetic diversity and conservation units: dealing with the species-population continuum in the age of genomics. Front Ecol Evol. 2018;6:165. 10.3389/fevo.2018.00165.

[4] Wold J, Koepfli K-P, Galla SJ, et al. Expanding the conservation genomics toolbox: incorporating structural variants to enhance genomic studies for species of conservation concern. Mol Ecol. 2021;30:5949–65. 10.1111/mec.16141. 34424587 PMC9290615

[5] Kardos M, Taylor HR, Ellegren H, et al. Genomics advances the study of inbreeding depression in the wild. Evol Appl. 2016;9:1205–18. 10.1111/eva.12414. 27877200 PMC5108213

[6] Karamanlidis AA, Skrbinšek T, Amato G, et al. Genetic and demographic history define a conservation strategy for earth's most endangered pinniped, the Mediterranean monk seal Monachus monachus. Sci Rep. 2021;11:373. 10.1038/s41598-020-79712-1. 33431977 PMC7801404

[7] Flanagan SP, Forester BR, Latch EK, et al. Guidelines for planning genomic assessment and monitoring of locally adaptive variation to inform species conservation. Evol Appl. 2018;11:1035–52. 10.1111/eva.12569. 30026796 PMC6050180

[8] Eizaguirre C, Baltazar-Soares M. Evolutionary conservation—evaluating the adaptive potential of species. Evol Appl. 2014;7:963–67. 10.1111/eva.12227.

[9] Balard A, Baltazar-Soares M, Eizaguirre C, et al. An epigenetic toolbox for conservation biologists. Evol Appl. 2024;17:e13699. 10.1111/eva.13699. 38832081 PMC11146150

[10] Feil R, Fraga MF. Epigenetics and the environment: emerging patterns and implications. Nat Rev Genet. 2012;13:97–109. 10.1038/nrg3142. 22215131

[11] Rey O, Eizaguirre C, Angers B, et al. Linking epigenetics and biological conservation: towards a conservation epigenetics perspective. Funct Ecol. 2020;34:414–27. 10.1111/1365-2435.13429.

[12] Lamka GF, Harder AM, Sundaram M, et al. Epigenetics in ecology, evolution, and conservation. Front Ecol Evol. 2022;10:871791. 10.3389/fevo.2022.871791.

[13] Laine VN, Sepers B, Lindner M, et al. An ecologist's guide for studying DNA methylation variation in wild vertebrates. Mol Ecol Resour. 2023;23:1488–508. 10.1111/1755-0998.13624. 35466564

[14] IUCN . The IUCN Red List of Threatened Species (Version 2024-1). 2024. https://www.iucnredlist.org. Accessed 18 July 2024.

[15] Wallace BP, DiMatteo AD, Bolten AB, et al. Global conservation priorities for marine turtles. PLoS One. 2011;6:e24510. 10.1371/journal.pone.0024510. 21969858 PMC3182175

[16] Yntema CL, Mrosovsky N. Critical periods and pivotal temperatures for sexual differentiation in loggerhead sea turtles. Can J Zool. 1982;60:1012–16. 10.1139/z82-141.

[17] Hawkes L, Broderick A, Godfrey M, et al. Climate change and marine turtles. Endang Species Res. 2009;7:137–54. 10.3354/esr00198.

[18] Laloë J-O, Cozens J, Renom B, et al. Effects of rising temperature on the viability of an important sea turtle rookery. Nature Clim Change. 2014;4:513–18. 10.1038/nclimate2236.

[19] Mitchell N, Janzen FJ. Temperature-dependent sex determination and contemporary climate change. Sex Dev. 2010;4:129–40. 10.1159/000282494. 20145383

[20] Lockley EC, Eizaguirre C. Effects of global warming on species with temperature-dependent sex determination: bridging the gap between empirical research and management. Evol Appl. 2021;14:2361–77. 10.1111/eva.13226. 34745331 PMC8549623

[21] Komoroske LM, Jensen MP, Stewart KR, et al. Advances in the application of genetics in marine turtle biology and conservation. Front Mar Sci. 2017;4:156. 10.3389/fmars.2017.00156.

[22] Venegas D, Marmolejo-Valencia A, Valdes-Quezada C, et al. Dimorphic DNA methylation during temperature-dependent sex determination in the sea turtle Lepidochelys olivacea. Gen Comp Endocrinol. 2016;236:35–41. 10.1016/j.ygcen.2016.06.026. 27342379

[23] Ge C, Ye J, Weber C, et al. The histone demethylase KDM6B regulates temperature-dependent sex determination in a turtle species. Science. 2018;360:645–48. 10.1126/science.aap8328. 29748283

[24] Piferrer F . Epigenetic mechanisms in sex determination and in the evolutionary transitions between sexual systems. Phil Trans R Soc B. 2021;376:20200110. 10.1098/rstb.2020.0110. 34247505 PMC8273503

[25] Bentley BP, Carrasco-Valenzuela T, Ramos EKS, et al. Divergent sensory and immune gene evolution in sea turtles with contrasting demographic and life histories. Proc Natl Acad Sci U S A. 2023;120: e2201076120. 10.1073/pnas.2201076120. 36749728 PMC9962930

[26] Guo Y, Tang J, Zhuo Z, et al. The first high-quality chromosome-level genome of eretmochelys imbricata using HiFi and Hi-C data. Sci Data. 2023;10:604. 10.1038/s41597-023-02522-3. 37689728 PMC10492850

[27] Chang G, Jones S, Leelakumari S, et al. The genome sequence of the Loggerhead sea turtle, Caretta caretta Linnaeus 1758. F1000Res. 2023;12:336. 10.12688/f1000research.131283.2. 37455852 PMC10338980

[28] Thorburn D-MJ, Sagonas K, Binzer-Panchal M, et al. Origin matters: using a local reference genome improves measures in population genomics. Mol Ecol Resour. 2023;23:1706–23. 10.1111/1755-0998.13838. 37489282

[29] Taxonera A, Fairweather K, Jesus A, et al. Cabo Verde: sea turtles “in abundance”. In: SWOT report—state of the world's sea turtlesVol. XVII. San Francisco: Oceanic Society; 2022. https://www.seaturtlestatus.org/articles/cabo-verde-sea-turtles-in-abundance. Accessed 29 June 2023.

[30] Stiebens VA, Merino SE, Roder C, et al. Living on the edge: how philopatry maintains adaptive potential. Proc R Soc B. 2013;280:20130305. 10.1098/rspb.2013.0305. PMC3774223 23720544

[31] Owens DWM, Ruiz GJ. New methods of obtaining blood and cerebrospinal fluid from marine turtles. Herpetologica. 1980;36:17–20. https://www.jstor.org/stable/3891847.

[32] Zhang T, Li H, Ma S, et al. The newest Oxford Nanopore R10.4.1 full-length 16S rRNA sequencing enables the accurate resolution of species-level microbial community profiling. Appl Environ Microb. 2023;89:e00605–23. 10.1128/aem.00605-23. PMC10617388 37800969

[33] Wick RR, Judd LM, Holt KE. Performance of neural network basecalling tools for Oxford Nanopore sequencing. Genome Biol. 2019;20:129. 10.1186/s13059-019-1727-y. 31234903 PMC6591954

[34] King T, Butcher S, Zalewski L. Apocrita—high performance computing cluster for Queen Mary University of London. Zenodo. 2017. 10.5281/ZENODO.438045. Accessed 28 August 2024.

[35] Wick RR . 2018. PoreChop (Version 0.2.4). https://github.com/rrwick/Porechop/releases/tag/v0.2.4. Accessed July 2024.

[36] Filipović I, Rašić G, Hereward J, et al. A high-quality de novo genome assembly based on nanopore sequencing of a wild-caught coconut rhinoceros beetle (Oryctes rhinoceros). BMC Genomics. 2022;23:426. 10.1186/s12864-022-08628-z. 35672676 PMC9172067

[37] De Coster W, D'Hert S, Schultz DT, et al. NanoPack: visualizing and processing long-read sequencing data. Bioinformatics. 2018;34:2666–69. 10.1093/bioinformatics/bty149. 29547981 PMC6061794

[38] Vurture GW, Sedlazeck FJ, Nattestad M, et al. GenomeScope: fast reference-free genome profiling from short reads. Bioinformatics. 2017;33:2202–4. 10.1093/bioinformatics/btx153. 28369201 PMC5870704

[39] Krueger F . 2019. TrimGalore (Version 0.6.5). https://github.com/FelixKrueger/TrimGalore/releases/tag/0.6.5. Accessed July 2024.

[40] Kolmogorov M, Yuan J, Lin Y, et al. Assembly of long, error-prone reads using repeat graphs. Nat Biotechnol. 2019;37:540–46. 10.1038/s41587-019-0072-8. 30936562

[41] Oxford Nanopore Technologies . 2021. Medaka (Version 1.3.3). https://github.com/nanoporetech/medaka/releases/tag/v1.3.3. Accessed 1 July 2024.

[42] Walker BJ, Abeel T, Shea T, et al. Pilon: an integrated tool for comprehensive microbial variant detection and genome assembly improvement. PLoS One. 2014;9:e112963. 10.1371/journal.pone.0112963. 25409509 PMC4237348

[43] Laetsch DR, Blaxter ML. BlobTools: interrogation of genome assemblies. F1000Res. 2017;6:1287. 10.12688/f1000research.12232.1.

[44] Buchfink B, Xie C, Huson DH. Fast and sensitive protein alignment using DIAMOND. Nat Methods. 2015;12:59–60. 10.1038/nmeth.3176. 25402007

[45] The UniProt Consortium . UniProt: the universal protein knowledgebase in 2021. Nucleic Acids Res. 2021;49:D480–89. 10.1093/nar/gkaa1100. 33237286 PMC7778908

[46] Guan D, McCarthy SA, Wood J, et al. Identifying and removing haplotypic duplication in primary genome assemblies. Bioinformatics. 2020;36:2896–98. 10.1093/bioinformatics/btaa025. 31971576 PMC7203741

[47] Meng G, Li Y, Yang C, et al. MitoZ: a toolkit for animal mitochondrial genome assembly, annotation and visualization. Nucleic Acids Res. 2019;47:e63. 10.1093/nar/gkz173. 30864657 PMC6582343

[48] Li H, Handsaker B, Wysoker A, et al. The sequence alignment/map format and SAMtools. Bioinformatics. 2009;25:2078–79. 10.1093/bioinformatics/btp352. 19505943 PMC2723002

[49] Alonge M, Lebeigle L, Kirsche M, et al. Automated assembly scaffolding using RagTag elevates a new tomato system for high-throughput genome editing. Genome Biol. 2022;23:258. 10.1186/s13059-022-02823-7. 36522651 PMC9753292

[50] Gurevich A, Saveliev V, Vyahhi N, et al. QUAST: quality assessment tool for genome assemblies. Bioinformatics. 2013;29:1072–75. 10.1093/bioinformatics/btt086. 23422339 PMC3624806

[51] Simão FA, Waterhouse RM, Ioannidis P, et al. BUSCO: assessing genome assembly and annotation completeness with single-copy orthologs. Bioinformatics. 2015;31:3210–12. 10.1093/bioinformatics/btv351. 26059717

[52] Mapleson D, Garcia Accinelli G, Kettleborough G, et al. KAT: a K-mer analysis toolkit to quality control NGS datasets and genome assemblies. Bioinformatics. 2017;33:574–76. 10.1093/bioinformatics/btw663. 27797770 PMC5408915

[53] Rhie A, Walenz BP, Koren S, et al. Merqury: reference-free quality, completeness, and phasing assessment for genome assemblies. Genome Biol. 2020;21:245. 10.1186/s13059-020-02134-9. 32928274 PMC7488777

[54] Wang Z, Pascual-Anaya J, Zadissa A, et al. The draft genomes of soft-shell turtle and green sea turtle yield insights into the development and evolution of the turtle-specific body plan. Nat Genet. 2013;45:701–6. 10.1038/ng.2615. 23624526 PMC4000948

[55] Flynn JM, Hubley R, Goubert C, et al. RepeatModeler2 for automated genomic discovery of transposable element families. Proc Natl Acad Sci U S A. 2020;117:9451–57. 10.1073/pnas.1921046117. 32300014 PMC7196820

[56] Abrusán G, Grundmann N, DeMester L, et al. TEclass—a tool for automated classification of unknown eukaryotic transposable elements. Bioinformatics. 2009;25:1329–1330. 10.1093/bioinformatics/btp084. 19349283

[57] Smit AFA, Hubley R, Green P. 2022. RepeatMasker (Version 4.1.4). http://www.repeatmasker.org. Accessed 1 July 2024.

[58] Leinonen R, Sugawara H, Shumway M. The Sequence Read Archive. Nucleic Acids Res. 2011;39:D19–D21. 10.1093/nar/gkq1019. 21062823 PMC3013647

[59] Banerjee SM, Stoll JA, Allen CD, et al. Species and population specific gene expression in blood transcriptomes of marine turtles. BMC Genomics. 2021;22:346. 10.1186/s12864-021-07656-5. 33985425 PMC8117300

[60] Chow JC, Anderson PE, Shedlock AM. Sea turtle population genomic discovery: global and locus-specific signatures of polymorphism, selection, and adaptive potential. Genome Biol Evolut. 2019;11:2797–806. 10.1093/gbe/evz190. PMC6786478 31504487

[61] Hernández-Fernández J, Pinzón Velasco AM, López Barrera EA, et al. De novo assembly and functional annotation of blood transcriptome of loggerhead turtle, and in silico characterization of peroxiredoxins and thioredoxins. PeerJ. 2021;9:e12395. 10.7717/peerj.12395. 34820176 PMC8606161

[62] Bolger AM, Lohse M, Usadel B. Trimmomatic: a flexible trimmer for Illumina sequence data. Bioinformatics. 2014;30:2114–20. 10.1093/bioinformatics/btu170. 24695404 PMC4103590

[63] Dobin A, Davis CA, Schlesinger F, et al. STAR: ultrafast universal RNA-seq aligner. Bioinformatics. 2013;29:15–21. 10.1093/bioinformatics/bts635. 23104886 PMC3530905

[64] Hoff KJ, Lange S, Lomsadze A, et al. BRAKER1: unsupervised RNA-seq-based genome annotation with GeneMark-ET and AUGUSTUS. Bioinformatics. 2016;32:767–69. 10.1093/bioinformatics/btv661. 26559507 PMC6078167

[65] Venturini L, Caim S, Kaithakottil GG, et al. Leveraging multiple transcriptome assembly methods for improved gene structure annotation. Gigascience. 2018;7:giy093. 10.1093/gigascience/giy093. PMC6105091 30052957

[66] Grabherr MG, Haas BJ, Yassour M, et al. Trinity: reconstructing a full-length transcriptome without a genome from RNA-seq data. Nat Biotechnol. 2011;29:644–52. 10.1038/nbt.1883. 21572440 PMC3571712

[67] Wu TD, Watanabe CK. GMAP: a genomic mapping and alignment program for mRNA and EST sequences. Bioinformatics. 2005;21:1859–75. 10.1093/bioinformatics/bti310. 15728110

[68] Mapleson D, Venturini L, Kaithakottil G, et al. Efficient and accurate detection of splice junctions from RNA-seq with Portcullis. Gigascience. 2018;7:giy131. 10.1093/gigascience/giy131. PMC6302956 30418570

[69] Haas BJ . 2018. TransDecoder (Version 5.5.0). https://github.com/TransDecoder/TransDecoder/releases/tag/TransDecoder-v5.5.0. Accessed 1 July 2024.

[70] Haas BJ, Salzberg SL, Zhu W, et al. Automated eukaryotic gene structure annotation using EVidenceModeler and the Program to assemble spliced alignments. Genome Biol. 2008;9:R7. 10.1186/gb-2008-9-1-r7. 18190707 PMC2395244

[71] Dainat J . AGAT: another Gff analysis toolkit to handle annotations in any GTF/GFF format (Version 0.9.1). Zenodo. 2022. 10.5281/zenodo.6488306.

[72] Pertea G, Pertea M. GFF utilities: gffRead and GffCompare. F1000Res. 2020;9:304. 10.12688/f1000research.23297.2. PMC7222033 32489650

[73] Jones P, Binns D, Chang H-Y, et al. InterProScan 5: genome-scale protein function classification. Bioinformatics. 2014;30:1236–40. 10.1093/bioinformatics/btu031. 24451626 PMC3998142

[74] Cantarel BL, Korf I, Robb SMC, et al. MAKER: an easy-to-use annotation pipeline designed for emerging model organism genomes. Genome Res. 2008;18:188–96. 10.1101/gr.6743907. 18025269 PMC2134774

[75] Klughammer J, Romanovskaia D, Nemc A, et al. Comparative analysis of genome-scale, base-resolution DNA methylation profiles across 580 animal species. Nat Commun. 2023;14:232. 10.1038/s41467-022-34828-y. 36646694 PMC9842680

[76] Oxford Nanopore Technologies . 2023. ModKit (Version 0.1.9). https://github.com/nanoporetech/modkit/releases/tag/v0.1.9. Accessed 1 July 2024.

[77] Krueger F, Andrews SR. Bismark: a flexible aligner and methylation caller for bisulfite-seq applications. Bioinformatics. 2011;27:1571–72. 10.1093/bioinformatics/btr167. 21493656 PMC3102221

[78] Cristofari R . 2023. merge_CpG.py. https://github.com/rcristofari/penguin-tools/blob/master/merge_CpG.py. Accessed 30 April 2023.

[79] Akalin A, Kormaksson M, Li S, et al. methylKit: a comprehensive R package for the analysis of genome-wide DNA methylation profiles. Genome Biol. 2012;13:R87. 10.1186/gb-2012-13-10-r87. 23034086 PMC3491415

[80] Wreczycka K, Gosdschan A, Yusuf D, et al. Strategies for analyzing bisulfite sequencing data. J Biotechnol. 2017;261:105–15. 10.1016/j.jbiotec.2017.08.007. 28822795

[81] Jones PA . Functions of DNA methylation: islands, start sites, gene bodies and beyond. Nat Rev Genet. 2012;13:484–92. 10.1038/nrg3230. 22641018

[82] Akalin A, Franke V, Vlahoviček K, et al. genomation: a toolkit to summarize, annotate and visualize genomic intervals. Bioinformatics. 2015;31:1127–29. 10.1093/bioinformatics/btu775. 25417204

[83] Lawrence M, Huber W, Pagès H, et al. Software for computing and annotating genomic ranges. PLoS Comput Biol. 2013;9:e1003118. 10.1371/journal.pcbi.1003118. 23950696 PMC3738458

[84] Heckwolf MJ, Meyer BS, Häsler R, et al. Two different epigenetic information channels in wild three-spined sticklebacks are involved in salinity adaptation. Sci Adv. 2020;6:eaaz1138. 10.1126/sciadv.aaz1138. 32219167 PMC7083608

[85] Sagonas K, Meyer BS, Kaufmann J, et al. Experimental parasite infection causes genome-wide changes in DNA methylation. Mol Biol Evol. 2020;37:2287–99. 10.1093/molbev/msaa084. 32227215 PMC7531312

[86] R Core Team . R: a language and environment for statistical computing. Vienna, Austria: R Foundation for Statistical Computing; 2021. https://www.R-project.org/.

[87] Wickham H . ggplot2: elegant graphics for data analysis. New York, NY: Springer; 2016. 10.1007/978-3-319-24277-4.

[88] Li H . Minimap2: pairwise alignment for nucleotide sequences. Bioinformatics. 2018;34:3094–100. 10.1093/bioinformatics/bty191. 29750242 PMC6137996

[89] Cabanettes F, Klopp C. D-GENIES: dot plot large genomes in an interactive, efficient and simple way. PeerJ. 2018;6:e4958. 10.7717/peerj.4958. 29888139 PMC5991294

[90] Li H, Durbin R. Inference of human population history from whole genome sequence of a single individual. Nature. 2011;475:493–96. 10.1038/nature10231. 21753753 PMC3154645

[91] Morin PA, Archer FI, Avila CD, et al. Reference genome and demographic history of the most endangered marine mammal, the vaquita. Mol Ecol Resour. 2021;21:1008–20. 10.1111/1755-0998.13284. 33089966 PMC8247363

[92] Vilaça ST, Piccinno R, Rota-Stabelli O, et al. Divergence and hybridization in sea turtles: inferences from genome data show evidence of ancient gene flow between species. Mol Ecol. 2021;30:6178–92. 10.1111/mec.16113. 34390061 PMC9292604

[93] Li H . Aligning sequence reads, clone sequences and assembly contigs with BWA-MEM. arXiv. 2013. https://10.48550/arXiv.1303.3997.

[94] Broad Institute . Picard (Version 2.26.9). https://broadinstitute.github.io/picard. Accessed 31 January 2022.

[95] Danecek P, Bonfield JK, Liddle J, et al. Twelve years of SAMtools and BCFtools. Gigascience. 2021;10:giab008. 10.1093/gigascience/giab008. PMC7931819 33590861

[96] Piovano S, Clusa M, Carreras C, et al. Different growth rates between loggerhead sea turtles (Caretta caretta) of Mediterranean and Atlantic origin in the Mediterranean Sea. Mar Biol. 2011;158:2577–87. 10.1007/s00227-011-1759-7.

[97] Hansen J, Sato M, Russell G, et al. Climate sensitivity, sea level and atmospheric carbon dioxide. Phil Trans R Soc A. 2013;371:1–31. 10.1098/rsta.2012.0294. PMC3785813 24043864

[98] Clark PU, Shakun JD, Rosenthal Y, et al. Global and regional temperature change over the past 4.5 million years. Science. 2024;383:884–90. 10.1126/science.adi1908. 38386742

[99] Robinson JA, Räikkönen J, Vucetich LM, et al. Genomic signatures of extensive inbreeding in Isle Royale wolves, a population on the threshold of extinction. Sci Adv. 2019;5:eaau0757. 10.1126/sciadv.aau0757. 31149628 PMC6541468

[100] McKenna A, Hanna M, Banks E, et al. The Genome Analysis Toolkit: a MapReduce framework for analyzing next-generation DNA sequencing data. Genome Res. 2010;20:1297–303. 10.1101/gr.107524.110. 20644199 PMC2928508

[101] Martin SH . 2016. popgenWindows. https://github.com/simonhmartin/genomics_general/blob/master/popgenWindows.py. Accessed 30 May 2022.

[102] Machado CRD, Domit C, Pucci MB, et al. Heterochromatin and microsatellites detection in karyotypes of four sea turtle species: interspecific chromosomal differences. Genet Mol Biol. 2020;43:e20200213. 10.1590/1678-4685-GMB-2020-0213. 33270075 PMC7734918

[103] Altschul SF, Gish W, Miller W, et al. Basic local alignment search tool. J Mol Biol. 1990;215:403–10. 10.1016/S0022-2836(05)80360-2. 2231712

[104] Gu Z, Gu L, Eils R, et al. circlize implements and enhances circular visualization in R. Bioinformatics. 2014;30:2811–12. 10.1093/bioinformatics/btu393. 24930139

[105] Emms DM, Kelly S. OrthoFinder: solving fundamental biases in whole genome comparisons dramatically improves orthogroup inference accuracy. Genome Biol. 2015;16:157. 10.1186/s13059-015-0721-2. 26243257 PMC4531804

[106] Szklarczyk D, Gable AL, Nastou KC, et al. The STRING database in 2021: customizable protein–protein networks, and functional characterization of user-uploaded gene/measurement sets. Nucleic Acids Res. 2021;49:D605–12. 10.1093/nar/gkaa1074. 33237311 PMC7779004

[107] Guigó R . Genome annotation: from human genetics to biodiversity genomics. Cell Genomics. 2023;3:100375. 10.1016/j.xgen.2023.100375. 37601977 PMC10435374

[108] Olova N, Krueger F, Andrews S, et al. Comparison of whole-genome bisulfite sequencing library preparation strategies identifies sources of biases affecting DNA methylation data. Genome Biol. 2018;19:33. 10.1186/s13059-018-1408-2. 29544553 PMC5856372

[109] Suzuki MM, Bird A. DNA methylation landscapes: provocative insights from epigenomics. Nat Rev Genet. 2008;9:465–76. 10.1038/nrg2341. 18463664

[110] Liu Y, Rosikiewicz W, Pan Z, et al. DNA methylation-calling tools for Oxford Nanopore sequencing: a survey and human epigenome-wide evaluation. Genome Biol. 2021;22:295. 10.1186/s13059-021-02510-z. 34663425 PMC8524990

[111] Liu X, Ni Y, Wang D, et al. Unraveling the whole genome DNA methylation profile of zebrafish kidney marrow by Oxford Nanopore sequencing. Sci Data. 2023;10:532. 10.1038/s41597-023-02431-5. 37563176 PMC10415270

[112] Gombert S, Jahn K, Pathak H, et al. Comparison of methylation estimates obtained via MinION nanopore sequencing and sanger bisulfite sequencing in the TRPA1 promoter region. BMC Med Genomics. 2023;16:257. 10.1186/s12920-023-01694-6. 37872581 PMC10591399

[113] Sigurpalsdottir BD, Stefansson OA, Holley G, et al. A comparison of methods for detecting DNA methylation from long-read sequencing of human genomes. Genome Biol. 2024;25:69. 10.1186/s13059-024-03207-9. 38468278 PMC10929077

[114] Ni Y, Liu X, Simeneh ZM, et al. Benchmarking of Nanopore R10.4 and R9.4.1 flow cells in single-cell whole-genome amplification and whole-genome shotgun sequencing. Comput Struct Biotechnol J. 2023;21:2352–64. 10.1016/j.csbj.2023.03.038. 37025654 PMC10070092

[115] Simpson JT, Workman RE, Zuzarte PC, et al. Detecting DNA cytosine methylation using nanopore sequencing. Nat Methods. 2017;14:407–10. 10.1038/nmeth.4184. 28218898

[116] Xu L, Seki M. Recent advances in the detection of base modifications using the Nanopore sequencer. J Hum Genet. 2020;65:25–33. 10.1038/s10038-019-0679-0. 31602005 PMC7087776

[117] Shen L, Zhang Y. 5-Hydroxymethylcytosine: generation, fate, and genomic distribution. Curr Opin Cell Biol. 2013;25:289–96. 10.1016/j.ceb.2013.02.017. 23498661 PMC4060438

[118] Martínez-Botí MA, Foster GL, Chalk TB, et al. Plio-pleistocene climate sensitivity evaluated using high-resolution CO2 records. Nature. 2015;518:49–54. 10.1038/nature14145. 25652996

[119] McClymont EL, Ho SL, Ford HL, et al. Climate evolution through the onset and intensification of Northern Hemisphere glaciation. Rev Geophys. 2023;61:e2022RG000793. 10.1029/2022RG000793.

[120] Ford HL, Chalk TB. SIDEBAR. The mid-pleistocene Enigma. Oceanog. 2020;33:101–103. 10.5670/oceanog.2020.216.

[121] Lawrence KT, Sigman DM, Herbert TD, et al. Time-transgressive North Atlantic productivity changes upon Northern Hemisphere glaciation. Paleoceanography. 2013;28:740–51. 10.1002/2013PA002546.

[122] Lamy F, Winckler G, Arz HW, et al. Five million years of antarctic circumpolar current strength variability. Nature. 2024;627:789–96. 10.1038/s41586-024-07143-3. 38538940 PMC10972744

[123] Baltazar-Soares M, Klein JD, Correia SM, et al. Distribution of genetic diversity reveals colonization patterns and philopatry of the loggerhead sea turtles across geographic scales. Sci Rep. 2020;10:18001. 10.1038/s41598-020-74141-6. 33093463 PMC7583243

[124] McQueen HA, Fantes J, Cross SH, et al. CpG islands of chicken are concentrated on microchromosomes. Nat Genet. 1996;12:321–24. 10.1038/ng0396-321. 8589727

[125] Waters PD, Patel HR, Ruiz-Herrera A, et al. Microchromosomes are building blocks of bird, reptile, and mammal chromosomes. Proc Natl Acad Sci U S A. 2021;118:e2112494118. 10.1073/pnas.2112494118. 34725164 PMC8609325

[126] Papin C, Le Gras S, Ibrahim A, et al. CpG islands shape the epigenome landscape. J Mol Biol. 2021;433:166659. 10.1016/j.jmb.2020.09.018. 33010306

[127] Ziller MJ, Gu H, Müller F, et al. Charting a dynamic DNA methylation landscape of the human genome. Nature. 2013;500:477–81. 10.1038/nature12433. 23925113 PMC3821869

[128] Hoelzel AR, Bruford MW, Fleischer RC. Conservation of adaptive potential and functional diversity. Conserv Genet. 2019;20:1–5. 10.1007/s10592-019-01151-x.

[129] Harewood L, Fraser P. The impact of chromosomal rearrangements on regulation of gene expression. Hum Mol Genet. 2014;23:R76–R82. 10.1093/hmg/ddu278. 24907073

[130] Valenzuela N, Adams DC. Chromosome number and sex determination coevolve in turtles. Evolution. 2011;65:1808–13. 10.1111/j.1558-5646.2011.01258.x. 21644965

[131] Lee L, Montiel EE, Navarro-Domínguez BM, et al. Chromosomal rearrangements during turtle evolution altered the synteny of genes involved in vertebrate sex determination. Cytogenet Genome Res. 2019;157:77–88. 10.1159/000497302. 30808820

[132] Schroeder AL, Metzger KJ, Miller A, et al. A novel candidate gene for temperature-dependent sex determination in the common snapping turtle. Genetics. 2016;203:557–71. 10.1534/genetics.115.182840. 26936926 PMC4858799

[133] Elango N, Yi SV. DNA methylation and structural and functional bimodality of vertebrate promoters. Mol Biol Evol. 2008;25:1602–8. 10.1093/molbev/msn110. 18469331

[134] Keller TE, Han P, Yi SV. Evolutionary transition of promoter and gene body DNA methylation across invertebrate–vertebrate boundary. Mol Biol Evol. 2016;33:1019–28. 10.1093/molbev/msv345. 26715626 PMC4776710

[135] De Paoli-Iseppi R, Deagle BE, McMahon CR, et al. Measuring animal age with DNA methylation: from humans to wild animals. Front Genet. 2017;8:106. 10.3389/fgene.2017.00106. 28878806 PMC5572392

[136] Viitaniemi HM, Verhagen I, Visser ME, et al. Seasonal variation in genome-wide DNA methylation patterns and the onset of seasonal timing of reproduction in great tits. Genome Biol Evol. 2019;11:970–83. 10.1093/gbe/evz044. 30840074 PMC6447391

[137] Grant OA, Wang Y, Kumari M, et al. Characterising sex differences of autosomal DNA methylation in whole blood using the Illumina EPIC array. Clin Epigenet. 2022;14:62. 10.1186/s13148-022-01279-7. PMC9107695 35568878

[138] Yousefi PD, Suderman M, Langdon R, et al. DNA methylation-based predictors of health: applications and statistical considerations. Nat Rev Genet. 2022;23:369–83. 10.1038/s41576-022-00465-w. 35304597

[139] Xu R, Li S, Guo S, et al. Environmental temperature and human epigenetic modifications: a systematic review. Environ Pollut. 2020;259:113840. 10.1016/j.envpol.2019.113840. 31884209

[140] Mäkinen H, Van Oers K, Eeva T, et al. The effect of experimental lead pollution on DNA methylation in a wild bird population. Epigenetics. 2022;17:625–41. 10.1080/15592294.2021.1943863. 34369261 PMC9235896

[141] Yen EC, Gilbert JD, Balard A, et al. DNA methylation carries signatures of sublethal effects under thermal stress in loggerhead sea turtles. Evol Appl. 2024;17:e70013. 10.1111/eva.70013. 39286762 PMC11403127

[142] Bock SL, Smaga CR, McCoy JA, et al. Genome-wide DNA methylation patterns harbour signatures of hatchling sex and past incubation temperature in a species with environmental sex determination. Mol Ecol. 2022;31:5487–505. 10.1111/mec.16670. 35997618 PMC9826120

[143] Mork L, Capel B. Conserved action of β-catenin during female fate determination in the red-eared slider turtle. Evol Dev. 2013;15:96–106. 10.1111/ede.12020. 25098635

[144] Liu J, Xiao Q, Xiao J, et al. Wnt/β-catenin signalling: function, biological mechanisms, and therapeutic opportunities. Sig Transduct Target Ther. 2022;7:1–23. 10.1038/s41392-021-00762-6. PMC8724284 34980884

[145] Tezak B, Straková B, Fullard DJ, et al. Higher temperatures directly increase germ cell number, promoting feminization of red-eared slider turtles. Curr Biol. 2023;33:3017–23.e2. 10.1016/j.cub.2023.06.008. 37354900

[146] Rhen T, Schroeder A. Molecular mechanisms of sex determination in reptiles. Sex Dev. 2010;4:16–28. 10.1159/000282495. 20145384 PMC2918650

[147] Kent J, Wheatley SC, Andrews JE, et al. A male-specific role for SOX9 in vertebrate sex determination. Development. 1996;122:2813–22. 10.1242/dev.122.9.2813. 8787755

[148] Moreno-Mendoza N, Harley VR, et al. Temperature regulates SOX9 expression in cultured gonads of *lepidochelys olivacea*, a species with temperature sex determination. Dev Biol. 2001;229:319–26. 10.1006/dbio.2000.9952. 11150238

[149] Kohno S, Katsu Y, Urushitani H, et al. Potential contributions of heat shock proteins to temperature-dependent sex determination in the American alligator. Sex Dev. 2010;4:73–87. 10.1159/000260374. 19940440 PMC2855287

[150] Capel B . Vertebrate sex determination: evolutionary plasticity of a fundamental switch. Nat Rev Genet. 2017;18:675–89. 10.1038/nrg.2017.60. 28804140

[151] Yen EC . 2024. Article_CarCar_GenomeAssembly. https://github.com/eugeniecyen/Article_CarCar_GenomeAssembly. Accessed 3 April 2025.

[152] Yen EC, Gilbert JD, Balard A, et al. Supporting data for “Chromosome-Level Genome Assembly and Methylome Profile Yield Insights for the Conservation of Endangered Loggerhead Sea Turtles.” GigaScience Database. 2025. 10.5524/102690. PMC12143204 40478729

