# Supplementary material for: Chromosome-level genome assembly and methylome profile yield insights for the conservation of endangered loggerhead sea turtles
Source: Gigascience. 2025 Jun 6;14:giaf054. doi: 10.1093/gigascience/giaf054 (PMC12143204; doi:10.1093/gigascience/giaf054)
Supplement: giaf054_Supplemental_File [file giaf054_supplemental_file.docx]

**Supplementary Material**

**Chromosome-level genome assembly and methylome profile yield insights for conservation of endangered loggerhead sea turtles**

**Authors**

Eugenie C. Yen^1*^, James D. Gilbert^1^, Alice Balard^1^, Albert Taxonera^2^, Kirsten Fairweather^2^, Heather L. Ford^3^, Doko-Miles Thorburn^1^, Stephen J. Rossiter^1^, José M. Martín-Durán^1^, Christophe Eizaguirre^1^

**Affiliations**

^1^ School of Biological and Behavioural Sciences, Queen Mary University of London, London, E1 4DQ, United Kingdom

^2^ Project Biodiversity, Mercado Municipal, local 22 Santa Maria, Ilha do Sal, Cabo Verde

^3^ School of Geography, Queen Mary University of London, London, E1 4DQ, United Kingdom

^*^ **Corresponding Author:** Eugenie C. Yen. School of Biological and Behavioural Sciences, G.E. Fogg Building, Mile End Road, Queen Mary University of London, London, E1 4DQ, UK. Email: [e.yen[at]qmul.ac.uk](mailto:e.yen@qmul.ac.uk)

| **BioProject** | **SRA Run** | **BioSample** | **Associated publication** | **Tissue** | **Life stage** | **Sex** | **Treatment** | **Raw bases (Gbp)** | **Raw reads** | **Passed reads** | **Hint type generated** |
| --- | --- | --- | --- | --- | --- | --- | --- | --- | --- | --- | --- |
| PRJNA663187 | SRR12630873 | SAMN16122281 | Chow et al. (2021) | Gonad | Hatchling | Female | None | 10.2 | 50746307 | 49954134 (98.44%) | Transcript |
| PRJNA663187 | SRR12630874 | SAMN16122280 | Chow et al. (2021) | Gonad | Hatchling | Female | None | 10.3 | 51399125 | 50152916 (97.58%) | Transcript |
| PRJNA663187 | SRR12630879 | SAMN16122282 | Chow et al. (2021) | Gonad | Hatchling | Male | None | 9.3 | 46557205 | 45820268 (98.42%) | Transcript, transcriptome |
| PRJNA663187 | SRR12630881 | SAMN16122283 | Chow et al. (2021) | Gonad | Hatchling | Male | None | 2.2 | 11018312 | 10879472 (98.74%) | Transcript |
| PRJNA560561 | SRR10032986 | SAMN12591173 | Hernández-Fernández et al. (2021) | Blood | Hatchling | Unknown | None | 15.7 | 77607764 | 76009322 (97.94%) | Transcript |
| PRJNA560561 | SRR10032987 | SAMN12591166 | Hernández-Fernández et al. (2021) | Blood | Hatchling | Unknown | None | 16.2 | 80140038 | 78357616 (97.78%) | Transcript |
| PRJNA560561 | SRR10032988 | SAMN12591165 | Hernández-Fernández et al. (2021) | Blood | Hatchling | Unknown | None | 16.4 | 81364511 | 79461565 (97.66%) | Transcript |
| PRJNA560561 | SRR10032989 | SAMN12591164 | Hernández-Fernández et al. (2021) | Blood | Adult | Female | None | 5.8 | 28500836 | 27552342 (96.67%) | Transcript |
| PRJNA560561 | SRR10032990 | SAMN12591163 | Hernández-Fernández et al. (2021) | Blood | Adult | Male | None | 5.6 | 27498122 | 26591514 (96.70%) | Transcript |
| PRJNA560561 | SRR10032991 | SAMN12591162 | Hernández-Fernández et al. (2021) | Blood | Juvenile | Female | None | 5 | 24727270 | 23791771 (96.22%) | Transcript |
| PRJNA560561 | SRR10032992 | SAMN12591161 | Hernández-Fernández et al. (2021) | Blood | Juvenile | Female | None | 5.4 | 26568011 | 25725953 (96.83%) | Transcript |
| PRJNA560561 | SRR10032993 | SAMN06350885 | Hernández-Fernández et al. (2021) | Blood | Juvenile | Male | None | 5.4 | 26826849 | 25946591 (96.72%) | Transcript |
| PRJNA649079 | SRR12335440 | SAMN15657957 | NA | Brain | Hatchling | Unknown | None | 3.2 | 10443220 | 9989577 (95.66%) | Transcript, transcriptome |
| PRJNA649079 | SRR12335451 | SAMN15657956 | NA | Heart | Hatchling | Unknown | None | 2.2 | 7281215 | 6917002 (95.00%) | Transcript, transcriptome |
| PRJNA649079 | SRR12335462 | SAMN15657955 | NA | Brain | Hatchling | Unknown | None | 10.7 | 35334430 | 33529137 (94.89%) | Transcript |
| PRJNA649079 | SRR12335473 | SAMN15657962 | NA | Heart | Hatchling | Unknown | Heat shock | 6 | 19906468 | 19075205 (95.82%) | Transcript |
| PRJNA649079 | SRR12335484 | SAMN15657961 | NA | Brain | Hatchling | Unknown | Heat shock | 5.8 | 19324895 | 18544037 (95.96%) | Transcript |
| PRJNA649079 | SRR12335506 | SAMN15657959 | NA | Brain | Hatchling | Unknown | Heat shock | 3.8 | 12717687 | 12169458 (95.69%) | Transcript |
| PRJNA649079 | SRR12335527 | SAMN15657964 | NA | Heart | Hatchling | Unknown | Heat shock | 2.6 | 8671833 | 8296669 (95.67%) | Transcript |
| PRJNA649079 | SRR12335528 | SAMN15657963 | NA | Brain | Hatchling | Unknown | Heat shock | 4.1 | 13668130 | 13062514 (95.57%) | Transcript |
| PRJNA649079 | SRR12335529 | SAMN15657954 | NA | Heart | Hatchling | Unknown | None | 1.7 | 5736034 | 5441041 (94.86%) | Transcript |
| PRJNA649079 | SRR12335530 | SAMN15657953 | NA | Brain | Hatchling | Unknown | None | 7.7 | 25603564 | 24423753 (95.39%) | Transcript |
| PRJNA339812 | SRR5330501 | SAMN06350885 | Hernández-Fernández et al. (2017) | Blood | Juvenile | Female | None | 5.4 | 26826849 | 25998957 (96.91%) | Transcript |
| PRJNA660024 | SRR12540984 | SAMN15933003 | Banerjee et al. (2021) | Blood | Unknown | Unknown | None | 8.3 | 27664060 | 23059350 (83.35%) | Transcript |

**Table S1. Publicly available RNA-Seq reads mined for genome annotation.** Reads were downloaded from the NCBI Sequence Read Archive (Leinonen, Sugawara and Shumway, 2011). These comprised of 746,132,735 reads from 24 loggerheads across three life stages (hatchling, juvenile and adult), four tissue types (blood, gonad, brain and heart) and both sexes.

| **Sample ID** | **Date** | **Locality** | **Island** | **Country** | **Latitude** | **Longitude** |
| --- | --- | --- | --- | --- | --- | --- |
| SLL063 | 29/07/2021 | Algodoeiro Beach | Sal | Cabo Verde | 16.62028 | -22.92938 |
| SLL065 | 29/07/2021 | Algodoeiro Beach | Sal | Cabo Verde | 16.62042 | -22.92945 |
| SLL142 | 29/07/2021 | Algodoeiro Beach | Sal | Cabo Verde | 16.61960 | -22.92927 |
| SLL143 | 29/07/2021 | Algodoeiro Beach | Sal | Cabo Verde | 16.61625 | -22.92858 |
| SLL144 | 29/07/2021 | Algodoeiro Beach | Sal | Cabo Verde | 16.61834 | -22.55732 |
| SLL146 | 29/07/2021 | Algodoeiro Beach | Sal | Cabo Verde | 16.62004 | -22.92929 |
| SLL171 | 29/07/2021 | Algodoeiro Beach | Sal | Cabo Verde | 16.61585 | -22.92860 |
| SLL176 | 29/07/2021 | Algodoeiro Beach | Sal | Cabo Verde | 16.61627 | -22.92860 |
| SLL188 | 29/07/2021 | Algodoeiro Beach | Sal | Cabo Verde | 16.61597 | -22.92858 |
| SLL189 | 29/07/2021 | Algodoeiro Beach | Sal | Cabo Verde | 16.61633 | -22.92857 |

**Table S2. Metadata for ten nesting loggerheads sampled for WGBS.**

| **Sample ID** | **Total read pairs** | **Mean mapping efficiency (%)** | **Bisulfite conversion efficiency (%)** | **Total CpGs after de-stranding** | **Mean CpG coverage after de-stranding** | **Total CpGs overlapping with ONT dataset** | **Mean methylation (%)** | **Highly methylated (>70%)**  **CpGs (%)** |
| --- | --- | --- | --- | --- | --- | --- | --- | --- |
| SLL063 | 132,232,345 | 79.0 | 99.30 | 25,445,337 | 9.07 | 16,320,919 | 75.2 | 74.4 |
| SLL065 | 132,185,458 | 82.2 | 99.38 | 25,509,991 | 9.58 | 17,094,778 | 75.6 | 74.8 |
| SLL142 | 132,204,488 | 82.6 | 99.39 | 25,492,037 | 9.36 | 16,869,208 | 74.3 | 72.8 |
| SLL143 | 132,161,944 | 84.3 | 99.37 | 25,519,785 | 9.70 | 17,091,793 | 75.2 | 73.7 |
| SLL144 | 132,219,979 | 81.6 | 99.38 | 25,499,082 | 9.47 | 17,350,101 | 74.8 | 73.7 |
| SLL146 | 132,165,428 | 77.0 | 99.36 | 25,501,839 | 9.20 | 16,619,463 | 76.7 | 77.0 |
| SLL171 | 132,186,272 | 78.5 | 99.47 | 25,417,602 | 8.90 | 17,923,159 | 76.3 | 77.2 |
| SLL176 | 132,158,064 | 76.0 | 99.43 | 25,501,178 | 8.99 | 16,921,251 | 76.3 | 77.2 |
| SLL188 | 132,272,286 | 75.8 | 99.43 | 25,519,119 | 9.16 | 17,054,395 | 75.6 | 75.6 |
| SLL189 | 132,138,954 | 74.4 | 99.43 | 25,449,602 | 8.63 | 17,951,427 | 75.0 | 75.5 |

**Table S3. WGBS summary statistics for ten nesting loggerheads.** Alignment, de-duplication, and methylation calling was performed with Bismark v.0.22.1 (Krueger and Andrews, 2011).

| **Assembly** | **Complete**  **BUSCOs (%)** | **Single copy**  **BUSCOs (%)** | **Duplicated**  **BUSCOs (%)** | **Fragmented**  **BUSCOs (%)** | **Missing**  **BUSCOs (%)** |
| --- | --- | --- | --- | --- | --- |
| **CarCar_QM_v1.21.12_Sc** (Loggerhead sea turtle, scaffolded) | 97.1 | 96.2 | 0.9 | 0.4 | 2.5 |
| **CarCar_GSC_CCare_1.0**  (Loggerhead sea turtle, scaffolded) | 96.1 | 95.2 | 0.9 | 0.4 | 3.5 |
| **rDerCor1.pri.v4**  (Leatherback sea turtle, scaffolded) | 96.3 | 95.3 | 1.0 | 0.6 | 3.1 |
| **rCheMyd1.pri.v2**  (Green sea turtle, scaffolded) | 97.2 | 96.2 | 1.0 | 0.4 | 2.4 |
| **CheMyd_1.0**  (Green sea turtle, contig-level) | 95.9 | 94.8 | 1.1 | 1.3 | 2.8 |
| **ASM3001250v1**  (Hawksbill sea turtle, scaffolded) | 97.1 | 96.0 | 1.1 | 0.4 | 2.5 |

**Table S4. Full BUSCO summary for genome assemblies across sea turtle species.** BUSCO scores were calculated against the Sauropsida gene set (n=7480) with BUSCO v.5.1.2 in genome mode (Simão *et al.*, 2015).

| **Element type** | **Total count** | **Total length (bp)** | **% of sequence** |
| --- | --- | --- | --- |
| **Retroelements** | 2,157,560 | 579,942,877 | 27.0 |
| SINEs | 162,817 | 16,136,269 | 0.75 |
| LINEs | 764,233 | 280,847,563 | 13.1 |
| LTRs | 1,230,510 | 282,959,045 | 13.2 |
| **DNA transposons** | 1,346,354 | 209,182,328 | 9.75 |
| **Unclassified** | 835,962 | 119,299,710 | 5.56 |

**Table S5. Repetitive element summary statistics.** Statistics generated by RepeatMasker v.4.1.4 (Smit, Hubley, and Green 2022).

| **Annotation statistics** |  |
| --- | --- |
| Total repeat sequence masked (Mbp) | 919.0 (42.8% of genome) |
| Total genes | 33,887 |
| Total genes with functional annotations | 27,817 (82.1% of genes) |
| Total genes with GO annotations | 19,966 (58.9% of genes) |
| Total exons | 363,356 |
| Total single exon genes | 3686 |
| Total introns in coding sequence | 328,525 |
| Mean gene length (bp) | 50,969 |
| Longest gene length (bp) | 1,079,287 |
| Mean exon length (bp) | 164 |
| Longest exon length (bp) | 13,855 |
| Mean intron length in coding sequence (bp) | 5035 |
| Longest intron length in coding sequence (bp) | 414,767 |

**Table S6. Gene annotation summary statistics.** Annotation statistics were computed with AGAT v.0.9.1 (Dainat, 2022).

| **Annotation** | **Complete**  **BUSCOs (%)** | **Single copy**  **BUSCOs (%)** | **Duplicated**  **BUSCOs (%)** | **Fragmented**  **BUSCOs (%)** | **Missing**  **BUSCOs (%)** |
| --- | --- | --- | --- | --- | --- |
| **CarCar_QM_v1.21.12_Sc** (Loggerhead sea turtle, scaffolded) | 95.4 | 94.4 | 1.0 | 1.1 | 3.5 |
| **CarCar_GSC_CCare_1.0**  (Loggerhead sea turtle, scaffolded) | 97.8 | 96.8 | 1.0 | 0.2 | 2.0 |
| **rDerCor1.pri.v4**  (Leatherback sea turtle, scaffolded) | 98.2 | 97.1 | 1.1 | 0.5 | 1.3 |
| **rCheMyd1.pri.v2**  (Green sea turtle, scaffolded) | 98.9 | 97.8 | 1.1 | 0.3 | 0.8 |
| **CheMyd_1.0**  (Green sea turtle, contig-level) | 95.8 | 94.9 | 0.9 | 1.5 | 2.7 |
| **ASM3001250v1**  (Hawksbill sea turtle, scaffolded) | 92.3 | 91.2 | 1.1 | 2.3 | 5.4 |

**Table S7. Full BUSCO summary for genome annotations across sea turtle species.** BUSCO scores were calculated on the longest gene isoforms against the Sauropsida gene set (n=7480) with BUSCO v.5.1.2 in protein mode (Simão *et al.*, 2015).

| **Sample ID** | **df** | **F-value** | **p-value** |
| --- | --- | --- | --- |
| **Methylation per gene-associated CpG** | | | |
| SLL063 | 7,742,051 | 79,568 | p<0.0001 |
| SLL065 | 7,996,047 | 77,578 | p<0.0001 |
| SLL142 | 7,917,843 | 79,869 | p<0.0001 |
| SLL143 | 7,917,350 | 85,633 | p<0.0001 |
| SLL144 | 8,079,404 | 75,596 | p<0.0001 |
| SLL146 | 7,856,588 | 79,536 | p<0.0001 |
| SLL171 | 8,088,838 | 85,898 | p<0.0001 |
| SLL176 | 7,934,149 | 71,308 | p<0.0001 |
| SLL188 | 7,917,946 | 74,108 | p<0.0001 |
| SLL189 | 8,148,392 | 80,926 | p<0.0001 |
| **Mean methylation per gene** | | | |
| SLL063 | 83,408 | 499.1 | p<0.0001 |
| SLL065 | 83,774 | 402.0 | p<0.0001 |
| SLL142 | 83,654 | 417.9 | p<0.0001 |
| SLL143 | 83,841 | 526.9 | p<0.0001 |
| SLL144 | 83,855 | 369.4 | p<0.0001 |
| SLL146 | 83,659 | 500.3 | p<0.0001 |
| SLL171 | 84,058 | 480.4 | p<0.0001 |
| SLL176 | 83,866 | 368.9 | p<0.0001 |
| SLL188 | 83,832 | 247.7 | p<0.0001 |
| SLL189 | 83,960 | 345.0 | p<0.0001 |

**Table S8. Linear model results for the interaction term per individual WGBS methylome.** ANOVA results for the interaction term of the linear model: lm(ONT methylation value ~ WGBS methylation value * Feature type), performed separately for each of the ten WGBS methylomes. This analysis compared methylation values at gene-associated CpGs (top) and mean methylation per gene (bottom).

| **Sample**  **ID** | **Exons** | | | **Introns** | | | **Promoters** | | | **Intergenic <10kb from TSS)** | | |
| --- | --- | --- | --- | --- | --- | --- | --- | --- | --- | --- | --- | --- |
|  | **df** | **R^2^** | **p-value** | **df** | **R^2^** | **p-value** | **df** | **R^2^** | **p-value** | **df** | **R^2^** | **p-value** |
| **Methylation per gene-associated CpG** | | | | | | | | | | | | |
| SLL063 | 396,916 | 0.72 | p<0.0001 | 5,782,546 | 0.65 | p<0.0001 | 403,097 | 0.92 | p<0.0001 | 1,159,492 | 0.76 | p<0.0001 |
| SLL065 | 414,915 | 0.73 | p<0.0001 | 5,963,218 | 0.65 | p<0.0001 | 421,894 | 0.92 | p<0.0001 | 1,196,020 | 0.77 | p<0.0001 |
| SLL142 | 409,912 | 0.72 | p<0.0001 | 5,906,192 | 0.65 | p<0.0001 | 413,284 | 0.92 | p<0.0001 | 1,188,455 | 0.76 | p<0.0001 |
| SLL143 | 411,118 | 0.71 | p<0.0001 | 5,882,721 | 0.64 | p<0.0001 | 411,505 | 0.91 | p<0.0001 | 1,212,006 | 0.75 | p<0.0001 |
| SLL144 | 414,337 | 0.71 | p<0.0001 | 6,021,102 | 0.64 | p<0.0001 | 411,006 | 0.91 | p<0.0001 | 1,232,959 | 0.75 | p<0.0001 |
| SLL146 | 405,424 | 0.72 | p<0.0001 | 5,869,793 | 0.65 | p<0.0001 | 401,315 | 0.92 | p<0.0001 | 1,180,056 | 0.76 | p<0.0001 |
| SLL171 | 422,997 | 0.72 | p<0.0001 | 5,985,665 | 0.64 | p<0.0001 | 435,157 | 0.92 | p<0.0001 | 1,245,019 | 0.76 | p<0.0001 |
| SLL176 | 416,002 | 0.71 | p<0.0001 | 5,912,198 | 0.64 | p<0.0001 | 399,082 | 0.91 | p<0.0001 | 1,206,867 | 0.76 | p<0.0001 |
| SLL188 | 417,070 | 0.72 | p<0.0001 | 5,875,665 | 0.64 | p<0.0001 | 417,109 | 0.92 | p<0.0001 | 1,208,102 | 0.76 | p<0.0001 |
| SLL189 | 425,190 | 0.72 | p<0.0001 | 6,049,465 | 0.64 | p<0.0001 | 430,153 | 0.92 | p<0.0001 | 1,243,584 | 0.75 | p<0.0001 |
| **Mean methylation per gene** | | | | | | | | | | | | |
| SLL063 | 19770 | 0.87 | p<0.0001 | 18,853 | 0.93 | p<0.0001 | 23,380 | 0.95 | p<0.0001 | 21,405 | 0.91 | p<0.0001 |
| SLL065 | 19972 | 0.88 | p<0.0001 | 18,882 | 0.94 | p<0.0001 | 23,490 | 0.96 | p<0.0001 | 21,430 | 0.93 | p<0.0001 |
| SLL142 | 19897 | 0.88 | p<0.0001 | 18,882 | 0.94 | p<0.0001 | 23,441 | 0.96 | p<0.0001 | 21,434 | 0.92 | p<0.0001 |
| SLL143 | 19940 | 0.87 | p<0.0001 | 18,905 | 0.93 | p<0.0001 | 23,522 | 0.95 | p<0.0001 | 21,474 | 0.92 | p<0.0001 |
| SLL144 | 19972 | 0.87 | p<0.0001 | 18,901 | 0.94 | p<0.0001 | 23,510 | 0.95 | p<0.0001 | 21,472 | 0.92 | p<0.0001 |
| SLL146 | 19887 | 0.88 | p<0.0001 | 18,886 | 0.93 | p<0.0001 | 23,437 | 0.95 | p<0.0001 | 21,449 | 0.91 | p<0.0001 |
| SLL171 | 20053 | 0.88 | p<0.0001 | 18,924 | 0.94 | p<0.0001 | 23,598 | 0.96 | p<0.0001 | 21,483 | 0.92 | p<0.0001 |
| SLL176 | 19978 | 0.87 | p<0.0001 | 18,912 | 0.94 | p<0.0001 | 23,507 | 0.95 | p<0.0001 | 21,469 | 0.92 | p<0.0001 |
| SLL188 | 19962 | 0.88 | p<0.0001 | 18,907 | 0.94 | p<0.0001 | 23,510 | 0.96 | p<0.0001 | 21,453 | 0.92 | p<0.0001 |
| SLL189 | 20012 | 0.88 | p<0.0001 | 18,917 | 0.94 | p<0.0001 | 23,563 | 0.96 | p<0.0001 | 21,468 | 0.92 | p<0.0001 |

**Table S9. Pearson’s correlation test results per individual WGBS methylome.** Results from a Pearson’s pairwise correlation test comparing methylation values between the reference ONT methylome and each of the ten WGBS methylomes separately. Correlations were performed separately for each feature type (exons, introns, promoters, gene-associated intergenic), given an interaction by feature type was found to significant in **Table S8**. This analysis compared methylation values at gene-associated CpGs (top) and mean methylation per gene (bottom).

| **Alignment**  **identity** | **Leatherback sea turtle** | **Green sea turtle** | **Hawksbill sea turtle** |
| --- | --- | --- | --- |
| No match | 7.57 | 2.81 | 1.34 |
| <25% | 1.47 | 0.10 | 0.03 |
| 25-50% | 87.58 | 7.01 | 1.25 |
| 50-75% | 3.37 | 90.07 | 81.03 |
| >75% | 0 | 0.01 | 16.35 |

**Table S10. Summary of whole genome alignments between the loggerhead sea turtle and other sea turtle species.** Alignments and statistics produced by D-GENIES v.1.5.0 (Cabanettes and Klopp, 2018)

| **Mean promoter methylation status** | **Proportion (%) per cluster** | | | | |
| --- | --- | --- | --- | --- | --- |
|  | **Dark blue** | **Dark purple** | **Mauve** | **Pink** | **Yellow** |
| High | 33.33 | 41.67 | 20 | 42.86 | 50 |
| Intermediate | 6.67 | 8.33 | 20 | 14.29 | 0 |
| Low | 60.00 | 50.00 | 60 | 42.86 | 50 |

**Table S11. Proportions of promoter methylation categories in the top five functional clusters.** Methylation status of TSD-linked gene promoters in the reference individual methylome (i.e. blood of a nesting female) were assigned into high (>70%), intermediate (30-70%) or low (<30%) categories based on the bimodal distribution observed (**Figure 5D**). The proportion of gene promoters falling into these three categories are provided for the five largest functional clusters identified via Markov Clustering from our STRING functional association network created for TSD-linked genes (**Figure 5E**).

**Text S1. Extended methods and results for mitochondrial genome assembly and annotation.** We assembled and annotated the mitochondrial genome from our Illumina data. To extract a read set enriched for mitochondrial sequence, reads were mapped via BWA-MEM v.0.7.17 (Li, 2013) against a published loggerhead mitochondrial assembly (Drosopoulou et al., 2012). Mapped reads were inputted to MitoZ v.3.4 (Meng *et al.,* 2019) with the Megahit assembler. Our assembly consisted of a circular 16,574 bp contig with 37 genes (13 protein coding genes, 22 tRNA genes and 2 rRNA genes, **Figure S4**). This assembly was 98.9% identical to a loggerhead mitochondrial genome from a Greek population (Drosopoulou *et al.,* 2012). Divergence likely reflects genetic structure between the North Atlantic and Mediterranean nesting groups, driven by female philopatry in sea turtles (Baltazar-Soares *et al*., 2020; Tolve *et al.,* 2023). To assign the reference individual to a mitochondrial haplogroup, control region haplotype sequences found in the Cabo Verde nesting group (Baltazar-Soares et al., 2020) were downloaded from NCBI GenBank (Sayers *et al.,* 2021) and aligned against our assembly via BLASTn v.2.7.1+ (Altschul *et al.,* 1990). The control region was 99.9% similar to the CC-A1.4 haplotype (GenBank ID: EU179439.1). The reference individual is therefore a member of Haplogroup I (CC-A1), the oldest and commonest loggerhead lineage in Cabo Verde (Baltazar-Soares *et al.,* 2020).

**Text S2. Extended methods for methylation calling from WGBS data of ten loggerheads.**

Raw WGBS reads were trimmed for remaining adapters and filtered for a mean Phred score >Q20 using cutadapt v.2.10 (Martin, 2011). Using Bismark v.0.22.1 with default options (Krueger and Andrews, 2011), trimmed reads were aligned against our reference assembly, giving a mean mapping efficiency of 79.1 ± 3.37 (SD) % per sample (**Table S3**). Alignments were deduplicated with Bismark in paired end mode, followed by merging and sorting with samtools v.1.9 (Li *et al.*, 2009). Methylation calling was then performed in Bismark. Percentage methylation totalled across CHG and CHH sites were calculated to obtain an estimate of bisulfite conversion efficiency (Laine *et al.*, 2023). This gave a mean of 99.39 ± 0.047 (SD) %, indicating high conversion efficiency (**Table S3**). To improve coverage and minimise pseudo-replication, we further de-stranded adjacent cytosines per CpG site using the ‘merge_CpG.py’ script (Cristofari, 2023), as methylation occurs symmetrically at CpG sites in vertebrates (Klughammer *et al.*, 2023). On average, this resulted in 25,485,557 ± 35,209 (SD) CpG sites per sample, with a de-stranded coverage of 9.2 ± 0.33 (SD) X (**Table S3**). Methylation calls at CpG sites were further processed in RStudio v.4.2.2 (R Core Team, 2021) with the methylKit package v.1.24.0 (Akalin *et al.*, 2012). CpG sites were excluded if they had a coverage lower than 8X to match the filtering threshold applied to the ONT methylome, or if they were within the 99.9th percentile to account for PCR bias (Wreczycka *et al.*, 2017). Coverage was then normalised between samples using methylKit’s ‘normalizeCoverage’ function. As a final filtering step, we retained CpG sites that were covered in at least 75% of individuals, leaving 24,299,151 CpG sites for downstream analyses. For each CpG site, percentage methylation was calculated per individual with methylKit’s ‘percMethylation’ function, then the mean across all individuals was calculated per CpG site to provide an average WGBS methylome representative of the population.

**Text S3. Extended methods for annotating gene feature types.**

To annotate the feature type upon which CpG sites reside, we used the R packages genomation v.1.30.0 (Akalin *et al.*, 2015) and GenomicRanges v.1.50.2 (Lawrence *et al.*, 2013) alongside our reference genome. Promoter regions were defined as 1500 bp upstream and up to 500 bp downstream of a transcriptional start site (TSS; Heckwolf *et al.*, 2020). All CpG sites were assigned to one of four feature types using genomation’s ‘annotateWithGeneParts’ function, in the following order of precedence when features overlapped: promoter, exon, intron or intergenic region. To attach functional gene information to CpG sites, those in genic regions (i.e., located on a promoter, intron or exon) were associated to a gene using the ‘findOverlaps’ function of GenomicRanges. DMS in intergenic regions were associated to a gene using genomation’s ‘getAssociationWithTSS’ function, if they were less than 10 kb away from the nearest TSS (Heckwolf *et al.*, 2020).

**Text S4. Extended methods for identification and curation of TSD-linked genes.**

Bentley *et al.,* (2023) compiled the most comprehensive list to date of 223 genes with documented links to temperature-dependent sex determination (TSD) pathways. Firstly, we manually curated this list by removing 11 genes that were not found in either the green or leatherback genomes, consolidating GLU and NAGLU which referred to the same gene, and correcting the gene name ‘ST6GALC2’ to ‘ST6GAL2’. We further replaced four sequences that did not map to the corresponding gene: the A2M sequence was replaced to XM_037912788.2 for the green turtle, the PDGFA sequence was replaced to XR_003565502.3 in the green turtle and XR_005295469.2 in the leatherback turtle, the PDGFB sequence was replaced to XM_037881931.2 in the green turtle and XM_038387707.2 in the leatherback turtle, and ST6GAL2 was replaced to XM_007054611.4 in the green turtle and XM_043509904.1 in the leatherback turtle.

Using our curated list of TSD-linked genes, orthologues were identified in our loggerhead assembly via BLASTn v.2.11.0 with parameters ‘-evalue 1e-30’ and ‘-perc_identity 70’ (Altschul *et al.*, 1990) against gene sequences from the VGP green turtle annotation, which is more closely related to the loggerhead turtle than the leatherback turtle. To verify that true orthologues were selected, a manual curation step integrating BLAST homology and gene name information was conducted. This involved looking up the gene name for every BLAST hit in our loggerhead functional annotation, and retaining those that matched the query gene name. We took the top BLAST hit if there was no match to query gene name, due to genes having multiple aliases or missing annotation information. For loggerhead genes that matched sequences in two chromosomal locations, hits were retained if they were both syntenic or neither syntenic with the other species. If one sequence was syntenic and the other was not, the non-syntenic sequence was removed under the conservative assumption of it being an assembly or orthologue identification error. This left 201 unique TSD-linked genes in our loggerhead genome for downstream analyses (five genes annotated in two locations; 206 total loci).

**
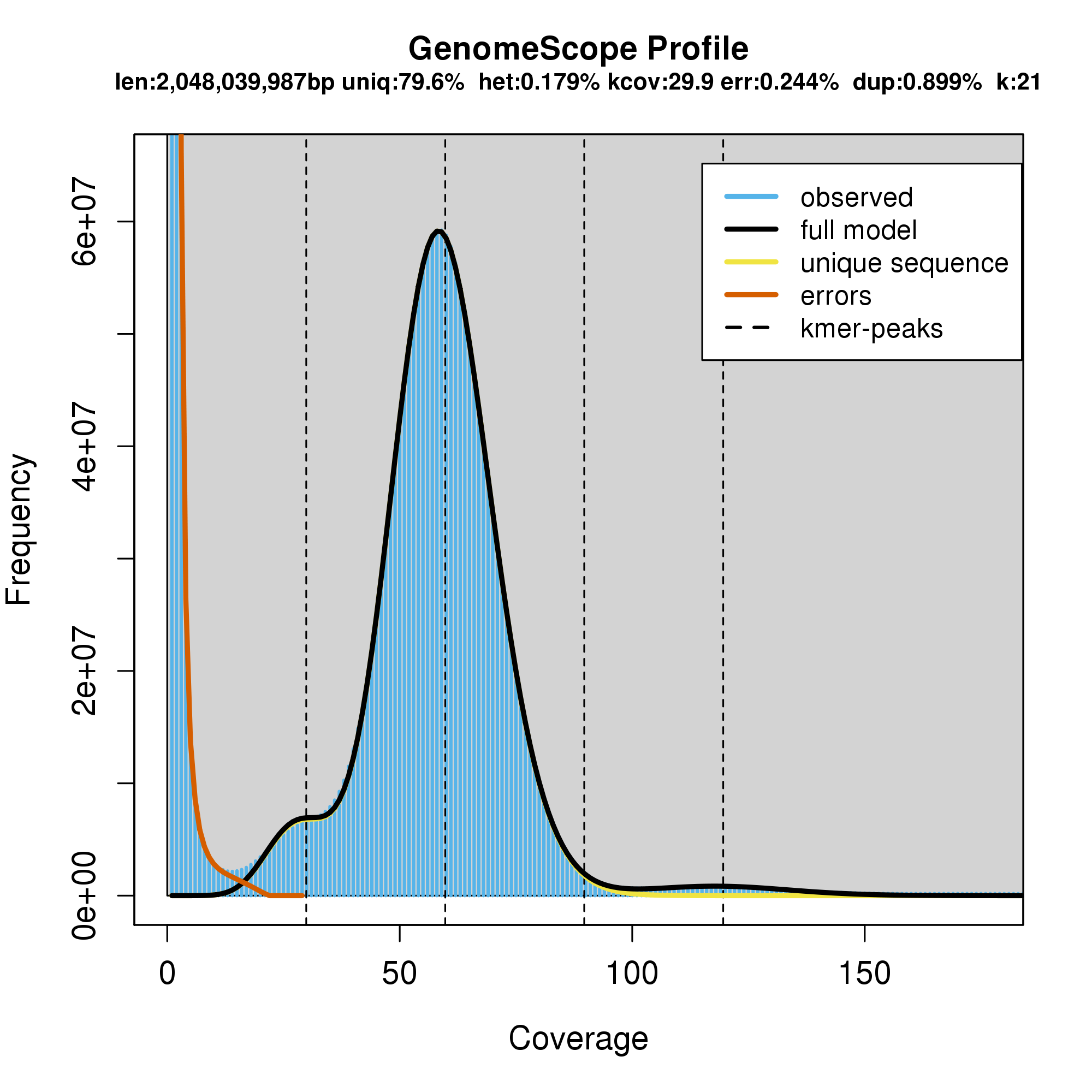
**

**Figure S1. GenomeScope profile for our loggerhead genome.** Produced using GenomeScope (Vurture *et al.*, 2017) on k-mers derived from our raw, Illumina reads, with parameter k=21. Estimated haploid genome size is 2.05 Gbp, heterozygosity is 0.179% and repeat fraction is 20.4%. These metrics were used to help choose downstream parameters.


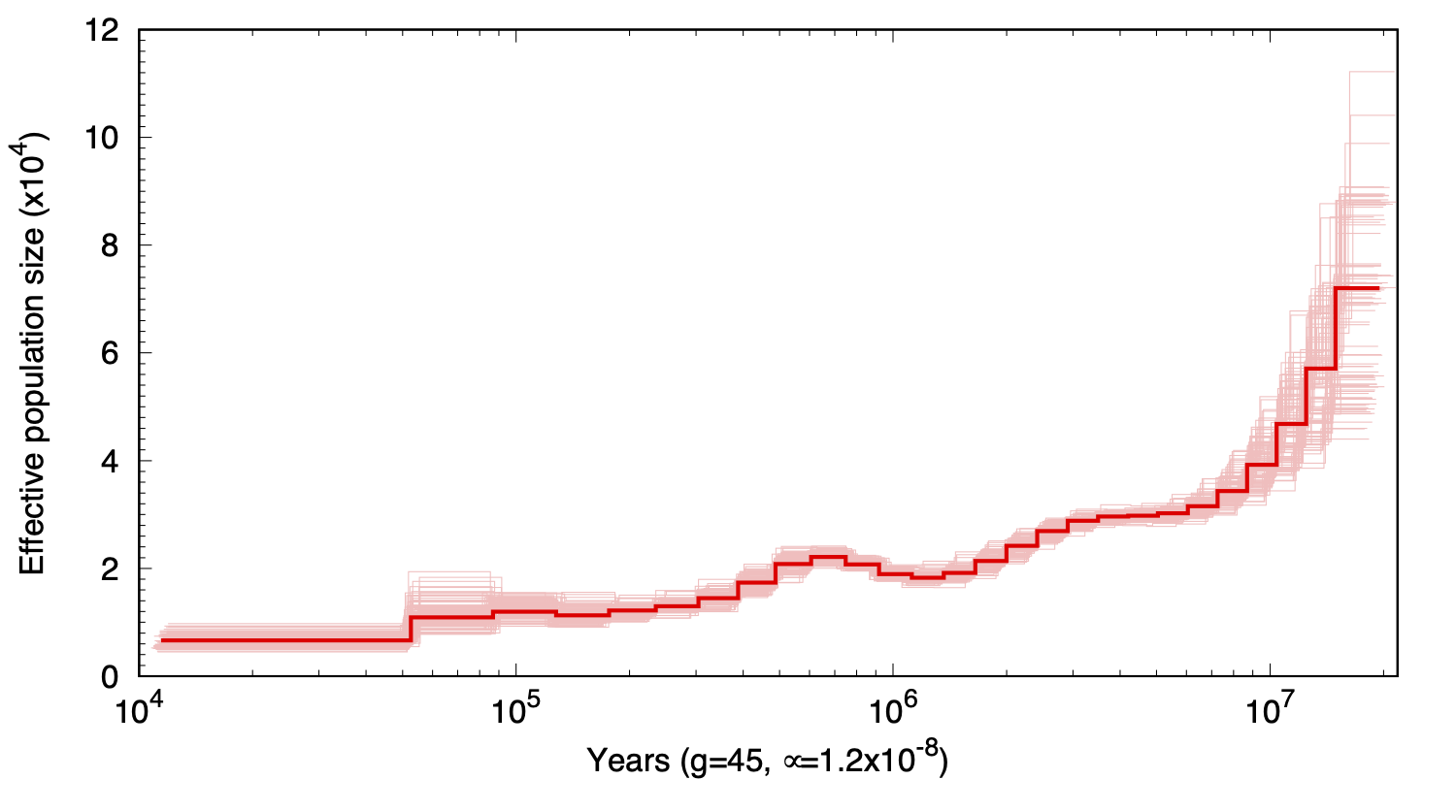

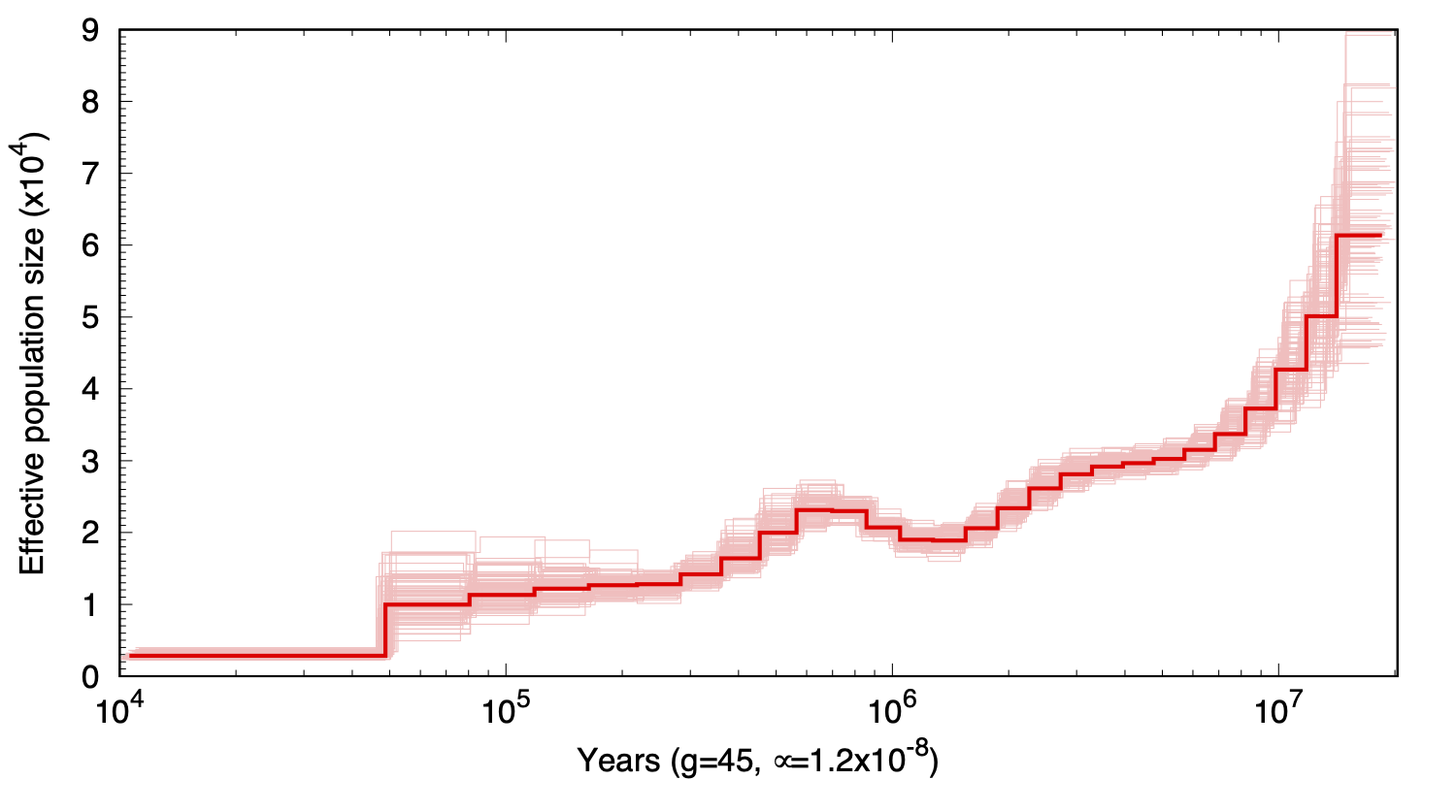


**A**

**B**

**C**

**D**

**Figure S2. Auxiliary PSMC tests with 100 bootstraps**. PSMC (Li and Durbin, 2011) run from the 11 macrochromosomes of the loggerhead genome (80.8% of assembly) for **(A)** SLK063 (Cabo Verde, East Atlantic), and **(B)** SAMN20502673 (Brazil, West Atlantic). PSMC was also run on the 17 microchromosomes (19.2% of assembly) for **(C)** SLK063 and **(D)** SAMN20502673, confirming overall patterns were comparable with macrochromosomes.


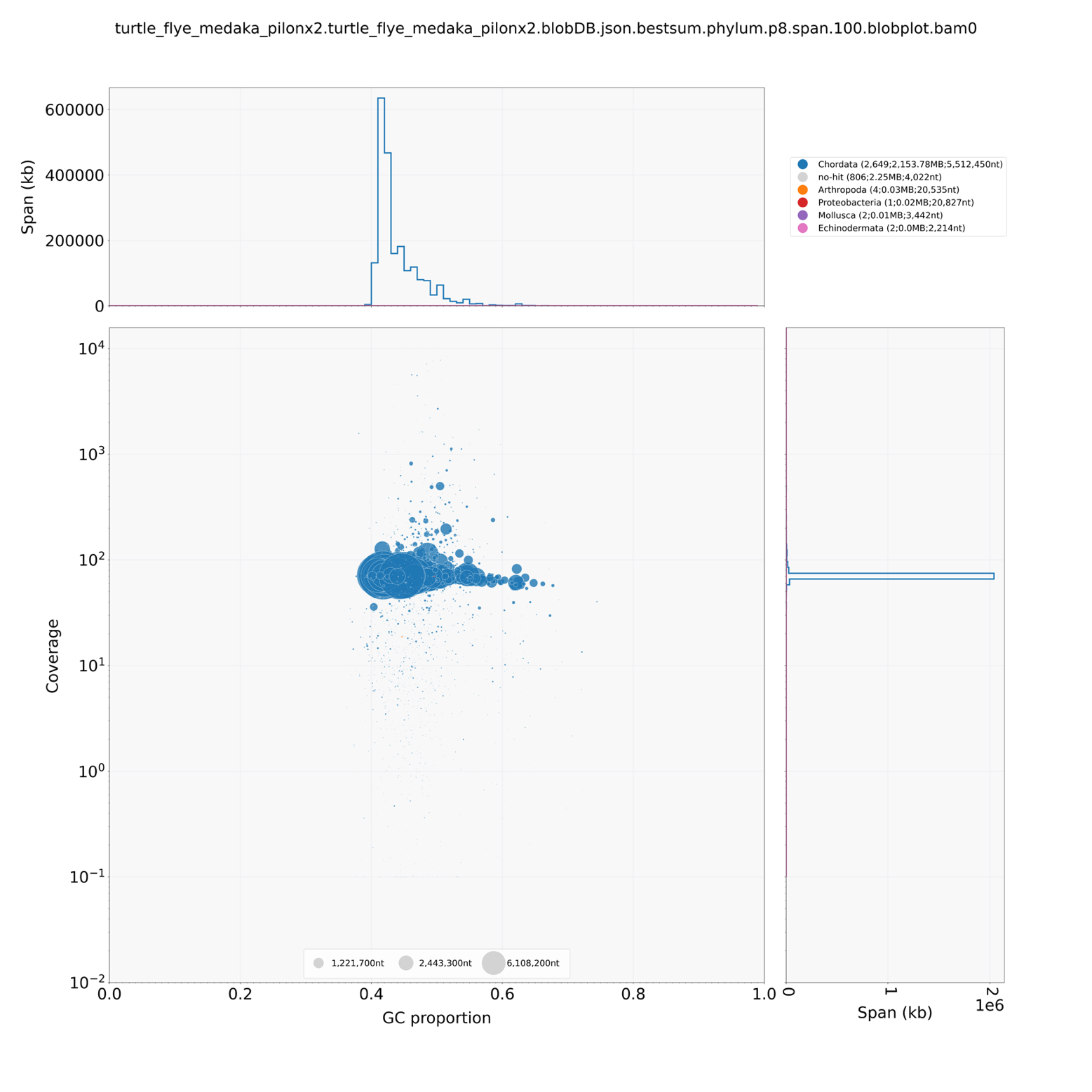

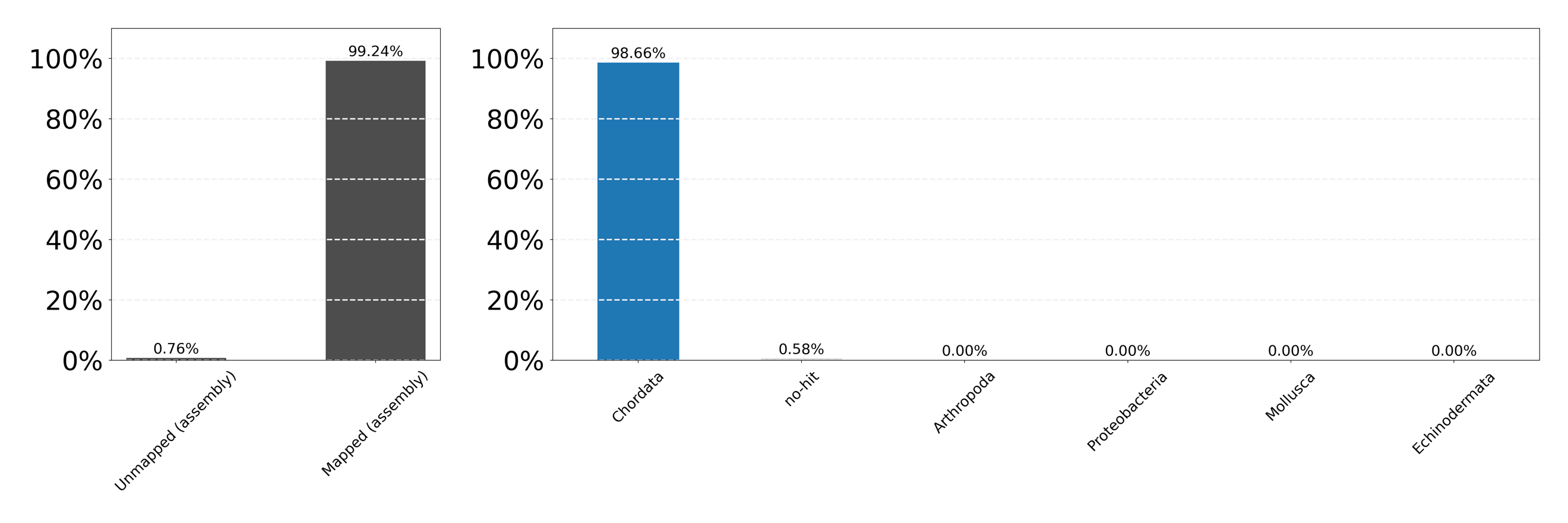


**Figure S3. GC-coverage blob-plot to evaluate assembly contamination.** Outputted by BlobTools v.1.1.1 (Laetsch and Blaxter, 2017) with our contig-level assembly. Scaffolds are coloured by phylum, and blob size represents scaffold length. Frequency histograms are plotted along the side of each axis. Lower bar charts show the proportion of scaffolds that were mapped to each phylum. Of the 99.24% of scaffolds that successfully mapped to our assembly, 98.66% of scaffolds mapped to Chordata (blue), and 0.58% produced no hit. This confirms minimal taxonomic contamination from other phyla in our assembly.


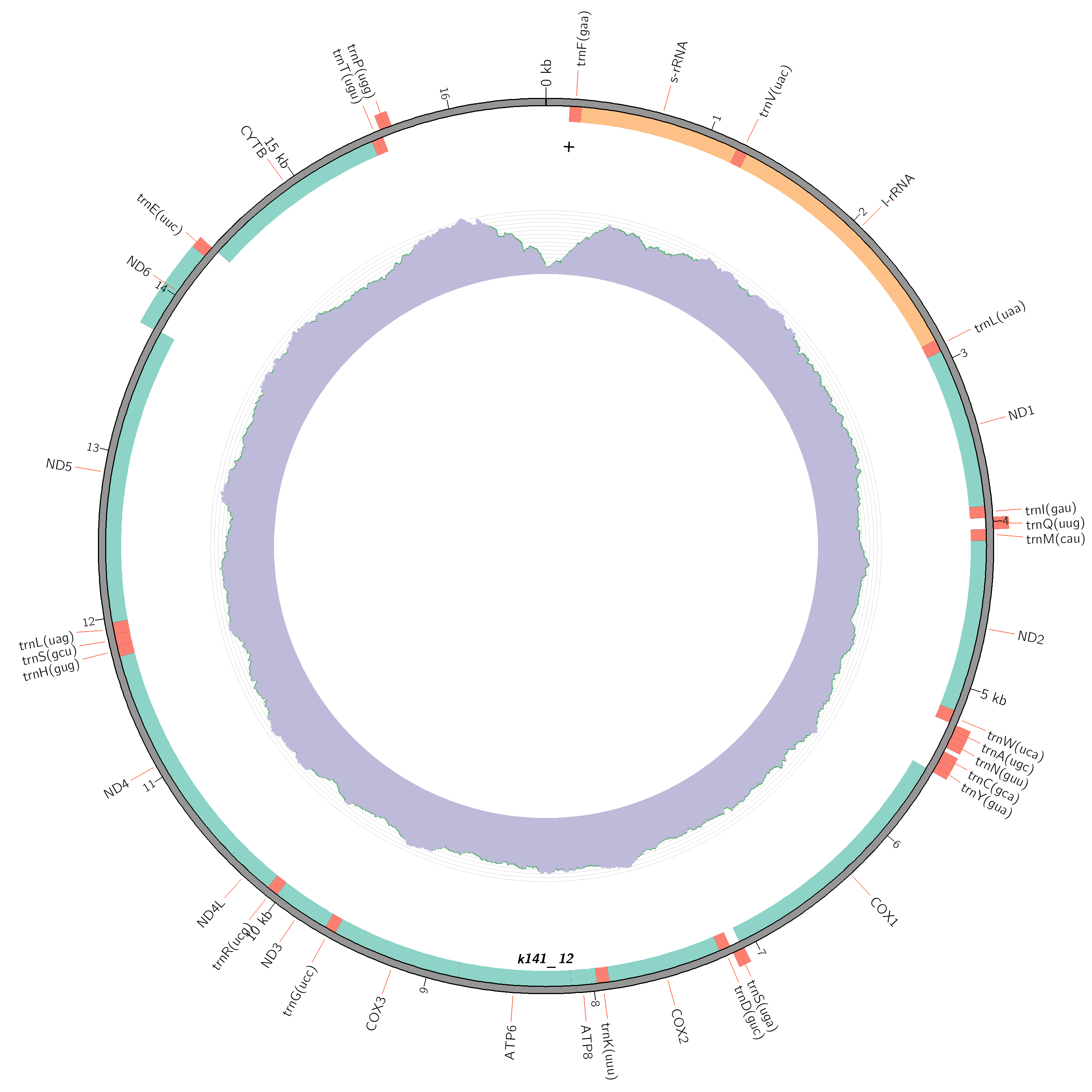


**Figure S4. Mitochondrial genome assembly and annotation.** Length: 16,574 bp, GC content: 38.75%, total number of genes: 37 (protein coding genes: 13, tRNA genes: 22, rRNA genes: 2). The inner purple ring visualises coverage. Mitochondrial assembly, annotation and visualisation were performed with MitoZ v.3.4 (Meng *et al.*, 2019).

**
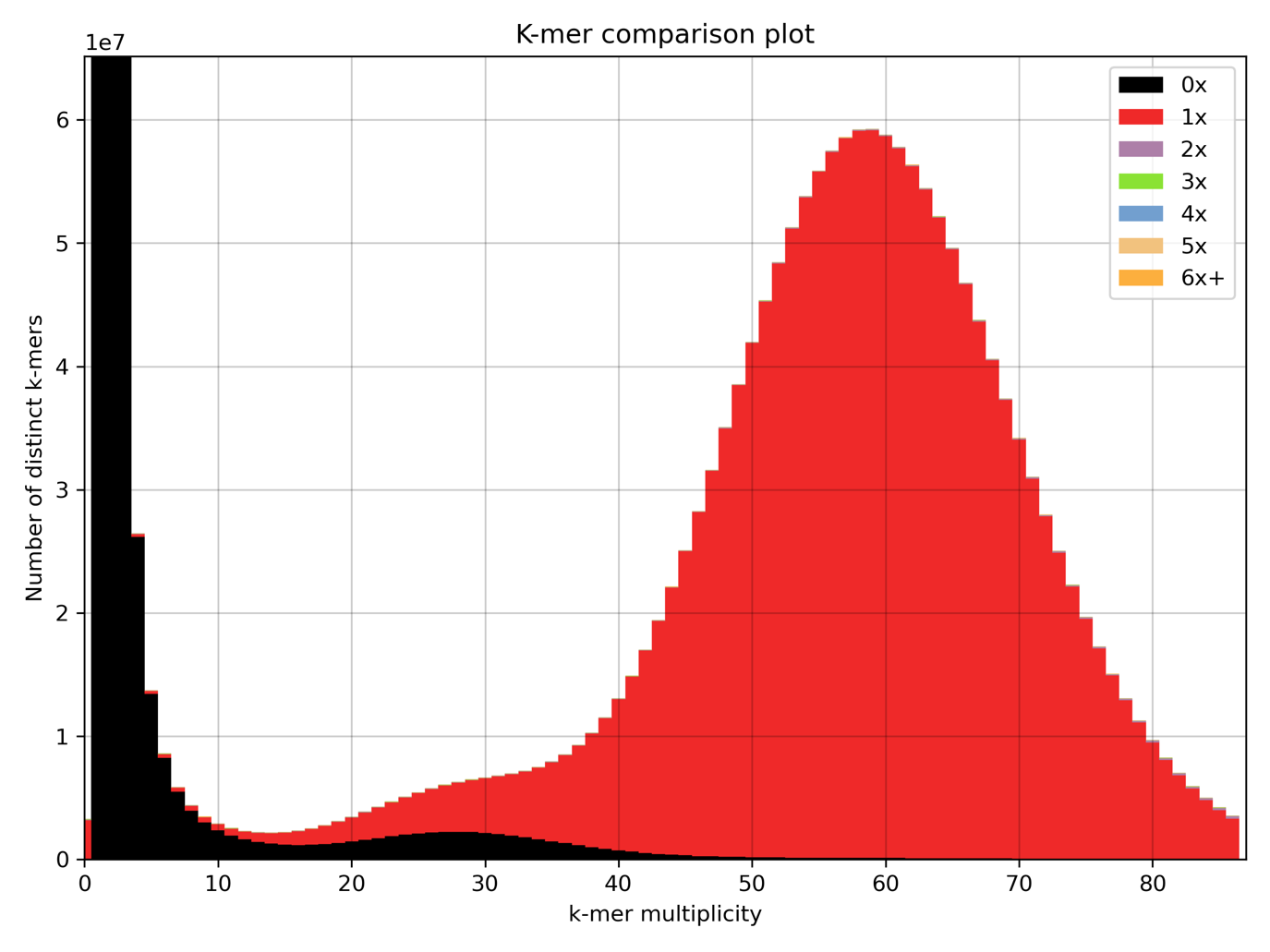
**

**Figure S5. K-mer spectrum plot of our loggerhead assembly.** Produced using KAT v.2.4.1 (Mapleson *et al.*, 2017), showing the frequency of k-mers in the assembly versus frequency of k-mers (i.e. sequencing coverage) in the raw Illumina reads. The first, black peak corresponds to k-mers present in the raw reads but missing from the assembly due to sequencing errors. The second, smaller peak corresponds to k-mers from heterozygous regions, and third peak corresponds to k-mers from homozygous regions. This plot confirms that our assembly is well haploidised.


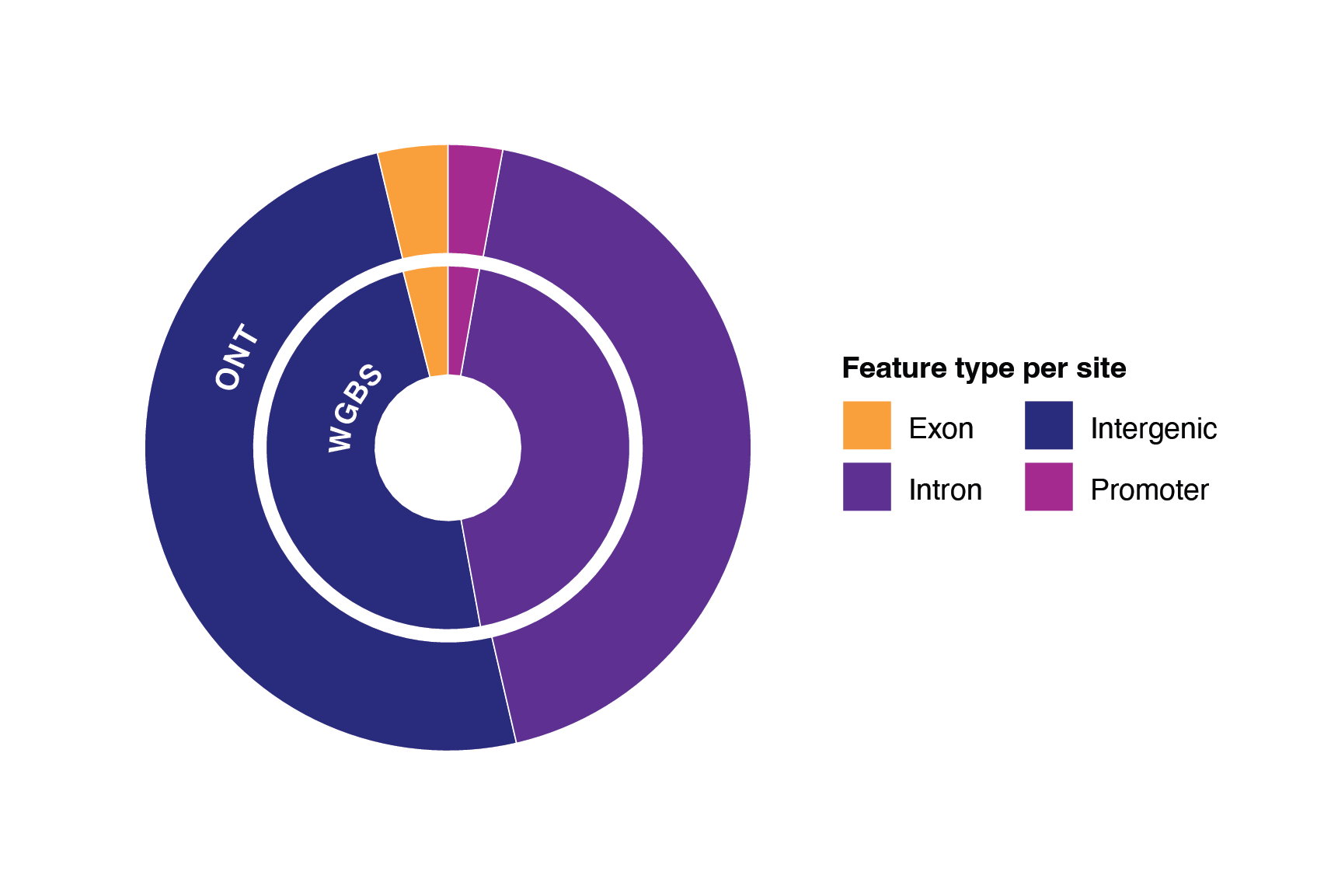


**Figure S6. Genome-wide distribution of highly methylated (>70%) CpGs across feature types by site**. The outer ring shows the distribution called from the ONT methylome of the reference individual (n=19,606,231 CpGs), with 3.74% on exons (yellow), 24.64% on introns (dark purple), 2.90% on promoters (mauve) and 49.89% being intergenic (dark blue). The inner ring shows the distribution called from the average of ten WGBS methylomes (n=17,950,597 CpGs), with 3.96% on exons, 24.96% on introns, 2.78% on promoters and 49.91% being intergenic. Highly methylated CpGs are similarly distributed across feature types between sequencing methods over the entire genome (χ2=0.0387, p=0.998).


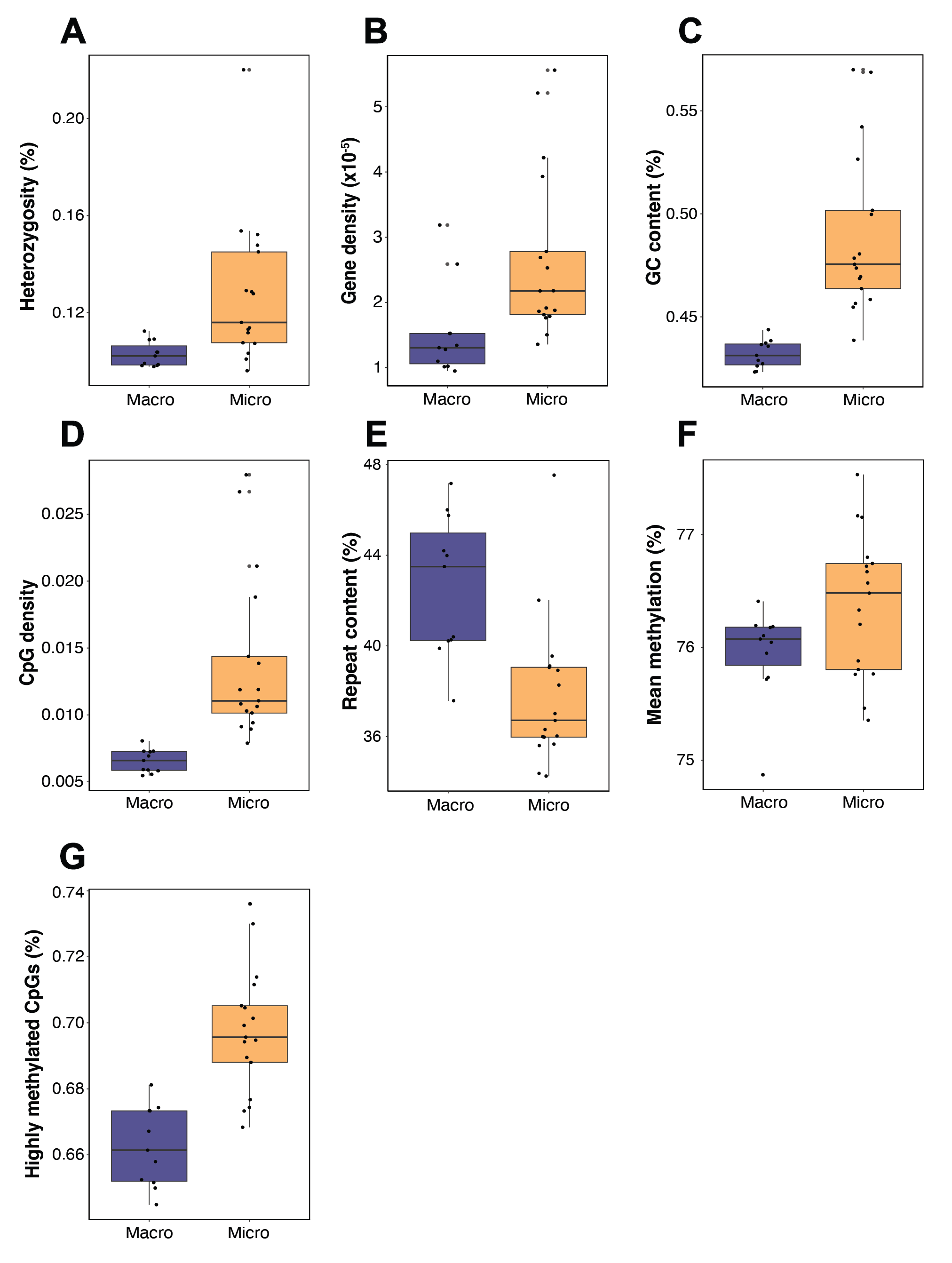


**Figure S7. Comparison of macro- and microchromosomes properties.** Boxplots comparing genetic and epigenetic properties per chromosome, for the 11 macrochromosomes (dark blue) and 17 microchromosomes (yellow) of the loggerhead genome. Comparisons for: **(A)** heterozygosity (%), **(B)** gene density (total genes over chromosome length, x10^-5^), **(C)** GC content (%), **(D)** CpG density (total CpGs over chromosome length), **(E)** methylation (%), **(F)** proportion of highly methylated (>70%) CpGs (%), and (**G**) mean repeat content (%). Microchromosomes are more heterozygous (W=29, p=0.002), gene-dense (W=28, p=0.001), GC-rich, CpG-dense (W=1, p<0.0001), possess a larger proportion of highly methylated CpGs (W=54, p<0.0001), and are less repeat-rich (W=166, p=0.0003) with the notable exception of chromosome 28, which has the highest repeat content overall.

**Figure S8. Violin plot of mean methylation per gene.** For TSD-linked genes (n=199; purple) versus non-TSD-linked genes (n=11,560 single-copy orthologues between sea turtle species; yellow).


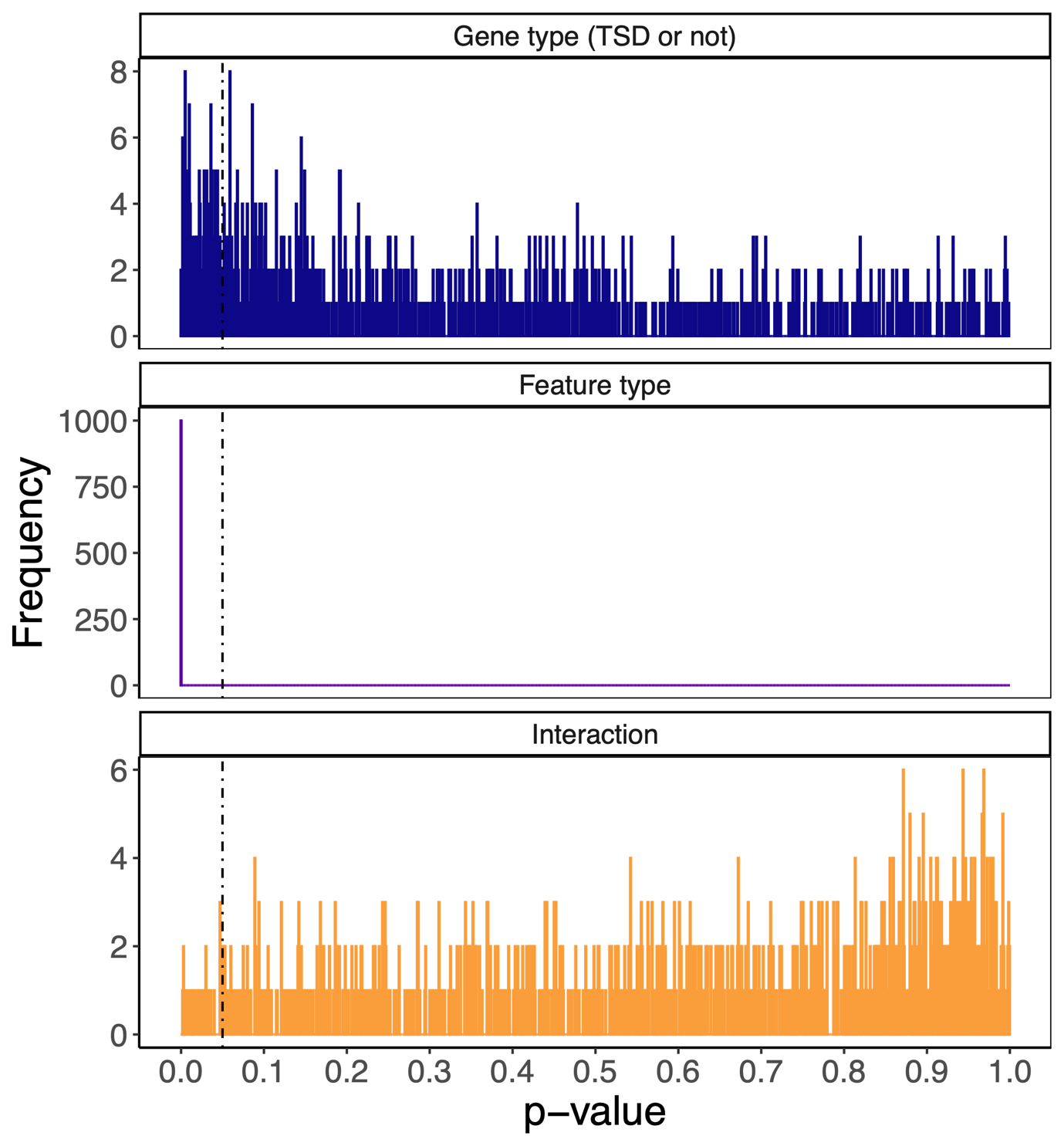


**Figure S9. P-value histogram from comparing mean methylation between TSD-linked genes and non-TSD-linked genes.** A linear model was used to test if mean methylation differed between 199 TSD-linked genes and 1000 random subsets of 199 non-TSD-linked genes, sampled from 11,560 single-copy orthologues shared between sea turtle species. Histograms are plotted for the p-values per iteration (n=1000), for each term in the ANOVA results table: gene category (TSD-linked versus non-TSD-linked, dark blue), genomic feature type (purple) and their interaction (Gene category x Feature type, yellow). The dashed line represents p=0.05. 161 (16.1%) tests passed p<0.05 for gene category, 1000 (100%) tests passed for feature type and 28 (0.28%) tests passed for the interaction term.


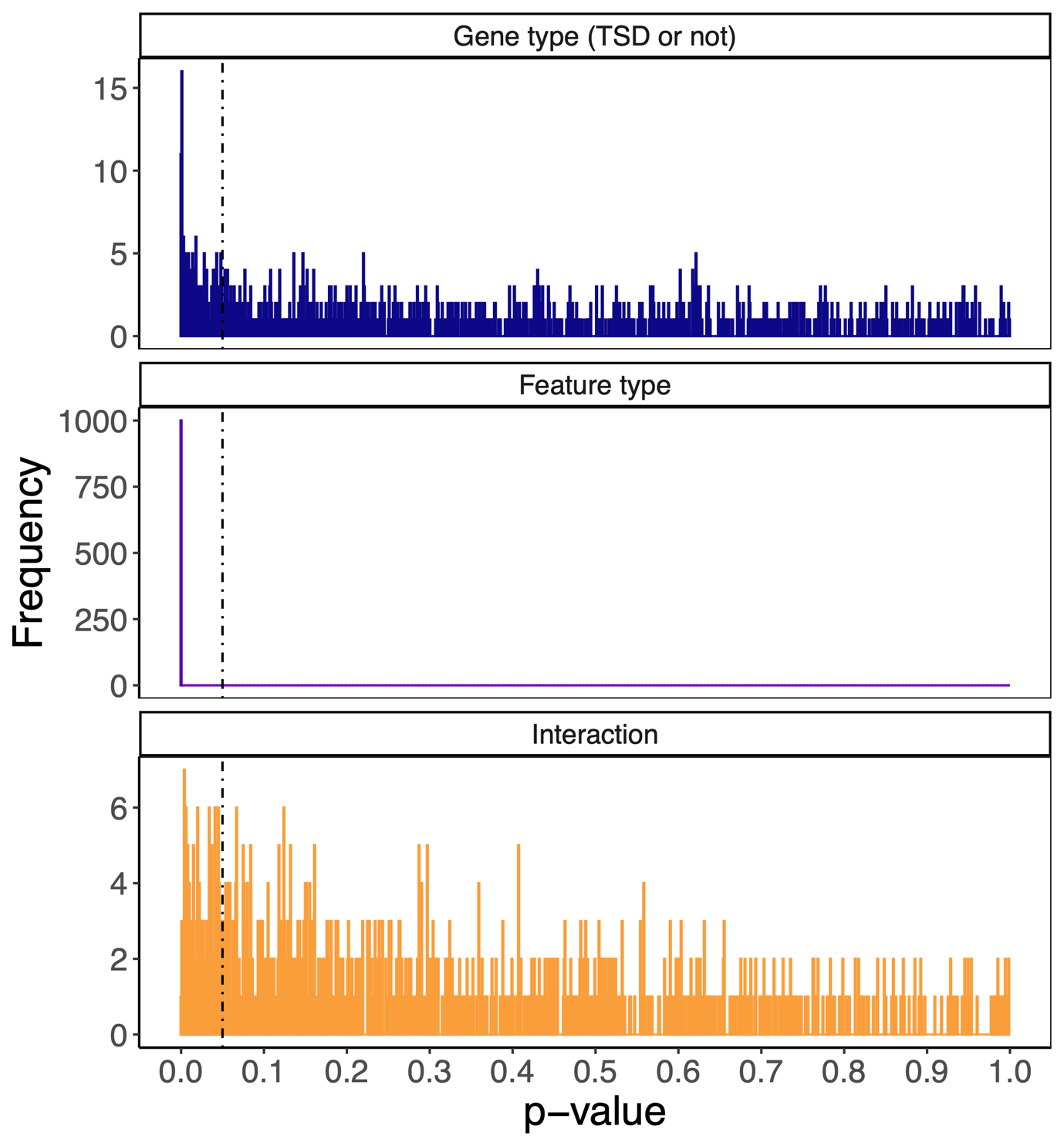


**Figure S10. P-value histogram from comparing the proportion of highly methylated CpGs between TSD-linked genes and non-TSD-linked genes.** A quasipoisson generalised linear model was used to test if the count of highly methylated CpGs differed between 199 TSD-linked genes and 1000 random subsets of 199 non-TSD-linked genes, sampled from 11,560 single-copy orthologues shared between sea turtle species. An offset of total CpG count was included in the model. Histograms are plotted for the p-values per iteration (n=1000), for each term in the ANOVA results table: gene category (TSD-linked versus non-TSD-linked, dark blue), genomic feature type (purple) and their interaction (Gene category x Feature type, yellow). The dashed line represents p=0.05. 171 (17.1%) tests passed p<0.05 for gene category, 1000 (100%) tests passed for feature type and 152 (1.52%) tests passed for the interaction term.

**SUPPLEMENTARY REFERENCES**

Akalin A, Franke V, Vlahoviček K, Mason CE, Schübeler D. genomation: a toolkit to summarize, annotate and visualize genomic intervals. Bioinformatics. 2015; doi: 10.1093/bioinformatics/btu775.

Akalin A, Kormaksson M, Li S, Garrett-Bakelman FE, Figueroa ME, Melnick A, et al. methylKit: a comprehensive R package for the analysis of genome-wide DNA methylation profiles. Genome Biology. 2012; doi: 10.1186/gb-2012-13-10-r87.

Altschul SF, Gish W, Miller W, Myers EW, Lipman DJ. Basic local alignment search tool. Journal of Molecular Biology. 1990; doi: 10.1016/S0022-2836(05)80360-2.

Baltazar-Soares M, Klein JD, Correia SM, Reischig T, Taxonera A, Roque SM, et al. Distribution of genetic diversity reveals colonization patterns and philopatry of the loggerhead sea turtles across geographic scales. Scientific Reports. Nature Publishing Group. 2020; doi: 10.1038/s41598-020-74141-6.

Bentley BP, Carrasco-Valenzuela T, Ramos EKS, Pawar H, Souza Arantes L, Alexander A, et al. Divergent sensory and immune gene evolution in sea turtles with contrasting demographic and life histories. Proceedings of the National Academy of Sciences. 2023; doi: 10.1073/pnas.2201076120.

Cabanettes F, Klopp C. D-GENIES: dot plot large genomes in an interactive, efficient and simple way. PeerJ. 2018; doi: 10.7717/peerj.4958.

Cristofari R. (2022). merge_CpG.py. https://github.com/rcristofari/penguin-tools/blob/master/merge_CpG.py. Accessed April 2023.

Dainat J. (2022). AGAT: Another Gff Analysis Toolkit to handle annotations in any GTF/GFF format (Version 0.9.1). Zenodo. 10.5281/zenodo.6488306.

Drosopoulou E, Tsiamis G, Mavropoulou M, Vittas S, Katselidis KA, Schofield G, et al. The complete mitochondrial genome of the loggerhead turtle Caretta caretta (Testudines: Cheloniidae): Genome description and phylogenetic considerations. Mitochondrial DNA. Taylor & Francis; 2012; doi: 10.3109/19401736.2011.637109.

Heckwolf MJ, Meyer BS, Häsler R, Höppner MP, Eizaguirre C, Reusch TBH. Two different epigenetic information channels in wild three-spined sticklebacks are involved in salinity adaptation. Science Advances. American Association for the Advancement of Science. 2020; doi: 10.1126/sciadv.aaz1138.

Klughammer J, Romanovskaia D, Nemc A, Posautz A, Seid CA, Schuster LC, et al. Comparative analysis of genome-scale, base-resolution DNA methylation profiles across 580 animal species. Nat Commun. Nature Publishing Group. 2023; doi: 10.1038/s41467-022-34828-y.

Krueger F, Andrews SR. Bismark: a flexible aligner and methylation caller for Bisulfite-Seq applications. Bioinformatics. 2011; doi: 10.1093/bioinformatics/btr167.

Laetsch DR, Blaxter ML. BlobTools: Interrogation of genome assemblies. F1000Res. 2017; doi: 10.12688/f1000research.12232.1.

Laine VN, Sepers B, Lindner M, Gawehns F, Ruuskanen S, van Oers K. An ecologist’s guide for studying DNA methylation variation in wild vertebrates. Molecular Ecology Resources. 2023; doi: 10.1111/1755-0998.13624.

Lawrence M, Huber W, Pagès H, Aboyoun P, Carlson M, Gentleman R, et al. Software for Computing and Annotating Genomic Ranges. PLOS Computational Biology. Public Library of Science. 2013; doi: 10.1371/journal.pcbi.1003118.

Leinonen R, Sugawara H, Shumway M. The Sequence Read Archive. Nucleic Acids Res. 2011; doi: 10.1093/nar/gkq1019.

Li H, Handsaker B, Wysoker A, Fennell T, Ruan J, Homer N, et al. The Sequence Alignment/Map format and SAMtools. Bioinformatics. 2009; doi: 10.1093/bioinformatics/btp352.

Li H, Durbin R. Inference of Human Population History From Whole Genome Sequence of A Single Individual. Nature. 2011; doi: 10.1038/nature10231.

Li H. Aligning sequence reads, clone sequences and assembly contigs with BWA-MEM. arXiv. 2013; 10.48550/arXiv.1303.3997.

Mapleson D, Garcia Accinelli G, Kettleborough G, Wright J, Clavijo BJ. KAT: a K-mer analysis toolkit to quality control NGS datasets and genome assemblies. Bioinformatics. 2017; doi: 10.1093/bioinformatics/btw663.

Martin M. Cutadapt removes adapter sequences from high-throughput sequencing reads. EMBnet.journal. 2011; doi: 10.14806/ej.17.1.200.

Meng G, Li Y, Yang C, Liu S. MitoZ: a toolkit for animal mitochondrial genome assembly, annotation and visualization. Nucleic Acids Research. 2019; doi: 10.1093/nar/gkz173.

R Core Team. R: A Language and Environment for Statistical Computing. Vienna, Austria: R Foundation for Statistical Computing; 2021. https://www.R-project.org/. Accessed December 2022.

Sayers EW, Bolton EE, Brister JR, Canese K, Chan J, Comeau DC, et al.. Database resources of the National Center for Biotechnology Information. Nucleic Acids Res. 2021; doi: 10.1093/nar/gkab1112.

Simão FA, Waterhouse RM, Ioannidis P, Kriventseva EV, Zdobnov EM. BUSCO: assessing genome assembly and annotation completeness with single-copy orthologs. Bioinformatics. 2015; doi: 10.1093/bioinformatics/btv351.

Smit AFA, Hubley R, Green P. (2022). RepeatMasker (Version 4.1.4). <http://www.repeatmasker.org>.

Tolve L, Iannucci A, Garofalo L, Ninni A, Capobianco Dondona A, Ceciarini I, et al.. Whole mitochondrial genome sequencing provides new insights into the phylogeography of loggerhead turtles (Caretta caretta) in the Mediterranean Sea. Mar Biol. 2023; doi: 10.1007/s00227-023-04325-x.

Vurture GW, Sedlazeck FJ, Nattestad M, Underwood CJ, Fang H, Gurtowski J, et al.. GenomeScope: fast reference-free genome profiling from short reads. Bioinformatics. 2017; doi: 10.1093/bioinformatics/btx153.

Wreczycka K, Gosdschan A, Yusuf D, Grüning B, Assenov Y, Akalin A. Strategies for analyzing bisulfite sequencing data. Journal of Biotechnology. 2017; doi: 10.1016/j.jbiotec.2017.08.007.
