# Supplementary material for: Chromosome-level genome assembly and methylome profile yield insights for the conservation of endangered loggerhead sea turtles
Source: Gigascience. 2025 Jun 6;14:giaf054. doi: 10.1093/gigascience/giaf054 (PMC12143204; doi:10.1093/gigascience/giaf054)
Supplement: giaf054_GIGA-D-24-00386_original_submission [file giaf054_giga-d-24-00386_original_submission.pdf]

GigaScience

Chromosome-level genome assembly and methylome profile yield insights for the conservation of endangered loggerhead sea turtles

--Manuscript Draft--

**Manuscript Number:**	GIGA-D-24-00386
**Full Title:**	Chromosome-level genome assembly and methylome profile yield insights for the conservation of endangered loggerhead sea turtles
**Article Type:**	Data Note
**Funding Information:**	Natural Environment Research Council (NE/V001469/1) — Prof Christophe Eizaguirre<br>Natural Environment Research Council (NE/X012077/1) — Prof Christophe Eizaguirre<br>National Geographic Society (NGS-59158R-19) — Prof Christophe Eizaguirre<br>Natural Environment Research Council (NE/S007229/1) — Miss Eugenie C Yen

Abstract:

Background: Characterising genetic and epigenetic diversity is crucial for assessing the adaptive potential of threatened populations and species in the face of climate change. Sea turtles are particularly vulnerable due to their temperature-dependent sex determination (TSD) system, which increases feminisation and extinction risk under future climate scenarios. High-quality genomic and epigenomic resources will therefore support conservation efforts for these endangered flagship species with such plastic traits.

Findings: We generated a chromosome-level genome assembly for the loggerhead sea turtle (Caretta caretta) from the globally important Cabo Verde rookery. Using Oxford Nanopore Technology (ONT) and Illumina reads followed by homology-guided scaffolding, we achieved a contiguous (N50: 129.7 Mbp) and complete (BUSCO: 97.1%) assembly, with 98.9% of the genome scaffolded into 28 chromosomes and 29,883 annotated genes. We then extracted the ONT-derived methylome and validated it via whole genome bisulfite sequencing of ten loggerheads from the same population. Applying our novel resources, we reconstructed population size fluctuations and matched them with major climatic events and niche availability. We identified microchromosomes as key regions for monitoring genetic diversity and epigenetic flexibility. Isolating 191 TSD-linked genes, we further built the largest network of functional associations and methylation patterns for sea turtles to date.

Conclusions: We present a high-quality loggerhead sea turtle genome and methylome from the globally significant East Atlantic population. By leveraging ONT sequencing, we generate genomic and epigenomic resources simultaneously, and showcase the potential of this approach for driving molecular insights for conservation of endangered sea turtles.

**Corresponding Author:**	Eugenie C Yen, BA (Hons), MPhil<br>Queen Mary University of London<br>London, UNITED KINGDOM
**Corresponding Author Secondary Information:**	
**Corresponding Author's Institution:**	Queen Mary University of London
**Corresponding Author's Secondary Institution:**	
**First Author:**	Eugenie C Yen, BA (Hons), MPhil
**First Author Secondary Information:**	
**Order of Authors:**	Eugenie C Yen, BA (Hons), MPhil
	James D Gilbert
	Alice Balard

Powered by Editorial Manager® and ProduXion Manager® from Aries Systems Corporation

	Albert Taxonera
	Kirsten Fairweather
	Heather L Ford
	Doko-Miles J Thorburn
	Stephen J Rossiter
	José M Martín-Durán
	Christophe Eizaguirre
**Order of Authors Secondary Information:**	
**Additional Information:**	
**Question**	**Response**
Are you submitting this manuscript to a special series or article collection?	No
**Experimental design and statistics**<br><br>Full details of the experimental design and statistical methods used should be given in the Methods section, as detailed in our Minimum Standards Reporting Checklist. Information essential to interpreting the data presented should be made available in the figure legends.<br><br>Have you included all the information requested in your manuscript?	Yes
**Resources**<br><br>A description of all resources used, including antibodies, cell lines, animals and software tools, with enough information to allow them to be uniquely identified, should be included in the Methods section. Authors are strongly encouraged to cite Research Resource Identifiers (RRIDs) for antibodies, model organisms and tools, where possible.<br><br>Have you included the information requested as detailed in our Minimum Standards Reporting Checklist?	Yes
**Availability of data and materials**	No

Powered by Editorial Manager® and ProduXion Manager® from Aries Systems Corporation

All datasets and code on which the conclusions of the paper rely must be either included in your submission or deposited in publicly available repositories (where available and ethically appropriate), referencing such data using a unique identifier in the references and in the “Availability of Data and Materials” section of your manuscript.<br><br>Have you have met the above requirement as detailed in our Minimum Standards Reporting Checklist?	
If not, please give reasons for any omissions below.<br><br>as follow-up to "**Availability of data and materials**<br><br>All datasets and code on which the conclusions of the paper rely must be either included in your submission or deposited in publicly available repositories (where available and ethically appropriate), referencing such data using a unique identifier in the references and in the “Availability of Data and Materials” section of your manuscript.<br><br>Have you have met the above requirement as detailed in our Minimum Standards Reporting Checklist?<br><br>"	All sequencing data is available on ENA via study accession PRJEB79015. Genome assemblies, annotations and other data files are in the process of being uploaded to ENA, and thus are not publicly available yet, but will be made available to reviewers on GigaDB.

Manuscript

Click here to access/download;Manuscript;Yen2024_Manuscript.docx

Chromosome-level genome assembly and methylome profile yield insights for the conservation of endangered loggerhead sea turtles

Authors

Eugenie C. Yen[1*], James D. Gilbert[1], Alice Balard[1], Albert Taxonera[2], Kirsten Fairweather[2], Heather L. Ford[3], Doko-Miles J. Thorburn[1], Stephen J. Rossiter[1], José M. Martín-Durán[1], Christophe Eizaguirre[1]

Affiliations

[1] School of Biological and Behavioural Sciences, Queen Mary University of London, London, E1 4DQ, UK

[2] Project Biodiversity, Mercado Municipal, local 22 Santa Maria, Ilha do Sal, Cabo Verde

[3] School of Geography, Queen Mary University of London, London, E1 4NS, UK

Emails

Eugenie C. Yen: e.yen@qmul.ac.uk (* Corresponding Author)

James D. Gilbert: j.gilbert@qmul.ac.uk

Alice Balard: alice.cam.balard@gmail.com

Albert Taxonera: albert.taxo@projectbiodiversity.org

Kirsten Fairweather: seaturtles@projectbiodiversity.org

Heather L. Ford: h.ford@qmul.ac.uk

Doko-Miles J. Thorburn: miles.thorburn@gmail.com

Stephen J. Rossiter: s.j.rossiter@qmul.ac.uk

José M. Martín-Durán: chema.martin@qmul.ac.uk

Christophe Eizaguirre: c.eizaguirre@qmul.ac.uk

ABSTRACT

Background: Characterising genetic and epigenetic diversity is crucial for assessing the adaptive potential of threatened populations and species in the face of climate change. Sea turtles are particularly vulnerable due to their temperature-dependent sex determination (TSD) system, which increases feminisation and extinction risk under future climate scenarios. High-quality genomic and epigenomic resources will therefore support conservation efforts for these endangered flagship species with such plastic traits.

Findings: We generated a chromosome-level genome assembly for the loggerhead sea turtle (*Caretta caretta*) from the globally important Cabo Verde rookery. Using Oxford Nanopore Technology (ONT) and Illumina reads followed by homology-guided scaffolding, we achieved a contiguous (N50: 129.7 Mbp) and complete (BUSCO: 97.1%) assembly, with 98.9% of the genome scaffolded into 28 chromosomes and 29,883 annotated genes. We then extracted the ONT-derived methylome and validated it via whole genome bisulfite sequencing of ten loggerheads from the same population. Applying our novel resources, we reconstructed population size fluctuations and matched them with major climatic events and niche availability. We identified microchromosomes as key regions for monitoring genetic diversity and epigenetic flexibility. Isolating 191 TSD-linked genes, we further built the largest network of functional associations and methylation patterns for sea turtles to date.

Conclusions: We present a high-quality loggerhead sea turtle genome and methylome from the globally significant East Atlantic population. By leveraging ONT sequencing, we generate genomic and epigenomic resources simultaneously, and showcase the potential of this approach for driving molecular insights for conservation of endangered sea turtles.

Keywords: reference genome, loggerhead sea turtle, endangered species, epigenomics, DNA methylation, temperature-dependent sex determination, Oxford Nanopore Technology

BACKGROUND

With biodiversity declining at an alarming rate [1], genomic tools are increasingly being deployed to inform conservation management strategies for endangered species [2]. Characterising genetic diversity [3, 4], inbreeding [5], demographic history [6], and locally adapted genomic regions [7] can all provide insights into the adaptive potential of populations and species [8]. Conservation epigenomics has recently gained momentum, driven by technological advancements and falling sequencing costs [9]. This is a promising approach as it offers an additional layer of molecular information that is directly linked to the environment [10]. For instance, quantifying epigenetic variation can aid in assessing a population's capacity for adaptive plastic responses, or provide biomarkers that reflect individual health and environmental exposure [9, 11, 12]. In particular, DNA methylation—the addition of a methyl group to cytosine residues to regulate gene expression—is the best-described epigenetic modification in non-model species to date [13].

Sea turtles comprise seven species, with six assigned as Vulnerable to Critically Endangered and one as Data Deficient by the IUCN Red List [14]. Beyond threats such as bycatch, poaching, and coastal development [15], sea turtles are climate-vulnerable because of their temperature-dependent sex determination (TSD) system, wherein higher incubation temperatures induce female development [16]. As multiple theoretical studies have predicted complete feminisation and subsequent population collapse by 2100 in line with future climate scenarios [17, 18], it is essential to assess whether sea turtles can sustain viable sex ratios via adaptive responses [19, 20]. Although their capacity for genetic evolution is constrained by long generation times and small effective population sizes [21], plastic responses via epigenetic mechanisms could offer alternative pathways, especially as they already play a role in TSD regulation [22, 23, 24]. A lack of high-quality reference genomes previously hindered such

molecular insights in sea turtles. However, this situation is changing through the efforts of international sequencing consortia. Chromosome-level genome assemblies have now been released for the green (*Chelonia mydas)* and leatherback (*Dermochelys coriacea*) sea turtles by the Vertebrate Genomes Project (VGP) [25], and a loggerhead sea turtle (*Caretta caretta*) from the Adriatic Sea by the Canada BioGenome Project (CBP) [26]. Yet, given the well-known influence of reference bias on downstream population-based analyses, continuing to build genome resources remains crucial [27].

Here we present a chromosome-level assembly for a loggerhead sea turtle from the Cabo Verde (East Atlantic) nesting aggregation (**Figure 1A**, Population IUCN Red List Status: Endangered), which is now the largest worldwide for this species [28]. The population is composed of genetically distinct nesting groups maintained by strong female philopatry across the archipelago [29]. A reference assembly for this globally important rookery will improve genomic studies by eliminating reference bias, as well as provide a high-quality loggerhead genome from the East Atlantic nesting group for comparative studies. We simultaneously present the first methylome profile derived from Oxford Nanopore Technology (ONT) reads for a sea turtle species, validated with whole genome bisulfite sequencing (WGBS) of ten loggerheads from the same population. We next applied these novel resources to describe synteny, demographic history, and genomic properties. Lastly, we described the chromosomal locations of over 190 TSD-linked genes, then created a map of their methylation status and predicted functional associations, which will be a useful resource for future epigenetic studies of these endangered TSD species.

METHODS

Reference sample collection

On the 31st of August 2020, we sampled blood from a wild female loggerhead turtle (ID: SLK063, Permit: 013/DNA/2020) that nested on Sal Island of the Cabo Verde Archipelago. Here, the nesting season extends between late June and October. The sampling site (16.62123 °N, -22.92972 °E) was on Algodoeiro Beach, which consists of 800 m of sandy coastline. Blood was collected from the dorsal cervical sinus with a 40 mm, 21-gauge needle and 5 ml syringe following oviposition [30]. A Passive Integrated Transponder tag was added to the front right flipper for identification [31]. The sample was stored in a lithium heparin tube then centrifuged for 1 min at 3000 rpm to separate plasma and blood cells. Samples were stored at -18°C during the field season, then at -80°C following transport to Queen Mary University of London (London, UK).

DNA extraction, sequencing and quality control

Genomic DNA was extracted from blood cells using the QIAGEN Genomic-Tips 100G Kit (Qiagen, Germany). For ONT sequencing, libraries were constructed using an SQK-LSK109 Ligation Sequencing Kit and sequencing was conducted on the PromethION 24 platform with a FLO-PRO002 flow cell (Oxford Nanopore Technologies, UK). Base calling was performed via Guppy v.4.0.11 in high-accuracy mode [32]. This generated 11,643,721 reads (50,075,750,398 bp, ~23.3X coverage) with an N50 of 8,230 bp (base pairs). All downstream bioinformatic steps were conducted on the Apocrita High Performance Computing Cluster [33]. Adapters were trimmed with PoreChop v.0.2.4 [34] and reads were filtered for a Phred score >Q8 and length >500 bp with NanoFilt v.2.6.0 [35]. This passed 8,288,359 reads (40,371,088,741 bp, 80.6%), with an N50 of 8518 bp.

We also generated Illumina sequencing data for polishing. Libraries were constructed by fragmenting DNA via sonification, end polishing, A-tailing and adapter ligation, polymerase

chain reaction (PCR) amplification with P5 and indexed P7 oligos, and purification with the AMPure XP system. Sequencing was performed with 150 bp paired end reads on the NovaSeq 6000 platform (Illumina, USA). This gave 1,050,248,476 reads (157,537,271,400 bp, ~73.3X coverage). To aid downstream parameter selection, haploid genome size, heterozygosity and repeat content were estimated via GenomeScope (**Figure S1)** [36]. Reads were trimmed for adapters and filtered for a Phred score >Q20 with TrimGalore v.0.6.5 [37], passing 1,050,248,476 reads (156,382,436,354 bp, 99.3%).

De novo assembly

ONT reads were assembled using Flye v.2.8.3 [38] in '--asm-coverage 40' mode. A polished consensus sequence was produced using Medaka v.1.3.3 with the 'r941_prom_high_g4011' model [39]. Using our high-coverage Illumina reads from the same sample, two rounds of error polishing were performed with Pilon v.1.24 [40]. The contamination level was assessed via BlobTools v.1.1.1 [41] with Diamond BLASTx v.2.0.11 [42], comparing against the 2021_03 release of the UniProt reference proteomes database [43]. The assembly was haploidised using Purge_Dups v.1.2.5 [44] to give a contig-level assembly "CarCar_QM_v1_2021_12". In addition, we assembled and annotated the mitochondrial genome from our Illumina data with MitoZ v.3.4 (see **Text S1** for extended methods) [45].

Homology-guided scaffolding

We used the chromosome-scale loggerhead assembly produced by the CBP [26] for homology-guided scaffolding. Unplaced contigs were removed with SAMtools v.1.9 [46]. The remaining 28 chromosomal scaffolds served as a reference for homology-based scaffolding into chromosomes using RagTag v.2.1.0 [47]. We supply two versions of our assembly: (1) 'CarCar_QM_v1_2021_12_Scaff' with 28 chromosomes and unplaced contigs/scaffolds, and

(2) ‘CarCar_QM_v1_2021_12_Scaff_With_Chr0’ where unplaced contigs/scaffolds were concatenated into a single scaffold ‘SLK063_ragtag_chr0’ with 100 bp of Ns as gap padding. Note this artificial chromosome was created as an option to aid certain computational analyses by reducing assembly fragmentation, and does not represent true positional information.

Assembly quality assessment

Contiguity was evaluated with QUAST v.5.0.2 [48]. Completeness was assessed with Benchmarking Universal Single-Copy Ortholog (BUSCO) scores, using BUSCO v.5.1.2 [49] in genome mode against the ‘sauropsida_odb10’ database (n=7480 BUSCOs). K-mer-based assessments were performed by comparing k-mers from our Illumina reads against the assembly with parameter K=21. A k-mer spectrum was produced using KAT v.2.4.1 [50] and an assembly consensus quality value (QV) was calculated with Merqury v.1.3 [51]. Contiguity and completeness comparisons were also conducted against all assemblies available for sea turtles: (1) ‘CarCar_GSC_CCare_1.0’ produced by the CBP for the Adriatic loggerhead turtle [26], (2) ‘rDerCor1.pri.v4’ and (3) ‘rCheMyd1.pri.v2’ produced by the VGP for the leatherback and green turtle respectively [25], and (4) ‘CheMyd_1.0’ which was the first draft assembly for the green turtle [52].

Genome annotation

A repeat library was built using RepeatModeler v.2.0.4 in ‘LTRStruct’ mode to discover lateral terminal repeats [53]. To exclude potential gene families, the repeat library was compared against the proteome of the VGP green sea turtle [25] via Diamond BLASTp v.2.0.11 [42]. Transposable elements (TEs) were classified using TEclass [54], then the curated repeat library was used for annotation via RepeatMasker v.4.1.4 [55].

For gene annotation, paired-end RNA-Seq reads (n=746,132,735, **Table S1**) from 24 loggerheads across three life stages (hatchling, juvenile and adult), four tissue types (blood, gonad, brain and heart) and both sexes were mined from the Sequence Read Archive [56, 57, 58, 59]. Reads were trimmed with Trimmomatic v.0.36 [60], mapped with STAR v.2.7.10a [61], and sorted with SAMtools v.1.9 [46]. Alignments were then supplied to BRAKER1 for gene prediction [62]. Note that species-specific training was attempted but resulted in a poorer annotation. Chicken (*Gallus gallus domesticus*) pre-trained parameters were hence chosen, as they were the most related species available. ProtHint v.2.6.0 [63] was used to prepare protein hints from OrthoDB's odb10 Vertebrata database [64], as well as predicted proteomes of the VGP green and leatherback sea turtle [25]. These were supplied to BRAKER2 for gene prediction. TSEBRA v.1.0.3 [65] was then implemented in 'pref_braker1' mode to combine BRAKER1 and BRAKER2 outputs into a set of best predictions.

Concurrently, gene prediction followed the Mikado pipeline v.2.2.4 [66] with whole transcriptome and transcript-based hints. Transcriptomes included a blood transcriptome produced from eight loggerheads across three life stages [59], and a transcriptome we assembled *de novo* using Trinity v.2.14 [67] with RNA-Seq data (**Table S1**) for gonad, brain, and heart tissue of three hatchlings [58]. Transcriptomes were mapped to our assembly via GMAP v.2021-12-17 [68], with a 99.56% and 99.98% alignment rate, respectively. From our STAR alignments, curated intron junctions were produced with Portcullis v.1.2.3 [69] and open reading frames were calculated using TransDecoder v.5.5.0 [70]. All evidence types were subsequently supplied for gene prediction by Mikado.

The BRAKER and Mikado gene sets were merged using the PASA pipeline v.2.5.2 [71] with three rounds of comparison. Finally, the gene set was filtered and standardised with AGAT

v.0.9.1 [72], and in-frame stop codons were removed with gffread v.0.12.7 [73]. To assess completeness, BUSCO v.5.1.2 [49] was run on the longest isoforms in protein mode against the 'sauropsida_odb10' database (n=7480 BUSCOs). Homology-based functional information was assigned via Diamond BLASTp v.2.0.11 [42] against the SwissProt database v.2022_03_02 [43]. Gene Ontology (GO) terms were added using InterProScan 5 v.5.60-92.0 with HMMER databases: Gene3D-4.3.0, PANTHER-17.0, Pfam-35.0, PIRSR-2021, SFLD-4, SUPERFAMILY-1.75, TIGRFAM-15.0 [74]. All functional annotations were attached via MAKER v. 2.31.9 [75].

ONT methylation call and validation with WGBS

From our ONT reads, we called 5-methylcytosine (5mC) and 5-hydroxymethylcytosine (5hmC) modifications in the CpG (5'-C-phosphate-G-3) context via Guppy v.6.5.7 [32] in configuration mode 'dna_r9.4.1_450bps_modbases_5hmc_5mc_cg_hac_prom'. We focused on CpGs as this is the main methylation context in vertebrates [76]. As methylation occurs symmetrically at CpG sites in vertebrates [76], calls were de-stranded per CpG then converted to bedMethyl files using Modkit v.0.1.9 mpileup [77]. This retained 26,449,075 CpGs.

To validate that our ONT-derived methylation call was comparable to a gold-standard method, we generated WGBS-derived methylomes of ten nesting loggerheads from the same population and locality as the reference individual (**Table S3**), using the same sampling protocol. Genomic DNA was extracted with a QIAGEN DNeasy® Blood and Tissue Kit (Qiagen, Germany). DNBseq libraries were constructed and sequenced with 100 bp paired-end reads on an MGI DNBSEQ platform (BGI, Hong Kong), generating 132,192,522 ± 40,360 (SD) reads per sample (**Table S4**). Full methods for methylation calling are available in **Text S2.** Briefly, alignment and methylation calling were performed via Bismark v.0.22.1 [78], with 79.1 ± 3.37

(SD) % mapping efficiency. CpGs were de-stranded using the ‘merge_CpG.py’ script [79], resulting in a coverage of 9.2 ± 0.33 (SD) X (**Table S4**). Using the R package methylKit v.1.24.0 [80], CpGs were filtered for coverage between 5X and the 99.9th percentile [81], then retained if they were covered in >75% of individuals, leaving 24,299,151 CpGs. For each CpG, a methylation percentage was calculated per individual with methylKit’s ‘percMethylation’ function. The mean across all individuals provided an average WGBS methylome representative of the population.

To compare ONT and WGBS methylation calls, 5mC and 5hmC modifications were merged for the ONT data because WGBS cannot distinguish between them. As methylation patterns are known to differ between genomic feature types [82], we calculated mean methylation across four categories (promoter, exon, intron and intergenic regions; see extended methods in **Text S3**) with the R packages genomation v.1.30.0 [83] and GenomicRanges v.1.50.2 [84]. A chi-squared test was used to evaluate if highly methylated (>70%) CpGs [85] were distributed differently across feature types between ONT and WGBS data. Secondly, a linear model was used to test if mean methylation per gene was correlated between ONT and WGBS data, interacting by feature type. The gene-associated feature types included were promoters, exons, introns and intergenic regions <10 kbp (kilo base pairs) from the nearest transcription start site (TSS) [86]. All statistical analyses were conducted in R v.4.2.2 [87] and all plots were produced with the R package ggplot2 v.3.4.2 [88].

Genome-wide synteny between sea turtle species

To investigate genome-level synteny between sea turtle species, we mapped our loggerhead assembly against the VGP green and leatherback assemblies [25]. Genomes were aligned using

minimap2 v.2.18-r1015 with parameter '-f 0.02' [89] and dot plots were produced in D-GENIES v.1.5.0 [90].

Demographic history

We performed Pairwise Sequentially Markovian Coalescent (PSMC) analysis [91] to reconstruct the effective population size (Ne) of the Cabo Verdean loggerhead population (East Atlantic), using our Illumina reads for the reference individual SLK063 [92]. To extend insights across the Atlantic Ocean, we repeated this analysis with Illumina reads for a loggerhead from a Brazilian (Bahia, West Atlantic) population (BioSample: SAMN20502673, SRA Run: SRR15328383) [93]. Reads were aligned via BWA-MEM v.0.7.17 [94], with a mapping rate of 99.8% for SLK063 and 98.7% for SAMN20502673. Alignments were sorted with SAMtools v.1.9 [46] and duplicates were tagged with Picard MarkDuplicates v.2.26.9 [95]. Variant calling and consensus building were conducted in BCFtools v.1.19 with a base and mapping filter of >Q30 [96]. Sites between a third and twice the mean coverage (SLK063: ~51.7X, SAMN20502673: ~22.5X) were retained [25]. PSMC v.0.6.5 was run on the eleven macrochromosomes (1.73 Gbp, 80.8% of total assembly) with parameters '-N25 -t15 -r5 -b -p "4+25*2+4+6' [25] and 100 bootstraps over ~17 million years ago (Mya). PSMC was also run on microchromosomes to verify that patterns were similar (**Figure S2)**. Outputs were scaled with a mutation rate of 1.2^{-8} [25] and a generation time of 45 years. This was calculated by adding the age of maturity to half the reproductive longevity [93], where the age of maturity was estimated as ~30 years in Cabo Verde using the length-at-age relationship [97]. Global mean surface temperature anomaly was plotted relative to pre-industrial times with climate data inferred from marine sediments [98, 99].

Genome properties

We used our Illumina data for the reference individual to estimate genome-wide heterozygosity [100]. Reads were aligned via BWA-MEM v.0.7.17 with a mapping rate of 99.6% [94], sorted with SAMtools v.1.9 [46], and PCR duplicates were tagged with Picard MarkDuplicates v.2.26.9 [95]. Variants were called including monomorphic sites with GATK v.4.2.6.1 HaplotypeCaller in '-ERC BP_RESOLUTION' mode, followed by genotyping via GenotypeGVCFs with expected heterozygosity set to 0.00179, as estimated by GenomeScope (**Figure S1**) [101]. Unused alternate alleles were removed, and sites between a third and twice the mean coverage (~56.1X) were retained [25]. Other filters applied were quality by depth >2.0, root mean square mapping quality >50.0, mapping quality rank sum test >−12.5, read position rank sum test >−8.0, Fisher strand bias <60.0 and strand odds ratio <3.0. With the remaining 2,086,358,400 sites, heterozygosity was computed in non-overlapping 100 kbp windows with the 'popgenWindows.py' script in '-indHet' mode [102].

Next, we summarised a selection of genetic and methylation properties per chromosome in our reference assembly, to explore differences between the 11 macrochromosomes and 17 microchromosomes of the loggerhead genome [103]. Genetic properties included were mean heterozygosity, gene density (number of genes by chromosome length) and CpG density (number of CpG sites by chromosome length). Methylation properties included were mean methylation and proportion of highly methylated (>70%) CpGs. Wilcoxon rank-sum tests were used to investigate mean differences between chromosome types per property. To examine relationships between properties, separate linear models were implemented to test whether different genome properties were correlated, with an interaction by chromosome type to test if relationships differed between macro- and microchromosomes.

TSD-linked genes: identification and synteny between sea turtle species

To identify TSD-linked genes in our loggerhead assembly (**Text S4** for extended methods), we used a list of 223 genes compiled by Bentley et al. (2023). These genes have documented links to TSD, primarily from studies of freshwater turtles and alligators [25]. Following manual curation of this list, we identified the longest isoform orthologues of VGP green turtle sequences [25] in our loggerhead genome via BLASTn v.2.11.0 [104]. These were manually verified by integrating BLAST output and gene name information. For loggerhead genes that matched sequences on two chromosomes, both locations were retained if they were syntenic with other species, under the assumption of a conserved duplication across sea turtles. If one sequence was syntenic and the other not, the non-syntenic sequence was removed under the conservative assumption of assembly error. This gave 201 unique TSD-linked genes for downstream analyses. We next assessed the chromosomal locations of TSD-linked genes in our loggerhead assembly against the VGP green and leatherback assemblies. After filtering for genes present in all three species, 199 genes across 205 loci were retained for comparison and plotted with the R package Circlize v.0.4.16 [105].

TSD-linked genes: comparison of methylation patterns

We examined whether methylation differs between TSD-linked and non-TSD-linked genes in the loggerhead blood methylome. For the non-TSD-linked gene set, we used single-copy orthologues representing evolutionarily conserved genes in sea turtles. These were identified with OrthoFinder v.2.5.4 [106] in nucleotide mode between our loggerhead, VGP green, and VGP leatherback genomes [25], excluding TSD-linked genes. Overall, 200 unique TSD-linked genes and 15,041 single-copy orthologues were covered in our loggerhead methylome and included in analyses. We tested whether methylation differs between gene categories. To satisfy statistical assumptions, 1000 random subsamples of 200 orthogroups were generated for comparison against the 200 TSD-linked genes. For each subsample, a linear model was

used to test if mean methylation was associated with gene category, with an interaction by feature type. A quasipoisson generalised linear model was used to test whether the highly methylated CpG count was associated with gene category in an interaction by feature type, with an offset of total CpG count.

TSD-linked genes: a functional association map

We built a functional association network for TSD-linked genes with the STRING v.12.0 database [107]. Protein sequences from the VGP green turtle [25] were available on STRING and used as query sequences. Proteins were retained if they matched the name of the target gene or had a sequence match >80%. This left 191 proteins, which were searched with the options: full STRING network, 0.4 confidence, and 5% false discovery rate stringency. The Markov Clustering algorithm was used to identify clusters in the network with inflation parameter 2.2. Promoter methylation status in our loggerhead genome was annotated onto the protein network per TSD-linked gene, with three categories based on the bimodal distribution observed: high (>70%), low (<30%) and intermediate (30-70%) methylation. A chi-squared test was used to investigate if proportions of promoter methylation categories differed between the five largest clusters.

RESULTS AND DISCUSSION

Genomic resources generated

Genome assembly

By combining long ONT and short Illumina read sequencing, we produced a contig-level assembly (**Table 1**) with a total size of 2.146 Gbp, 1,799 contigs, and an N50 of 5.51 Mbp (Mega base pairs). A blob-plot confirmed minimal contamination, with 99.2% of contigs mapping to Chordata and the remainder yielding no taxonomic hits (**Figure S3).** We also

assembled and annotated the mitochondrial genome from our Illumina reads, consisting of a circular 16,574 bp contig with 37 genes (**Text S1, Figure S4**). Following homology-guided scaffolding against the same species [26], 98.9% (2.123 Gbp) of the assembly was placed into 28 chromosomal scaffolds, with 698 unplaced contigs (22.7 Mbp; 1.06% of assembly). This elevated our assembly to chromosome-level contiguity with an N50 of 129.73 Mbp, comparable to the best assemblies available for sea turtle species (**Figure 1B, Table 1**). With a BUSCO score of 97.1% (Single copy: 96.2%, Duplicated: 0.9%, Fragmented: 0.4%, Missing: 2.5%) our assembly is the most complete among sea turtles after the VGP green genome [25], and the most complete loggerhead genome to date (**Table 1; Table S4** for BUSCO summaries). Quality is further supported by a QV score of Q41.1 (>99.99% assembly accuracy) and a k-mer spectrum indicating successful haploidisation (**Figure S5)**. Overall, these comparisons demonstrate that our loggerhead assembly is a high-quality contribution to sea turtle genome resources.

**Assembly**	**Size (Gb)**	**Total scaffold / contig count**	**Longest scaffold / contig (Mbp)**	**N50 (Mbp)**	**N50 count**	**GC content (%)**	**Complete BUSCOs (%)**
**CarCar_QM_v1_2021_12** (Loggerhead turtle, contig)	2.146	1799	24.43	5.51	118	43.94	97.1%
**CarCar_QM_v1_2021_12_Scaff** (Loggerhead turtle, scaffolded)	2.146	726	352.82	129.73	5	43.94	97.1%
**CarCar_GSC_CCare_1.0** (Loggerhead turtle, scaffolded)	2.134	2008	345.74	130.96	5	44.03	96.1%
**rDerCor1.pri.v4** (Leatherback turtle, scaffolded)	2.165	41	354.45	137.57	5	43.35	96.3%
**rCheMyd1.pri.v2** (Green turtle, scaffolded)	2.134	93	348.27	134.43	5	44.01	97.2%
**CheMyd_1.0** (Green turtle, contig)	2.132	140,023	22.92	4.07	149	43.43	95.9%

Table 1. Comparison of assembly quality metrics across sea turtle species. 'CarCar_QM_v1_2021_12_Scaff' is our chromosome-scale loggerhead (*Caretta caretta*) assembly. 'CarCar_GSC_CCare_1.0' is the chromosome-scale loggerhead assembly released by the Canada BioGenome Project [26] 'rCheMyd1.pri.v2' and 'rDerCor1.pri. are chromosome-scale green (*Chelonia mydas*) and leatherback (*Dermochelys coriacea*) assemblies respectively, released by the Vertebrate Genomes Project [25]. 'CheMyd_1.0' is the first, draft green assembly [52]. BUSCO scores (full summary in **Table S4**) were calculated against the Sauropsida gene set (n=7480) with BUSCO v.5.1.2 [49].

Genome annotation

We identified and masked 924.6 Mbp (43.1% of assembly) of repetitive elements (**Table S5**). A total of 29,883 genes were annotated, of which 23,690 genes (82.0%) were functionally annotated via homology and 16,954 genes (58.7%) were assigned GO terms (**Table S6**). Our annotation had a BUSCO completeness score of 94.7% (Single copy: 93.9%, Duplicated: 0.8%, Fragmented: 1.1%, Missing: 4.2%). This is less complete than existing sea turtle annotations (**Table 1, Table S7**), likely due to the use of sub-optimal parameters for BRAKER training [63, 108]. Future improvement could involve species-specific tuning and manual curation steps.

Methylation call

By calling methylation from our ONT reads, we provide an additional layer of molecular information to facilitate epigenomic insights for sea turtle conservation. In total, 22,327,230 CpGs had only 5mC modifications, 120,983 CpGs had only 5hmC modifications, and 2,986,762 CpGs had a combination of both. To verify our ONT methylation call, we compared calls with ten loggerhead methylomes re-sequenced via WGBS, the current gold-standard for base-resolution methylation analysis [109]. The proportion of highly methylated CpGs was 74.1% for the ONT methylome versus 73.9% for the WGBS methylome. Methylation estimates were therefore consistent between sequencing methods, as well as a reported genome-wide methylation level of ~70% across Testudines [76]. Highly methylated CpGs were also similarly distributed across feature types between sequencing methods (χ^2=0.0387, p=0.998, **Figure 2A**). For 24,884 genes covered by both methods (98.1% of ONT gene set, 98.2% of WGBS gene set), mean methylation per gene was strongly and positively correlated between ONT and WGBS (R^2=0.92, t=1007.2, p<0.0001), with an interaction by feature type, as expected

(WGBS methylation x Feature type: $F_{1,3}$=48.9, p<0.0001, **Figure 2B**). The demonstrated comparability with WGBS supports ONT as a robust alternative approach for methylation analysis [110, 111]. By measuring real-time ionic current fluctuations, ONT enables the simultaneous acquisition of genomic and methylation sequencing data from native DNA. This maximises molecular insights whilst minimising technical biases introduced by amplification and bisulfite conversion. Furthermore, ONT can distinguish between epigenetic modification types which have different regulatory implications, such as 5mC and 5hmC [112].

Application of genomic resources

Genome-wide synteny between sea turtle species

The loggerhead genome was highly syntenic against the green (**Figure 3A)** and leatherback genomes overall (**Figure 3B**). Possible inversions were detected on chromosomes 4 and 9 of the loggerhead genome against both species, which should be verified whether they are true loggerhead-specific rearrangements or assembly artefacts. 90.1% of sequences exhibited >50% similarity and 2.81% lacked a match against the green, versus 3.37% with >50% similarity and 7.57% without a match against the leatherback (**Table S8**). This aligns with phylogenetic expectations, as loggerheads and greens are both members of the Cheloniidae family and diverged ~40 Mya, compared to the deeper split ~75 Mya from leatherbacks in the Dermochelyidae family [93]. Nonetheless, our results extend the observation of high genomic stability in the sea turtle lineage [25].

Demographic history

We used PSMC to reconstruct the effective population size (Ne) of loggerheads from East Atlantic (ID: SLK063, Cabo Verde) and West Atlantic (ID: SAMN20502673, Bahia State, Brazil) [93] populations. An overall decline in Ne was detected across ~17 Mya of

reconstruction (**Figure 4**). In line with contemporary estimates, the East Atlantic population had a higher Ne (~5000) than the West Atlantic population (~2000) near present times, although the West Atlantic population had higher Ne over the populations' histories. Interestingly, Ne fluctuations were similar in timing and amplitude for both populations. This suggests that major climatic and oceanic processes affecting the entire Atlantic Ocean, rather than region-specific events, were the primary drivers of loggerhead demographic changes. Specifically, a population contraction occurred during the onset and intensification of Northern Hemisphere Glaciation (~3.3-2.4 Mya), as global temperatures and atmospheric carbon dioxide dropped and ice sheets expanded [113, 114]. This period of cooling, coupled with the migration of high productivity centres from polar to equatorial regions, likely reduced habitable zones for loggerheads, as reflected in the relatively rapid decrease in Ne during this interval. During the mid-Pleistocene Transition (~1.25-0.60 Mya), prolonged and intensified glacial intervals resulted in glacial cooling and expansion of ice sheets [115]. At that time, productivity centres shifted from equatorial regions toward subpolar regions and were increasingly variable on glacial-interglacial time scales [116, 117]. These shifts may coincide with Ne expansion in loggerheads, which also matches their proposed migration history in the Atlantic Ocean [29]. Altogether, our results emphasise the importance of climate and niche availability for sea turtle population dynamics, which can inform predictive models of demographic responses to current anthropogenic climate change.

Genome properties

Genome-wide heterozygosity was estimated as ~0.12% for our loggerhead genome, in alignment with ~0.11% reported for an Adriatic loggerhead [26]. This places the genomic diversity of loggerheads at approximately four times the heterozygosity of leatherbacks (~0.0029%) and half of greens (~0.25%) [25], likely reflecting their effective population sizes.

Genomic diversity further varied across the 28 chromosomes, with microchromosomes being more heterozygous than macrochromosomes (W=29, p=0.002, **Figure S6A**). Heterozygosity was best predicted by an interaction between chromosome length and type (Length x Type: $F_{1,24}$=12.3, p=0.002, **Figure 3C)**, with a steep negative correlation in microchromosomes ($F_{1,15}$=15.4, p=0.001) but none detected for macrochromosomes ($F_{1,15}$=4.50, p=0.063). Microchromosomes were also more gene-dense (W=28, p=0.001, **Figure S6B**), GC-rich (W=1, p<0.0001, **Figure S6C**) and CpG-dense (W=1, p<0.0001, **Figure S6D**), consistent with patterns described in other sea turtles [25] and wider vertebrates [118, 119].

We next performed comparisons to address the knowledge gap of methylation differences between macro- and microchromosomes. Mean methylation was similar between chromosome types (W=54, p= 0.07, **Figure S6E**), but microchromosomes had a greater proportion of highly methylated CpGs (W=54, p<0.0001; **Figure S6F**). This likely stems from higher CpG density on microchromosomes providing more raw genetic substrate for methylation [120], as supported by a strong, positive correlation ($F_{1,24}$=56.5, p<0.0001, **Figure 3D**) that was independent of chromosome type (CpG density x Type: $F_{1,24}$=0.08, p=0.90). Similar relationships were observed for highly methylated sites against heterozygosity ($F_{1,24}$=50.30, p<0.0001, Heterozygosity x Type: $F_{1,24}$=0.04, p=0.84; **Figure 3E**) and gene density ($F_{1,24}$=65.89, p<0.0001, Gene density x Type: $F_{1,24}$=0.048, p=0.83; **Figure 3F**). Therefore, microchromosomes have greater methylation potential and realised levels than macrochromosomes, with correlations between methylation and other genome properties likely shaped by evolutionary forces that act consistently across chromosome types. These results highlight the specific properties of vertebrate microchromosomes [119] and add novel insights into their methylation potential for sea turtles. With higher heterozygosity, gene density, and regulatory opportunities via CpG methylation, microchromosomes could serve as hotspots for

both adaptive evolution and plastic responses to the environment. As such, microchromosomes offer promising large-scale regions for monitoring and preserving functional genetic and epigenetic diversity in sea turtles [8, 121].

TSD-linked genes: synteny between sea turtle species

As TSD species, loggerheads are particularly vulnerable to climate change, with extreme population feminisation and collapse predicted by the end of the century [20]. To gain molecular insights into this mechanism, we compared the chromosomal locations of 199 TSD-linked genes present in our loggerhead genome, VGP green, and VGP leatherback genomes. Consistent with Bentley et al. (2023), most TSD-linked genes are single-copy and reside on equivalent chromosomes in sea turtle species (**Figure 5A, 5B**). Only the EP300 (E1A binding protein p300) gene was not syntenic between loggerheads and greens (**Figure 5A**). Although it mapped to chromosome 1 in all species, a duplicate on chromosome 10 was found in the green and leatherback genomes. Validation is required to resolve if this represents a true duplicate in greens and leatherbacks that has been lost in loggerheads. Two genes were syntenic between loggerheads and greens but not leatherbacks (**Figure 5B**): CIRBP (Cold-inducible RNA-binding protein; loggerhead/green: chromosome 25; leatherback: chromosome 27) and IFIT5 (Interferon Induced Protein with Tetratricopeptide Repeats 5; loggerhead/green: chromosome 7; leatherback: chromosome 1). These may reflect fission or fusion events after the divergence of the Cheloniidae and Dermochelyidae lineages. As rearrangements and copy number variants can alter gene regulation [122], those three genes may contribute to inter-species differences in the TSD response curve and should be investigated. Nevertheless, such structural variants seem rare in sea turtles overall, unlike in other Testudines [123, 124]. Finer scale genetic variation may instead be more important, particularly within species. For example, a SNP on the CIRBP gene, which we found to be non-syntenic against leatherbacks,

influences TSD in snapping turtles (*Chelydra serpentina*) and exhibits varying allele frequencies with latitude [125]. It is thus crucial to continue characterising genetic variants that contribute to the adaptive potential of the TSD mechanism, to evaluate whether different sea turtle species and populations can evolve to mitigate sex ratio skew in the face of rising temperatures as climate change progresses.

TSD-linked genes: methylation patterns

Given genetic differences between sea turtles are low and epigenetic regulation can contribute to the adaptive potential of TSD species [9, 24], we next provide insights into the methylation status of TSD-linked genes. We tested if methylation differed between 200 TSD-linked genes present in the loggerhead genome, versus 1000 random subsets of 200 non-TSD-linked genes sampled from 15,041 single-copy orthologues identified between sea turtle species. Mean methylation was not associated to gene category (TSD-linked or non-TSD-linked genes), as supported by only 155 out of 1000 (15.5%) tests crossing a 'significance threshold' of $p<0.05$ (**Figure S7**). The interaction between gene category and feature type did not reveal differences either, with 38 out of 1000 (0.38%) tests passing a threshold of $p<0.05$ (**Figure S7**), and similar methylation distributions observed across all feature types (**Figure 5C, 5D**). Instead, feature type was the sole determinant of mean methylation, with all 1000 tests (100%) passing $p<0.05$ (**Figure S7**), as expected given the importance of genomic context on methylation patterns and functions [82]. Similar results were observed when testing associations against the proportion of highly methylated CpGs (**Figure S8**). Exons, introns and gene-associated intergenic space (<10 kbp from TSS) were mostly hypermethylated, with a peak of methylation centred on ~80% (**Figure 5C, 5D**). In contrast, methylation was bimodally distributed for promoters, with peaks of low (~10%) and high (~80%) methylation (**Figure 5C, 5D**). These distributions are consistent with previously described patterns in vertebrates [126, 127].

We characterised large-scale methylation patterns from blood. This is a valuable tissue for conservation monitoring as it can be collected via minimally invasive sampling, yet still report on an individual's physiology [128, 129, 130], health [131], and environmental exposure [132, 133]. For example, a non-lethal method for sexing sea turtle hatchlings is urgently required to assess nest sex ratios as global temperatures continue to rise [20]. Whereas a previous study identified 16 differentially methylated regions between sexes of green turtle adults via reduced representation sequencing, no regions could distinguish sexes for earlier life-stages [134]. Methylome-wide scans should thus be used to identify biomarkers of sex, as conducted in relation to thermal stress in a study that employed this reference assembly [135].

TSD-linked genes: a functional association map

Using the STRING database [107], we built a network of functional associations between proteins coded by 191 TSD-linked genes **(Figure 5E**). In total, 119 TSD-linked genes had at least one connection identified via Markov Clustering (n=28 clusters total), and the five largest clusters encompassed 54 TSD-linked genes (45.4%). Members of the biggest cluster (n=15, dark blue) were mainly linked to Wnt signalling. Connections were centred on CTNNB1 (beta-catenin), which is critical for female determination in vertebrates [136, 137]. The next largest cluster (n=12, dark purple) was primarily composed of epigenetic regulators. The most connected protein was EP300 (E1A binding protein p300), a histone acetyltransferase that controls cell proliferation, with germ cell numbers proposed to influence TSD in a freshwater turtle [138]. The third cluster (n=11, mauve) contained genes with well-described roles in TSD and steroid hormone signalling [139]. SOX9 (SRY-related HMG box gene 9), a key regulator of male differentiation [140, 141], was most connected. Although the functional nature of the fourth cluster (n=8, pink) was less obvious, the most connected protein was the histone

demethylase KDM6B (Lysine Demethylase 6B), demethylase which is causally linked to temperature-dependent male determination in freshwater turtles [23]. The final cluster contains heat shock proteins and chaperones (n=7, yellow), which could play a role in temperature sensing and transduction during TSD [142]. These identified clusters already suggest patterns surrounding TSD in sea turtles. Sensory systems associated with heat shock responses could act as environmental messengers that link with epigenetic regulators to alter gene expression, cell proliferation, and subsequently activate known endocrine systems. With growing genomic tools for sea turtles, this hypothesis will need to be tested.

Given the prevalence of epigenetic regulators in the functional network, we overlaid promoter methylation status of TSD-linked genes from our loggerhead blood methylome (**Figure 5E**). In total, 67 genes (33.8%) were highly (>70%) methylated, 96 genes (48.0%) were lowly (<30%) methylated, and 36 genes (18.2%) had intermediate methylation. The proportions of each promoter methylation category differed amongst the five largest functional clusters (χ^2=41.697, p<0.0001, **Table S9**). The lowest methylated clusters involved Wnt signalling genes (dark blue; high: 33.3%, intermediate: 6.7%, low: 60%) and the well-described TSD-linked/hormone signalling genes (mauve; high: 20%, intermediate: 20%, low: 60%). No clusters were hypermethylated, as the heat shock cluster (yellow) contained the largest proportion of highly methylated genes, with an equal split between highly and lowly methylated genes. With promoter methylation classically linked to transcriptional repression [82], we may have captured female-specific methylation signatures on a gene network scale. By integrating methylation and functional association information into a single map, we facilitate the investigation into the relationships between gene function, connectivity, and epigenetic regulation in the context of TSD for sea turtles.

CONCLUSIONS

To support conservation efforts of threatened loggerhead sea turtles, as well as all sea turtles and TSD species in general, we present a chromosome-scale genome representing the globally significant East Atlantic nesting group. Our reference assembly will enhance genomic inferences for this population, and contribute a new genome for comparative studies within and between species. Moreover, we anticipate our ONT-derived methylome can guide future epigenetic study design. To demonstrate their potential, we applied these novel resources to yield a variety of insights relevant to the conservation of sea turtles. At the population level, we emphasise the role of climate change and niche availability on effective population size fluctuations. On the chromosome level, we recommend microchromosomes as special regions for monitoring functional genetic and epigenetic diversity. Finally, we bring gene-level insights into the TSD cascade of sea turtles, highlighting three TSD-linked genes with potential inter-species rearrangements, and providing a map of functional associations and methylation status for TSD-linked genes to assist sex biomarker discovery. By simultaneously generating genome and methylome resources to provide molecular insights in loggerhead sea turtles, our study showcases the application of this dual framework for informing the conservation of an endangered, flagship species.

DATA AVAILABILITY

All sequencing data reported in this article are available on the European Nucleotide Archive (ENA) repository, under study accession PRJEB79015. Genome assemblies and annotations are in the process of being uploaded to ENA, and can be made available to reviewers. All other supporting data would be uploaded to GigaDB. All scripts will be deposited to our GitHub repository [143].

LIST OF ABBREVIATIONS

5mC: 5-methylcytosine, 5hmC: 5-hydroxymethylcytosine, bp: base pairs, CBP: Canada BioGenome Project, CpG: 5'-C-phosphate-G-3, ENA: European Nucleotide Archive, Gbp: giga base pairs, kbp: kilo base pairs, Mbp: mega base pairs, Mya: million years ago, Ne: effective population size, ONT: Oxford Nanopore Technology, PCR: polymerase chain reaction, PSMC: Pairwise Sequentially Markovian Coalescent, TSS: transcription start site, VGP: Vertebrate Genomes Project, WGBS: whole genome bisulfite sequencing

SUPPLEMENTARY FILES

Table S1. Publicly available RNA-Seq reads mined for genome annotation

Table S2. Metadata for ten nesting loggerheads sampled for WGBS

Table S3. WGBS summary statistics for ten nesting loggerheads

Table S4. Full BUSCO summary for genome assemblies across sea turtle species

Table S5. Repetitive element summary statistics

Table S6. Genome annotation summary statistics

Table S7. Full BUSCO summary for genome annotations across sea turtle species

Table S8. Summary of genome alignment identity between sea turtle species

Table S9. Proportions of promoter methylation categories in the top five functional clusters

Text S1. Extended methods and results for mitochondrial genome assembly and annotation

Text S2. Extended methods for methylation calling from WGBS data of ten loggerheads

Text S3. Extended methods for annotating gene feature types

Text S4. Extended methods for identification and curation of TSD-linked genes

Figure S1. GenomeScope profile for our loggerhead genome

Figure S2. Auxiliary PSMC tests with 100 bootstraps

Figure S3. GC-coverage blob-plot to evaluate assembly contamination

Figure S4. Circos plot of our loggerhead mitochondrial assembly and annotation

Figure S5. K-mer spectrum plot of our loggerhead assembly

Figure S6. Comparison of genome properties between macro- and microchromosomes

Figure S7. P-value histogram from comparing mean methylation between TSD-linked genes and non-TSD-linked genes

Figure S8. P-value histogram from comparing the proportion of highly methylated CpGs between TSD-linked genes and non-TSD-linked genes

DECLARATIONS

Ethics approval

All sample collection adhered to national legislation and were approved by the Direção Nacional do Ambiente de Cabo Verde (Permits: 13/DNA/2020, 037/DNA/2021).

Competing interests

The authors declare they have no competing interests.

Funding

This work was funded by UK Research and Innovation (NERC, NE/V001469/1, NE/X012077/1 to C.E and J.M.M-D) and National Geographic (NGS-59158R-19 to C.E) grants. Additional financial support was awarded by the London NERC Doctoral Training Partnership studentship to E.C.Y (NERC, NE/S007229/1).

Author contributions

E.C.Y and C.E designed the study. E.C.Y, C.E, A.T and K.F collected blood samples. E.C.Y performed genome assembly, annotation and quality assessment, with contributions from

J.M.M-D. A.B, E.C.Y and D-M.J.T performed DNA methylation analysis of ONT reads. E.C.Y performed sample processing and methylation analysis for WGBS. E.C.Y performed genome property and synteny analyses. E.C.Y performed PSMC, with input and guidance on geoclimatic events from H.L.F. J.D.G identified TSD-linked genes, orthogroup genes, mapped chromosomal locations and produced the functional association network. E.C.Y analysed methylation of TSD-linked genes. E.C.Y wrote the manuscript with contributions from C.E, J.D.G, H.L.F, S.J.R, J.M.M-D and feedback from all authors.

Acknowledgements

The authors thank all staff with Project Biodiversity (Sal, Cabo Verde) for their support in the field. We also thank Chloe Economou (Queen Mary University of London, UK) for conducting DNA extractions for the reference individual and William Tyne (Queen Mary University of London, UK) for ONT library preparation and sequencing.

FIGURE LEGENDS

Figure 1. A chromosome-scale genome assembly for endangered loggerhead sea turtles from the East Atlantic nesting group. (A) A loggerhead turtle nesting in Cabo Verde (Sal Island), our reference population. Photo credit: Project Biodiversity. **(B)** Contiguity of publicly available sea turtle genomes. 'CarCar_QM_v1_2021_12_Scaff' (dark blue) is our chromosome-scale loggerhead (*Caretta caretta*) assembly. 'CarCar_GSC_CCare_1.0' (dark purple) is the chromosome-scale loggerhead assembly from the Adriatic Sea, released by the Canada BioGenome Project [26]. 'rCheMyd1.pri.v2' (mauve) and 'rDerCor1.pri.v4' (yellow) are chromosome-scale green (*Chelonia mydas*) and leatherback (*Dermochelys coriacea*) assemblies respectively, released by the Vertebrate Genomes Project [25]. 'CheMyd_1.0'

(pink) is the first, draft green assembly [52]. Our assembly is of comparable contiguity to the best genomes currently available for sea turtles.

Figure 2. Validation of ONT-derived methylome with WGBS of ten loggerheads. (A) Genome-wide distribution of highly methylated (>70%) CpGs across feature types by site. The outer ring shows the distribution called from the ONT methylome of the reference individual (n=19,606,231 CpGs), with 3.74% on exons (yellow), 24.64% on introns (dark purple), 2.90% on promoters (mauve) and 49.89% being intergenic (dark blue). The inner ring shows the distribution called from the average of ten WGBS methylomes (n=17,950,597 CpGs), with 3.96% on exons, 24.96% on introns, 2.78% on promoters and 49.91% being intergenic. Highly methylated CpGs are similarly distributed across feature types between sequencing methods over the entire genome (χ^2=0.0387, p=0.998). **(B)** Correlation of mean methylation (%) per gene (n=24,884) between ONT and average WGBS methylation calls, by feature type: exons, introns, promoters and gene-associated intergenic regions (<10 kbp from TSS). There is a strong, positive correlation between sequencing methods for genes per feature type (R^2=0.92, t=1007.2, p<0.0001).

Figure 3. Comparison of genome-level and chromosome-level characteristics. (A-B) Alignment dotplots of our loggerhead assembly against the Vertebrate Genomes Project [25] **(A)** green assembly and **(B)** leatherback assembly (top 100,000 alignments shown). Axes show chromosome number (1-11: macrochromosomes, 12-28: microchromosomes, 0: unplaced contigs). Colours represent alignment sequence identity (%). There is strong synteny with minimal chromosome-scale structural rearrangements between sea turtle species, supporting high genomic stability. **(C-F)** Relationships between genome properties in macro- (dark blue) versus microchromosomes (yellow) of the loggerhead genome. **(C)** Mean heterozygosity (%)

exhibits an interaction by chromosome length (Gbp) and type ($F_{1,24}$=12.3, p=0.002), with a negative correlation in microchromosomes but no correlation in macrochromosomes. **(D)** The proportion of highly methylated (>70%) CpGs (%) is higher overall for microchromosomes, but the positive correlation with CpG density (total CpGs by chromosome length; $F_{1,24}$=56.5, p<0.0001) does not interact with chromosome type ($F_{1,24}$=0.08, p=0.90). A similar relationship is observed between highly methylated CpGs (%) and **(E)** mean heterozygosity (%; ($F_{1,24}$=50.30, p<0.0001, Interaction: $F_{1,24}$=0.04, p=0.84), and **(F)** gene density (total genes by chromosome length x10^{-5}; $F_{1,24}$=65.89, p<0.0001, Interaction: F1,24=0.048, p=0.83). This suggests that relationships between methylation and other genetic properties are a genome-level property unaffected by chromosome type.

Figure 4. Demographic history reconstruction for loggerhead populations across the East and West Atlantic Ocean. The East Atlantic population is in Cabo Verde (SLK063, blue line) and the West Atlantic population is in Bahia State, Brazil (SAMN20502673, purple line) [93]. Effective population size (Ne, x10^4) was reconstructed using PSMC over ~17 Mya (log-scaled). Marine sediments were used to infer the global mean surface temperature anomaly (red and blue climate stripes) compared to pre-industrial times [98, 99]. Letters designate geoclimatic events of interest: A: Northern Hemisphere Glaciation (~3.3 to 2.4 Mya). During this period, global temperature and atmospheric carbon dioxide decreased and ice sheets expanded [113, 114]. High productivity centres shifted from polar to equatorial regions [116], likely forcing migration and shrinkage of ecological niches. B: Mid-Pleistocene Transition (~1.25 to 0.60 Mya). A period of glacial cooling and ice sheet growth [115] when productivity centres shifted from the equator toward subpolar regions [116, 117], allowing population expansion.

Figure 5. Molecular insights into TSD-linked genes: synteny, methylation and functional associations. (A-B) Chromosomal locations of 199 TSD-linked genes across 205 loci in our loggerhead assembly against the Vertebrate Genomes Project [25] **(A)** green assembly and **(B)** leatherback assembly. Chromosomes of the loggerhead assembly are plotted on the left, with colours representing the 28 chromosomes. Dashed red lines indicate genes that mapped to different locations between species (loggerhead versus green: EP300, loggerhead versus leatherback: EP300, CIRBP, IFIT5). There is overall high synteny of TSD-linked genes between sea turtle species. (**C)** Violin plots of mean methylation (%) per gene by feature type for TSD-linked (n=200 genes; purple) versus non-TSD-linked genes (n=15,041 single-copy orthologues between sea turtle species; yellow). **(D)** Density plots of mean methylation (%) per gene by feature type between 200 TSD-linked genes (purple) and a random subset of 200 non-TSD-linked genes (yellow). Both plots show that methylation is distributed differently across feature types, but similarly between TSD-linked versus non-TSD-linked genes. **(E)** Predicted functional association network of proteins coded by TSD-linked genes, created with STRING [107]. 119 genes with at least one connection are shown (n=28 clusters total). Nodes represent gene IDs and edges represent associations. Edge colours represent different evidence types used for association prediction. Non-grey node colours represent the five largest functional clusters identified via Markov Clustering. Letters on nodes represent methylation status of gene promoters in the reference individual methylome. Categories are based on the bimodal methylation distribution observed in **Figure 5D**: H: highly methylated (>70%), L: lowly methylated (<30%), I: intermediate methylation (30-70%). 67 promoters (33.8%) were hyper-methylated, 96 (48%) were hypo-methylated and 36 (18.2%) had intermediate methylation. Proportions of each promoter methylation category were different amongst the five largest functional clusters (χ^2=41.697, p<0.0001).

REFERENCES

1. Ceballos G, Ehrlich PR, Barnosky AD, García A, Pringle RM, Palmer TM. Accelerated modern human–induced species losses: Entering the sixth mass extinction. *Sci Adv*. 2015; doi: 10.1126/sciadv.1400253.

2. Theissinger K, Fernandes C, Formenti G, Bista I, Berg PR, Bleidorn C, et al. How genomics can help biodiversity conservation. *Trends in Genetics*. 2023; doi: 10.1016/j.tig.2023.01.005.

3. Coates DJ, Byrne M, Moritz C. Genetic Diversity and Conservation Units: Dealing With the Species-Population Continuum in the Age of Genomics. Front Ecol Evol. Frontiers; 2018; doi: 10.3389/fevo.2018.00165.

4. Wold J, Koepfli K-P, Galla SJ, Eccles D, Hogg CJ, Le Lec MF, et al.. Expanding the conservation genomics toolbox: Incorporating structural variants to enhance genomic studies for species of conservation concern. *Molecular Ecology*. 2021; doi: 10.1111/mec.16141.

5. Kardos M, Taylor HR, Ellegren H, Luikart G, Allendorf FW. Genomics advances the study of inbreeding depression in the wild. *Evolutionary Applications*. 2016; doi: 10.1111/eva.12414.

6. Karamanlidis AA, Skrbinšek T, Amato G, Dendrinos P, Gaughran S, Kasapidis P, et al..Genetic and demographic history define a conservation strategy for earth's most

endangered pinniped, the Mediterranean monk seal Monachus monachus. *Sci Rep*. 2021; doi: 10.1038/s41598-020-79712-1.

7. Flanagan SP, Forester BR, Latch EK, Aitken SN, Hoban S. Guidelines for planning genomic assessment and monitoring of locally adaptive variation to inform species conservation. *Evolutionary Applications*. 2018; doi: 10.1111/eva.12569.

8. Eizaguirre C, Baltazar-Soares M. Evolutionary conservation—evaluating the adaptive potential of species. *Evolutionary Applications*. 2014; doi: 10.1111/eva.12227.

9. Balard A, Baltazar-Soares M, Eizaguirre C, Heckwolf MJ. An epigenetic toolbox for conservation biologists. *Evolutionary Applications*. 2024; doi: 10.1111/eva.13699.

10. Feil R, Fraga MF. Epigenetics and the environment: emerging patterns and implications. *Nature Reviews Genetics*. 2012; doi: 10.1038/nrg3142.

11. Rey O, Eizaguirre C, Angers B, Baltazar-Soares M, Sagonas K, Prunier JG, et al. Linking epigenetics and biological conservation: Towards a conservation epigenetics perspective. *Functional Ecology*. 2020; doi: 10.1111/1365-2435.13429.

12. Lamka GF, Harder AM, Sundaram M, Schwartz TS, Christie MR, DeWoody JA, et al. Epigenetics in Ecology, Evolution, and Conservation. *Frontiers in Ecology and Evolution*. 2022; 10.3389/fevo.2022.871791.

13. Laine VN, Sepers B, Lindner M, Gawehns F, Ruuskanen S, van Oers K. An ecologist's guide for studying DNA methylation variation in wild vertebrates. *Molecular Ecology Resources*. 2023; doi: 10.1111/1755-0998.13624.

14. IUCN. The IUCN Red List of Threatened Species (Version 2024-1). 2024; https://www.iucnredlist.org. Accessed July 2024.

15. Wallace BP, DiMatteo AD, Bolten AB, Chaloupka MY, Hutchinson BJ, Abreu-Grobois FA, et al. Global Conservation Priorities for Marine Turtles. *PLOS ONE*. 2011; doi: 10.1371/journal.pone.0024510.

16. Yntema CL, Mrosovsky N. Critical periods and pivotal temperatures for sexual differentiation in loggerhead sea turtles. *Can J Zool*. 1982; doi: 10.1139/z82-141.

17. Hawkes L, Broderick A, Godfrey M, Godley B. Climate change and marine turtles. *Endang Species Res*. 2009; doi: 10.3354/esr00198.

18. Laloë J-O, Cozens J, Renom B, Taxonera A, Hays GC. Effects of rising temperature on the viability of an important sea turtle rookery. *Nature Climate Change*. 2014; doi: 10.1038/nclimate2236.

19. Mitchell N, Janzen FJ. Temperature-Dependent Sex Determination and Contemporary Climate Change. *Sexual Development*. 2010; doi: 10.1159/000282494.

20. Lockley EC, Eizaguirre C. Effects of global warming on species with temperature-dependent sex determination: Bridging the gap between empirical research and management. *Evol Appl*. 2021. doi: 10.1111/eva.13226.

21. Komoroske LM, Jensen MP, Stewart KR, Shamblin BM, Dutton PH. Advances in the Application of Genetics in Marine Turtle Biology and Conservation. *Front Mar Sci*. *Frontiers*. 2017; doi: 10.3389/fmars.2017.00156.

22. Venegas D, Marmolejo-Valencia A, Valdes-Quezada C, Govenzensky T, Recillas-Targa F, Merchant-Larios H. Dimorphic DNA methylation during temperature-dependent sex determination in the sea turtle Lepidochelys olivacea. *General and Comparative Endocrinology*. 2016; doi: 10.1016/j.ygcen.2016.06.026.

23. Ge C, Ye J, Weber C, Sun W, Zhang H, Zhou Y, et al.. The histone demethylase KDM6B regulates temperature-dependent sex determination in a turtle species. *Science*. American Association for the Advancement of Science. 2018; doi: 10.1126/science.aap8328.

24. Piferrer F. Epigenetic mechanisms in sex determination and in the evolutionary transitions between sexual systems. *Philosophical Transactions of the Royal Society B: Biological Sciences*. Royal Society. 2021; doi: 10.1098/rstb.2020.0110.

25. Bentley BP, Carrasco-Valenzuela T, Ramos EKS, Pawar H, Souza Arantes L, Alexander A, et al. Divergent sensory and immune gene evolution in sea turtles with contrasting demographic and life histories. *Proceedings of the National Academy of Sciences*. 2023; doi: 10.1073/pnas.2201076120.

26. Chang G, Jones S, Leelakumari S, Ashkani J, Culibrk L, O'Neill K, et al.. The genome sequence of the Loggerhead sea turtle, Caretta caretta Linnaeus 1758. *F1000Res*. 2023; doi: 10.12688/f1000research.131283.2.

27. Thorburn D-MJ, Sagonas K, Binzer-Panchal M, Chain FJJ, Feulner PGD, Bornberg-Bauer E, et al.. Origin matters: Using a local reference genome improves measures in population genomics. *Molecular Ecology Resources*. 2023; doi: 10.1111/1755-0998.13838.

28. Taxonera A, Fairweather K, Jesus A, Gonzalves A, Queiruga A, et al. Cabo Verde: Sea Turtles "In Abundance". In: SWOT Report - State of the World's Sea Turtles Vol. XVII. 2022; https://www.seaturtlestatus.org/articles/cabo-verde-sea-turtles-in-abundance. Accessed June 2023.

29. Baltazar-Soares M, Klein JD, Correia SM, Reischig T, Taxonera A, Roque SM, et al. Distribution of genetic diversity reveals colonization patterns and philopatry of the loggerhead sea turtles across geographic scales. *Scientific Reports*. 2020; doi: 10.1038/s41598-020-74141-6.

30. Owens DWM, Ruiz GJ. New Methods of Obtaining Blood and Cerebrospinal Fluid from Marine Turtles. *Herpetologica*. 1980; 36:17–20.

31. Stiebens VA, Merino SE, Roder C, Chain FJJ, Lee PLM, Eizaguirre C. Living on the edge: how philopatry maintains adaptive potential. *Proceedings of the Royal Society B: Biological Sciences*. 2013; doi: 10.1098/rspb.2013.0305.

32. Wick RR, Judd LM, Holt KE. Performance of neural network basecalling tools for Oxford Nanopore sequencing. *Genome Biology*. 2019; doi: 10.1186/s13059-019-1727-y.

33. King T, Butcher S, & Zalewski L. (2017). Apocrita - High Performance Computing Cluster For Queen Mary University Of London. *Zenodo*. 10.5281/ZENODO.438045.

34. Wick RR. (2018). PoreChop (Version 0.2.4). https://github.com/rrwick/Porechop/releases/tag/v0.2.4.

35. De Coster W, D'Hert S, Schultz DT, Cruts M, Van Broeckhoven C. NanoPack: visualizing and processing long-read sequencing data. *Bioinformatics*. 2018; doi: 10.1093/bioinformatics/bty149.

36. Vurture GW, Sedlazeck FJ, Nattestad M, Underwood CJ, Fang H, Gurtowski J, et al.. GenomeScope: fast reference-free genome profiling from short reads. *Bioinformatics*. 2017; doi: 10.1093/bioinformatics/btx153.

37. Krueger F. (2019). TrimGalore (Version 0.6.5). https://github.com/FelixKrueger/TrimGalore/releases/tag/0.6.5.

38. Kolmogorov M, Yuan J, Lin Y, Pevzner PA. Assembly of long, error-prone reads using repeat graphs. *Nature Biotechnology*. 2019; doi: 10.1038/s41587-019-0072-8.

39. Oxford Nanopore Technologies. (2021). Medaka (Version 1.3.3). https://github.com/nanoporetech/medaka/releases/tag/v1.3.3.

40. Walker BJ, Abeel T, Shea T, Priest M, Abouelliel A, Sakthikumar S, et al. Pilon: An Integrated Tool for Comprehensive Microbial Variant Detection and Genome Assembly Improvement. *PLOS ONE*. 2014; doi: 10.1371/journal.pone.0112963.

41. Laetsch DR, Blaxter ML. BlobTools: Interrogation of genome assemblies. *F1000Res*. 2017; doi: 10.12688/f1000research.12232.1.

42. Buchfink B, Xie C, Huson DH. Fast and sensitive protein alignment using DIAMOND. *Nat Methods*. 2015; doi: 10.1038/nmeth.3176.

43. The UniProt Consortium. UniProt: the universal protein knowledgebase in 2021. *Nucleic Acids Research*. 2021; doi: 10.1093/nar/gkaa1100.

44. Guan D, McCarthy SA, Wood J, Howe K, Wang Y, Durbin R. Identifying and removing haplotypic duplication in primary genome assemblies. *Bioinformatics*. 2020; doi: 10.1093/bioinformatics/btaa025.

45. Meng G, Li Y, Yang C, Liu S. MitoZ: a toolkit for animal mitochondrial genome assembly, annotation and visualization. *Nucleic Acids Research*. 2019; doi: 10.1093/nar/gkz173.

46. Li H, Handsaker B, Wysoker A, Fennell T, Ruan J, Homer N, et al. The Sequence Alignment/Map format and SAMtools. *Bioinformatics*. 2009; doi: 10.1093/bioinformatics/btp352.

47. Alonge M, Lebeigle L, Kirsche M, Jenike K, Ou S, Aganezov S, et al. Automated assembly scaffolding using RagTag elevates a new tomato system for high-throughput genome editing. Genome Biology. 2022; doi: 10.1186/s13059-022-02823-7.

48. Gurevich A, Saveliev V, Vyahhi N, Tesler G. QUAST: quality assessment tool for genome assemblies. *Bioinformatics*. 2013; doi: 10.1093/bioinformatics/btt086.

49. Simão FA, Waterhouse RM, Ioannidis P, Kriventseva EV, Zdobnov EM. BUSCO: assessing genome assembly and annotation completeness with single-copy orthologs. *Bioinformatics*. 2015; doi: 10.1093/bioinformatics/btv351.

50. Mapleson D, Garcia Accinelli G, Kettleborough G, Wright J, Clavijo BJ. KAT: a K-mer analysis toolkit to quality control NGS datasets and genome assemblies. *Bioinformatics*. 2017; doi: 10.1093/bioinformatics/btw663.

51. Rhie A, Walenz BP, Koren S, Phillippy AM. Merqury: reference-free quality, completeness, and phasing assessment for genome assemblies. *Genome Biology*. 2020; doi: 10.1186/s13059-020-02134-9.

52. Wang Z, Pascual-Anaya J, Zadissa A, Li W, Niimura Y, Huang Z, et al. The draft genomes of soft-shell turtle and green sea turtle yield insights into the development and evolution of the turtle-specific body plan. *Nature Genetics*. 2013; doi: 10.1038/ng.2615.

53. Flynn JM, Hubley R, Goubert C, Rosen J, Clark AG, Feschotte C, et al.. RepeatModeler2 for automated genomic discovery of transposable element families. *Proceedings of the National Academy of Sciences*. Proceedings of the National Academy of Sciences. 2020; doi: 10.1073/pnas.1921046117.

54. Abrusán G, Grundmann N, DeMester L, Makalowski W. TEclass—a tool for automated classification of unknown eukaryotic transposable elements. *Bioinformatics*. 2009; doi: 10.1093/bioinformatics/btp084.

55. Smit AFA, Hubley R, Green P. (2022). RepeatMasker (Version 4.1.4). http://www.repeatmasker.org.

56. Leinonen R, Sugawara H, Shumway M. The Sequence Read Archive. *Nucleic Acids Res*. 2011; doi: 10.1093/nar/gkq1019.

57. Banerjee SM, Stoll JA, Allen CD, Lynch JM, Harris HS, Kenyon L, et al. Species and population specific gene expression in blood transcriptomes of marine turtles. *BMC Genomics*. 2021; doi: 10.1186/s12864-021-07656-5.

58. Chow JC, Anderson PE, Shedlock AM. Sea Turtle Population Genomic Discovery: Global and Locus-Specific Signatures of Polymorphism, Selection, and Adaptive Potential. *Genome Biology and Evolution*. 2019; doi: 10.1093/gbe/evz190.

59. Hernández-Fernández J, Pinzón Velasco AM, López Barrera EA, Rodríguez Becerra MDP, Villanueva-Cañas JL, Alba MM, et al.. De novo assembly and functional annotation of blood transcriptome of loggerhead turtle, and in silico characterization of peroxiredoxins and thioredoxins. *PeerJ*. 2021; doi: 10.7717/peerj.12395.

60. Bolger AM, Lohse M, Usadel B. Trimmomatic: a flexible trimmer for Illumina sequence data. *Bioinformatics*. 2014; doi: 10.1093/bioinformatics/btu170.

61. Dobin A, Davis CA, Schlesinger F, Drenkow J, Zaleski C, Jha S, et al.. STAR: ultrafast universal RNA-seq aligner. *Bioinformatics*. 2013; doi: 10.1093/bioinformatics/bts635.

62. Hoff KJ, Lange S, Lomsadze A, Borodovsky M, Stanke M. BRAKER1: Unsupervised RNA-Seq-Based Genome Annotation with GeneMark-ET and AUGUSTUS. *Bioinformatics*. 2016; doi: 10.1093/bioinformatics/btv661.

63. Brůna T, Hoff KJ, Lomsadze A, Stanke M, Borodovsky M. BRAKER2: automatic eukaryotic genome annotation with GeneMark-EP+ and AUGUSTUS supported by a protein database. *NAR Genomics and Bioinformatics*. 2021; doi: 10.1093/nargab/lqaa108.

64. Kriventseva EV, Kuznetsov D, Tegenfeldt F, Manni M, Dias R, Simão FA, et al. OrthoDB v10: sampling the diversity of animal, plant, fungal, protist, bacterial and viral genomes for evolutionary and functional annotations of orthologs. Nucleic Acids Research. 2019; doi: 10.1093/nar/gky1053.

65. Gabriel L, Hoff KJ, Brůna T, Borodovsky M, Stanke M. TSEBRA: transcript selector for BRAKER. *BMC Bioinformatics*. 2021; doi: 10.1186/s12859-021-04482-0.

66. Venturini L, Caim S, Kaithakottil GG, Mapleson DL, Swarbreck D. Leveraging multiple transcriptome assembly methods for improved gene structure annotation. *GigaScience*. 2018; doi: 10.1093/gigascience/giy093.

67. Grabherr MG, Haas BJ, Yassour M, Levin JZ, Thompson DA, Amit I, et al.. Trinity: reconstructing a full-length transcriptome without a genome from RNA-Seq data. *Nat Biotechnol*. 2011; doi: 10.1038/nbt.1883.

68. Wu TD, Watanabe CK. GMAP: a genomic mapping and alignment program for mRNA and EST sequences. *Bioinformatics*. 2005; doi: 10.1093/bioinformatics/bti310.

69. Mapleson D, Venturini L, Kaithakottil G, Swarbreck D. Efficient and accurate detection of splice junctions from RNA-seq with Portcullis. *GigaScience*. 2018; doi: 10.1093/gigascience/giy131.

70. Haas BJ. (2018). TransDecoder (Version 5.5.0). https://github.com/TransDecoder/TransDecoder/releases/tag/TransDecoder-v5.5.0.

71. Haas BJ, Salzberg SL, Zhu W, Pertea M, Allen JE, Orvis J, et al. Automated eukaryotic gene structure annotation using EVidenceModeler and the Program to Assemble Spliced Alignments. *Genome Biol*. 2008; doi: 10.1186/gb-2008-9-1-r7.

72. Dainat J. (2022). AGAT: Another Gff Analysis Toolkit to handle annotations in any GTF/GFF format (Version 0.9.1). *Zenodo*. 10.5281/zenodo.6488306.

73. Pertea M, Pertea GM, Antonescu CM, Chang T-C, Mendell JT, Salzberg SL. StringTie enables improved reconstruction of a transcriptome from RNA-seq reads. *Nat Biotechnol*. 2015; doi: 10.1038/nbt.3122.

74. Jones P, Binns D, Chang H-Y, Fraser M, Li W, McAnulla C, et al.. InterProScan 5: genome-scale protein function classification. *Bioinformatics*. 2014; doi: 10.1093/bioinformatics/btu031.

75. Cantarel BL, Korf I, Robb SMC, Parra G, Ross E, Moore B, et al.. MAKER: An easy-to-use annotation pipeline designed for emerging model organism genomes. *Genome Res*. 2008; doi: 10.1101/gr.6743907.

76. Klughammer J, Romanovskaia D, Nemc A, Posautz A, Seid CA, Schuster LC, et al. Comparative analysis of genome-scale, base-resolution DNA methylation profiles across 580 animal species. *Nat Commun*. 2023; doi: 10.1038/s41467-022-34828-y.

77. Oxford Nanopore Technologies. (2023). ModKit (Version 0.1.9). https://github.com/nanoporetech/modkit/releases/tag/v0.1.9.

78. Krueger F, Andrews SR. Bismark: a flexible aligner and methylation caller for Bisulfite-Seq applications. *Bioinformatics*. 2011; doi: 10.1093/bioinformatics/btr167.

79. Cristofari R. (2022). merge_CpG.py. https://github.com/rcristofari/penguin-tools/blob/master/merge_CpG.py. Accessed April 2023.

80. Akalin A, Kormaksson M, Li S, Garrett-Bakelman FE, Figueroa ME, Melnick A, et al. methylKit: a comprehensive R package for the analysis of genome-wide DNA methylation profiles. *Genome Biology*. 2012; doi: 10.1186/gb-2012-13-10-r87.

81. Wreczycka K, Gosdschan A, Yusuf D, Grüning B, Assenov Y, Akalin A. Strategies for analyzing bisulfite sequencing data. *Journal of Biotechnology*. 2017; doi: 10.1016/j.jbiotec.2017.08.007.

82. Jones PA. Functions of DNA methylation: islands, start sites, gene bodies and beyond. *Nature Reviews Genetics*. 2012; doi: 10.1038/nrg3230.

83. Akalin A, Franke V, Vlahoviček K, Mason CE, Schübeler D. genomation: a toolkit to summarize, annotate and visualize genomic intervals. *Bioinformatics*. 2015; doi: 10.1093/bioinformatics/btu775.

84. Lawrence M, Huber W, Pagès H, Aboyoun P, Carlson M, Gentleman R, et al. Software for Computing and Annotating Genomic Ranges. *PLOS Computational Biology*. Public Library of Science. 2013; doi: 10.1371/journal.pcbi.1003118.

85. Sagonas K, Meyer BS, Kaufmann J, Lenz TL, Häsler R, Eizaguirre C. Experimental Parasite Infection Causes Genome-Wide Changes in DNA Methylation. *Molecular Biology and Evolution*. 2020; doi: 10.1093/molbev/msaa084.

86. Heckwolf MJ, Meyer BS, Häsler R, Höppner MP, Eizaguirre C, Reusch TBH. Two different epigenetic information channels in wild three-spined sticklebacks are involved in salinity adaptation. *Science Advances*. American Association for the Advancement of Science. 2020; doi: 10.1126/sciadv.aaz1138.

87. R Core Team. R: A Language and Environment for Statistical Computing. Vienna, Austria: R Foundation for Statistical Computing; 2021. https://www.R-project.org/. Accessed December 2022.

88. Wickham H. ggplot2: Elegant Graphics for Data Analysis. New York, NY: Springer. 2016.

89. Li H. Minimap2: pairwise alignment for nucleotide sequences. *Bioinformatics*. 2018; doi: 10.1093/bioinformatics/bty191.

90. Cabanettes F, Klopp C. D-GENIES: dot plot large genomes in an interactive, efficient and simple way. *PeerJ*. 2018; doi: 10.7717/peerj.4958.

91. Li H, Durbin R. Inference of Human Population History From Whole Genome Sequence of A Single Individual. *Nature*. 2011; doi: 10.1038/nature10231.

92. Morin PA, Archer FI, Avila CD, Balacco JR, Bukhman YV, Chow W, et al. Reference genome and demographic history of the most endangered marine mammal, the vaquita. *Molecular Ecology Resources*. 2021; doi: 10.1111/1755-0998.13284.

93. Vilaça ST, Piccinno R, Rota-Stabelli O, Gabrielli M, Benazzo A, Matschiner M, et al.. Divergence and hybridization in sea turtles: Inferences from genome data show evidence of ancient gene flow between species. *Mol Ecol*. 2021; doi: 10.1111/mec.16113.

94. Li H. Aligning sequence reads, clone sequences and assembly contigs with BWA-MEM. *arXiv*. 2013; 10.48550/arXiv.1303.3997.

95. Picard (Version 2.26.9). https://broadinstitute.github.io/picard. Accessed January 2022.

96. Danecek P, Bonfield JK, Liddle J, Marshall J, Ohan V, Pollard MO, et al. Twelve years of SAMtools and BCFtools. *GigaScience*. 2021; doi: 10.1093/gigascience/giab008.

97. Piovano S, Clusa M, Carreras C, Giacoma C, Pascual M, Cardona L. Different growth rates between loggerhead sea turtles (Caretta caretta) of Mediterranean and Atlantic origin in the Mediterranean Sea. *Marine Biology*. 2011; doi: 10.1007/s00227-011-1759-7.

98. Hansen J, Sato M, Russell G, Kharecha P. Climate sensitivity, sea level and atmospheric carbon dioxide. *Philosophical Transactions: Mathematical, Physical and Engineering Sciences*. The Royal Society; 371:1–312013; 10.1098/rsta.2012.0294.

99. Clark PU, Shakun JD, Rosenthal Y, Köhler P, Bartlein PJ. Global and regional temperature change over the past 4.5 million years. *Science*. American Association for the Advancement of Science. 2024; doi: 10.1126/science.adi1908.

100. Robinson JA, Räikkönen J, Vucetich LM, Vucetich JA, Peterson RO, Lohmueller KE, et al. Genomic signatures of extensive inbreeding in Isle Royale wolves, a population on the threshold of extinction. *Science Advances*. American Association for the Advancement of Science; 2019; doi: 10.1126/sciadv.aau0757.

101. McKenna A, Hanna M, Banks E, Sivachenko A, Cibulskis K, Kernytsky A, et al. The Genome Analysis Toolkit: A MapReduce framework for analyzing next-generation DNA sequencing data. *Genome Res*. 2010; doi: 10.1101/gr.107524.110.

102. Martin SH. (2016). popgenWindows. https://github.com/simonhmartin/genomics_general/blob/master/popgenWindows.py. Accessed May 2022.

103. Machado CRD, Domit C, Pucci MB, Gazolla CB, Glugoski L, Nogaroto V, et al. Heterochromatin and microsatellites detection in karyotypes of four sea turtle species: Interspecific chromosomal differences. *Genet Mol Biol*. 2020. doi: 10.1590/1678-4685-GMB-2020-0213.

104. Altschul SF, Gish W, Miller W, Myers EW, Lipman DJ. Basic local alignment search tool. *Journal of Molecular Biology*. 1990; doi: 10.1016/S0022-2836(05)80360-2.

105. Gu Z, Gu L, Eils R, Schlesner M, Brors B. circlize implements and enhances circular visualization in R. *Bioinformatics*. 2014; doi: 10.1093/bioinformatics/btu393.

106. Emms DM, Kelly S. OrthoFinder: solving fundamental biases in whole genome comparisons dramatically improves orthogroup inference accuracy. *Genome Biology*. 2015; doi: 10.1186/s13059-015-0721-2.

107. Szklarczyk D, Gable AL, Nastou KC, Lyon D, Kirsch R, Pyysalo S, et al.. The STRING database in 2021: customizable protein–protein networks, and functional characterization of user-uploaded gene/measurement sets. *Nucleic Acids Res*. 2020; doi: 10.1093/nar/gkaa1074.

108. Hoff K, Lomsadze A, Borodovsky M, Stanke M. Whole-Genome Annotation with BRAKER. *Methods Mol Biol*. 2019; doi: 10.1007/978-1-4939-9173-0_5.

109. Olova N, Krueger F, Andrews S, Oxley D, et al. Comparison of whole-genome bisulfite sequencing library preparation strategies identifies sources of biases affecting DNA methylation data. *Genome Biology*. 2018; doi: 10.1186/s13059-018-1408-2.

110. Simpson JT, Workman RE, Zuzarte PC, David M, Dursi LJ, Timp W. Detecting DNA cytosine methylation using nanopore sequencing. *Nat Methods*. doi: 10.1038/nmeth.4184.

111. Xu L, Seki M. Recent advances in the detection of base modifications using the Nanopore sequencer. *Journal of Human Genetics*. 2020; doi: 10.1038/s10038-019-0679-0.

112. Shen L, Zhang Y. 5-Hydroxymethylcytosine: generation, fate, and genomic distribution. *Curr Opin Cell Biol*. 2013; doi: 10.1016/j.ceb.2013.02.017.

113. Martínez-Botí MA, Foster GL, Chalk TB, Rohling EJ, Sexton PF, Lunt DJ, et al. Plio-Pleistocene climate sensitivity evaluated using high-resolution CO2 records. *Nature*. 2015; doi: 10.1038/nature14145.

114. McClymont EL, Ho SL, Ford HL, Bailey I, Berke MA, Bolton CT, et al. Climate Evolution Through the Onset and Intensification of Northern Hemisphere Glaciation. *Reviews of Geophysics*. 2023; doi: 10.1029/2022RG000793.

115. Ford HL, Chalk TB. SIDEBAR. The Mid-Pleistocene Enigma. *Oceanography*. 2020; doi: 10.5670/oceanog.2020.216.

116. Lawrence KT, Sigman DM, Herbert TD, Riihimaki CA, Bolton CT, Martinez-Garcia A, et al. Time-transgressive North Atlantic productivity changes upon Northern Hemisphere glaciation. *Paleoceanography*. 2013; doi: 10.1002/2013PA002546.

117. Lamy F, Winckler G, Arz HW, Farmer JR, Gottschalk J, Lembke-Jene L, et al. Five million years of Antarctic Circumpolar Current strength variability. *Nature*. 2024; doi: 10.1038/s41586-024-07143-3.

118. McQueen HA, Fantes J, Cross SH, Clark VH, Archibald AL, Bird AP. CpG islands of chicken are concentrated on microchromosomes. *Nat Genet*. 1996; doi: 10.1038/ng0396-321.

119. Waters PD, Patel HR, Ruiz-Herrera A, Álvarez-González L, Lister NC, Simakov O, et al. Microchromosomes are building blocks of bird, reptile, and mammal chromosomes. *Proceedings of the National Academy of Sciences*. 2021; doi: 10.1073/pnas.2112494118.

120. Papin C, Le Gras S, Ibrahim A, Salem H, Karimi MM, Stoll I, et al. CpG Islands Shape the Epigenome Landscape. *Journal of Molecular Biology*. 2021; doi: 10.1016/j.jmb.2020.09.018.

121. Hoelzel AR, Bruford MW, Fleischer RC. Conservation of adaptive potential and functional diversity. *Conserv Genet*. 2019; doi: 10.1007/s10592-019-01151-x.

122. Harewood L, Fraser P. The impact of chromosomal rearrangements on regulation of gene expression. *Human Molecular Genetics*. 2014; doi: 10.1093/hmg/ddu278.

123. Valenzuela N, Adams DC. CHROMOSOME NUMBER AND SEX DETERMINATION COEVOLVE IN TURTLES. *Evolution*. 2011; doi: 10.1111/j.1558-5646.2011.01258.x.

124. Lee L, Montiel EE, Navarro-Domínguez BM, Valenzuela N. Chromosomal Rearrangements during Turtle Evolution Altered the Synteny of Genes Involved in Vertebrate Sex Determination. *Cytogenet Genome Res*. 2019; doi: 10.1159/000497302.

125. Schroeder AL, Metzger KJ, Miller A, Rhen T. A Novel Candidate Gene for Temperature-Dependent Sex Determination in the Common Snapping Turtle. *Genetics*. 2016; doi: 10.1534/genetics.115.182840.

126. Elango N, Yi SV. DNA Methylation and Structural and Functional Bimodality of Vertebrate Promoters. *Molecular Biology and Evolution*. 2008; doi: 10.1093/molbev/msn110.

127. Keller TE, Han P, Yi SV. Evolutionary Transition of Promoter and Gene Body DNA Methylation across Invertebrate–Vertebrate Boundary. *Mol Biol Evol*. 2016; doi: 10.1093/molbev/msv345.

128. De Paoli-Iseppi R, Deagle BE, McMahon CR, Hindell MA, Dickinson JL, Jarman SN. Measuring Animal Age with DNA Methylation: From Humans to Wild Animals. *Front Genet*. Frontiers. 2017; doi: 10.3389/fgene.2017.00106.

129. Viitaniemi HM, Verhagen I, Visser ME, Honkela A, van Oers K, Husby A. Seasonal Variation in Genome-Wide DNA Methylation Patterns and the Onset of Seasonal Timing of Reproduction in Great Tits. *Genome Biology and Evolution*. 2019; doi: 10.1093/gbe/evz044.

130. Grant OA, Wang Y, Kumari M, Zabet NR, Schalkwyk L. Characterising sex differences of autosomal DNA methylation in whole blood using the Illumina EPIC array. *Clinical Epigenetics*. 2022; doi: 10.1186/s13148-022-01279-7.

131. Yousefi PD, Suderman M, Langdon R, Whitehurst O, Davey Smith G, Relton CL. DNA methylation-based predictors of health: applications and statistical considerations. *Nat Rev Genet*. 2022; doi: 10.1038/s41576-022-00465-w.

132. Xu R, Li S, Guo S, Zhao Q, Abramson MJ, Li S, et al. Environmental temperature and human epigenetic modifications: A systematic review. *Environmental Pollution*. 2020; doi: 10.1016/j.envpol.2019.113840.

133. Mäkinen H, Van Oers K, Eeva T, Ruuskanen S. The effect of experimental lead pollution on DNA methylation in a wild bird population. *Epigenetics*. 2022. doi: 10.1080/15592294.2021.1943863.

134. Mayne B, Mustin W, Baboolal V, Casella F, Ballorain K, Barret M, et al. Differential methylation between sex in adult green sea turtle skin biopsies. *Frontiers in Marine Science*. 2021; doi: 10.3389/fmars.2023.1169808.

135. Yen EC, Gilbert JD, Balard A, Afonso IO, Fairweather K, Newlands D, et al. DNA methylation carries signatures of sublethal effects under thermal stress in loggerhead sea turtles. *bioRxiv*. 2023; doi: 10.1101/2023.11.22.568239.

136. Mork L, Capel B. Conserved action of β-catenin during female fate determination in the red-eared slider turtle. *Evolution & Development*. 2013; doi: 10.1111/ede.12020.

137. Liu J, Xiao Q, Xiao J, Niu C, Li Y, Zhang X, et al.. Wnt/β-catenin signalling: function, biological mechanisms, and therapeutic opportunities. *Sig Transduct Target Ther*. 2022; doi: 10.1038/s41392-021-00762-6.

138. Tezak B, Straková B, Fullard DJ, Dupont S, McKey J, Weber C, et al.. Higher temperatures directly increase germ cell number, promoting feminization of red-eared slider turtles. *Current Biology*. 2023; doi: 10.1016/j.cub.2023.06.008.

139. Rhen T, Schroeder A. Molecular Mechanisms of Sex Determination in Reptiles. *SXD*. Karger Publishers. 2010; doi: 10.1159/000282495.

140. Kent J, Wheatley SC, Andrews JE, Sinclair AH, Koopman P. A male-specific role for SOX9 in vertebrate sex determination. *Development*. 1996; doi: 10.1242/dev.122.9.2813.

141. Moreno-Mendoza N, Harley VR, Merchant-Larios H. Temperature Regulates SOX9 Expression in Cultured Gonads of *Lepidochelys olivacea*, a Species with Temperature Sex Determination. *Developmental Biology*. 2001; doi: 10.1006/dbio.2000.9952.

142. Kohno S, Katsu Y, Urushitani H, Ohta Y, Iguchi T, Guillette Jr LJ. Potential Contributions of Heat Shock Proteins to Temperature-Dependent Sex Determination in the American Alligator. *Sexual Development*. 2009; doi: 10.1159/000260374.

143. Yen EC. (2024). Article_CarCar_GenomeAssembly. https://github.com/eugeniecyen/Article_CarCar_GenomeAssembly.

Figure 1

Click here to access/download;Figure;Fig1.png

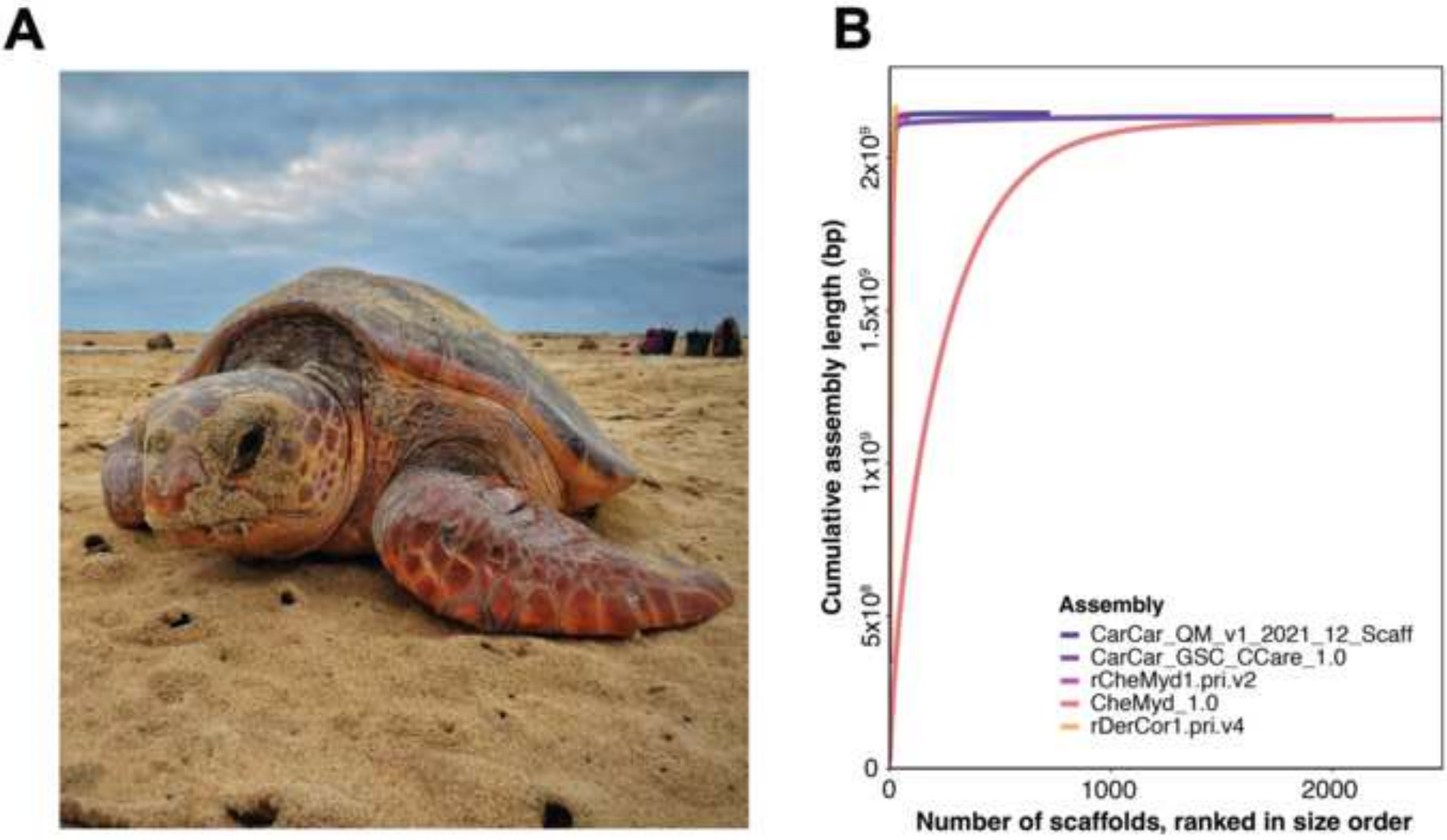

Figure 2

Click here to access/download;Figure;Fig2.png

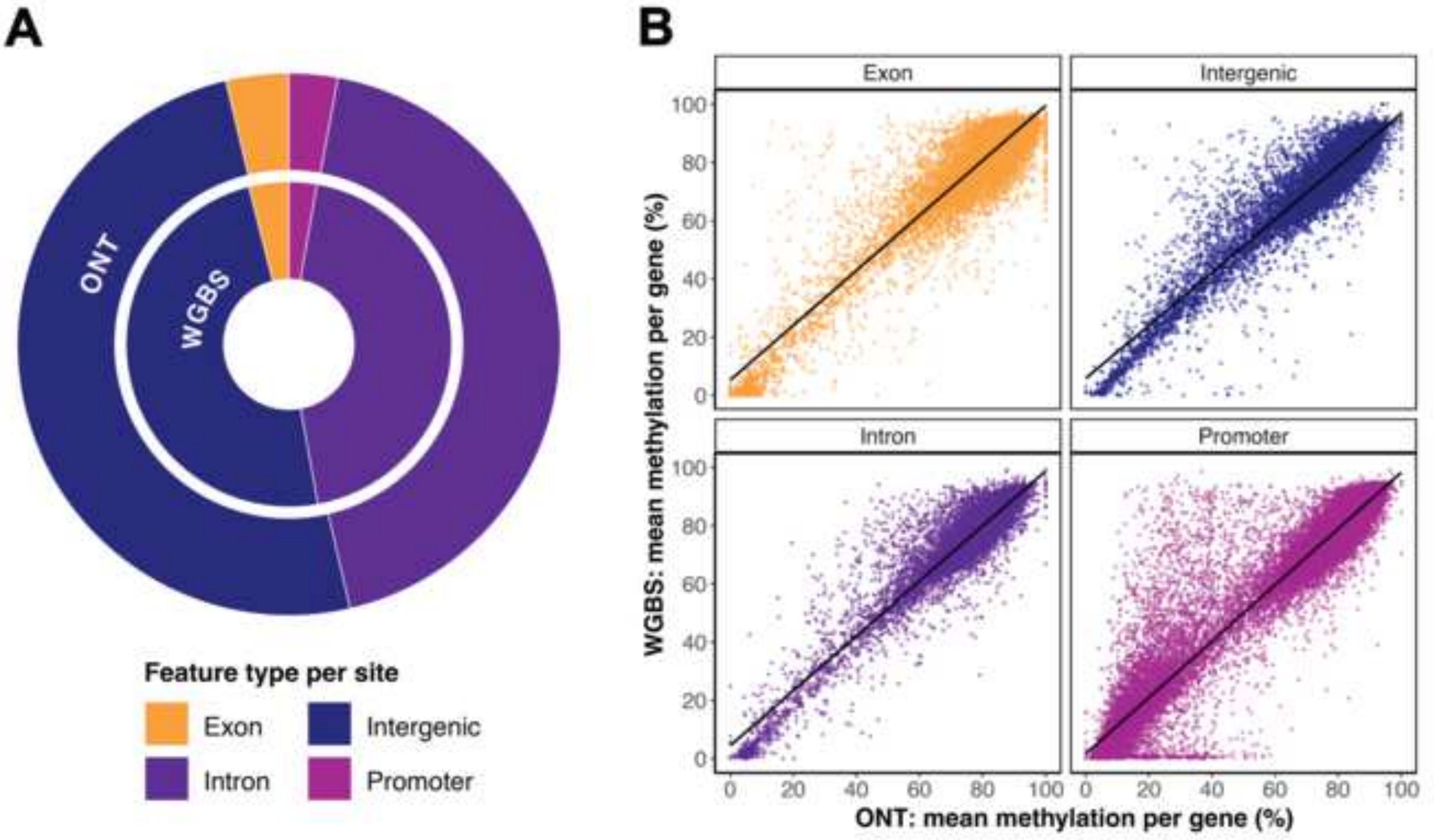

Figure 3

Click here to access/download;Figure;Fig3.png

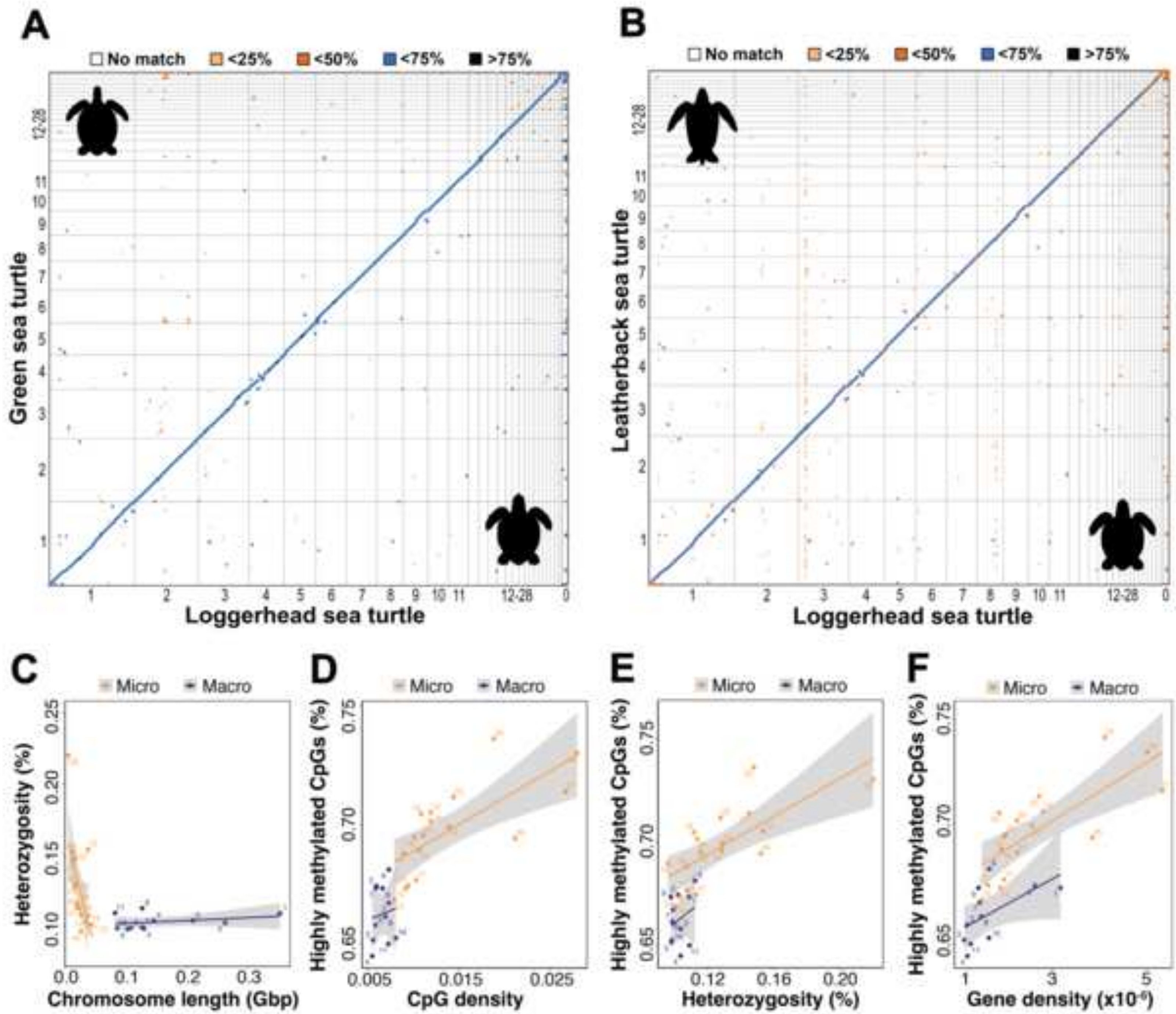

Figure 4

Click here to access/download;Figure;Fig4.png

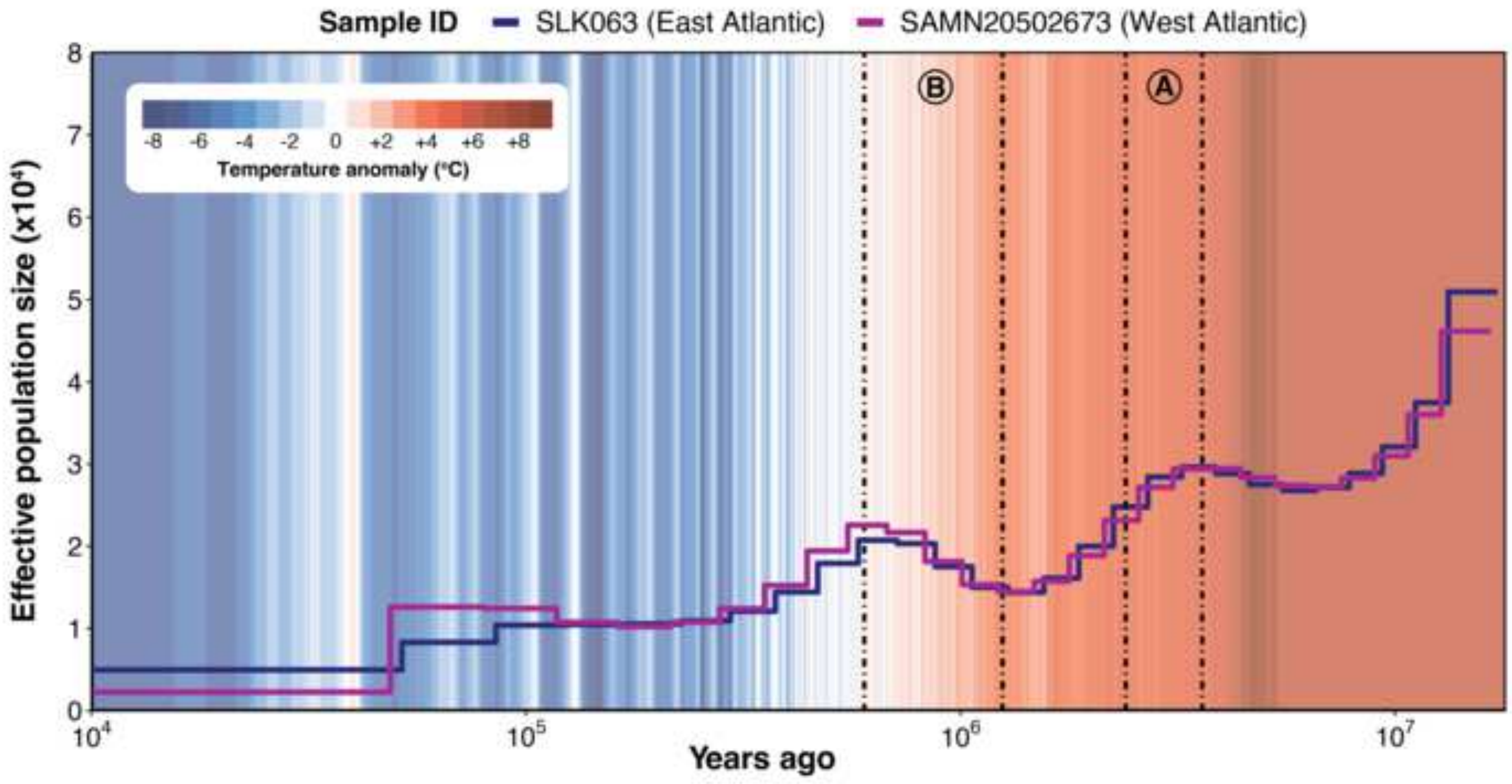

Figure 5

Click here to access/download;Figure;Fig5.png

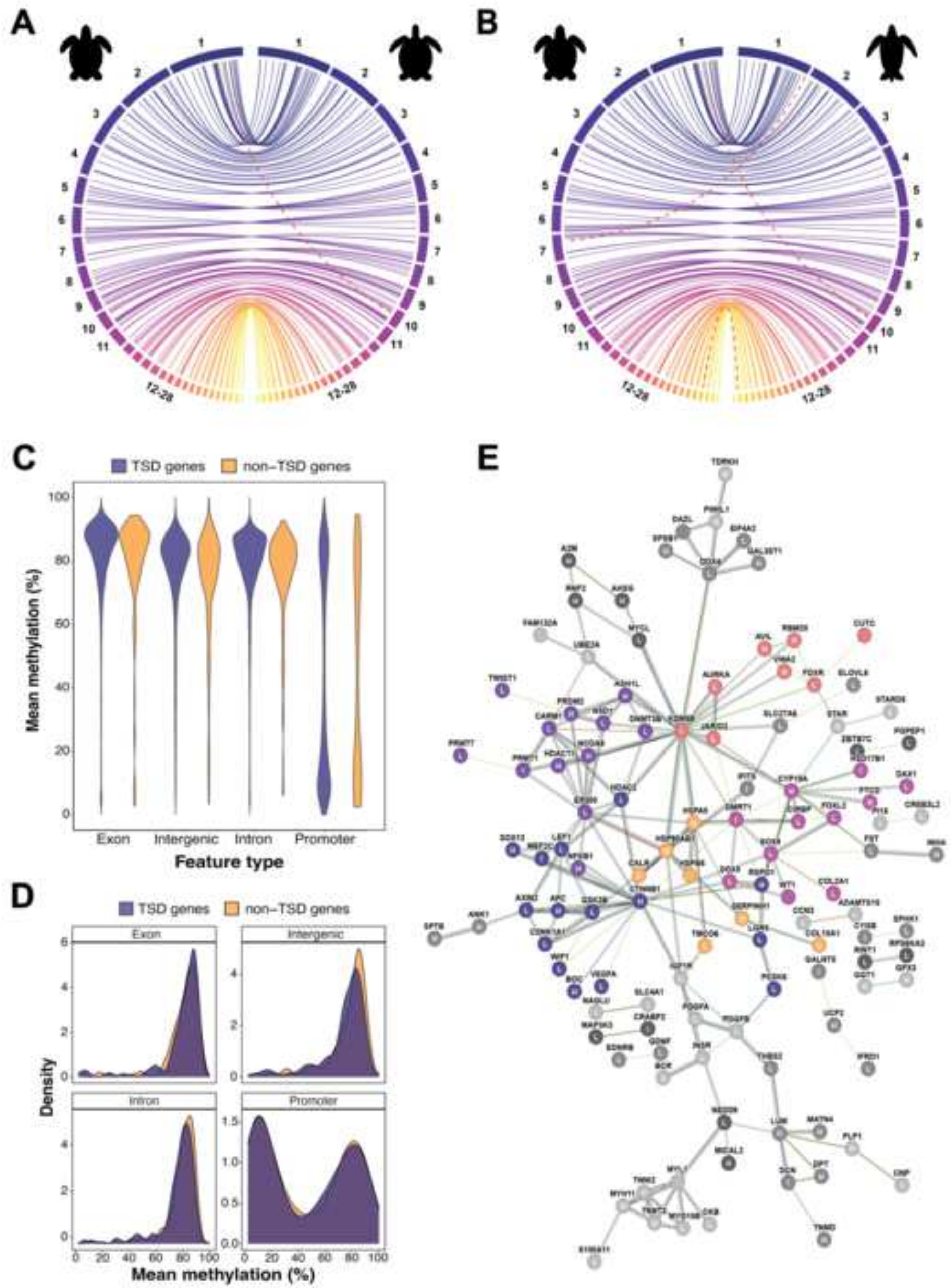

Click here to access/download
Supplementary Material
Yen2024_Supplementary_Material.docx

Cover Letter

Click here to access/download;Personal Cover;Yen2024_Cover_Letter_3000c.pdf

Ms Eugenie C. Yen
School of Biological and Chemical Sciences
Queen Mary University of London
Fogg Building, Mile End Road,
London, E1 4NS, UK
e.yen@qmul.ac.uk

Date: 9th September 2024

Dear Editor,

We hereby submit our manuscript "**Chromosome-level genome assembly and methylome profile yield insights for the conservation of endangered loggerhead sea turtles**" for consideration as a GigaScience Data Note.

Characterising genetic and epigenetic diversity is urgently needed to assess the adaptive potential of populations/species under global warming. This is a major goal of the field of conservation genomics, yet it is not always clear how genetic resources can guide conservation efforts. Here, we focus on sea turtles as endangered flagship species. They are especially climate-vulnerable because they have temperature-dependent sex determination (TSD), with complete feminisation and demographic collapse predicted by 2100. Using Oxford Nanopore Technology (ONT) and Illumina sequencing, we present a chromosome-level reference genome and annotation for a loggerhead sea turtle (Caretta caretta, IUCN: Vulnerable Species) from the East Atlantic rookery. This is the world's largest loggerhead nesting aggregation, yet it remains heavily threatened (IUCN: Endangered Population). Our assembly provides the first loggerhead reference genome for this globally important population, and the second, most complete assembly for loggerheads. Given strong genetic structure among loggerhead populations driven by philopatry, our assembly will improve genomic studies by mitigating reference bias for this rookery, and add an East Atlantic loggerhead genome for comparative studies. To our best knowledge, we also offer the first ONT-derived methylome for a sea turtle species, verified by comparison to whole genome bisulfite sequencing of ten loggerheads from the same population.

To demonstrate their potential, we applied our resources to yield insights relevant for sea turtle conservation. We related demographic fluctuations to climate change. We highlighted microchromosomes as regions of high genetic diversity and epigenetic potential. We finally focused on 191 TSD-linked genes, building the largest map of functional associations and methylation status for sea turtles to date.

Overall, we showcase the power of ONT sequencing for generating genomic and epigenomic data simultaneously, to drive genome resource creation and molecular insights. This dual approach is important for sea turtles and other TSD species, which possess a tight plastic link to the environment. Our chromosome-level genome assembly, annotation, and methylome for loggerhead turtles will be reused to perform genomic/epigenomic studies to conserve these vulnerable charismatic species.

All sequencing data are available via ENA study PRJEB79015. Genome assemblies, annotations and other data files are being uploaded, and can be made available to reviewers. All scripts will be uploaded to GitHub. We declare no competing interests. All authors approved this submission, which has not been submitted for publication elsewhere.

Thank you for your consideration.

Sincerely,

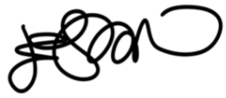

Eugenie C. Yen, on behalf of all authors
