# Supplementary material for: Chromosome-level genome assembly and methylome profile yield insights for the conservation of endangered loggerhead sea turtles
Source: Gigascience. 2025 Jun 6;14:giaf054. doi: 10.1093/gigascience/giaf054 (PMC12143204; doi:10.1093/gigascience/giaf054)
Supplement: giaf054_GIGA-D-24-00386_Revision_1 [file giaf054_giga-d-24-00386_revision_1.pdf]

GigaScience

Chromosome-level genome assembly and methylome profile yield insights for the conservation of endangered loggerhead sea turtles

--Manuscript Draft--

**Manuscript Number:**	GIGA-D-24-00386R1
**Full Title:**	Chromosome-level genome assembly and methylome profile yield insights for the conservation of endangered loggerhead sea turtles
**Article Type:**	Data Note
**Funding Information:**	Natural Environment Research Council (NE/V001469/1) — Prof Christophe Eizaguirre<br>Natural Environment Research Council (NE/X012077/1) — Prof Christophe Eizaguirre<br>National Geographic Society (NGS-59158R-19) — Prof Christophe Eizaguirre<br>Natural Environment Research Council (NE/S007229/1) — Miss Eugenie C Yen
**Abstract:**	Background: Characterising genetic and epigenetic diversity is crucial for assessing the adaptive potential of threatened populations and species in the face of climate change. Sea turtles are particularly vulnerable due to their temperature-dependent sex determination (TSD) system, which heightens the risk of extreme sex ratio bias and extinction under future climate scenarios. High-quality genomic and epigenomic resources will therefore support conservation efforts for these endangered flagship species with such plastic traits.<br>Findings: We generated a chromosome-level genome assembly for the loggerhead sea turtle (Caretta caretta) from the globally important Cabo Verde rookery. Using Oxford Nanopore Technology (ONT) and Illumina reads followed by homology-guided scaffolding to the same species, we achieved a contiguous (N50: 129.7 Mbp) and complete (BUSCO: 97.1%) assembly, with 98.9% of the genome scaffolded into 28 chromosomes and 33,887 annotated genes. We also extracted the blood methylome profile from our ONT reads, which was confirmed to be representative of the reference population via whole genome bisulfite sequencing of ten additional loggerheads from the same population. Applying our novel resources, we revealed high conservation of synteny between sea turtle species, reconstructed population size fluctuations in line with major climatic events, and identified microchromosomes as key regions for monitoring genetic diversity and epigenetic flexibility. Isolating 199 TSD-linked genes, we further built a large network of functional protein associations and blood-based methylation patterns.<br>Conclusions: We present a high-quality loggerhead sea turtle genome and methylome from the globally significant East Atlantic population. By leveraging ONT sequencing, we generate genomic and epigenomic resources simultaneously, and showcase the potential of this approach for driving molecular insights for conservation of endangered sea turtles.
**Corresponding Author:**	Eugenie C Yen, BA (Hons), MPhil<br>Queen Mary University of London<br>London, UNITED KINGDOM
**Corresponding Author Secondary Information:**	
**Corresponding Author's Institution:**	Queen Mary University of London
**Corresponding Author's Secondary Institution:**	
**First Author:**	Eugenie C Yen, BA (Hons), MPhil
**First Author Secondary Information:**	
**Order of Authors:**	Eugenie C Yen, BA (Hons), MPhil<br>James D Gilbert

Powered by Editorial Manager® and ProduXion Manager® from Aries Systems Corporation

	Alice Balard
	Albert Taxonera
	Kirsten Fairweather
	Heather L Ford
	Doko-Miles J Thorburn
	Stephen J Rossiter
	José M Martín-Durán
	Christophe Eizaguirre
**Order of Authors Secondary Information:**	
**Response to Reviewers:**	Dear Editors,<br><br>Thank you for providing us with the opportunity to submit a revised version of our manuscript GIGA-D-24-00386 “Chromosome-level genome assembly and methylome profile yield insights for the conservation of endangered loggerhead sea turtles for publication as a Data Note in GigaScience. We thank the Editorial Team and reviewers for their positive assessment of our manuscript.<br><br>We have taken all their comments onboard and addressed them thoroughly in our point-by-point response provided in blue in the document "Yen2024_Genome_Response_To_Reviewers_Rev1"<br><br>All authors approved this submission. Please, let me know if you require any additional information.<br><br>Best regards,<br>Eugenie C. Yen (on behalf of all authors)
**Additional Information:**	
**Question**	**Response**
Are you submitting this manuscript to a special series or article collection?	No
**Experimental design and statistics**<br><br>Full details of the experimental design and statistical methods used should be given in the Methods section, as detailed in our Minimum Standards Reporting Checklist. Information essential to interpreting the data presented should be made available in the figure legends.<br><br>Have you included all the information requested in your manuscript?	Yes
**Resources**<br><br>A description of all resources used, including antibodies, cell lines, animals	Yes

and software tools, with enough information to allow them to be uniquely identified, should be included in the Methods section. Authors are strongly encouraged to cite Research Resource Identifiers (RRIDs) for antibodies, model organisms and tools, where possible.<br><br>Have you included the information requested as detailed in our Minimum Standards Reporting Checklist?	
**Availability of data and materials**<br><br>All datasets and code on which the conclusions of the paper rely must be either included in your submission or deposited in publicly available repositories (where available and ethically appropriate), referencing such data using a unique identifier in the references and in the "Availability of Data and Materials" section of your manuscript.<br><br>Have you have met the above requirement as detailed in our Minimum Standards Reporting Checklist?	No
If not, please give reasons for any omissions below.<br><br>as follow-up to "**Availability of data and materials**<br><br>All datasets and code on which the conclusions of the paper rely must be either included in your submission or deposited in publicly available repositories (where available and ethically appropriate), referencing such data using a unique identifier in the references and in the "Availability of Data and Materials" section of your manuscript.<br><br>Have you have met the above	All sequencing data is available on ENA via study accession PRJEB79015. Genome assemblies, annotations and other data files are in the process of being uploaded to ENA, and thus are not publicly available yet, but will be made available to reviewers on GigaDB.

requirement as detailed in our Minimum Standards Reporting Checklist?<br><br>"	

main text Click here to access/download;Manuscript;Yen2024_Genome_Manuscript_

Chromosome-level genome assembly and methylome profile yield insights for the conservation of endangered loggerhead sea turtles

Authors

Eugenie C. Yen[1*], James D. Gilbert[1], Alice Balard[1], Albert Taxonera[2], Kirsten Fairweather[2], Heather L. Ford[3], Doko-Miles J. Thorburn[1], Stephen J. Rossiter[1], José M. Martín-Durán[1], Christophe Eizaguirre[1]

Affiliations

[1] School of Biological and Behavioural Sciences, Queen Mary University of London, London, E1 4DQ, UK

[2] Project Biodiversity, Mercado Municipal, local 22 Santa Maria, Ilha do Sal, Cabo Verde

[3] School of Geography, Queen Mary University of London, London, E1 4NS, UK

Emails

Eugenie C. Yen: e.yen@qmul.ac.uk (* Corresponding Author)

James D. Gilbert: j.gilbert@qmul.ac.uk

Alice Balard: alice.cam.balard@gmail.com

Albert Taxonera: albert.taxo@projectbiodiversity.org

Kirsten Fairweather: seaturtles@projectbiodiversity.org

Heather L. Ford: h.ford@qmul.ac.uk

Doko-Miles J. Thorburn: miles.thorburn@gmail.com

Stephen J. Rossiter: s.j.rossiter@qmul.ac.uk

José M. Martín-Durán: chema.martin@qmul.ac.uk

Christophe Eizaguirre: c.eizaguirre@qmul.ac.uk

Eugenie C Yen [0000-0003-4992-782X]; James D Gilbert [0000-0003-2808-2004]; Alice Balard [0000-0002-0942-7479]; Heather L Ford [0000-0002-8081-7023]; Doko-Miles J Thorburn [0000-0002-0120-8829]; Stephen J Rossiter [0000-0002-3881-4515]; José M Martín-Durán [0000-0002-2572-1061]; Christophe Eizaguirre [0000-0002-8673-7649]

ABSTRACT

Background: Characterising genetic and epigenetic diversity is crucial for assessing the adaptive potential of threatened populations and species in the face of climate change. Sea turtles are particularly vulnerable due to their temperature-dependent sex determination (TSD) system, which heightens the risk of extreme sex ratio bias and extinction under future climate scenarios. High-quality genomic and epigenomic resources will therefore support conservation efforts for these endangered flagship species with such plastic traits.

Findings: We generated a chromosome-level genome assembly for the loggerhead sea turtle (*Caretta caretta*) from the globally important Cabo Verde rookery. Using Oxford Nanopore Technology (ONT) and Illumina reads followed by homology-guided scaffolding to the same species, we achieved a contiguous (N50: 129.7 Mbp) and complete (BUSCO: 97.1%) assembly, with 98.9% of the genome scaffolded into 28 chromosomes and 33,887 annotated genes. We also extracted the blood methylome profile from our ONT reads, which was confirmed to be representative of the reference population via whole genome bisulfite sequencing of ten additional loggerheads from the same population. Applying our novel resources, we revealed high conservation of synteny between sea turtle species, reconstructed population size fluctuations in line with major climatic events, and identified microchromosomes as key regions for monitoring genetic diversity and epigenetic flexibility. Isolating 199 TSD-linked genes, we further built a large network of functional protein associations and blood-based methylation patterns.

Conclusions: We present a high-quality loggerhead sea turtle genome and methylome from the globally significant East Atlantic population. By leveraging ONT sequencing, we generate genomic and epigenomic resources simultaneously, and showcase the potential of this approach for driving molecular insights for conservation of endangered sea turtles.

Keywords: reference genome, loggerhead sea turtle, endangered species, epigenomics, DNA methylation, temperature-dependent sex determination, Oxford Nanopore Technology

BACKGROUND

With biodiversity declining at an alarming rate [1], genomic tools are increasingly being deployed to inform conservation management strategies for endangered species [2]. Characterising genetic diversity [3, 4], inbreeding [5], demographic history [6], and locally adapted genomic regions [7] can all provide insights into the adaptive potential of populations and species [8]. Conservation epigenomics has recently gained momentum, driven by technological advancements and falling sequencing costs [9]. This is a promising framework as it offers an additional layer of molecular information that can directly link individuals to their environment [10]. For instance, quantifying epigenetic variation can aid in assessing a population's capacity for adaptive plastic responses, or provide biomarkers that reflect individual health and environmental exposure [9, 11, 12]. In particular, DNA methylation—the addition of a methyl group to cytosine residues to regulate gene expression—is the best-described epigenetic modification in non-model species to date [13].

There are seven species of sea turtles, with six classified as Vulnerable to Critically Endangered and one as Data Deficient by the IUCN Red List [14]. Beyond threats such as bycatch, poaching, and coastal development [15], sea turtles are climate-vulnerable because of their

poikilothermic physiology and temperature-dependent sex determination (TSD) system, where higher incubation temperatures induce female development [16]. As theoretical studies predict a significant shift towards female-biased primary sex ratios and subsequent population collapse by 2100 [17, 18], it is essential to assess whether sea turtles can sustain viable sex ratios via adaptive responses [19, 20]. Although their capacity for genetic evolution is constrained by long generation times and small effective population sizes [21], plastic responses via epigenetic mechanisms could offer alternative pathways, especially as they already play a role in TSD regulation [22, 23, 24]. A lack of high-quality reference genomes previously hindered such molecular insights in sea turtles. However, this situation is changing, with chromosome-level genome assemblies now released for the green sea turtle (*Chelonia mydas,* NCBI TaxID: 8469*)* [25], leatherback sea turtle (*Dermochelys coriacea,* NCBI TaxID: 27794) [25], hawksbill sea turtle (*Eretmochelys imbricata,* NCBI TaxID: 27787) [26], and a loggerhead sea turtle (*Caretta caretta,* NCBI TaxID: 8467) [27]. The latter was sequenced by the Canada BioGenome Project (CBP) and represents individuals from the Adriatic Sea. Yet, given the well-known influence of reference bias on downstream population-based analyses, continuing to build genome resources remains crucial [28].

Here, we present a chromosome-level assembly for a loggerhead sea turtle from the Cabo Verde (East Atlantic) nesting aggregation (**Figure 1A**, Population IUCN Red List Status: Endangered), which is now the largest worldwide for this species [29]. The population is composed of genetically distinct nesting groups maintained by strong female philopatry across the archipelago [30]. Our population-specific assembly complements the existing loggerhead turtle genome ‘GSC_CCare_1.0’ [27] by eliminating reference bias for genomic studies of the globally important East Atlantic rookery, while providing a high-quality loggerhead genome for comparative studies. In addition, we present the first methylome profile derived from

Oxford Nanopore Technology (ONT) reads for a sea turtle species, which we compared against methylomes of ten loggerheads from the same population obtained via whole genome bisulfite sequencing (WGBS). We next applied our novel resources to describe genome-wide synteny, demographic history, and genomic properties of our target genome. Lastly, we described the chromosomal locations of 199 TSD-linked genes, then created a map of their methylation status and predicted functional associations, providing a useful resource for future epigenetic studies of these endangered TSD species.

METHODS

Reference sample collection

On the 31st of August 2020, we sampled blood from a wild female loggerhead turtle (ID: SLK063, Permit: 013/DNA/2020) that nested on Sal Island of the Cabo Verde Archipelago. The nesting season extends between late June and October at this rookery. The sampling site (16.62123, -22.92972) was on Algodoeiro Beach, which consists of 800 m of sandy coastline. Blood was collected from the dorsal cervical sinus with a 40 mm, 21-gauge needle and 5 ml syringe following oviposition [31]. A Passive Integrated Transponder tag was added to the front right flipper for identification [30]. The sample was stored in a lithium heparin tube then centrifuged for 1 min at 3000 rpm to separate plasma and blood cells. Samples were stored at -18°C during the field season, then at -80°C following transport to Queen Mary University of London (London, UK).

DNA extraction, sequencing and quality control

Genomic DNA was extracted from the nucleated blood cells using a QIAGEN Genomic-Tips 100G Kit (Qiagen, Germany). For ONT sequencing, libraries were constructed using an SQK-LSK109 Ligation Sequencing Kit and sequencing was conducted on the PromethION 24

platform with a FLO-PRO002 (R9.4.1 chemistry) flow cell (Oxford Nanopore Technologies, UK) with a benchmarked sequencing error rate of ~7.16% [32]. Base calling was performed via Guppy v.4.0.11 in high-accuracy mode [33]. This generated 11,643,721 reads (50,075,750,398 bp, ~23.3X sequencing depth) with an N50 of 8,230 bp (base pairs). All downstream bioinformatic steps were conducted on the Apocrita High Performance Computing Cluster [34]. Adapters were trimmed with PoreChop v.0.2.4 [35] and reads were filtered for a Phred score >Q8 [36] and length >500 bp with NanoFilt v.2.6.0 [37]. This passed 8,288,359 reads (40,371,088,741 bp, 80.6%, ~18.8X sequencing depth), with an N50 of 8518 bp.

We also generated Illumina sequencing data for assembly polishing. Libraries were constructed by fragmenting DNA via sonification, end polishing, A-tailing and adapter ligation, polymerase chain reaction (PCR) amplification with P5 and indexed P7 oligos, and purification with the AMPure XP system. Sequencing was performed with 150 bp paired end reads on the NovaSeq 6000 platform (Illumina, USA) (RRID:SCR_016387). This gave 1,050,248,476 reads (157,537,271,400 bp, ~73.3X sequencing depth). Haploid genome size, heterozygosity, and repeat content were estimated via GenomeScope (RRID:SCR_017014) (**Figure S1)** [38]. Reads were trimmed for adapters and filtered for a Phred score >Q20 with TrimGalore v.0.6.5 (RRID:SCR_011847) [39], passing 1,050,248,476 reads (156,382,436,354 bp, 99.3%,~72.9X sequencing depth).

De novo assembly

ONT reads were assembled using Flye v.2.8.3 (RRID:SCR_017016) [40] in '--asm-coverage 40' mode. A polished consensus sequence was produced using Medaka v.1.3.3 with the 'r941_prom_high_g4011' model [41]. Using our Illumina reads from the same sample, two rounds of error polishing were performed with Pilon v.1.24 [42]. The contamination level was

assessed via BlobTools v.1.1.1 [43] with Diamond BLASTx v.2.0.11(RRID:SCR_001653) [44], comparing against the 2021_03 release of the UniProt reference proteomes database [45]. The assembly was haploidised using Purge_Dups v.1.2.5 [46] to give a contig-level assembly 'CarCar_QM_v1.21.12'. In addition, we assembled and annotated the mitochondrial genome 'CarCar_QM_v1.21.12'_Mito' from our Illumina data with MitoZ v.3.4 (see **Text S1** for extended methods) [47].

Homology-guided scaffolding

We used the chromosome-level Adriatic loggerhead turtle assembly 'GSC_CCare_1.0' produced by the CBP for homology-guided scaffolding [27]. Unplaced contigs were removed with SAMtools v.1.9 (RRID:SCR_002105) [48], then the remaining 28 chromosomal scaffolds served as a reference for homology-based scaffolding into chromosomes using RagTag v.2.1.0 [49]. We supply two versions of our assembly: (1) 'CarCar_QM_v1.21.12_Sc' with 28 chromosomes and unplaced contigs/scaffolds, and (2) 'CarCar_QM_v1.21.12_Sc_Chr0' where unplaced contigs/scaffolds were concatenated into a single scaffold 'SLK063_ragtag_chr0' with 100 bp of Ns as gap padding. Note this artificial chromosome was created as an option to facilitate certain computational analyses by reducing assembly fragmentation and does not represent true positional information.

Assembly quality assessment

Contiguity was evaluated with QUAST v.5.0.2 (RRID:SCR_001228) [50]. Completeness was assessed with Benchmarking Universal Single-Copy Ortholog (BUSCO) scores, using BUSCO v.5.1.2 (RRID:SCR_015008) [51] in genome mode against the 'sauropsida_odb10' database (n=7480 BUSCOs). K-mer-based assessments were performed by comparing k-mers from our Illumina reads against the assembly with parameter K=21. A k-mer spectrum was produced

using KAT v.2.4.1 [52] and an assembly consensus quality value (QV) was calculated with Merqury v.1.3 [53]. Contiguity and completeness comparisons were also conducted against all assemblies currently available for sea turtles: (1) 'CarCar_GSC_CCare_1.0' produced by the CBP for the Adriatic loggerhead turtle [27], (2) 'rDerCor1.pri.v4' for the leatherback turtle and (3) 'rCheMyd1.pri.v2' for the green turtle, both produced by the Vertebrate Genomes Project (VGP) [25], (4) 'ASM3001250v1' produced by an independent group for the hawksbill turtle [26], and (5) 'CheMyd_1.0' which was the first draft assembly for the green turtle [54].

Genome annotation

We performed genome annotation for our chromosome-level assembly 'CarCar_QM_v1.21.12_Sc'. A repeat library was built using RepeatModeler v.2.0.4 (RRID:SCR_015027) in 'LTRStruct' mode to discover long terminal repeats [55]. To exclude potential gene families, the repeat library was compared against the proteome of the 'rCheMyd1.pri.v2' green turtle annotation [25] via Diamond BLASTp v.2.0.11 (RRID:SCR_001010) [44]. Transposable elements (TEs) were classified using TEclass [56], then the curated repeat library was used for annotation via RepeatMasker v.4.1.4 (RRID:SCR_012954) [57].

For gene annotation, paired-end RNA-Seq reads (n=746,132,735, **Table S1**) from 24 loggerhead turtles across three life stages (hatchling, juvenile and adult), four tissue types (blood, gonad, brain and heart) and both sexes were mined from the Sequence Read Archive [58, 59, 60, 61]. Reads were trimmed with Trimmomatic v.0.36 (RRID:SCR_011848) [62], mapped with STAR v.2.7.10a [63], and sorted with SAMtools v.1.9 [48]. Alignments were then supplied to BRAKER1 for gene prediction [64]. Note that species-specific training was attempted, but it resulted in a poorer annotation. Pre-trained parameters for the chicken (*Gallus*

gallus domesticus) were hence applied, as the most related species available. Concurrently, gene prediction followed the Mikado pipeline v.2.2.4 [65] with whole transcriptome and transcript-based hints. Transcriptomes included a publicly available blood transcriptome produced from eight loggerhead turtles across three life stages [61], and a second transcriptome we assembled *de novo* using Trinity v.2.14 [66] with publicly available RNA-Seq data (**Table S1**) from gonad, brain, and heart tissue of three hatchlings [60]. Transcriptomes were mapped to our assembly via GMAP v.2021-12-17 [67], with a 99.56% and 99.98% alignment rate, respectively. From our STAR alignments, intron junctions were curated with Portcullis v.1.2.3 [68] and open reading frames were calculated using TransDecoder v.5.5.0 [69]. All evidence types were subsequently supplied for gene prediction by Mikado.

The BRAKER and Mikado gene sets were merged using the PASA pipeline v.2.5.2 (RRID:SCR_014656) [70] with three rounds of comparison. Finally, the gene set was filtered and standardised with AGAT v.0.9.1 [71], and in-frame stop codons were removed with gffread v.0.12.7 in '-V -H' mode [72]. To assess completeness, BUSCO v.5.1.2 [51] was run on the longest isoforms in protein mode against the 'sauropsida_odb10' database (n=7480 BUSCOs). Homology-based functional information was assigned via Diamond BLASTp v.2.0.11 [44] against the SwissProt database v.2022_03_02 [45]. Gene Ontology (GO) terms were added using InterProScan 5 v.5.60-92.0 with HMMER databases: Gene3D-4.3.0, PANTHER-17.0, Pfam-35.0, PIRSR-2021, SFLD-4, SUPERFAMILY-1.75, TIGRFAM-15.0 [73]. All functional annotations were attached via MAKER v. 2.31.9 (RRID:SCR_005309) [74].

ONT methylation call and comparison with population-level WGBS methylation calls

From our ONT reads, we called 5-methylcytosine (5mC) and 5-hydroxymethylcytosine (5hmC) modifications in the CpG (5'-C-phosphate-G-3) context via Guppy v.6.5.7 [33] in

configuration mode ‘dna_r9.4.1_450bps_modbases_5hmc_5mc_cg_hac_prom’. We focused on CpGs as this is the main methylation context in vertebrates [75]. Since methylation occurs symmetrically at CpGs in vertebrates [75], calls were de-stranded per CpG then converted to bedMethyl files using Modkit v.0.1.9 mpileup [76].

To evaluate whether our ONT-derived methylation call was comparable to population-level methylation profiles obtained via a gold-standard methylation sequencing method, we generated WGBS-derived methylomes of ten nesting adult female loggerhead turtles from the same population and locality as the reference individual (**Table S2**), using the same blood sampling protocol. Genomic DNA was extracted with a QIAGEN DNeasy® Blood and Tissue Kit (Qiagen, Germany). DNBseq libraries were constructed and sequenced with 100 bp paired-end reads on an MGI DNBSEQ platform (BGI, Hong Kong), generating 132,192,522 ± 40,360 (SD) reads per sample (**Table S3**). Extended methods for methylation calling are available in **Text S2.** Briefly, alignment and methylation calling were performed via Bismark v.0.22.1 [77] with 79.1 ± 3.37 (SD) % mapping efficiency. CpGs were de-stranded using the ‘merge_CpG.py’ script [78], resulting in a sequencing depth of 9.2 ± 0.33 (SD) X (**Table S3**). Using the R package methylKit v.1.24.0 [79], CpGs were excluded if they had a sequencing depth lower than 8X to match the filtering threshold applied to the ONT dataset, or within the 99.9th percentile to account for PCR bias [80]. Finally, CpGs were retained if they were covered in >75% of individuals. For each CpG, methylation value (%) was calculated per individual with methylKit’s ‘percMethylation’ function.

To compare ONT and WGBS methylation calls, 5mC and 5hmC modifications were first merged for the ONT dataset because WGBS cannot distinguish between them. As methylation patterns are known to differ between genomic feature types [81], we assigned CpG locations

across four categories (promoter, exon, intron, and intergenic regions; extended methods in **Text S3**) with the R packages genomation v.1.30.0 [82] and GenomicRanges v.1.50.2 [83]. Intergenic regions were considered to be gene-associated if they lay within 10 kbp (kilo base pairs) from the nearest transcription start site (TSS) [84]. Prior to conducting statistical tests, we verified that the data met underlying assumptions for parametric testing by assessing the normality and homoscedasticity of residuals. When data violated assumptions, appropriate non-parametric alternatives were used. Linear models were used to test whether (1) methylation value at gene-associated CpGs and (2) mean methylation across genes from the ONT methylome were correlated with those from the WGBS methylomes, with an interaction by gene-associated feature type. Pearson's correlation coefficients were calculated post-hoc to characterise the correlation between ONT and WGBS methylomes separately per feature type. All correlations were performed against each of the ten WGBS samples individually, as well as against the mean methylation value per CpG/gene across all ten samples. This 'average population WGBS methylome' was chosen to better represent the population-level methylation profile over individual-level noise. Finally, a chi-squared test was used to evaluate whether the frequency of highly methylated (>70%) CpGs across the whole genome were distributed differently over feature type categories between the ONT methylome and the 'average population WGBS methylome' [85]. All statistical analyses were conducted in R v.4.2.2 [86] and all plots were produced with the R package ggplot2 v.3.4.2 [87].

Genome-wide synteny between sea turtle species

To investigate genome-level synteny among different sea turtle species, we mapped our loggerhead turtle assembly against the chromosome-level green [25], leatherback [25], and hawksbill [26] turtle assemblies. Genomes were aligned using minimap2 v.2.18-r1015 with parameter '-f 0.02' [88] and dot plots were produced in D-GENIES v.1.5.0 [89].

Demographic history

We performed Pairwise Sequentially Markovian Coalescent (PSMC) analysis [90] to reconstruct the effective population size (Ne) of the Cabo Verdean loggerhead turtle population (East Atlantic), using our Illumina reads for the reference individual SLK063 [91]. To extend insights across the Atlantic Ocean, we repeated this analysis with publicly available Illumina reads for a loggerhead from a Brazilian (Bahia, West Atlantic) population (BioSample: SAMN20502673, SRA Run: SRR15328383) [92]. Reads were aligned via BWA-MEM v.0.7.17 [93], with a mapping rate of 99.8% for SLK063 and 98.7% for SAMN20502673. Alignments were sorted with SAMtools v.1.9 [48] and duplicates were tagged with Picard MarkDuplicates v.2.26.9 [94]. Variant calling and consensus building were conducted in BCFtools v.1.19 with a base and mapping filter of >Q30 [95]. Sites between a third and twice the mean sequencing depth (SLK063: ~51.7X, SAMN20502673: ~22.5X) were retained [25]. PSMC v.0.6.5 was run on the eleven macrochromosomes (1.73 Gbp, 80.8% of total assembly) with parameters '-N25 -t15 -r5 -b -p "4+25*2+4+6' [25] and 100 bootstraps. PSMC was also run on microchromosomes to verify that patterns were similar (**Figure S2)**. Outputs were scaled with a mutation rate of 1.2^{-8} [25] and a generation time of 45 years. This was calculated by adding the age of maturity to half the reproductive longevity [92], where the age of maturity was estimated as ~30 years in Cabo Verde using the length-at-age relationship [96]. Global mean surface temperature anomaly was plotted relative to pre-industrial times with climate data inferred from marine sediments [97, 98].

Genome properties

We used our Illumina data for the reference individual to estimate genome-wide heterozygosity [99]. Reads were aligned via BWA-MEM v.0.7.17 with a mapping rate of 99.6% [93], sorted

with SAMtools v.1.9 [48], then PCR duplicates were tagged with Picard MarkDuplicates v.2.26.9 [94]. Variants were called including monomorphic sites with GATK v.4.2.6.1 [100] HaplotypeCaller in '-ERC BP_RESOLUTION' mode, followed by genotyping via GenotypeGVCFs with expected heterozygosity set to 0.00179, as estimated by GenomeScope (**Figure S1**). Unused alternate alleles were removed, and sites between a third and twice the mean sequencing depth (~56.1X) were retained [25]. Other filters applied were quality by depth >2.0, root mean square mapping quality >50.0, mapping quality rank sum test >−12.5, read position rank sum test >−8.0, Fisher strand bias <60.0 and strand odds ratio <3.0 [100]. Heterozygosity was computed in non-overlapping 100 kbp windows with the 'popgenWindows.py' script in '-indHet' mode [101].

Next, we summarised a selection of genetic and methylation properties per chromosome in our reference assembly, to explore differences between the 11 macrochromosomes and 17 microchromosomes of the loggerhead genome [102]. Genetic properties included were heterozygosity (%), gene density (number of genes by chromosome length), CpG density (number of CpG sites by chromosome length), and repeat content (%). Methylation properties included were mean methylation and proportion of highly methylated (>70%) CpGs. Non-parametric Wilcoxon rank-sum tests were used to investigate median differences between chromosome types per property, since Shapiro-Wilk tests indicated non-normally distributed data. To examine relationships between properties, separate linear models were implemented to test whether different properties were correlated, with an interaction by chromosome type to test if relationships differed between macro- and microchromosomes.

TSD-linked genes: identification and synteny between sea turtle species

To identify TSD-linked genes in our loggerhead turtle genome (**Text S4** for extended methods), we used a list of 223 genes compiled by Bentley et al. (2023). These genes have documented links to TSD, primarily from studies of freshwater turtles and alligators [25]. Following manual curation of this list, we identified the longest isoform orthologues of the 'rCheMyd1.pri.v2' green turtle sequences [25] in our loggerhead turtle genome via BLASTn v.2.11.0 [103]. These were manually verified by integrating BLAST output and gene name information. For loggerhead genes that matched sequences on two chromosomes, both locations were retained if they were syntenic with the green turtle's genes, under the assumption of a conserved duplication between these sea turtle species. If one sequence was syntenic and the other not, the non-syntenic sequence was removed under the conservative assumption of assembly error. This retained 199 unique TSD-linked genes across 202 loci for downstream analyses. The chromosomal locations of TSD-linked genes in our loggerhead turtle genome were visualised and compared against the leatherback, green, and hawksbill turtle genomes with the R package Circlize v.0.4.16 [104]. All 199 TSD-linked genes in the loggerhead turtle genome annotation were present in the green and leatherback turtle genome annotations, while 192 TSD-linked genes were present in the less complete hawksbill turtle genome annotation for comparison.

TSD-linked genes: comparison of methylation patterns

We examined whether methylation differs between TSD-linked and non-TSD-linked genes in the loggerhead ONT blood methylome. For the non-TSD-linked gene set, we used single-copy orthologues representing evolutionarily conserved genes in sea turtles. These were identified with OrthoFinder v.2.5.4 [105] in nucleotide mode between our loggerhead turtle genome, as well as the green [25], leatherback [25], and hawksbill [26] turtle genomes, excluding TSD-linked genes. Overall, 11,211 single-copy orthologues were covered in our loggerhead methylome and included in this analysis. We tested whether methylation differs between gene

categories. To satisfy statistical assumptions, 1000 random subsamples of 199 orthogroups were generated for comparison against the 199 TSD-linked genes. For each subsample, a linear model was used to test if mean methylation was associated with gene category, with an interaction by feature type. A quasipoisson generalised linear model was used to test whether the highly methylated CpG count was associated with gene category in an interaction by feature type, with an offset of total CpG count.

TSD-linked genes: a functional association map

We built a functional association network for TSD-linked genes with the STRING v.12.0 database [106]. Protein sequences from the VGP green turtle [25] were available on STRING and used as query sequences. Proteins were retained if they matched the name of the target gene or had a sequence homology match >80%. This left 191 proteins, which were searched with the options: full STRING network, 0.4 confidence, and 5% false discovery rate stringency. The Markov Clustering algorithm was used to identify clusters in the network with inflation parameter 2.2 [106]. Promoter methylation status from our reference loggerhead turtle's blood methylome was also annotated onto the protein network per TSD-linked gene, with three categories based on the bimodal distribution observed: high (>70%), low (<30%) and intermediate (30-70%) methylation. A chi-squared test was used to investigate if frequencies of promoter methylation categories differed between the five largest clusters.

RESULTS AND DISCUSSION

Genomic resources generated

Genome assembly

By combining long ONT and short Illumina read sequencing, we produced a contig-level assembly (**Table 1**) with a total size of 2.146 Gbp, 1,799 contigs, and an N50 of 5.51 Mbp

(Mega base pairs). A blob-plot confirmed minimal contamination, with 99.2% of contigs mapping to Chordata and the remainder yielding no taxonomic hits (**Figure S3).** We also assembled and annotated the mitochondrial genome from our Illumina reads, consisting of a circular 16,574 bp contig with 37 genes (**Text S1, Figure S4**). Following homology-guided scaffolding against the same species [27], 98.9% (2.123 Gbp) of the assembly was placed into 28 chromosomal scaffolds, with 698 unplaced contigs (22.7 Mbp; 1.06% of assembly). This elevated our assembly to chromosome-level contiguity with an N50 of 129.73 Mbp, comparable to the best assemblies available for sea turtle species (**Table 1**). With a BUSCO score of 97.1% (Single copy: 96.2%, Duplicated: 0.9%, Fragmented: 0.4%, Missing: 2.5%) our assembly is the most complete among sea turtles after the 'rCheMyd1.pri.v2' green turtle genome [25], and the most complete loggerhead genome to date (**Table 1, Table S4** for BUSCO summaries). Quality is further supported by a QV score of Q41.1 (>99.99% assembly accuracy) and a k-mer spectrum indicating successful haploidisation (**Figure S5)**. Overall, these comparisons demonstrate that our East Atlantic loggerhead turtle assembly is a high-quality contribution to sea turtle genome resources.

**Assembly**	**Size (Gb)**	**Total scaffold / contig count**	**Longest scaffold / contig (Mbp)**	**N50 (Mbp)**	**N50 count**	**GC content (%)**	**Complete BUSCOs (%)**
**CarCar_QM_v1.21.12** (Loggerhead sea turtle, contig)	2.146	1799	24.43	5.51	118	43.94	97.1
**CarCar_QM_v1.21.12_Sc** (Loggerhead sea turtle, scaffolded)	2.146	726	352.82	129.73	5	43.94	97.1
**CarCar_GSC_CCare_1.0** (Loggerhead sea turtle, scaffolded)	2.134	2008	345.74	130.96	5	44.03	96.1
**rDerCor1.pri.v4** (Leatherback sea turtle, scaffolded)	2.165	41	354.45	137.57	5	43.35	96.3
**rCheMyd1.pri.v2** (Green sea turtle, scaffolded)	2.134	93	348.27	134.43	5	44.01	97.2
**CheMyd_1.0** (Green sea turtle, contig)	2.132	140,023	22.92	4.07	149	43.43	95.9
**ASM3001250v1** (Hawksbill sea turtle, scaffolded)	2.296	208	367.35	137.21	5	44.13	97.1

Table 1. Comparison of assembly quality across sea turtle species. 'CarCar_QM_v1.21.12' is our contig-level and 'CarCar_QM_v1.21.12_Sc' is our chromosome-level loggerhead sea turtle (*Caretta*

caretta) assembly representing the East Atlantic population. 'CarCar_GSC_CCare_1.0' is the chromosome-level loggerhead sea turtle assembly representing the Adriatic Sea population [27]. 'rCheMyd1.pri.v2' and 'rDerCor1.pri.v4' are chromosome-level green (*Chelonia mydas*) and leatherback (*Dermochelys coriacea*) sea turtle assemblies respectively [25]. 'CheMyd_1.0' is the first, draft green sea turtle assembly [54]. 'ASM3001250v1' is the chromosome-level hawksbill sea turtle (*Eretmochelys imbricata*) assembly [26]. BUSCO scores (full summaries in **Table S4**) were calculated against the Sauropsida gene set (n=7480) with BUSCO v.5.1.2 [49].

Genome annotation

We identified and masked 919 Mbp (42.8% of assembly) of repetitive elements (**Table S5** for element type breakdown). A total of 33,887 protein-coding genes were annotated with a mean gene length of 51.0 kbp, of which 27,817 genes (82.1%) were functionally annotated via homology and 19,966 genes (58.9%) were assigned GO terms (**Table S6** for full gene annotation statistics). Our annotation had a BUSCO completeness score of 95.4% (Single copy: 94.4%, Duplicated: 1.0%, Fragmented: 1.1%, Missing: 3.5%), which is of intermediate completeness between existing chromosome-level annotations for sea turtles (**Table S7** for full BUSCO summaries). Future improvements could involve optimising species-specific tuning and manual curation steps through collaborations with annotation experts [107].

Methylation call

By calling methylation from our ONT reads, we provide an additional layer of molecular information to facilitate epigenomic insights for loggerhead sea turtle conservation via minimally invasive blood sampling. Out of 26,449,075 CpGs in total, 22,327,230 CpGs had only 5mC modifications, 120,983 CpGs had only 5hmC modifications, and 2,986,762 CpGs had a combination of both (**Table 2**). The mean genome-wide methylation level was 76.0% and the proportion of highly methylated (>70%) CpGs was 74.1% for the ONT methylome. We also assessed whether our reference individual's ONT methylome was comparable to ten additional methylomes of adult female nesting loggerheads from the same population. These were sequenced via WGBS, as this is the current gold-standard for base-resolution methylation

analysis [108]. Averaged across all ten individuals, the mean genome-wide methylation level was 75.5 ± 0.75 (SD) % and the proportion of highly methylated (>70%) CpGs was 75.2 ± 1.58 (SD) % (**Table S3**). Methylation estimates were therefore consistent between the reference ONT and population-level WGBS methylation calls, as well as a reported genome-wide methylation level of ~70% across Testudines [75]. Highly methylated CpGs were similarly distributed across feature types between the ONT and 'average population WGBS methylome' (χ^2=0.0387, p=0.998, **Figure S6**).

At 9,341,292 gene-associated CpGs covered by both datasets, per-CpG methylation values between the ONT and 'average population WGBS methylome' exhibited an interaction by feature type ($F_{1,9303195}$=33,969, p<0.0001, **Figure 2A**), due to different positive correlations for each feature type (Exons: r(486,723)=0.81, p<0.0001; Introns: r(6,895,343)=0.75, p<0.0001; Promoters: r(499,018)=0.95, p<0.0001, Intergenic: r(1,422,111)=0.84, p<0.0001). When focusing on mean methylation per gene at 25,161 genes covered in both datasets, we also found an interaction by feature type ($F_{1,84631}$=210.3, p<0.0001, **Figure 2B**), with positive correlations and higher correlation coefficients than the CpG-based analysis (Exons: r(20,348)=0.93, p<0.0001; Introns: r(18,968)=0.96, p<0.0001; Promoters: r(23,771)=0.98, p<0.0001, Intergenic: r(21,544)=0.95, p<0.0001). This result is expected given the smoothening of individual-level noise on the per-gene level analysis versus the per-site level analysis [109]. Note that results were comparable when testing the ONT methylome against each of the ten WGBS methylomes separately (**Table S8-9**).

These results suggest that our ONT-derived methylome is representative of genome-wide methylation profiles for nesting female loggerheads from the East Atlantic population. Previous benchmarking studies via paired ONT and WGBS sequencing further demonstrate

ONT as a robust alternative approach for methylation analysis [110, 111, 112, 113], particularly following the arrival of R10.4 flow cell chemistry with <1% sequencing error rates [114]. By measuring real-time ionic current fluctuations, ONT enables the simultaneous acquisition of genomic and methylation sequencing data from native DNA [115, 116]. This maximises molecular insights while removing technical biases introduced by amplification and bisulfite conversion processes. Unlike WGBS, ONT can also distinguish between base modification types which have different regulatory implications, such as 5mC and 5hmC [117]. Together, these properties support ONT as a cost-effective and powerful sequencing method for conservation epigenomic studies.

ONT methylation call statistics	
Total CpGs	26,449,075
Total CpGs with 5mC modifications only	22,327,230 (84.4%)
Total CpGs with 5hmC modifications only	120,983 (0.46%)
Total CpGs with 5mC and 5hmC modifications	2,986,762 (11.3%)
Total unmethylated CpGs	1,014,100 (3.83%)
Mean / median sequencing depth per CpG	17.0 / 16.0

Table 2. ONT methylation call statistics for the reference individual.

Application of genomic resources

Genome-wide synteny between sea turtle species

The loggerhead turtle genome displayed high conservation of synteny against the leatherback (**Figure 1B**), green (**Figure 1C)**, and hawksbill turtle genomes (**Figure 1D**). Possible inversions were detected on chromosomes 4 and 9 of the loggerhead turtle genome against all other species, with future validation required to determine whether these represent true loggerhead-specific rearrangements or assembly artefacts. Sequence similarity between pairwise alignments (**Table S10**) was highest between the loggerhead and hawksbill turtle genomes (>50% identical: 97.4%, >75% identical: 16.4%), followed by the green turtle genome (>50% identical: >90.1%, >75% identical: 0.01%), and lastly the leatherback turtle

genome (>50% identical: 3.37%, >75% identical: 0%). This is coherent with phylogenetic expectations: within the Cheloniidae family, loggerheads turtles diverged from hawksbill turtles ~20 million years ago (Mya) and from green turtles ~40 Mya, compared to the deeper split of ~75 Mya from leatherback turtles in the Dermochelyidae family [92]. Overall, our results extend the observation of high genomic stability within the sea turtle lineage [25].

Demographic history

We used PSMC to reconstruct the effective population size (Ne) of loggerheads from East Atlantic (ID: SLK063, Cabo Verde) and West Atlantic (ID: SAMN20502673, Bahia State, Brazil) [92] populations. An overall decline in Ne was detected across ~17 million years of reconstruction (**Figure 3**). In line with contemporary estimates, the East Atlantic population had a higher Ne (~5000) than the West Atlantic population (~2000) near present times, although the West Atlantic population had higher Ne over the populations' histories. Interestingly, Ne fluctuations were similar in timing and amplitude for both populations. This suggests that major climatic and oceanic processes affecting the entire Atlantic Ocean, rather than region-specific events, were the primary drivers of loggerhead turtle demographic changes. Specifically, a population contraction occurred during the onset and intensification of Northern Hemisphere Glaciation (~3.3-2.4 Mya), as global temperatures and atmospheric carbon dioxide dropped and ice sheets expanded [118, 119]. This period of cooling, coupled with the migration of high productivity centres from polar to equatorial regions, likely reduced habitable zones for loggerhead turtles, as reflected in the relatively rapid decrease in Ne during this interval. During the mid-Pleistocene Transition (~1.25-0.60 Mya), prolonged and intensified glacial intervals resulted in glacial cooling and expansion of ice sheets [120]. At that time, productivity centres shifted from equatorial regions toward subpolar regions and were increasingly variable on glacial-interglacial time scales [121, 122]. These shifts may

coincide with Ne expansion in loggerhead turtles, which also matches their proposed migration history in the Atlantic Ocean [123]. Altogether, our new loggerhead turtle genome enabled us to trace population-level dynamics, emphasising the importance of climate and niche availability for sea turtles. Such information can inform predictive models of demographic responses to current anthropogenic climate change.

Genome properties

Genome-wide heterozygosity was estimated as ~0.12% for our loggerhead turtle genome, in alignment with ~0.11% reported for the Adriatic loggerhead turtle genome [27]. This places the genomic diversity of loggerhead turtles at approximately four times that of leatherback turtles (~0.0029%), less than half of green turtles (~0.25%), and about a third of hawksbill turtles (~0.33%) [25, 26]. Genome-wide heterozygosity patterns further varied among the 28 chromosomes, with microchromosomes being more heterozygous than macrochromosomes (W=29, p=0.002, **Figure S7A**). Heterozygosity was predicted by an interaction between chromosome length and type (Length x Type: $F_{1,24}$=12.3, p=0.002, **Figure 4A)**, with a negative correlation in microchromosomes ($F_{1,15}$=7.88, p=0.013) but none detected for macrochromosomes ($F_{1,9}$=1.09, p=0.324). Microchromosomes were also more gene-dense (W=28, p=0.001, **Figure S7B**), GC-rich (W=1, p<0.0001, **Figure S7C**), CpG-dense (W=1, p<0.0001, **Figure S7D**), and less repeat-rich overall (W=166, p=0.0003, **Figure S7E**). This is consistent with patterns described in other sea turtles [25] and wider vertebrates with both macro- and microchromosomes [124, 125].

We next performed comparisons to address the knowledge gap of methylation differences between macro- and microchromosomes. From our ONT-derived blood methylome, mean methylation was similar between chromosome types (W=54, p=0.07, **Figure S7F**), but

microchromosomes had a greater proportion of highly methylated CpGs ($W=54$, $p<0.0001$; **Figure S7G**). This likely stems from higher CpG density on microchromosomes providing more methylatable sites [126], as supported by a strong, positive correlation (CpG density: $F_{1,24}=56.5$, $p<0.0001$, Type: $F_{1,24}=8.67$, $p=0.007$, **Figure 4B**) without an interaction by chromosome type (CpG density x Type: $F_{1,24}=0.08$, $p=0.896$). This is functionally interesting as previous studies have shown that highly methylated CpG sites are often regulated more dynamically than lowly methylated CpG sites [127]. Correlations without interactions were also observed between the proportion of highly methylated CpGs against heterozygosity (Heterozygosity: $F_{1,24}=50.30$, $p<0.0001$, Type: $F_{1,24}=17.03$, $p=0.0004$, Heterozygosity x Type: $F_{1,24}=0.04$, $p=0.84$; **Figure 4C**) and gene density (Gene density: $F_{1,24}=65.89$, $p<0.0001$, Type: $F_{1,24}=8.67$, $p=0.007$, Gene density x Type: $F_{1,24}=0.048$, $p=0.83$; **Figure 4D**), suggesting relationships between methylation and those genetic properties are shaped by evolutionary forces acting consistently across chromosome types. There was no correlation between the proportion of highly methylated CpGs and repeat content ($F_{1,24}= 1.64$, $p=0.21$).

In addition to microchromosomes offering a greater combination of genetic variants via independent assortment, our results highlight the unique genetic properties of vertebrate microchromosomes and add novel insights into their methylation potential. With higher heterozygosity, gene density, and regulatory opportunities via CpG methylation, microchromosomes could serve as hotspots for both adaptive evolution and plastic responses to environmental change. As such, microchromosomes represent promising targets for monitoring and preserving functional genetic and epigenetic diversity in sea turtles and other vertebrates [128].

TSD-linked genes: synteny between sea turtle species

As TSD species, loggerhead turtles are particularly vulnerable to climate change, with strongly female-biased sex ratios and population collapse predicted by the end of the century [20]. To gain molecular insights into this system, we compared the chromosomal locations of 199 TSD-linked genes present in our loggerhead turtle genome against other available sea turtle genomes. Consistent with Bentley et al. (2023), most TSD-linked genes were single-copy and reside on equivalent chromosomes across sea turtle species (**Figure 5A-C**) [25]. Two genes were syntenic between all species except the leatherback turtle (**Figure 5A**): CIRBP (Cold-inducible RNA-binding protein; loggerhead/green: chromosome 25; leatherback: chromosome 27) and IFIT5 (Interferon Induced Protein with Tetratricopeptide Repeats 5; loggerhead/green: chromosome 7; leatherback: chromosome 1). Only the EP300 (E1A binding protein p300) gene was not syntenic between loggerheads and greens (**Figure 5B**). Although it mapped to chromosome 1 in all species, a duplicate on chromosome 10 was found in the green and leatherback genomes. All TSD-linked genes in the loggerhead turtle genome were syntenic with those present in the hawksbill turtle genome (**Figure 5C**).

As rearrangements and copy number variants can alter gene regulation [129], the three identified genes may contribute to inter-species differences in the TSD response curve and should be investigated. Nevertheless, structural variants seem rare in sea turtles overall, unlike in some other Testudines [130, 131]. Finer scale genetic variation may instead be more important, particularly within species. For example, a SNP on the CIRBP gene, which we found to be non-syntenic against leatherbacks, influences TSD in snapping turtles (*Chelydra serpentina*) and exhibits varying allele frequencies with latitude [132]. It is thus crucial to continue characterising genetic variants that contribute to the adaptive potential of the TSD mechanism, to evaluate whether different sea turtle species and populations can evolve to mitigate sex ratio skew as climate change progresses.

TSD-linked genes: methylation patterns

Given epigenetic mechanisms are involved in TSD regulation, we next provide broad insights into the methylation profile of 199 TSD-linked genes from our reference ONT blood methylome. Methylation distributions were similar between TSD-linked and non-TSD-linked genes comprised of 11,211 single-copy orthologues identified between sea turtle species (**Figure 5D, Figure S8**). This was supported by performing linear models comparing the 199 TSD-linked genes against 1000 random subsets of 199 non-TSD-linked genes, which revealed feature type as the sole determinant of both the mean methylation level (**Figure S9**) and the proportion of highly methylated CpG per gene (**Figure S10**). Exons, introns, and gene-associated intergenic space (<10 kbp from TSS) were mostly hypermethylated, with a peak centred on ~80% (**Figure 5D**, **Figure S8**). In contrast, methylation was bimodally distributed for promoters, with peaks of low (~10%) and high (~80%) methylation. These distributions are consistent with those described across mammalian vertebrates [75, 133, 134].

Overall, we provide a genome-wide description of blood-based methylation patterns across gene feature types to guide future study design for sea turtles. Blood is a valuable tissue for conservation monitoring, particularly in reptiles with nucleated erythrocytes, as it can be collected via minimally invasive sampling yet still report on an individual's developmental status [135, 136, 137], health [138], and environmental exposure [139, 140]. For example, a non-lethal method for sexing sea turtle hatchlings is urgently required to assess nest sex ratios as global temperatures continue to rise [20]. As expected, we did not uncover obvious differences between TSD-linked versus non-TSD-linked genes from blood tissue of an adult female loggerhead's genome, likely given the broad scale of comparison and the timing- and tissue-specificity of epigenetic regulation [22, 24]. Future studies could therefore employ

methylome-wide discovery scans [141] to identify blood-based biomarkers of sex in sea turtle hatchlings, as performed for American alligators (*Alligator mississippiensis*), another TSD reptilian species [142].

TSD-linked genes: a functional association map

Using the STRING database [106], we built a network of functional associations between proteins coded by TSD-linked genes **(Figure 5E**). In total, 119 TSD-linked genes had at least one connection identified via Markov Clustering (n=28 clusters total), and the five largest clusters encompassed 54 TSD-linked genes (45.4%). Members of the biggest cluster (n=15, dark blue) were mainly linked to Wnt signalling. Connections were centred on CTNNB1 (beta-catenin), which is critical for female determination in vertebrates [143, 144]. The next largest cluster (n=12, dark purple) was primarily composed of epigenetic regulators. The most connected protein was EP300 (E1A binding protein p300), a histone acetyltransferase that controls cell proliferation, with germ cell numbers proposed to influence TSD in a freshwater turtle [145]. The third cluster (n=11, mauve) contained genes with well-described roles in TSD and steroid hormone signalling [139]. SOX9 (SRY-related HMG box gene 9), a key regulator of male differentiation [146, 147], was most connected. Although the functional nature of the fourth cluster (n=8, pink) was less obvious, the most connected protein was the histone demethylase KDM6B (Lysine Demethylase 6B), demethylase which is causally linked to temperature-dependent male determination in freshwater turtles [23]. The final cluster contains heat shock proteins and chaperones (n=7, yellow), which could play a role in temperature sensing and transduction during TSD [149]. These protein clusters may already suggest patterns surrounding TSD in sea turtles. Sensory systems associated with heat shock responses could incorporate environmental inputs that link with epigenetic regulators to alter gene

expression, cell proliferation, and subsequently activate known endocrine systems [150]. With growing genomic tools for sea turtles, this hypothesis will need to be tested.

Given the prevalence of epigenetic regulators in the functional network, we overlaid promoter methylation status of TSD-linked genes from our reference loggerhead turtle's blood methylome (**Figure 5E**). In total, 67 genes (33.8%) were highly (>70%) methylated, 96 genes (48.0%) were lowly (<30%) methylated, and 36 genes (18.2%) had intermediate methylation. The proportions of each promoter methylation category differed amongst the five largest functional clusters (χ^2=41.697, p<0.0001, **Table S11**). The lowest methylated clusters involved Wnt signalling genes (dark blue; high: 33.3%, intermediate: 6.7%, low: 60%) and the well-described TSD-linked/hormone signalling genes (mauve; high: 20%, intermediate: 20%, low: 60%). No clusters were hypermethylated, as the heat shock cluster (yellow) contained the largest proportion of highly methylated genes, with an equal split between highly and lowly methylated genes. With promoter methylation classically linked to transcriptional repression [81], we may have captured female-specific methylation signatures on a broad gene network scale. Overall, this network serves to facilitate future investigations into the relationships between TSD-linked gene function, connectivity, and blood methylation status to aid sex biomarker discovery in sea turtles.

CONCLUSIONS

To support conservation efforts of threatened loggerhead sea turtles, as well as all sea turtles and TSD species in general, we present a chromosome-level genome representing the globally significant East Atlantic nesting group. Our reference assembly will enhance genomic inferences for this population, and contribute a new genome for comparative studies within and between species. Moreover, we anticipate our ONT-derived blood methylome profile can guide

future epigenetic study design. To demonstrate their potential, we applied these novel resources to yield a variety of insights relevant to the conservation of sea turtles. At the population level, we emphasise the role of climate change and niche availability on effective population size fluctuations. On the chromosome level, we recommend microchromosomes as special regions for monitoring functional genetic and epigenetic diversity. Finally, we bring gene-level insights into the TSD cascade of sea turtles, highlighting three TSD-linked genes with potential inter-species rearrangements, and providing a map of functional associations and blood-based methylation status for TSD-linked genes in an adult nesting female. By simultaneously generating genome and methylome resources to provide molecular insights in loggerhead sea turtles, our study showcases the application of this dual framework for informing the conservation of an endangered flagship species.

DATA AVAILABILITY

All sequencing data and the annotated genome assembly 'CarCar_QM_v1.21.12_Sc' are available under study PRJEB79015 on the European Nucleotide Archive (ENA). Supporting scripts are deposited in our GitHub repository [151]. Additional genome assembly, annotation, and methylome files mentioned in this article are available on *GigaScience* repository, GigaDB, alongside supporting data analyses [152].

LIST OF ABBREVIATIONS

5mC: 5-methylcytosine, 5hmC: 5-hydroxymethylcytosine, bp: base pairs, CBP: Canada BioGenome Project, CpG: 5'-C-phosphate-G-3, ENA: European Nucleotide Archive, Gbp: giga base pairs, kbp: kilo base pairs, Mbp: mega base pairs, Mya: million years ago, Ne: effective population size, ONT: Oxford Nanopore Technology, PCR: polymerase chain

reaction, PSMC: Pairwise Sequentially Markovian Coalescent, TSS: transcription start site, VGP: Vertebrate Genomes Project, WGBS: whole genome bisulfite sequencing

SUPPLEMENTARY FILES

Table S1. Publicly available RNA-Seq reads mined for genome annotation

Table S2. Metadata for ten nesting loggerheads sampled for WGBS

Table S3. WGBS summary statistics for ten nesting loggerheads

Table S4. Full BUSCO summary for genome assemblies across sea turtle species

Table S5. Repetitive element summary statistics

Table S6. Gene annotation summary statistics

Table S7. Full BUSCO summary for genome annotations across sea turtle species

Table S8. Linear model results for the interaction term per individual WGBS methylome

Table S9. Pearson's correlation test results per individual WGBS methylome

Table S10. Summary of whole genome alignments between the loggerhead sea turtle and other sea turtle species

Table S11. Proportions of promoter methylation categories in the top five functional clusters

Text S1. Extended methods and results for mitochondrial genome assembly and annotation

Text S2. Extended methods for methylation calling from WGBS data of ten loggerheads

Text S3. Extended methods for annotating gene feature types

Text S4. Extended methods for identification and curation of TSD-linked genes

Figure S1. GenomeScope profile for our loggerhead genome

Figure S2. Auxiliary PSMC tests with 100 bootstraps

Figure S3. GC-coverage blob-plot to evaluate assembly contamination

Figure S4. Mitochondrial genome assembly and annotation

Figure S5. K-mer spectrum plot of our loggerhead assembly

Figure S6. Genome-wide distribution of highly methylated (>70%) CpGs across feature types by site

Figure S7. Comparison of macro- and microchromosome properties

Figure S8. Violin plot of mean methylation per gene

Figure S9. P-value histogram from comparing mean methylation between TSD-linked genes and non-TSD-linked genes

Figure S10. P-value histogram from comparing the proportion of highly methylated CpGs between TSD-linked genes and non-TSD-linked genes

DECLARATIONS

Ethics approval

All sample collection adhered to national legislation and were approved by the Direção Nacional do Ambiente de Cabo Verde (Permits: 13/DNA/2020, 037/DNA/2021).

Competing interests

The authors declare they have no competing interests.

Funding

This work was funded by UK Research and Innovation (NERC, NE/V001469/1, NE/X012077/1 to C.E and J.M.M-D) and National Geographic (NGS-59158R-19 to C.E) grants. Additional financial support was awarded by the London NERC Doctoral Training Partnership studentship to E.C.Y (NERC, NE/S007229/1).

Author contributions

E.C.Y and C.E designed the study. E.C.Y, C.E, A.T and K.F collected blood samples. E.C.Y performed genome assembly, annotation and quality assessment, with contributions from J.M.M-D. A.B, E.C.Y and D-M.J.T performed DNA methylation analysis of ONT reads. E.C.Y performed sample processing and methylation analysis for WGBS. E.C.Y performed genome property and synteny analyses. E.C.Y performed PSMC, with input and guidance on geoclimatic events from H.L.F. J.D.G identified TSD-linked genes, orthogroup genes, mapped chromosomal locations and produced the functional association network. E.C.Y analysed methylation of TSD-linked genes. E.C.Y wrote the manuscript with contributions from C.E, J.D.G, H.L.F, S.J.R, J.M.M-D and feedback from all authors.

Acknowledgements

The authors thank all staff with Project Biodiversity (Sal, Cabo Verde) for their support in the field. We also thank Chloe Economou (Queen Mary University of London, UK) for conducting DNA extractions for the reference individual and William Tyne (Queen Mary University of London, UK) for ONT library preparation and sequencing.

FIGURE LEGENDS

Figure 1. A chromosome-level genome assembly for loggerhead sea turtles from the East Atlantic nesting aggregation. (A) A loggerhead sea turtle (*Caretta caretta;* CC) nesting in Cabo Verde on Sal Island, our reference population. Photo credit: Project Biodiversity. **(B-D)** Whole genome alignment dotplots of our loggerhead sea turtle assembly against publicly available chromosome-level assemblies for the **(B)** leatherback sea turtle (*Dermochelys coriacea;* DC; top 100,000 alignments shown), **(C)** green sea turtle (*Chelonia mydas;* CM), and **(D)** hawksbill sea turtle (*Eretmochelys imbricata*; EI). Axes show chromosome number

(1-11: macrochromosomes, 12-28: microchromosomes, 0: unplaced contigs) and colours represent alignment sequence identity (%).

Figure 2. Comparison of the ONT methylome with population-level WGBS methylomes. Correlation of methylation value (%) between the reference individual's ONT-derived methylome and the 'average population WGBS methylome', which represents the mean methylation of ten additional loggerhead sea turtles sequenced from the same population via WGBS. Correlations are split by gene feature type (exons, introns, promoters, and gene-associated intergenic regions <10 kbp from the TSS), plotted for **(A)** methylation per gene-associated CpG for a random subsample of 100,000 out of 9,341,292 gene-associated CpGs covered in both datasets, and **(B)** mean methylation per gene by feature type across 25,161 genes covered in both datasets.

Figure 3. Demographic history reconstruction for loggerhead sea turtle populations across the East and West Atlantic Ocean. The East Atlantic population is in Cabo Verde (SLK063, blue line) and the West Atlantic population is in Bahia State, Brazil (SAMN20502673, purple line) [92]. Effective population size (Ne, x10^4) was reconstructed using PSMC over ~17 Mya (log-scaled). Marine sediments were used to infer the global mean surface temperature anomaly (red and blue climate stripes) compared to pre-industrial times [97, 98]. Letters designate geoclimatic events of interest: A: Northern Hemisphere Glaciation (~3.3 to 2.4 Mya). B: Mid-Pleistocene Transition (~1.25 to 0.60 Mya).

Figure 4. Comparison of chromosome-level characteristics. Relationships between genome properties in macro- (dark blue) versus microchromosomes (yellow) of the loggerhead turtle genome. **(A)** Mean heterozygosity (%) exhibits an interaction by chromosome length (Gbp)

and type, with a negative correlation in microchromosomes but no correlation in macrochromosomes. **(B)** The proportion of highly methylated (>70%) CpGs (%) is higher overall for microchromosomes, and positively correlates with CpG density. A similar relationship is observed between highly methylated CpGs (%) against **(C)** mean heterozygosity (%), and **(D)** gene density (total genes by chromosome length x10^{-5}).

Figure 5. Molecular insights into TSD-linked genes: synteny, methylation, and protein functional associations. (A-B) Chromosomal locations of 199 TSD-linked genes across 201 loci in our loggerhead sea turtle assembly against the **(A)** leatherback (DC), **(B)** green (CM), and (**C**) hawksbill (EI) chromosome-level sea turtle assemblies. Chromosomes of the loggerhead assembly are plotted on the left, with colours representing the 28 chromosomes. Dashed red lines indicate genes that mapped to different locations between species (loggerhead versus green: EP300, loggerhead versus leatherback: EP300, CIRBP, IFIT5). **(D)** Density plots of mean methylation (%) per gene by feature type between 199 TSD-linked genes (purple) and a random subset of 199 out of 11,211 non-TSD-linked genes (yellow), identified as single-copy orthologue between sea turtle species (yellow). **(E)** Predicted functional association network of proteins coded by TSD-linked genes, created with STRING [107]. 119 genes with at least one connection are shown (n=28 clusters total). Nodes represent gene IDs and edges represent associations. Edge colours represent different evidence types used for association prediction. Non-grey node colours represent the five largest functional clusters identified via Markov Clustering. Letters on nodes represent methylation status of gene promoters in the reference individual methylome. Categories are based on the bimodal methylation distribution observed in **Figure 5D**: H: highly methylated (>70%), L: lowly methylated (<30%), I: intermediate methylation (30-70%). 67 promoters (33.8%) were hyper-methylated, 96 (48%) were hypo-methylated and 36 (18.2%) had intermediate methylation.

REFERENCES

1. Ceballos G, Ehrlich PR, Barnosky AD, García A, Pringle RM, Palmer TM. Accelerated modern human–induced species losses: Entering the sixth mass extinction. *Sci Adv*. 2015; doi: 10.1126/sciadv.1400253.

2. Theissinger K, Fernandes C, Formenti G, Bista I, Berg PR, Bleidorn C, et al. How genomics can help biodiversity conservation. *Trends in Genetics*. 2023; doi: 10.1016/j.tig.2023.01.005.

3. Coates DJ, Byrne M, Moritz C. Genetic Diversity and Conservation Units: Dealing With the Species-Population Continuum in the Age of Genomics. Front Ecol Evol. Frontiers; 2018; doi: 10.3389/fevo.2018.00165.

4. Wold J, Koepfli K-P, Galla SJ, Eccles D, Hogg CJ, Le Lec MF, et al.. Expanding the conservation genomics toolbox: Incorporating structural variants to enhance genomic studies for species of conservation concern. *Molecular Ecology*. 2021; doi: 10.1111/mec.16141.

5. Kardos M, Taylor HR, Ellegren H, Luikart G, Allendorf FW. Genomics advances the study of inbreeding depression in the wild. *Evolutionary Applications*. 2016; doi: 10.1111/eva.12414.

6. Karamanlidis AA, Skrbinšek T, Amato G, Dendrinos P, Gaughran S, Kasapidis P, et al..Genetic and demographic history define a conservation strategy for earth's most

endangered pinniped, the Mediterranean monk seal Monachus monachus. *Sci Rep*. 2021; doi: 10.1038/s41598-020-79712-1.

7. Flanagan SP, Forester BR, Latch EK, Aitken SN, Hoban S. Guidelines for planning genomic assessment and monitoring of locally adaptive variation to inform species conservation. *Evolutionary Applications*. 2018; doi: 10.1111/eva.12569.

8. Eizaguirre C, Baltazar-Soares M. Evolutionary conservation—evaluating the adaptive potential of species. *Evolutionary Applications*. 2014; doi: 10.1111/eva.12227.

9. Balard A, Baltazar-Soares M, Eizaguirre C, Heckwolf MJ. An epigenetic toolbox for conservation biologists. *Evolutionary Applications*. 2024; doi: 10.1111/eva.13699.

10. Feil R, Fraga MF. Epigenetics and the environment: emerging patterns and implications. *Nature Reviews Genetics*. 2012; doi: 10.1038/nrg3142.

11. Rey O, Eizaguirre C, Angers B, Baltazar-Soares M, Sagonas K, Prunier JG, et al. Linking epigenetics and biological conservation: Towards a conservation epigenetics perspective. *Functional Ecology*. 2020; doi: 10.1111/1365-2435.13429.

12. Lamka GF, Harder AM, Sundaram M, Schwartz TS, Christie MR, DeWoody JA, et al. Epigenetics in Ecology, Evolution, and Conservation. *Frontiers in Ecology and Evolution*. 2022; 10.3389/fevo.2022.871791.

13. Laine VN, Sepers B, Lindner M, Gawehns F, Ruuskanen S, van Oers K. An ecologist's guide for studying DNA methylation variation in wild vertebrates. *Molecular Ecology Resources*. 2023; doi: 10.1111/1755-0998.13624.

14. IUCN. The IUCN Red List of Threatened Species (Version 2024-1). 2024; https://www.iucnredlist.org. Accessed July 2024.

15. Wallace BP, DiMatteo AD, Bolten AB, Chaloupka MY, Hutchinson BJ, Abreu-Grobois FA, et al. Global Conservation Priorities for Marine Turtles. *PLOS ONE*. 2011; doi: 10.1371/journal.pone.0024510.

16. Yntema CL, Mrosovsky N. Critical periods and pivotal temperatures for sexual differentiation in loggerhead sea turtles. *Can J Zool*. 1982; doi: 10.1139/z82-141.

17. Hawkes L, Broderick A, Godfrey M, Godley B. Climate change and marine turtles. *Endang Species Res*. 2009; doi: 10.3354/esr00198.

18. Laloë J-O, Cozens J, Renom B, Taxonera A, Hays GC. Effects of rising temperature on the viability of an important sea turtle rookery. *Nature Climate Change*. 2014; doi: 10.1038/nclimate2236.

19. Mitchell N, Janzen FJ. Temperature-Dependent Sex Determination and Contemporary Climate Change. *Sexual Development*. 2010; doi: 10.1159/000282494.

20. Lockley EC, Eizaguirre C. Effects of global warming on species with temperature-dependent sex determination: Bridging the gap between empirical research and management. *Evol Appl*. 2021. doi: 10.1111/eva.13226.

21. Komoroske LM, Jensen MP, Stewart KR, Shamblin BM, Dutton PH. Advances in the Application of Genetics in Marine Turtle Biology and Conservation. *Front Mar Sci*. *Frontiers*. 2017; doi: 10.3389/fmars.2017.00156.

22. Venegas D, Marmolejo-Valencia A, Valdes-Quezada C, Govenzensky T, Recillas-Targa F, Merchant-Larios H. Dimorphic DNA methylation during temperature-dependent sex determination in the sea turtle Lepidochelys olivacea. *General and Comparative Endocrinology*. 2016; doi: 10.1016/j.ygcen.2016.06.026.

23. Ge C, Ye J, Weber C, Sun W, Zhang H, Zhou Y, et al.. The histone demethylase KDM6B regulates temperature-dependent sex determination in a turtle species. *Science*. American Association for the Advancement of Science. 2018; doi: 10.1126/science.aap8328.

24. Piferrer F. Epigenetic mechanisms in sex determination and in the evolutionary transitions between sexual systems. *Philosophical Transactions of the Royal Society B: Biological Sciences*. Royal Society. 2021; doi: 10.1098/rstb.2020.0110.

25. Bentley BP, Carrasco-Valenzuela T, Ramos EKS, Pawar H, Souza Arantes L, Alexander A, et al. Divergent sensory and immune gene evolution in sea turtles with contrasting demographic and life histories. *Proceedings of the National Academy of Sciences*. 2023; doi: 10.1073/pnas.2201076120.

26. Guo Y, Tang J, Zhuo Z, Huang J, Fu Z, Song J, et al.. The first high-quality chromosome-level genome of Eretmochelys imbricata using HiFi and Hi-C data. Sci Data. 2023; doi: 10.1038/s41597-023-02522-3.

27. Chang G, Jones S, Leelakumari S, Ashkani J, Culibrk L, O'Neill K, et al.. The genome sequence of the Loggerhead sea turtle, Caretta caretta Linnaeus 1758. *F1000Res*. 2023; doi: 10.12688/f1000research.131283.2.

28. Thorburn D-MJ, Sagonas K, Binzer-Panchal M, Chain FJJ, Feulner PGD, Bornberg-Bauer E, et al.. Origin matters: Using a local reference genome improves measures in population genomics. *Molecular Ecology Resources*. 2023; doi: 10.1111/1755-0998.13838.

29. Taxonera A, Fairweather K, Jesus A, Gonzalves A, Queiruga A, et al. Cabo Verde: Sea Turtles "In Abundance". In: SWOT Report - State of the World's Sea Turtles Vol. XVII. 2022; https://www.seaturtlestatus.org/articles/cabo-verde-sea-turtles-in-abundance. Accessed June 2023.

30. Stiebens VA, Merino SE, Roder C, Chain FJJ, Lee PLM, Eizaguirre C. Living on the edge: how philopatry maintains adaptive potential. *Proceedings of the Royal Society B: Biological Sciences*. 2013; doi: 10.1098/rspb.2013.0305.

31. Owens DWM, Ruiz GJ. New Methods of Obtaining Blood and Cerebrospinal Fluid from Marine Turtles. *Herpetologica*. 1980; 36:17–20.

32. Zhang T, Li H, Ma S, Cao J, Liao H, Huang Q, et al.. The newest Oxford Nanopore R10.4.1 full-length 16S rRNA sequencing enables the accurate resolution of species-level microbial community profiling. Applied and Environmental Microbiology. American Society for Microbiology; 2023; doi: 10.1128/aem.00605-23.

33. Wick RR, Judd LM, Holt KE. Performance of neural network basecalling tools for Oxford Nanopore sequencing. *Genome Biology*. 2019; doi: 10.1186/s13059-019-1727-y.

34. King T, Butcher S, & Zalewski L. (2017). Apocrita - High Performance Computing Cluster For Queen Mary University Of London. *Zenodo*. 10.5281/ZENODO.438045.

35. Wick RR. (2018). PoreChop (Version 0.2.4). https://github.com/rrwick/Porechop/releases/tag/v0.2.4. Accessed July 2024.

36. Filipović I, Rašić G, Hereward J, Gharuka M, Devine GJ, Furlong MJ, et al.. A high-quality de novo genome assembly based on nanopore sequencing of a wild-caught coconut rhinoceros beetle (Oryctes rhinoceros). *BMC Genomics*. 2022; doi: 10.1186/s12864-022-08628-z.

37. De Coster W, D'Hert S, Schultz DT, Cruts M, Van Broeckhoven C. NanoPack: visualizing and processing long-read sequencing data. *Bioinformatics*. 2018; doi: 10.1093/bioinformatics/bty149.

38. Vurture GW, Sedlazeck FJ, Nattestad M, Underwood CJ, Fang H, Gurtowski J, et al.. GenomeScope: fast reference-free genome profiling from short reads. *Bioinformatics*. 2017; doi: 10.1093/bioinformatics/btx153.

39. Krueger F. (2019). TrimGalore (Version 0.6.5). https://github.com/FelixKrueger/TrimGalore/releases/tag/0.6.5. Accessed July 2024.

40. Kolmogorov M, Yuan J, Lin Y, Pevzner PA. Assembly of long, error-prone reads using repeat graphs. *Nature Biotechnology*. 2019; doi: 10.1038/s41587-019-0072-8.

41. Oxford Nanopore Technologies. (2021). Medaka (Version 1.3.3). https://github.com/nanoporetech/medaka/releases/tag/v1.3.3. Accessed July 2024.

42. Walker BJ, Abeel T, Shea T, Priest M, Abouelliel A, Sakthikumar S, et al. Pilon: An Integrated Tool for Comprehensive Microbial Variant Detection and Genome Assembly Improvement. *PLOS ONE*. 2014; doi: 10.1371/journal.pone.0112963.

43. Laetsch DR, Blaxter ML. BlobTools: Interrogation of genome assemblies. *F1000Res*. 2017; doi: 10.12688/f1000research.12232.1.

44. Buchfink B, Xie C, Huson DH. Fast and sensitive protein alignment using DIAMOND. *Nat Methods*. 2015; doi: 10.1038/nmeth.3176.

45. The UniProt Consortium. UniProt: the universal protein knowledgebase in 2021. *Nucleic Acids Research*. 2021; doi: 10.1093/nar/gkaa1100.

46. Guan D, McCarthy SA, Wood J, Howe K, Wang Y, Durbin R. Identifying and removing haplotypic duplication in primary genome assemblies. *Bioinformatics*. 2020; doi: 10.1093/bioinformatics/btaa025.

47. Meng G, Li Y, Yang C, Liu S. MitoZ: a toolkit for animal mitochondrial genome assembly, annotation and visualization. *Nucleic Acids Research*. 2019; doi: 10.1093/nar/gkz173.

48. Li H, Handsaker B, Wysoker A, Fennell T, Ruan J, Homer N, et al. The Sequence Alignment/Map format and SAMtools. *Bioinformatics*. 2009; doi: 10.1093/bioinformatics/btp352.

49. Alonge M, Lebeigle L, Kirsche M, Jenike K, Ou S, Aganezov S, et al. Automated assembly scaffolding using RagTag elevates a new tomato system for high-throughput genome editing. Genome Biology. 2022; doi: 10.1186/s13059-022-02823-7.

50. Gurevich A, Saveliev V, Vyahhi N, Tesler G. QUAST: quality assessment tool for genome assemblies. *Bioinformatics*. 2013; doi: 10.1093/bioinformatics/btt086.

51. Simão FA, Waterhouse RM, Ioannidis P, Kriventseva EV, Zdobnov EM. BUSCO: assessing genome assembly and annotation completeness with single-copy orthologs. *Bioinformatics*. 2015; doi: 10.1093/bioinformatics/btv351.

52. Mapleson D, Garcia Accinelli G, Kettleborough G, Wright J, Clavijo BJ. KAT: a K-mer analysis toolkit to quality control NGS datasets and genome assemblies. *Bioinformatics*. 2017; doi: 10.1093/bioinformatics/btw663.

53. Rhie A, Walenz BP, Koren S, Phillippy AM. Merqury: reference-free quality, completeness, and phasing assessment for genome assemblies. *Genome Biology*. 2020; doi: 10.1186/s13059-020-02134-9.

54. Wang Z, Pascual-Anaya J, Zadissa A, Li W, Niimura Y, Huang Z, et al. The draft genomes of soft-shell turtle and green sea turtle yield insights into the development and evolution of the turtle-specific body plan. *Nature Genetics*. 2013; doi: 10.1038/ng.2615.

55. Flynn JM, Hubley R, Goubert C, Rosen J, Clark AG, Feschotte C, et al.. RepeatModeler2 for automated genomic discovery of transposable element families. *Proceedings of the National Academy of Sciences*. Proceedings of the National Academy of Sciences. 2020; doi: 10.1073/pnas.1921046117.

56. Abrusán G, Grundmann N, DeMester L, Makalowski W. TEclass—a tool for automated classification of unknown eukaryotic transposable elements. *Bioinformatics*. 2009; doi: 10.1093/bioinformatics/btp084.

57. Smit AFA, Hubley R, Green P. (2022). RepeatMasker (Version 4.1.4). http://www.repeatmasker.org. Accessed July 2024.

58. Leinonen R, Sugawara H, Shumway M. The Sequence Read Archive. *Nucleic Acids Res*. 2011; doi: 10.1093/nar/gkq1019.

59. Banerjee SM, Stoll JA, Allen CD, Lynch JM, Harris HS, Kenyon L, et al. Species and population specific gene expression in blood transcriptomes of marine turtles. *BMC Genomics*. 2021; doi: 10.1186/s12864-021-07656-5.

60. Chow JC, Anderson PE, Shedlock AM. Sea Turtle Population Genomic Discovery: Global and Locus-Specific Signatures of Polymorphism, Selection, and Adaptive Potential. *Genome Biology and Evolution*. 2019; doi: 10.1093/gbe/evz190.

61. Hernández-Fernández J, Pinzón Velasco AM, López Barrera EA, Rodríguez Becerra MDP, Villanueva-Cañas JL, Alba MM, et al.. De novo assembly and functional annotation of blood transcriptome of loggerhead turtle, and in silico characterization of peroxiredoxins and thioredoxins. *PeerJ*. 2021; doi: 10.7717/peerj.12395.

62. Bolger AM, Lohse M, Usadel B. Trimmomatic: a flexible trimmer for Illumina sequence data. *Bioinformatics*. 2014; doi: 10.1093/bioinformatics/btu170.

63. Dobin A, Davis CA, Schlesinger F, Drenkow J, Zaleski C, Jha S, et al.. STAR: ultrafast universal RNA-seq aligner. *Bioinformatics*. 2013; doi: 10.1093/bioinformatics/bts635.

64. Hoff KJ, Lange S, Lomsadze A, Borodovsky M, Stanke M. BRAKER1: Unsupervised RNA-Seq-Based Genome Annotation with GeneMark-ET and AUGUSTUS. *Bioinformatics*. 2016; doi: 10.1093/bioinformatics/btv661.

65. Venturini L, Caim S, Kaithakottil GG, Mapleson DL, Swarbreck D. Leveraging multiple transcriptome assembly methods for improved gene structure annotation. *GigaScience*. 2018; doi: 10.1093/gigascience/giy093.

66. Grabherr MG, Haas BJ, Yassour M, Levin JZ, Thompson DA, Amit I, et al.. Trinity: reconstructing a full-length transcriptome without a genome from RNA-Seq data. *Nat Biotechnol*. 2011; doi: 10.1038/nbt.1883.

67. Wu TD, Watanabe CK. GMAP: a genomic mapping and alignment program for mRNA and EST sequences. *Bioinformatics*. 2005; doi: 10.1093/bioinformatics/bti310.

68. Mapleson D, Venturini L, Kaithakottil G, Swarbreck D. Efficient and accurate detection of splice junctions from RNA-seq with Portcullis. *GigaScience*. 2018; doi: 10.1093/gigascience/giy131.

69. Haas BJ. (2018). TransDecoder (Version 5.5.0). https://github.com/TransDecoder/TransDecoder/releases/tag/TransDecoder-v5.5.0. Accessed July 2024.

70. Haas BJ, Salzberg SL, Zhu W, Pertea M, Allen JE, Orvis J, et al. Automated eukaryotic gene structure annotation using EVidenceModeler and the Program to Assemble Spliced Alignments. *Genome Biol*. 2008; doi: 10.1186/gb-2008-9-1-r7.

71. Dainat J. (2022). AGAT: Another Gff Analysis Toolkit to handle annotations in any GTF/GFF format (Version 0.9.1). *Zenodo*. 10.5281/zenodo.6488306.

72. Pertea G, Pertea M. GFF Utilities: GffRead and GffCompare. *F1000Res*. 2020; doi: 10.12688/f1000research.23297.2.

73. Jones P, Binns D, Chang H-Y, Fraser M, Li W, McAnulla C, et al.. InterProScan 5: genome-scale protein function classification. *Bioinformatics*. 2014; doi: 10.1093/bioinformatics/btu031.

74. Cantarel BL, Korf I, Robb SMC, Parra G, Ross E, Moore B, et al.. MAKER: An easy-to-use annotation pipeline designed for emerging model organism genomes. *Genome Res*. 2008; doi: 10.1101/gr.6743907.

75. Klughammer J, Romanovskaia D, Nemc A, Posautz A, Seid CA, Schuster LC, et al. Comparative analysis of genome-scale, base-resolution DNA methylation profiles across 580 animal species. *Nat Commun*. 2023; doi: 10.1038/s41467-022-34828-y.

76. Oxford Nanopore Technologies. (2023). ModKit (Version 0.1.9). https://github.com/nanoporetech/modkit/releases/tag/v0.1.9. Accessed July 2024.

77. Krueger F, Andrews SR. Bismark: a flexible aligner and methylation caller for Bisulfite-Seq applications. *Bioinformatics*. 2011; doi: 10.1093/bioinformatics/btr167.

78. Cristofari R. merge_CpG.py. https://github.com/rcristofari/penguin-tools/blob/master/merge_CpG.py. Accessed April 2023.

79. Akalin A, Kormaksson M, Li S, Garrett-Bakelman FE, Figueroa ME, Melnick A, et al. methylKit: a comprehensive R package for the analysis of genome-wide DNA methylation profiles. *Genome Biology*. 2012; doi: 10.1186/gb-2012-13-10-r87.

80. Wreczycka K, Gosdschan A, Yusuf D, Grüning B, Assenov Y, Akalin A. Strategies for analyzing bisulfite sequencing data. *Journal of Biotechnology*. 2017; doi: 10.1016/j.jbiotec.2017.08.007.

81. Jones PA. Functions of DNA methylation: islands, start sites, gene bodies and beyond. *Nature Reviews Genetics*. 2012; doi: 10.1038/nrg3230.

82. Akalin A, Franke V, Vlahoviček K, Mason CE, Schübeler D. genomation: a toolkit to summarize, annotate and visualize genomic intervals. *Bioinformatics*. 2015; doi: 10.1093/bioinformatics/btu775.

83. Lawrence M, Huber W, Pagès H, Aboyoun P, Carlson M, Gentleman R, et al. Software for Computing and Annotating Genomic Ranges. *PLOS Computational Biology*. Public Library of Science. 2013; doi: 10.1371/journal.pcbi.1003118.

84. Heckwolf MJ, Meyer BS, Häsler R, Höppner MP, Eizaguirre C, Reusch TBH. Two different epigenetic information channels in wild three-spined sticklebacks are involved

in salinity adaptation. *Science Advances*. American Association for the Advancement of Science. 2020; doi: 10.1126/sciadv.aaz1138.

85. Sagonas K, Meyer BS, Kaufmann J, Lenz TL, Häsler R, Eizaguirre C. Experimental Parasite Infection Causes Genome-Wide Changes in DNA Methylation. *Molecular Biology and Evolution*. 2020; doi: 10.1093/molbev/msaa084.

86. R Core Team. R: A Language and Environment for Statistical Computing. Vienna, Austria: R Foundation for Statistical Computing; 2021. https://www.R-project.org/. Accessed December 2022.

87. Wickham H. ggplot2: Elegant Graphics for Data Analysis. New York, NY: Springer. 2016. https://doi.org/10.1007/978-3-319-24277-4

88. Li H. Minimap2: pairwise alignment for nucleotide sequences. *Bioinformatics*. 2018; doi: 10.1093/bioinformatics/bty191.

89. Cabanettes F, Klopp C. D-GENIES: dot plot large genomes in an interactive, efficient and simple way. *PeerJ*. 2018; doi: 10.7717/peerj.4958.

90. Li H, Durbin R. Inference of Human Population History From Whole Genome Sequence of A Single Individual. *Nature*. 2011; doi: 10.1038/nature10231.

91. Morin PA, Archer FI, Avila CD, Balacco JR, Bukhman YV, Chow W, et al. Reference genome and demographic history of the most endangered marine mammal, the vaquita. *Molecular Ecology Resources*. 2021; doi: 10.1111/1755-0998.13284.

92. Vilaça ST, Piccinno R, Rota-Stabelli O, Gabrielli M, Benazzo A, Matschiner M, et al.. Divergence and hybridization in sea turtles: Inferences from genome data show evidence of ancient gene flow between species. *Mol Ecol*. 2021; doi: 10.1111/mec.16113.

93. Li H. Aligning sequence reads, clone sequences and assembly contigs with BWA-MEM. *arXiv*. 2013; 10.48550/arXiv.1303.3997.

94. Broad Institute. Picard (Version 2.26.9). https://broadinstitute.github.io/picard. Accessed January 2022.

95. Danecek P, Bonfield JK, Liddle J, Marshall J, Ohan V, Pollard MO, et al. Twelve years of SAMtools and BCFtools. *GigaScience*. 2021; doi: 10.1093/gigascience/giab008.

96. Piovano S, Clusa M, Carreras C, Giacoma C, Pascual M, Cardona L. Different growth rates between loggerhead sea turtles (Caretta caretta) of Mediterranean and Atlantic origin in the Mediterranean Sea. *Marine Biology*. 2011; doi: 10.1007/s00227-011-1759-7.

97. Hansen J, Sato M, Russell G, Kharecha P. Climate sensitivity, sea level and atmospheric carbon dioxide. *Philosophical Transactions: Mathematical, Physical and Engineering Sciences*. The Royal Society; 371:1–312013; 10.1098/rsta.2012.0294.

98. Clark PU, Shakun JD, Rosenthal Y, Köhler P, Bartlein PJ. Global and regional temperature change over the past 4.5 million years. *Science*. American Association for the Advancement of Science. 2024; doi: 10.1126/science.adi1908.

99. Robinson JA, Räikkönen J, Vucetich LM, Vucetich JA, Peterson RO, Lohmueller KE, et al. Genomic signatures of extensive inbreeding in Isle Royale wolves, a population on the threshold of extinction. *Science Advances*. American Association for the Advancement of Science; 2019; doi: 10.1126/sciadv.aau0757.

100. McKenna A, Hanna M, Banks E, Sivachenko A, Cibulskis K, Kernytsky A, et al. The Genome Analysis Toolkit: A MapReduce framework for analyzing next-generation DNA sequencing data. *Genome Res*. 2010; doi: 10.1101/gr.107524.110.

101. Martin SH. (2016). popgenWindows. https://github.com/simonhmartin/genomics_general/blob/master/popgenWindows.py. Accessed May 2022.

102. Machado CRD, Domit C, Pucci MB, Gazolla CB, Glugoski L, Nogaroto V, et al. Heterochromatin and microsatellites detection in karyotypes of four sea turtle species: Interspecific chromosomal differences. *Genet Mol Biol*. 2020. doi: 10.1590/1678-4685-GMB-2020-0213.

103. Altschul SF, Gish W, Miller W, Myers EW, Lipman DJ. Basic local alignment search tool. *Journal of Molecular Biology*. 1990; doi: 10.1016/S0022-2836(05)80360-2.

104. Gu Z, Gu L, Eils R, Schlesner M, Brors B. circlize implements and enhances circular visualization in R. *Bioinformatics*. 2014; doi: 10.1093/bioinformatics/btu393.

105. Emms DM, Kelly S. OrthoFinder: solving fundamental biases in whole genome comparisons dramatically improves orthogroup inference accuracy. *Genome Biology*. 2015; doi: 10.1186/s13059-015-0721-2.

106. Szklarczyk D, Gable AL, Nastou KC, Lyon D, Kirsch R, Pyysalo S, et al.. The STRING database in 2021: customizable protein–protein networks, and functional characterization of user-uploaded gene/measurement sets. *Nucleic Acids Res*. 2020; doi: 10.1093/nar/gkaa1074.

107. Guigó R. Genome annotation: From human genetics to biodiversity genomics. Cell Genomics. Elsevier; 2023; doi: 10.1016/j.xgen.2023.100375.

108. Olova N, Krueger F, Andrews S, Oxley D, et al. Comparison of whole-genome bisulfite sequencing library preparation strategies identifies sources of biases affecting DNA methylation data. *Genome Biology*. 2018; doi: 10.1186/s13059-018-1408-2.

109. Suzuki MM, Bird A. DNA methylation landscapes: provocative insights from epigenomics. *Nat Rev Genet*. 2008; doi: 10.1038/nrg2341.

110. Liu Y, Rosikiewicz W, Pan Z, Jillette N, Wang P, Taghbalout A, et al.. DNA methylation-calling tools for Oxford Nanopore sequencing: a survey and human epigenome-wide evaluation. *Genome Biology*. 2021; doi: 10.1186/s13059-021-02510-z.

111. Liu X, Ni Y, Wang D, Ye S, Yang M, Sun X, et al.. Unraveling the whole genome DNA methylation profile of zebrafish kidney marrow by Oxford Nanopore sequencing. Sci Data. 2023; doi: 10.1038/s41597-023-02431-5.

112. Gombert S, Jahn K, Pathak H, Burkert A, Schmidt G, Wiehlmann L, et al.. Comparison of methylation estimates obtained via MinION nanopore sequencing and sanger bisulfite sequencing in the TRPA1 promoter region. BMC Medical Genomics. 2023; doi: 10.1186/s12920-023-01694-6.

113. Sigurpalsdottir BD, Stefansson OA, Holley G, Beyter D, Zink F, Hardarson MÞ, et al.. A comparison of methods for detecting DNA methylation from long-read sequencing of human genomes. Genome Biology. 2024; doi: 10.1186/s13059-024-03207-9.

114. Ni Y, Liu X, Simeneh ZM, Yang M, Li R. Benchmarking of Nanopore R10.4 and R9.4.1 flow cells in single-cell whole-genome amplification and whole-genome shotgun sequencing. Comput Struct Biotechnol J. 2023; doi: 10.1016/j.csbj.2023.03.038.

115. Simpson JT, Workman RE, Zuzarte PC, David M, Dursi LJ, Timp W. Detecting DNA cytosine methylation using nanopore sequencing. 2017; *Nat Methods*. doi: 10.1038/nmeth.4184.

116. Xu L, Seki M. Recent advances in the detection of base modifications using the Nanopore sequencer. *Journal of Human Genetics*. 2020; doi: 10.1038/s10038-019-0679-0.

117. Shen L, Zhang Y. 5-Hydroxymethylcytosine: generation, fate, and genomic distribution. *Curr Opin Cell Biol*. 2013; doi: 10.1016/j.ceb.2013.02.017.

118. Martínez-Botí MA, Foster GL, Chalk TB, Rohling EJ, Sexton PF, Lunt DJ, et al. Plio-Pleistocene climate sensitivity evaluated using high-resolution CO2 records. *Nature*. 2015; doi: 10.1038/nature14145.

119. McClymont EL, Ho SL, Ford HL, Bailey I, Berke MA, Bolton CT, et al. Climate Evolution Through the Onset and Intensification of Northern Hemisphere Glaciation. *Reviews of Geophysics*. 2023; doi: 10.1029/2022RG000793.

120. Ford HL, Chalk TB. SIDEBAR. The Mid-Pleistocene Enigma. *Oceanography*. 2020; doi: 10.5670/oceanog.2020.216.

121. Lawrence KT, Sigman DM, Herbert TD, Riihimaki CA, Bolton CT, Martinez-Garcia A, et al. Time-transgressive North Atlantic productivity changes upon Northern Hemisphere glaciation. *Paleoceanography*. 2013; doi: 10.1002/2013PA002546.

122. Lamy F, Winckler G, Arz HW, Farmer JR, Gottschalk J, Lembke-Jene L, et al. Five million years of Antarctic Circumpolar Current strength variability. *Nature*. 2024; doi: 10.1038/s41586-024-07143-3.

123. Baltazar-Soares M, Klein JD, Correia SM, Reischig T, Taxonera A, Roque SM, et al. Distribution of genetic diversity reveals colonization patterns and philopatry of the

loggerhead sea turtles across geographic scales. *Scientific Reports*. 2020; doi: 10.1038/s41598-020-74141-6.

124. McQueen HA, Fantes J, Cross SH, Clark VH, Archibald AL, Bird AP. CpG islands of chicken are concentrated on microchromosomes. *Nat Genet*. 1996; doi: 10.1038/ng0396-321.

125. Waters PD, Patel HR, Ruiz-Herrera A, Álvarez-González L, Lister NC, Simakov O, et al. Microchromosomes are building blocks of bird, reptile, and mammal chromosomes. *Proceedings of the National Academy of Sciences*. 2021; doi: 10.1073/pnas.2112494118.

126. Papin C, Le Gras S, Ibrahim A, Salem H, Karimi MM, Stoll I, et al. CpG Islands Shape the Epigenome Landscape. *Journal of Molecular Biology*. 2021; doi: 10.1016/j.jmb.2020.09.018.

127. Ziller MJ, Gu H, Müller F, Donaghey J, Tsai LT-Y, Kohlbacher O, et al. Charting a dynamic DNA methylation landscape of the human genome. *Nature*. 2013; doi: 10.1038/nature12433.

128. Hoelzel AR, Bruford MW, Fleischer RC. Conservation of adaptive potential and functional diversity. *Conserv Genet*. 2019; doi: 10.1007/s10592-019-01151-x.

129. Harewood L, Fraser P. The impact of chromosomal rearrangements on regulation of gene expression. *Human Molecular Genetics*. 2014; doi: 10.1093/hmg/ddu278.

130. Valenzuela N, Adams DC. CHROMOSOME NUMBER AND SEX DETERMINATION COEVOLVE IN TURTLES. *Evolution*. 2011; doi: 10.1111/j.1558-5646.2011.01258.x.

131. Lee L, Montiel EE, Navarro-Domínguez BM, Valenzuela N. Chromosomal Rearrangements during Turtle Evolution Altered the Synteny of Genes Involved in Vertebrate Sex Determination. *Cytogenet Genome Res*. 2019; doi: 10.1159/000497302.

132. Schroeder AL, Metzger KJ, Miller A, Rhen T. A Novel Candidate Gene for Temperature-Dependent Sex Determination in the Common Snapping Turtle. *Genetics*. 2016; doi: 10.1534/genetics.115.182840.

133. Elango N, Yi SV. DNA Methylation and Structural and Functional Bimodality of Vertebrate Promoters. *Molecular Biology and Evolution*. 2008; doi: 10.1093/molbev/msn110.

134. Keller TE, Han P, Yi SV. Evolutionary Transition of Promoter and Gene Body DNA Methylation across Invertebrate–Vertebrate Boundary. *Mol Biol Evol*. 2016; doi: 10.1093/molbev/msv345.

135. De Paoli-Iseppi R, Deagle BE, McMahon CR, Hindell MA, Dickinson JL, Jarman SN. Measuring Animal Age with DNA Methylation: From Humans to Wild Animals. *Front Genet*. Frontiers. 2017; doi: 10.3389/fgene.2017.00106.

136. Viitaniemi HM, Verhagen I, Visser ME, Honkela A, van Oers K, Husby A. Seasonal Variation in Genome-Wide DNA Methylation Patterns and the Onset of Seasonal Timing of Reproduction in Great Tits. *Genome Biology and Evolution*. 2019; doi: 10.1093/gbe/evz044.

137. Grant OA, Wang Y, Kumari M, Zabet NR, Schalkwyk L. Characterising sex differences of autosomal DNA methylation in whole blood using the Illumina EPIC array. *Clinical Epigenetics*. 2022; doi: 10.1186/s13148-022-01279-7.

138. Yousefi PD, Suderman M, Langdon R, Whitehurst O, Davey Smith G, Relton CL. DNA methylation-based predictors of health: applications and statistical considerations. *Nat Rev Genet*. 2022; doi: 10.1038/s41576-022-00465-w.

139. Xu R, Li S, Guo S, Zhao Q, Abramson MJ, Li S, et al. Environmental temperature and human epigenetic modifications: A systematic review. *Environmental Pollution*. 2020; doi: 10.1016/j.envpol.2019.113840.

140. Mäkinen H, Van Oers K, Eeva T, Ruuskanen S. The effect of experimental lead pollution on DNA methylation in a wild bird population. *Epigenetics*. 2022. doi: 10.1080/15592294.2021.1943863.

141. Yen EC, Gilbert JD, Balard A, Afonso IO, Fairweather K, Newlands D, et al. DNA methylation carries signatures of sublethal effects under thermal stress in loggerhead sea turtles. *Evolutionary Applications*. 2024; doi: 10.1111/eva.70013.

142. Bock SL, Smaga CR, McCoy JA, Parrott BB. Genome-wide DNA methylation patterns harbour signatures of hatchling sex and past incubation temperature in a species with environmental sex determination. Molecular Ecology. 2022; doi: 10.1111/mec.16670.

143. Mork L, Capel B. Conserved action of β-catenin during female fate determination in the red-eared slider turtle. *Evolution & Development*. 2013; doi: 10.1111/ede.12020.

144. Liu J, Xiao Q, Xiao J, Niu C, Li Y, Zhang X, et al.. Wnt/β-catenin signalling: function, biological mechanisms, and therapeutic opportunities. *Sig Transduct Target Ther*. 2022; doi: 10.1038/s41392-021-00762-6.

145. Tezak B, Straková B, Fullard DJ, Dupont S, McKey J, Weber C, et al.. Higher temperatures directly increase germ cell number, promoting feminization of red-eared slider turtles. *Current Biology*. 2023; doi: 10.1016/j.cub.2023.06.008.

146. Rhen T, Schroeder A. Molecular Mechanisms of Sex Determination in Reptiles. *SXD*. Karger Publishers. 2010; doi: 10.1159/000282495.

147. Kent J, Wheatley SC, Andrews JE, Sinclair AH, Koopman P. A male-specific role for SOX9 in vertebrate sex determination. *Development*. 1996; doi: 10.1242/dev.122.9.2813.

148. Moreno-Mendoza N, Harley VR, Merchant-Larios H. Temperature Regulates SOX9 Expression in Cultured Gonads of *Lepidochelys olivacea*, a Species with Temperature Sex Determination. *Developmental Biology*. 2001; doi: 10.1006/dbio.2000.9952.

149. Kohno S, Katsu Y, Urushitani H, Ohta Y, Iguchi T, Guillette Jr LJ. Potential Contributions of Heat Shock Proteins to Temperature-Dependent Sex Determination in the American Alligator. *Sexual Development*. 2009; doi: 10.1159/000260374.

150. Capel B. Vertebrate sex determination: evolutionary plasticity of a fundamental switch. Nat Rev Genet. 2017; doi: 10.1038/nrg.2017.60.

151. Yen EC. (2024). Article_CarCar_GenomeAssembly. https://github.com/eugeniecyen/Article_CarCar_GenomeAssembly.

152. Yen EC, Gilbert JD, Balard A, Taxonera A, Fairweather K, Ford HL, et al. Supporting data for "Chromosome-level genome assembly and methylome profile yield insights for the conservation of endangered loggerhead sea turtles" GigaScience Database. 2025. https://doi.org/10.5524/102690

Figure 1

Click here to access/download;Figure;Fig1_Rev1.png

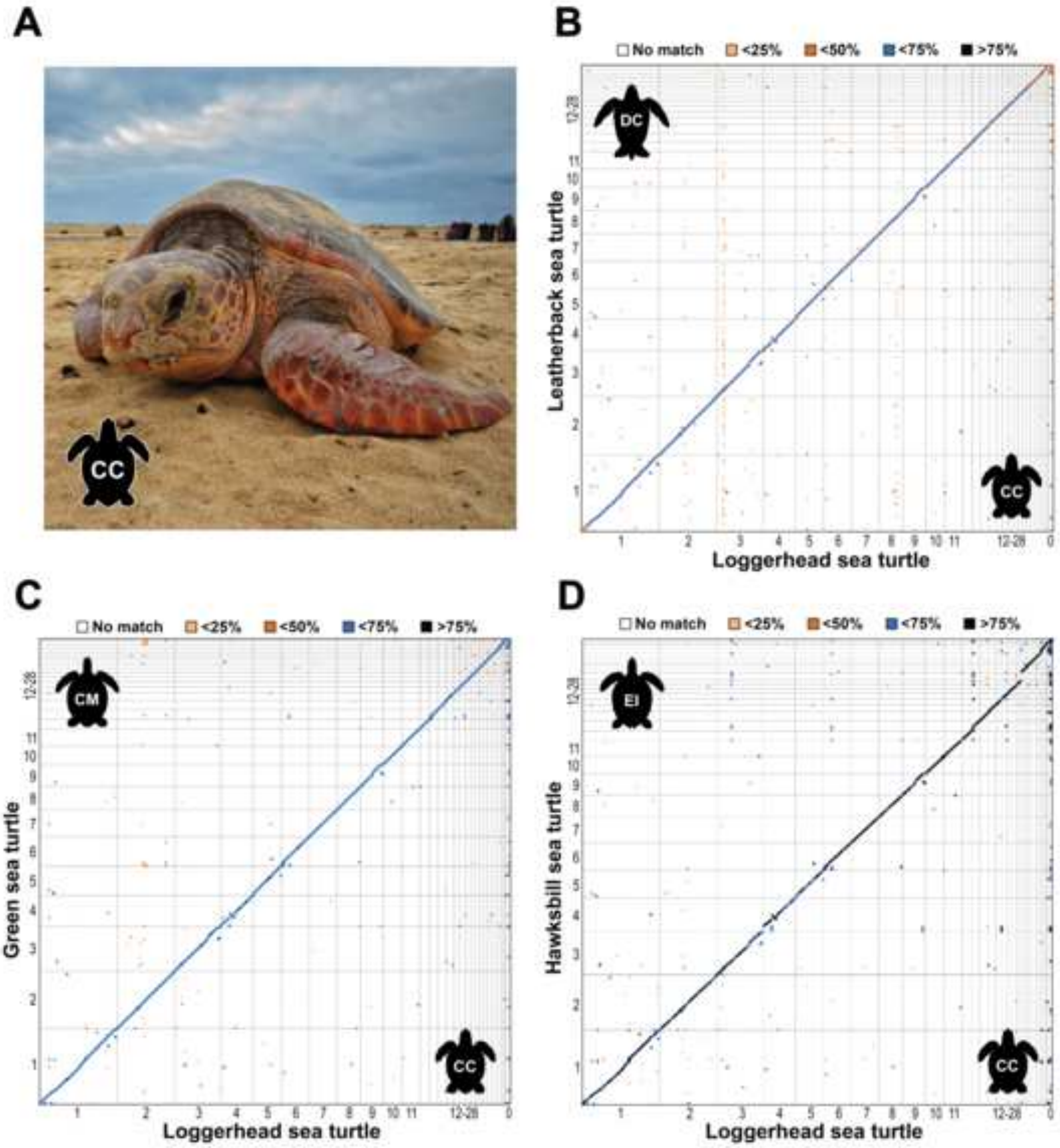

Figure 2

Click here to access/download;Figure;Fig2_Rev1.png

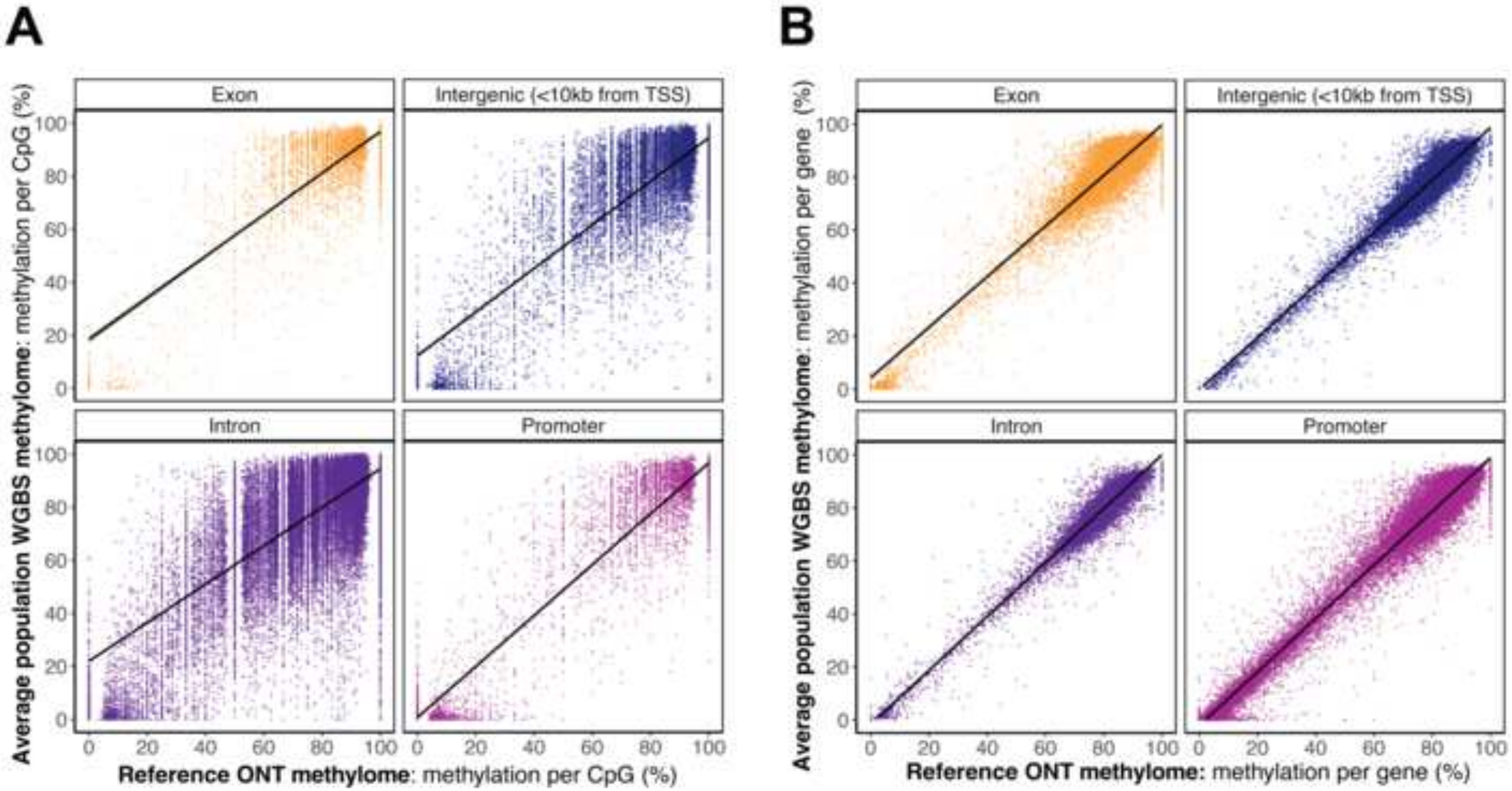

Figure 3

Click here to access/download;Figure;Fig3-Rev.png

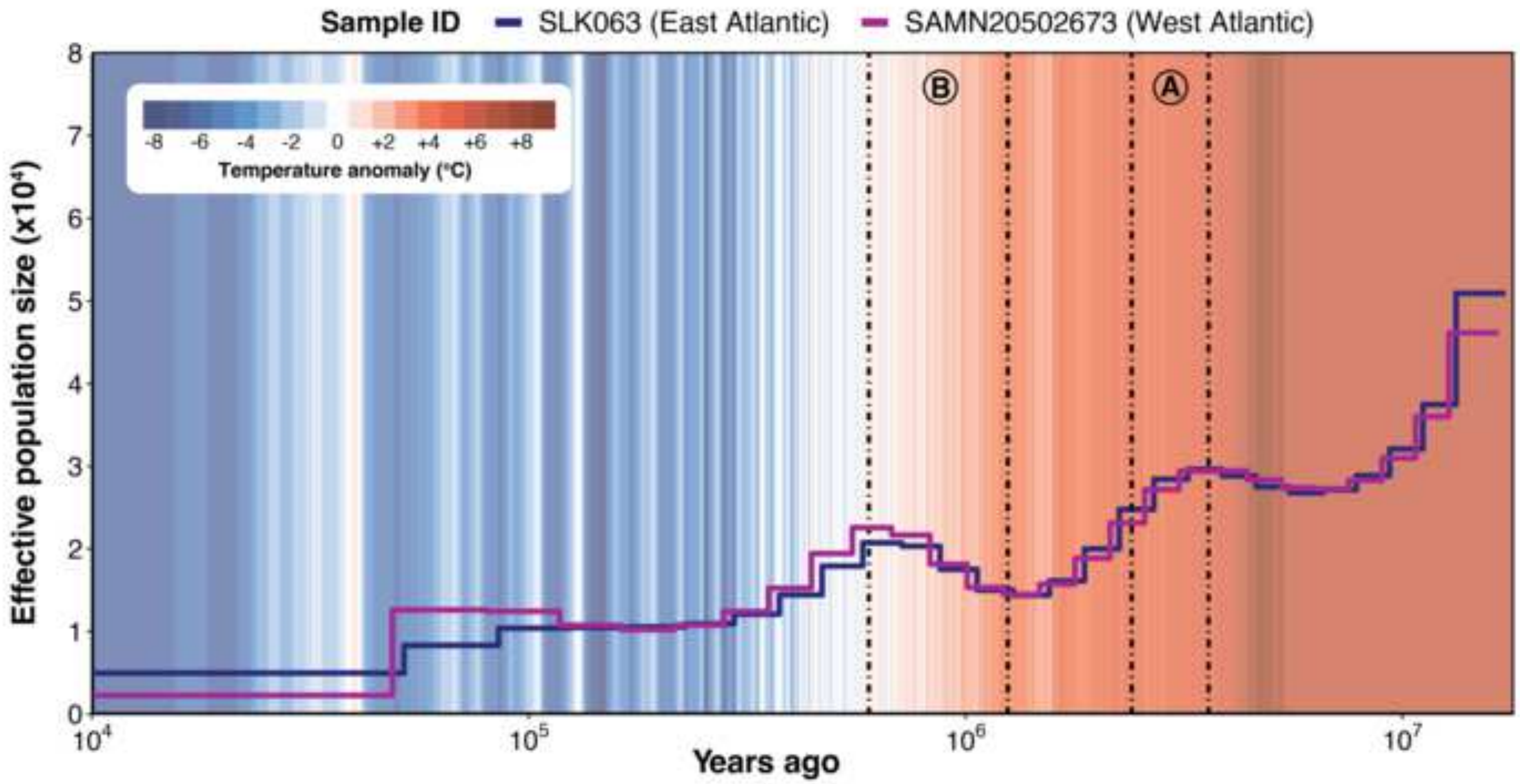

Click here to access/download;Figure;Fig4_Rev.png

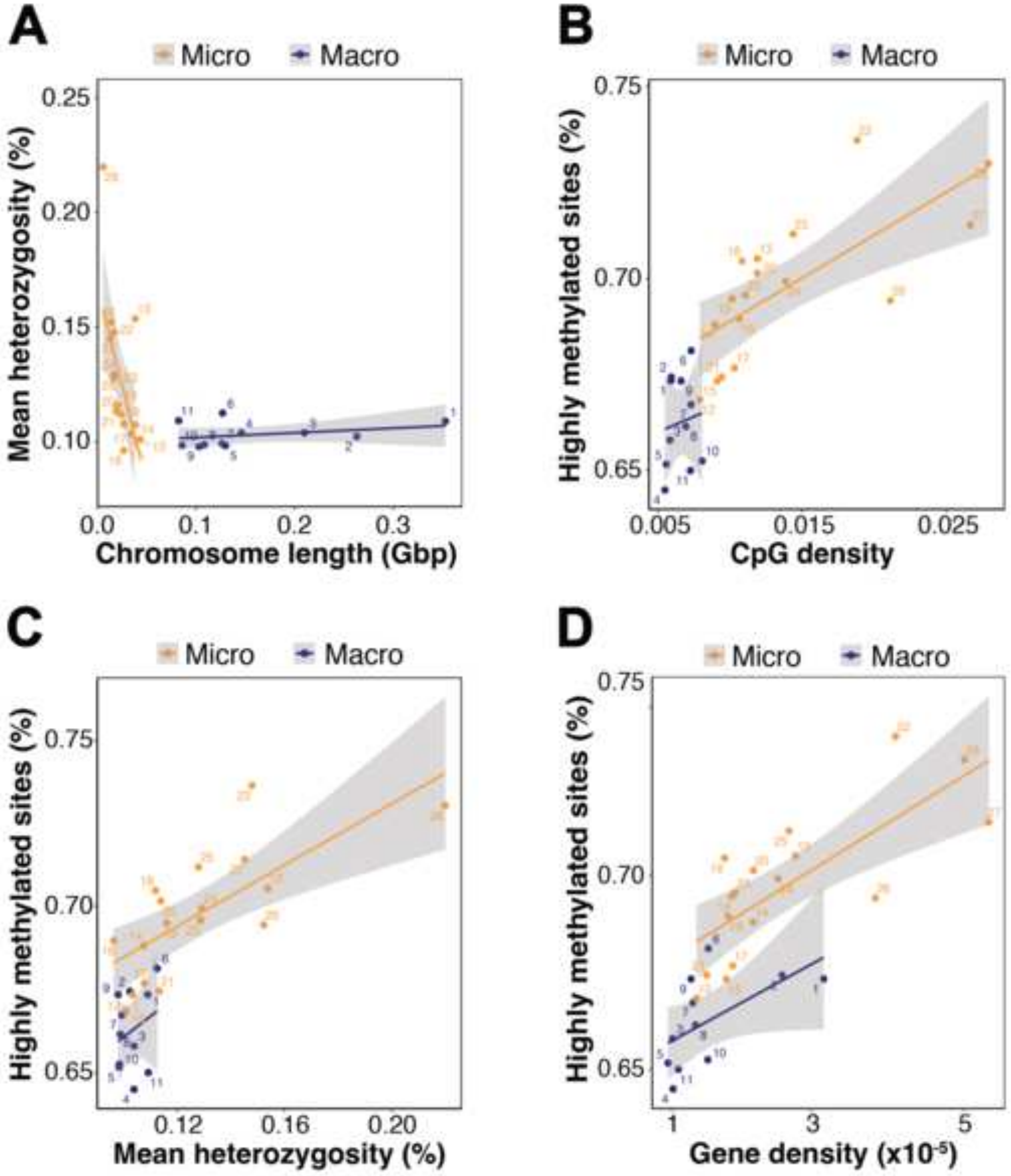

Figure 5 Click here to access/download;Figure;Fig5_Rev1.png

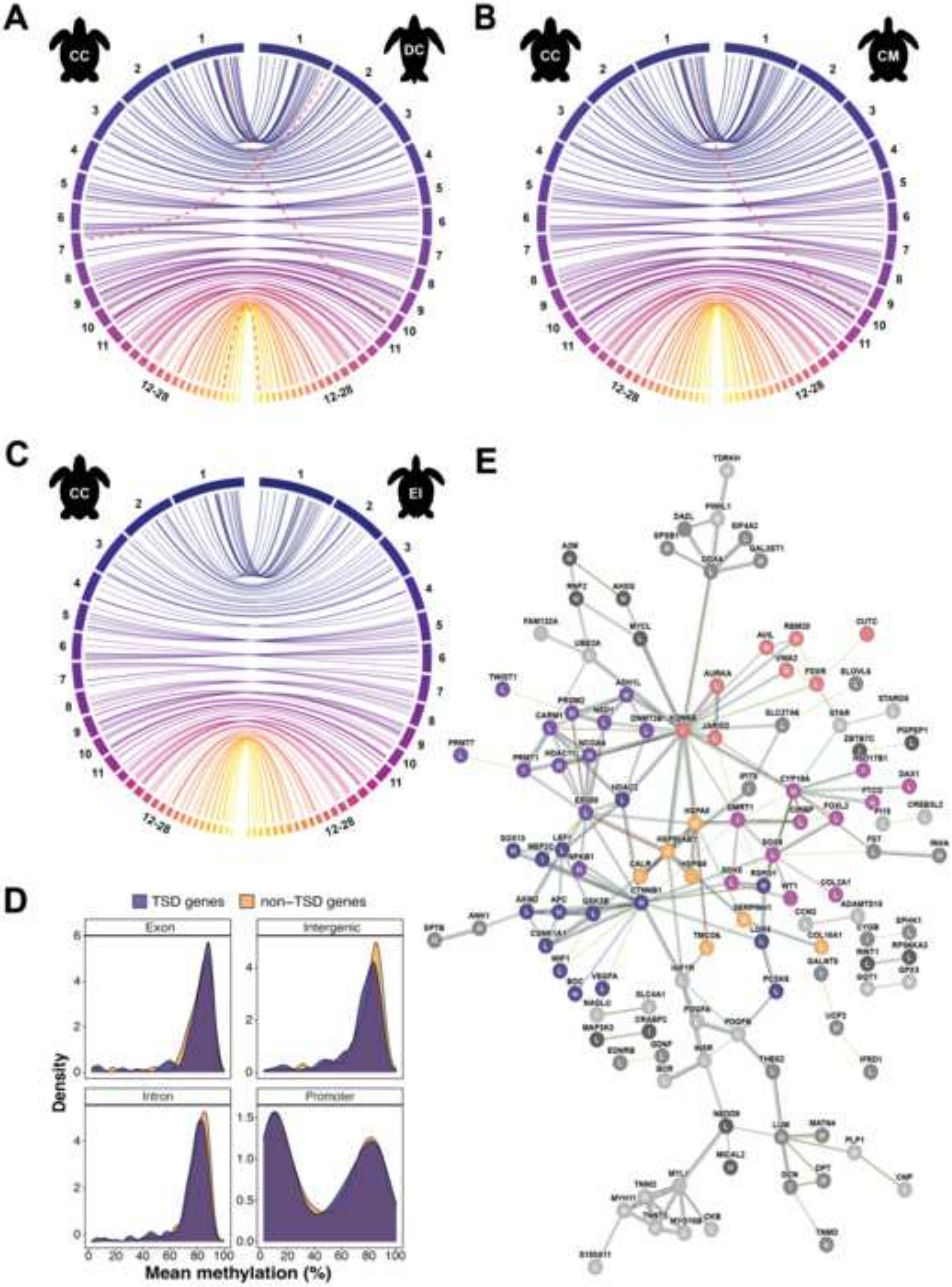

Click here to access/download
Supplementary Material
Yen2024_Genome_Supp_Material_Rev2.docx

Cover and response to Reviewers
Click here to access/download;Personal Cover;Yen2024_Genome_Response_To_Reviewers_Rev1.doc

Ms Eugenie C. Yen
School of Biological and Chemical Sciences
Queen Mary University of London
Fogg Building, Mile End Road,
London, E1 4NS, UK
e.yen@qmul.ac.uk

Date: 18th February 2024

Dear Editors,

Thank you for providing us with the opportunity to submit a revised version of our manuscript GIGA-D-24-00386 “**Chromosome-level genome assembly and methylome profile yield insights for the conservation of endangered loggerhead sea turtles** for publication as a Data Note in *GigaScience*.

We thank the Editorial Team and reviewers for their positive assessment of our manuscript. We have taken all their comments onboard and addressed them thoroughly in our point-by-point response provided below in blue.

All authors approved this submission. Please, let me know if you require any additional information.

Best regards,

Eugenie C. Yen (on behalf of all authors)

Editor

Dear Miss Yen,

Your manuscript "Chromosome-level genome assembly and methylome profile yield insights for the conservation of endangered loggerhead sea turtles" (GIGA-D-24-00386) has been assessed by our reviewers. Although it is of interest, we are unable to consider it for publication in its current form. The reviewers have raised a number of points which we believe would improve the manuscript and may allow a revised version to be published in GigaScience.

Their reports, together with any other comments, are below. Please also take a moment to check our website at https://www.editorialmanager.com/giga/ for any additional comments that were saved as attachments.

If you are able to fully address these points, we would encourage you to submit a revised manuscript to GigaScience. Once you have made the necessary corrections, please submit online at: https://www.editorialmanager.com/giga/

If you have forgotten your username or password please use the "Send Login Details" link to get your login information. For security reasons, your password will be reset.

Please include a point-by-point within the 'Response to Reviewers' box in the submission system. Please ensure you describe additional experiments that were carried out and include a detailed rebuttal of any criticisms or requested revisions that you disagreed with. Please also ensure that your revised manuscript conforms to the journal style, which can be found in the Instructions for Authors on the journal homepage. If the data and code has been modified in the revision process please be sure to update the public versions of this too.

The due date for submitting the revised version of your article is 10 Jan 2025.

I look forward to receiving your revised manuscript soon.

Best wishes,

Hongfang Zhang

GigaScience

www.gigasciencejournal.com

We thank the Associate Editor and the reviewers for their positive assessment of our manuscript. We have taken all comments onboard and addressed them thoroughly. Our point-by-point responses are provided in blue, alongside a revised manuscript with tracked changes. Line numbers refer to the document version in Simple Mark-Up/No Markup mode.

Following the suggestions, we have improved the genome annotation, added the hawksbill genome to broaden comparisons across sea turtle species, and added individual-level and CpG site-level comparisons between the ONT methylome and the average WGBS population methylome. We have further clarified the key points highlighted by our methylation results, namely better articulating the caveats and advantages of offering broad-level, blood methylation patterns from an adult female.

Overall, we thank the reviewers for their constructive and insightful suggestions that have helped to improve our manuscript.

Reviewer #1

The study presents high-quality genomic and methylomic data for loggerhead sea turtles, serving as a significant resource for further genomic and epigenomic research on this species. Notably, this is the first methylome derived from a sea turtle using ONT technology, offering a new, reliable method for studying the epigenetic characteristics of non-model organisms. Moreover, by integrating genomic and methylomic data, the authors analyze the functionality and methylation patterns of TSD-related genes, contributing fresh perspectives to the molecular mechanisms underlying TSD. While the study offers valuable data, there are several areas that could be enhanced.

1) Lack of Reference to Hawksbill Turtle Genome: The manuscript does not discuss any information regarding the hawksbill turtle genome. Given that hawksbills also published a comparative analysis of the loggerhead's genomic data, I recommend that the authors include relevant information or clarify why hawksbill data was not considered.

We would like to thank the reviewer for their comment. Internal discussions had occurred surrounding the inclusion of the hawksbill turtle genome, also a member of the Carettini tribe, which resulted in us not presenting comparisons. This was mostly motivated by the emergence of expected patterns when comparing against the more diverged green and leatherback turtle genomes. However, upon the reviewer's feedback, we agree this was not the ideal decision and have therefore included comparisons to the hawksbill genome in the revised manuscript. Note Guo *et al.* (2023) present a direct comparative analysis of the hawksbill vs green turtle genomes. Hence, following the reviewer's nice suggestion, our manuscript now provides the first comparison of loggerhead vs hawksbill genomes.

We added the hawksbill genome reference to **line 57**. In relation to genome assembly quality, we added assembly and annotation statistics for the hawksbill genome to **Table 1, Table S4, Table S7**, and **line 149**. In relation to genome-wide synteny between sea turtle species, we added a whole genome alignment plot of the loggerhead vs hawksbill genome as **Figure 1D**, with associated changes to **Table S10, line 241**, and **lines 419-430**.

Since the hawksbill annotation was less complete than our genome and the other available for the green and the leatherback turtles, we identified 192 TSD-linked genes for comparison against the 199 TSD-linked genes in our loggerhead annotation, as explained on **lines 305-306.** We also added the hawksbill genes to our synteny analysis of TSD-linked genes as **Figure 5C** with associated text additions on **lines 305-307** and **514-515**.

Overall, comparisons of synteny aligned very well with phylogenetic expectations, with the loggerhead vs hawksbill genomes being more similar than vs green and leatherback genomes. Finally, the hawksbill genome was also included during the identification of single copy orthologues between sea turtle species for comparison to TSD-linked genes as background non-TSD-linked genes, updated on **line 314**. This resulted in 11,211 single-copy orthologues for comparisons, as opposed to 15,041 originally.

2) Further Optimization of Genome Annotation: The authors acknowledge that the completeness of the genome annotation requires enhancement and mention future improvements such as species-specific parameter adjustments and manual curation. While it is understandable that time and resource constraints may have limited these optimizations prior to submission, it would be beneficial for the authors to clarify the reasons for this and outline a timeline for future enhancements.

Firstly, we are pleased to share that we have improved our annotation's BUSCO completeness score from 94.7% (S:93.9%, D:0.8%, F:1.1%, M:4.2%) to 95.4% (S:94.4%, D:1.0%, F:1.1%, M:3.5%), achieved by simplifying the BRAKER pipeline and removing the BRAKER2/TSEBRA step. This approach aims to incorporate protein homology-based hints to supplement gene annotation. However, likely due to sub-optimal parameters as previously discussed, this process was not efficient enough for our genome. Reflecting our improved annotation, alterations to the manuscript text can be found on **lines 360-368**, as well as annotation statistics reported in **Table S5-7**. Notably, with these improvements, our annotation is now of intermediate completeness (C: 95.4%) amongst all available chromosome-level sea turtle genomes following the addition of the hawksbill genome with a BUSCO score of 92.3% (S: 91.2%, D:1.1%, F:2.3%, M:5.4%).

We performed annotation to the best of our ability, although it of course does not reach the same level of quality as those produced by the Vertebrate Genomes Project and Canada BioGenome Project in collaboration with NCBI. These large sequencing consortia have in-house annotation specialists, including manual curation experts. We have expanded upon the statement on **line 368** to clarify this in response to the reviewer's suggestion. Nevertheless, our annotation is a valuable resource to publish alongside our genome assembly, and has indeed already been useful for a recently published study from our research lab (Yen *et al.* 2024 Evol Apps).

3) Information on Individual Variability in WGBS Results: The manuscript lacks specific information on inter-individual variability among the ten individuals in the WGBS data. I suggest that the authors consider adding this analysis or provide justification for its absence. If significant variability exists among individuals, averaging the methylomic data could obscure important biological information.

We thank the reviewer for raising this important point. As the goal of this comparison is to assess whether our reference ONT methylome is comparable and thus representative of the reference population via a gold-standard method, we chose to compare against an 'average population WGBS methylome' as it better represents the population-level methylation profile, rather than individual-level noise. However, we acknowledge that supplementing information on inter-individual variability is important to convince readers that this decision is justified.

The mean methylation level across all ten individuals was 75.5 ± 0.75 (SD) % and the proportion of highly methylated CpGs was 75.2 ± 1.58 (SD) %. The low standard deviations demonstrate the genome-wide methylation levels are similar among individuals. We have added these metrics calculated per individual to **Table S3,** with additional reporting of the mean ± SD on **lines 380-381.** We have also added the outputs of linear models and post-hoc Pearson's correlation tests correlating methylation levels from the reference ONT methylome against each of the ten WGBS methylomes separately. Model outputs per individual are reported in the new **Table S9 and S10**, with associated text additions on **lines 399-400**. Overall, results were very similar among individuals, as well as to the 'average population WGBS methylome'.

Overall, the new individual-level comparisons confirm that methylation profiles are similar amongst WGBS individuals, thus justifying our decision to compare against the 'average population WGBS methylome'. We have clarified this logic on **lines 229-232**. Note that other changes have also been implemented throughout this analysis section in response to later reviewer's comments.

4) Clarification on Statistical Tests and Data Processing: The manuscript employs several statistical tests such as t-tests, F-tests, and chi-squared tests. However, the methods section lacks detailed information on how the data was processed for these analyses. I recommend that the authors provide a more thorough explanation of the data preparation steps, assumptions checked, and justification for the choice of tests.

We thank the reviewer for their suggestion. All statistical tests applied followed standard protocols of data visualisation and assumption checking. We have added a general statement to convey this on **line 221-224**. Where a non-parametric alternative test was chosen due to significant violation of the normality assumption, we have added extra details outlining the decision process (**line 284-287).**

Chi-squared tests of independence implicitly met assumptions due to the structure of the datasets (i.e., mutually exclusive categorical variables, independent observations, data in frequencies/counts, values per cell >5 in >80% of cells). Indeed, this test is commonly used in methylation data, with a reference to a previous methylation study that performed the same test (ref 85: Sagonas *et al.*, 2020) as further justification.

In summary, this manuscript makes a significant contribution to the study of loggerhead turtle genomics and methylomics. Addressing the aforementioned points could further enhance the quality and impact of the work.

We would like to thank the reviewer for their helpful and thorough comments which have helped to improve the manuscript.

<u>Reviewer #2:</u>

This work offers a in improved version of the reference genome for the loggerhead sea turtle. The authors have also analyzed the methylation patterns of blood obtained from different individuals and with two methods. The resulting data set includes gene annotations, methylation levels and the specific analysis of methylation levels of genes involved in temperature-dependent sex determination (TSD). While the improvements offered by this work seem modest, I think that the data sets may provide important resources for future works.

-In my opinion, the use of a previous version of the same genome in the assembly process should be noted in the abstract. It would be enough to write "... followed by homolgy-guided scaffolding to GSC_CCare_1.0...".

We would like to thank the review for their comment. It gives us a chance to clarify that our genome assembly is not just an updated version of the same genome, with 'GSC_CCare_1.0' assembled from genomic data of two individuals sampled from an Adriatic Sea rehab centre. Rather, our genome was assembled for a wild nesting loggerhead representing the genetically distinct and globally important Cabo Verde (Northeast Atlantic) nesting aggregation (the largest in the world for this species). Consequently, while we leverage 'GSC_CCare_1.0' for homology-guided scaffolding, the genetic variation reflected in the assembly is different and specific to Cabo Verde. Such population-specific reference genomes are essential due to growing awareness of reference bias, which is particularly relevant in sea turtles due to strong genetic differentiation between populations maintained by philopatry (e.g., Stiebens *et al.*, 2013, Proceedings of the Royal Society B).

In line with focusing on the distinct population-specific offerings of our genome, we decided not to add 'GSC_CCare_1.0' to the abstract, as we did not want to reinforce this link and risk misleading readers that our genome is simply an improvement upon 'GSC_CCare_1.0'. We have instead added "against the same species" on **line 10** of the Abstract to be more explicit on what we performed homology-guided scaffolding against, and emphasised that 'GSC_CCare_1.0' was used in the Methods on **line 127.**

-If possible, the authors should clarify the taxonomic relationship between the reference individual in this work and the reference individual for the previous version of the genome (ref. 26). Is it the same NCBI taxid?

The NCBI TaxID is indeed the same, with both genomes representing the loggerhead sea turtle (*Caretta caretta).* To reiterate this, we have added its NCBI TaxID to **line 58**, as well as for all other mentioned sea turtle species for consistency. The sequenced individuals are however different.

-There is a mention to "lateral terminal repeats" at the "Genome annotation" section (page 7). I think it is a typo and it should read "long terminal repeats".

We thank the reviewer for catching this typo, which has now been corrected on **line 155.**

-In the same section, at page 9, reference 73 refers to StringTie, not gffread. In addition, it is not clear how "in-frame stop codons were removed". A simple way to unambiguously explain this would be to provide the options that were used, as with other programs.

The referencing error has been fixed – thank you to the reviewer for reviewing our manuscript so carefully. We have also added the requested information in relation to in-frame stop codons on **line 181**.

-I would revise the use of "coverage" versus "depth". For instance, the expression "...a coverage of 9.2(...)X" would be more precise as "...a sequencing depth of 9.2(...)X". Coverage should be a fraction or a percentage. However, this is only a piece of advice, as there is no strong consensus at the moment.

We have replaced "coverage" with "sequencing depth" throughout the manuscript.

-The interpretation of methylation patterns is always difficult. In my opinion, the manuscript should discuss several limitations about the results:

We thank the reviewer for drawing attention to the need to refine our interpretation of methylation patterns presented in this manuscript.

*First, using blood as the starting tissue is convenient but not ideal, as many methylation patterns are tissue-specific. The authors may want to add a reference to preliminary evidence that some methylation changes in blood cells are related to TSD (Bock et al., Mol Ecol. 2022; 31:5487-5505).

Indeed, blood cells are not ideal for dissecting the causal, mechanistic roles of DNA methylation in TSD, which are better suited to gonadal studies in laboratory-based systems. However, blood tissue is

extremely useful in the context of endangered wildlife conservation, particularly for cryptic, traits such as sex in sea turtles, which cannot be identified in hatchlings without sacrificing individuals. Blood is particularly well-suited for this conservation goal, as it can be collected via minimally invasive sampling and often represents systemic and cross-tissue correlations.

We chose to profile methylation patterns of biologically relevant TSD-linked genes, as they are often the first set of candidate genes focused upon when looking for methylation-based sex differences in TSD species (e.g., Radhakrishnan *et al.*, 2017 Epigenetics & Chromatin, Anastasiadi *et al.*, 2018 Epigenetics, Valdivieso *et al.*, 2022, Mol Ecol Resources). Hence, we extend broad insights into blood-based patterns to guide future studies searching for biomarkers of sex. TSD-linked genes could host blood-based methylation differences of interest, as methylation marks can persist across life stages and even cellular differentiation events (e.g., Kim *et al.*, 2011 Nature).

Overall, we have modified the text between **lines 529-554** to better highlight the caveats of blood-based insights, as well as its advantages in the context of identifying correlative methylation biomarkers for conservation monitoring of endangered sea turtles, with the reviewer's suggested reference added on **lines 553-554**.

*Second, the work examines broad patterns of methylation (all promoters, all coding sequences,...). While this may be interesting for descriptive purposes, it may also drown significant signals. The manuscript should mention this limitation.

Indeed, our aim was to describe the genome-wide methylation profile for sea turtles to guide future study design, as well as confirm it follows typical vertebrate methylation patterns. We have clarified these goals and limitations between **lines 529-554.** We agree that the broad-scale of comparison will likely mask significant sex-specific signals, and indeed we advocate for future studies that perform methylome-wide discovery scans on **line 552** when searching for methylation biomarkers of sex. Yet, without initial resources, this task would be difficult.

*Figure 2B shows methylation per gene. If the aim is to compare both kinds of sequencing, there should be at least one comparison of methylation per CpG, which might even be cathegorial or downsampled.

We thank the reviewer for this suggestion, which we have followed. We have repeated analyses at all 9,341,292 gene-associated CpGs that overlapped between both datasets. These additions are reported on **lines 224-227** of the Methods and **lines 388-400** of the Results/Discussion. We have replaced **Figure 2A** with a plot of methylation correlations at a random subsample of 100,000 gene-associated CpGs split by feature type for visualisation. The original donut plot was moved to **Figure S6** of the Supplement, as we feel the same information is communicated sufficiently by the chi-squared test result. We retained the gene region-level comparison in **Figure 2B**, since this is directly relevant to our analyses of the methylation profiles of TSD versus non-TSD-linked genes by feature type. Note, this figure is slightly different to the original submission, as we have now implemented a quality filter of Q8 on the WGBS to match the ONT data and improve comparability. We have changed the Methods on **line 210** to reflect this improvement.

Overall, from our linear model [lm(ONT methylation ~ Average population WGBS methylation * Feature type)] on a per-CpG basis, we found the interaction term to be significant ($F_{1,9303195}$=33969, p<0.0001), similarly to the original gene-level comparison ($F_{1,84631}$=210.3, p<0.0001). Since the interaction by feature type was significant, we next decided to perform post-hoc Pearson correlation tests separately per feature type to investigate the strength of correlation between methylation values from the ONT and 'average population WGBS methylome'. Overall, there was a strong positive correlation on a per-CpG basis across all feature types (Exons: r(486,723)=0.81, p<0.0001; Introns:

r(6,895,343)=0.75, p<0.0001; Promoters: r(499,018)=0.95, p<0.0001, Intergenic: r(1,422,111)=0.84, p<0.0001), compared to an even stronger positive correlation on a per-gene basis, as expected given noise smoothing effects (Exons: r(20,348)=0.93, p<0.0001; Introns: r(18,968)=0.96, p<0.0001; Promoters: r(23,771)=0.98, p<0.0001, Intergenic: r(21,544)=0.95, p<0.0001). This result is visualised well in **Figure 2.**

-The origin of the duplication of EP300 seems outside the scope of the manuscript. Nevertheless, given that the question is posed, the authors may want to perform a simple phylogenetic analysis of the sequences. Even the basic analysis of the annotated copies plus an outgroup is likely to give a robust answer to this question.

We agree that the evolutionary origin of possible structural variants, including EP300, is beyond the scope of this manuscript, which already contains plenty of analyses for a Data Note article. We have accordingly simplified the discussion of these results on **lines 511**. Our aim was to describe broad-scale synteny between sea turtle species for a subset of biologically relevant genes, extending similar findings from Bentley *et al.,* (2023) PNAS to the loggerhead sea turtle.

-For the benefit of non-specialists, the manuscript might include a brief mention of how microchromosomes allow a larger number of combinations of variants without chromosome recombination.

We thank the reviewer for their suggestion, and have added their suggestion to **lines 492-493.**

-Some expressions may be edited for clarity and precission. Examples are "which should be verified whether they are true" (page 17) and "microchromosomes have greater methylation potential and realised levels...".

We have edited the highlighted expressions on **lines 422** and **478.** We have also given particular attention to improving phrasing throughout all sections of the manuscript for better clarity and precision.

Overall, we would like to thank the reviewer for their insightful comments which have helped to improve the manuscript.

Reviewer #3

In this study, the authors generated a high-quality chromosome-level genome assembly and methylome for the loggerhead sea turtle (Caretta caretta) using a combination of Oxford Nanopore Technology (ONT) and Illumina sequencing. They also examined population size fluctuations, identified microchromosomes as key areas for monitoring genetic diversity and epigenetic flexibility, and focused on genes linked to temperature-dependent sex determination (TSD), with additional datasets from 10 individuals using whole-genome bisulfite sequencing (WGBS).

The study consists of three key parts: 1) genome sequencing and assembly, 2) benchmarking ONT methylation calls with WGBS, and 3) epigenetic patterning of TSD-linked genes, which was contextualized for future studies. The first part certainly includes relatively novel genomic resources that will provide valuable tools for conservation and population genomics. It's encouraging to see the

use of DNA modification detection via ONT, with a comprehensive analysis of 5mC and 5hmC methylomes alongside genomes—especially for chelonians, a group that is underrepresented among available vertebrate genomes. Benchmarking ONT methylation calls with WGBS is also relevant for the field (though some clarifications on the experimental design are necessary). However, I have several concerns regarding the biological rationale of certain study design choices and the conclusions drawn by the authors regarding the TSD-linked genes' methylation patterns.

Overall, this study provides valuable genomic resources for loggerhead sea turtles. However, some of the biological assumptions and study design choices regarding the methylation patterning require further clarification and a more robust discussion to ensure that the conclusions drawn can be supported by the data produced.

We would like to thank the reviewer for their positive assessment of our manuscript and the importance of the resources offered. We have carefully addressed all comments which help strengthen the background of our study.

Detailed comments to the authors

ABSTRACT

The abstract states: "Isolating 191 TSD-linked genes, we further built the largest network of functional associations and methylation patterns for sea turtles to date." Throughout the manuscript, this number changes. Please double-check and ensure consistency in the number of TSD-linked genes reported.

We thank the reviewer for drawing attention to how the different numbers of TSD-linked genes reported appear confusing. This arises from different numbers of TSD-linked genes being used for various analyses after question-specific filtering steps. To summarise, 199 TSD-linked genes were identified in the loggerhead genome and used for synteny comparisons across sea turtle species and methylation profiling within the reference ONT methylome. From those, 191 TSD-linked genes were then left for building the STRING protein-protein functional association map based on availability within the STRING database. We have changed the number of TSD-linked genes reported to 199 on **line 19** for consistency, as this represents the starting set of TSD-linked genes used for all analyses (described on **line 302**), with the STRING analysis-specific filtering step outlined on **lines 325-328.**

BACKGROUND

I suggest using the phrase "a skew toward female-biased sex ratios" instead of "feminisation" throughout the text for a clearer and more neutral description of the biological phenomenon. For example, the third sentence of the second paragraph could be revised as:

"As multiple theoretical studies have predicted a significant skew toward female-biased sex ratios and subsequent population collapse by 2100 in response to future climate scenarios."

We agree that referring to a skewed sex ratio is a more accurate representation of the issue faced by turtles. We have therefore implemented the reviewer's suggestion throughout the manuscript.

METHODS

Page 5, DNA extraction, sequencing, and quality control - first paragraph:

ONT kit chemistry numbers and flow cell types can be confusing for readers. Could you also clarify that the SQK-LSK109 kit used is associated with R9.4.1 flow cells, indicating the sequencing error profile of the technology?

We have added the suggested detail on **lines 96-97.**

Regarding the Phred score >Q8 cutoff: Q8 corresponds to a sequencing error rate of ~15-16%. Could you clarify the reasoning behind choosing this cutoff? Citing similar studies that have used this threshold would add support to your decision.

De novo genome assemblies for non-model species regularly use lower initial Phred score cut-offs to optimise the trade-off between number (especially of longer reads) versus quality of reads. The negative effects of including less accurate reads are minimised through rounds of error polishing steps with high coverage Illumina reads. This helps to correct errors present in the initial contigs, whilst retaining more long reads for contiguity. A common cutoff found in the literature for high-quality *de novo* genome assemblies of non-model species with R9.4 chemistry ONT reads is >Q7 (e.g., snout otter clam: Thai *et al.*, 2019 Front. Genet., Chinese mitten crab: Tang *et al.*, 2019, Front. Genet.), even going as low as >Q5 (e.g., *Fundulus* killifish species: Johnson *et al.*, 2020, GigaScience).

Guided by our raw read Phred score distribution (see screenshot below), we decided on the more stringent >Q8 cutoff, since we still retain the majority of the large peak of high-quality data. A >Q8 cutoff was also applied by Filipovíc *et al.*, (2022) BMC Genomics for assembly of the coconut rhinoceros beetle genome, where they similarly used Flye as their *de novo* assembler of R9.4 ONT reads, followed by error polishing with Illumina reads using Pilon.

Following the reviewer's suggestion, we have added a citation to Filipovíc *et al.*, (2022) on **line 102** of the Methods to support the reasoning in choosing this threshold.

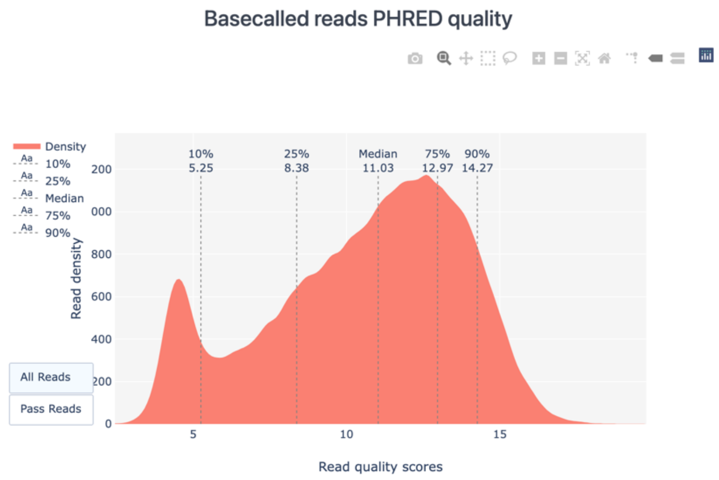

Page 8: I couldn't find the de novo assembled transcriptomes in the ENA or GigaDB repositories. Are these data publicly available? If so, it would be beneficial to provide the location.

We used two *de novo* transcriptomes during gene model prediction with the Mikado pipeline. The first transcriptome is publicly available, cited in the manuscript text on **line 171** (Ref 61: Hernández-Fernández *et al.*, 2021). We assembled the other transcriptome *de novo* using publicly available data

mined from SRA (Ref 60: Chow *et al.*, 2019 and unpublished data; details provided in **Table S1**). We have clarified these steps in **lines 170-173** of the Methods.

We did not originally include the transcriptome we assembled in GigaDB, as data were retrieved from a public database, rather than directly generated by us. Furthermore, we considered it to be a part of the genome annotation methodology, rather than a resource generated for our target East Atlantic loggerhead reference genome. We therefore refrained from including it as a genomic resource associated with this paper. However, if the reviewers and editors still prefer us to upload it, we will be happy to do so.

Page 9, ONT methylation call and validation with WGBS:

There's a discrepancy between the retained CpGs: you mention "26,449,075 CpGs" in one place and later report different numbers in the results section. Please clarify these numbers and ensure consistency. It would be helpful to include a table summarizing key metrics of the ONT methylation call, such as mean/median CpG site coverage, similar to Table S3.

26,449,075 CpGs were indeed analysed, however confusion was created as we only reported the number of CpGs with 5mC/5hmC modifications in the text. We have added **Table 2** to breakdown these numbers explicitly, including the number of CpGs without any 5mC/5hmC modifications (i.e., unmethylated), alongside the mean/median CpG coverage as suggested. Thanks to the reviewer for providing us with the opportunity to improve the clarity of these results.

Page 9, second paragraph: You mention "Ten nesting loggerheads." Please specify that these are ten adult loggerhead females for clarity. Additionally, correct the table references: Table S3 should be Table S2, Table S4 should be Table S3, etc.

We have implemented the reviewer's suggestion on **line 200**. We further thank the reviewer for catching the table referencing typos, which have now been corrected.

RESULTS AND DISCUSSION

Genome Assembly

Figure 1B: While Table 1 effectively illustrates the differences in contiguity levels, Figure 1B doesn't add much due to the difficulty in distinguishing closely aligned lines. If you retain the figure, I suggest using more contrastive colors to improve readability.

We have removed the original **Figure 1B** (contiguity plot) based on the reviewer's feedback, instead relocating the whole genome synteny plots as **Figure 1B-D.**

Genome Annotation: I agree that the lack of a pre-determined training parameter set for chelonians within the BRAKER pipeline leads to relatively incomplete gene model predictions. However, lifting over gene models from other sea turtle genomes and combining them with predictions (again using TSEBRA) would likely improve the overall completeness of the annotations.

We thank the reviewer for their interesting suggestion. Accordingly, we attempted to lift over gene models from the CBP's Adriatic loggerhead annotation onto our genome assembly using the popular Liftoff program (Shumate & Salzberg, 2021 Bioinformatics). A direct lift over resulted in a BUSCO score of 95.2% (S:94.4%, D:0.8%, F:0.7%, M:4.1%). However, when we tried to combine our original annotation with the lifted over annotation into a single consensus via EVidenceModeler, this resulted in a poorer quality annotation of BUSCO score 90.2% (S:89.3%, D:0.9%, F:1.1%, M:8.7%). Therefore, as both of these annotations were less complete than our improved *de novo* annotation (see details in response to an earlier comment), with a BUSCO score of 95.4% (S:94.4%, D:1.0%, F:1.1%, M:3.5%), we decided to retain our newer annotation.

Methylation Call and Validation

You state, "To verify our ONT methylation call, we compared calls with ten loggerhead methylomes re-sequenced via WGBS." Does this mean you generated an ONT methylome from a single individual and compared it to the average methylation levels from ten different individuals obtained with WGBS? If so, this may not be an ideal benchmarking strategy. Generating both ONT and WGBS data for all individuals would provide a more robust comparison. Clarifying this design would help the reader understand the validation process better. Additionally, consider citing relevant benchmarking studies.

Thank you to the reviewer for giving us the opportunity to clarify our approach. Indeed, we cannot directly benchmark ONT versus WGBS methylation calls here, as the same reference individual blood sample used for ONT was no longer available for paired WGBS sequencing. Therefore, as the next best option, we decided to compare against the mean methylation across ten additional individuals sequenced from the same population, with the same tissue, developmental stage, sex, and sampling protocol. We chose to compare against the average of ten individuals, so that comparisons would be more representative of the population-wide methylation profile over individual-level noise.

To avoid misleading the reader that we are performing a direct comparison of ONT versus WGBS methylation calls, we have rephrased the aim of this analysis to assessing whether our ONT-based methylome is comparable to the average population methylation profile sequenced via WGBS. Thus, we have shifted the angle from comparing sequencing methods to ensuring our reference methylome is representative of the reference population, with population-wide methylation profiles obtained via a trusted gold-standard method.

To reflect this amended angle, we renamed the Methods section title from "ONT methylation call validation with WGBS" to "ONT methylation call and comparison with population-level WGBS methylation calls" and renamed **Figure 2**'s title from "Validation of ONT-derived methylome with WGBS" to "Comparison of the ONT-derived methylome with population-level WGBS methylomes". We have also renamed the axes title headers in **Figure 2** from "ONT" to "Reference ONT methylome" and "WGBS" to "Average population WGBS methylome", with an explanation in the figure legend. In the manuscript text, we have clarified our aim on **lines 13-15** of the Abstract, **lines 230-232** of the Methods, and between **lines 377-380** and **402-304** of the Results/Discussion. We also added citations to existing benchmarking studies that compare paired ONT/WGBS sequencing (Liu et al., 2021, Liu et al., 2023, Gombert et al., 2023, Sigurpalsdottir et al., 2024) on **line 405**. We thank the reviewer again for the opportunity to clarify and improve this section of the manuscript.

In the last paragraph of this section, you highlight ONT as a robust alternative to WGBS but then use WGBS for the TSD-linked gene analysis. This appears somewhat contradictory. It might be useful to explain why WGBS was favored in this part of the analysis.

In agreement with the reviewer, we indeed used ONT, and not WGBS, for the TSD-linked gene analysis, and have emphasised this on **line 311** and **530** to minimise confusion. Noteworthy, we did try the analysis with WGBS (not included in the manuscript), and the results were extremely similar.

Genome Properties: Figures 3C-F were difficult to read to me (low resolution), and they don't seem directly related to Figures 3A and 3B. I suggest separating these figure groups for better clarity.

It appears that figure resolution was downgraded in the reviewer's copy. Following the reviewer's suggestion, we have separated these figure groups by moving the whole genome alignment plots to **Figure 1**, and retaining the macro vs microchromosome comparisons as **Figure 3**.

Additionally, it would be helpful to report or visualize the repeat content of both micro and macro chromosomes. Long-read sequencing assemblies are particularly effective at resolving repeat-rich regions, and microchromosomes are often repeat-rich. Highlighting this aspect would demonstrate the added value of long-read sequencing for assembling reference genomes of organisms like sea turtles.

We thank the reviewer for their suggestion and have added a comparison of % repeat content per chromosome to this manuscript section, with updated methods on **line 283.** As expected, microchromosomes had a lower repeat content than macrochromosomes overall (W=166, p=0.0003), with the exception of the outlier chromosome 28, which had the highest repeat content overall. The result of lower repeat content of microchromosomes is consistent with the literature (e.g., see Waters *et al.*, 2021 review). This result is reported on **lines 470** and added as a boxplot **Figure S7E,** with changes to the associated figure legend. The proportion of highly methylated CpGs was not correlated with repeat content ($F_{1,24}$=1.64, p=0.21), reported on **lines 489-490** of the Results/Discussion.

TSD-linked genes: methylation patterns

Testing methylation differences between TSD-linked and non-TSD-linked genes focusing on specific regulatory regions is potentially informative, but the biological rationale for expecting consistent differences between these two groups is unclear. TSD-linked genes are involved in dynamic, environmentally responsive processes, whereas non-TSD-linked single-copy orthologues (as used in the study) typically represent essential, evolutionarily conserved functions with more stable methylation patterns. The use of single-copy orthologues as a control set is problematic because these genes could serve fundamentally different roles. A more relevant comparison would be between TSD-linked genes and other genes involved in similarly dynamic, environmentally responsive pathways.

We thank the reviewer for thoughtfully considering this analysis. Our goal was to broadly compare whether environmentally responsive TSD-linked genes were different from the background gene methylation signal, rather than other environmentally responsive pathways. For example, the reviewer's suggestion of another environmentally responsive pathway could be genes related to immunity or the cellular heat shock response. However, it would be difficult to interpret what broad-level methylation differences mean when comparing such different pathways and phenotypes, i.e., what is the meaning of methylation differences between sex vs thermal stress vs immunity? This is further complicated by

not knowing the immune or thermal stress status of the reference female, as well as other phenotypic characteristics.

We thus retain our comparison of patterns against all 11,560 single-copy orthologues identified between sea turtle species (excluding known TSD-linked genes), as these represent the expected background gene methylation level. This comparison is justified, as TSD-linked genes themselves are also maintained as single-copy orthologues across sea turtle species (**Figure 5A-C**, Bentley et al., 2023 PNAS). Indeed, we might not expect consistent differences between TSD-linked versus non-TSD-linked genes in this broad-level comparison of genes within a single methylome of a nesting adult female, and report this result to guide future study design choices.

Additionally, all methylation data come from adult female blood (N=10, all from the same beach), which may not be the most appropriate approach for studying TSD, a process that primarily occurs during embryonic development, when temperature cues influence sex determination. Methylation patterns in adults may no longer reflect the active regulatory processes that control TSD during embryogenesis. In other words, adult methylation patterns could be influenced by factors such as reproductive status or aging, and may not reflect the regulation of TSD-linked genes during key developmental stages. These limitations/points should be addressed.

We completely agree with the reviewer that DNA methylation is tissue- and life stage-specific. However, our goal here is different from what the reviewer understood, and we are thus grateful for the opportunity to clarify it. Here, we aimed to provide the first genome-wide description of blood methylation profiles across gene feature types in a sea turtle species, with a particular focus on TSD-linked genes since they are biologically important to sea turtles. We did not attempt to make inferences on the epigenetic regulation of TSD genes in this manuscript from our methylation data. Such genome-wide methylation patterns are broadly consistent across life stages, instead reflecting species/clade-level differences (Klughammer *et al.*, 2023 Nat Communications). We offer a resource to guide future study design for sea turtles, particularly in the context of identifying correlative biomarkers of sex for conservation purposes. For example, promoters could be a particularly interesting feature type to focus the search for sex-specific methylation biomarkers, given its bimodal distribution (**Figure 5D**) and characterised link to gene regulation.

To clarify our rationale, we have rewritten the text between **lines 529-554** to better communicate the caveats and advantages of our analysis more explicitly, and simplified the description of results comparing TSD-linked versus non-TSD-linked genes (available in figure legends of **Figure S9** and **Figure S10**).

CONCLUSIONS

The manuscript would benefit from a discussion of how biological context (such as developmental stage) affects the interpretation of methylation patterns in this study.

We thank the reviewer for their suggestion, and have made the tissue and developmental stage explicit on **lines 600** and **607-608.** We decided not to discuss the biological context further here, as this section summarises the broad conclusions, with the suggested point discussed now added in response to the previous comment.

It is also worth mentioning that both ONT and WGBS require substantial amounts of input DNA, and blood samples from reptiles are ideal because of their nucleated red blood cells-this could be acknowledged as a practical advantage somewhere in the text.

We have added the reviewer's suggestion on **line 544.** As a note, the input requirements for ONT are not as high as WGBS because of the lack of a destructive DNA conversion treatment.

SUPPLEMENTARY INFO

Could you explain what "DMS" refers to in Text S3? This term isn't defined in the manuscript.

We thank the reviewer for catching this mistake – we have replaced "DMS" with "CpG sites".

There are two Figure S7, please change the last one to Figure S8.

This typo has now been corrected in the revised supplement.

SUPPORTING DATA

The FTP server data look good, but I couldn't find the de novo transcriptomes. Some files have long, confusing names—adding a README file in each directory would help clarify the contents.

We have re-organised and simplified the FTP server, with the query about *de novo* transcriptomes answered in response to an earlier comment.

Important note: It would be helpful to include line numbers in the manuscript to facilitate direct and effective feedback.

We apologise that our line numbers disappeared from the original submission and have included them in the revised manuscript.
