# Supplementary material for: Chromosome-level genome assembly and methylome profile yield insights for the conservation of endangered loggerhead sea turtles
Source: Gigascience. 2025 Jun 6;14:giaf054. doi: 10.1093/gigascience/giaf054 (PMC12143204; doi:10.1093/gigascience/giaf054)
Supplement: giaf054_Reviewer_1_Report_original_submission — Zhongduo Wang -- 10/8/2024 [file giaf054_reviewer_1_report_original_submission.pdf]

Reviewer Report

Title: Chromosome-level genome assembly and methylome profile yield insights for the conservation of endangered loggerhead sea turtles

Version: Original Submission **Date:** 10/8/2024

Reviewer name: Zhongduo Wang

Reviewer Comments to Author:

The study presents high-quality genomic and methylomic data for loggerhead sea turtles, serving as a significant resource for further genomic and epigenomic research on this species. Notably, this is the first methylome derived from a sea turtle using ONT technology, offering a new, reliable method for studying the epigenetic characteristics of non-model organisms. Moreover, by integrating genomic and methylomic data, the authors analyze the functionality and methylation patterns of TSD-related genes, contributing fresh perspectives to the molecular mechanisms underlying TSD. While the study offers valuable data, there are several areas that could be enhanced.1) Lack of Reference to Hawksbill Turtle Genome: The manuscript does not discuss any information regarding the hawksbill turtle genome. Given that hawksbills also published a comparative analysis of the loggerhead's genomic data, I recommend that the authors include relevant information or clarify why hawksbill data was not considered.2) Further Optimization of Genome Annotation: The authors acknowledge that the completeness of the genome annotation requires enhancement and mention future improvements such as species-specific parameter adjustments and manual curation. While it is understandable that time and resource constraints may have limited these optimizations prior to submission, it would be beneficial for the authors to clarify the reasons for this and outline a timeline for future enhancements.3) Information on Individual Variability in WGBS Results: The manuscript lacks specific information on inter-individual variability among the ten individuals in the WGBS data. I suggest that the authors consider adding this analysis or provide justification for its absence. If significant variability exists among individuals, averaging the methylomic data could obscure important biological information.4) Clarification on Statistical Tests and Data Processing: The manuscript employs several statistical tests such as t-tests, F-tests, and chi-squared tests. However, the methods section lacks detailed information on how the data was processed for these analyses. I recommend that the authors provide a more thorough explanation of the data preparation steps, assumptions checked, and justification for the choice of tests.In summary, this manuscript makes a significant contribution to the study of loggerhead turtle genomics and methylomics. Addressing the aforementioned points could further enhance the quality and impact of the work.

Level of Interest

Please indicate how interesting you found the manuscript: Choose an item.

Quality of Written English

Please indicate the quality of language in the manuscript: Choose an item.

Declaration of Competing Interests

Please complete a declaration of competing interests, considering the following questions:

- Have you in the past five years received reimbursements, fees, funding, or salary from an organisation that may in any way gain or lose financially from the publication of this manuscript, either now or in the future?
- Do you hold any stocks or shares in an organisation that may in any way gain or lose financially from the publication of this manuscript, either now or in the future?
- Do you hold or are you currently applying for any patents relating to the content of the manuscript?
- Have you received reimbursements, fees, funding, or salary from an organization that holds or has applied for patents relating to the content of the manuscript?
- Do you have any other financial competing interests?
- Do you have any non-financial competing interests in relation to this paper?

If you can answer no to all of the above, write 'I declare that I have no competing interests' below. If your reply is yes to any, please give details below.

I declare that I have no competing interests.

I agree to the open peer review policy of the journal. I understand that my name will be included on my report to the authors and, if the manuscript is accepted for publication, my named report including any attachments I upload will be posted on the website along with the authors' responses. I agree for my report to be made available under an Open Access Creative Commons CC-BY license (http://creativecommons.org/licenses/by/4.0/). I understand that any comments which I do not wish to be included in my named report can be included as confidential comments to the editors, which will not be published.

Choose an item.

To further support our reviewers, we have joined with Publons, where you can gain additional credit to further highlight your hard work (see: https://publons.com/journal/530/gigascience). On publication of this paper, your review will be automatically added to Publons, you can then choose whether or not to claim your Publons credit. I understand this statement.

Yes Choose an item.
