# Supplementary material for: Chromosome-level genome assembly and methylome profile yield insights for the conservation of endangered loggerhead sea turtles
Source: Gigascience. 2025 Jun 6;14:giaf054. doi: 10.1093/gigascience/giaf054 (PMC12143204; doi:10.1093/gigascience/giaf054)
Supplement: giaf054_Reviewer_2_Report_original_submission — Victor Quesada -- 10/9/2024 [file giaf054_reviewer_2_report_original_submission.pdf]

Reviewer Report

Title: Chromosome-level genome assembly and methylome profile yield insights for the conservation of endangered loggerhead sea turtles

Version: Original Submission **Date:** 10/9/2024

Reviewer name: VÃctor Quesada

Reviewer Comments to Author:

This work offers a in improved version of the reference genome for the loggerhead sea turtle. The authors have also analyzed the methylation patterns of blood obtained from different individuals and with two methods. The resulting data set includes gene annotations, methylation levels and the specific analysis of methylation levels of genes involved in temperature-dependent sex determination (TSD). While the improvements offered by this work seem modest, I think that the data sets may provide important resources for future works.-In my opinion, the use of a previous version of the same genome in the assembly process should be noted in the abstract. It would be enough to write "... followed by homolgy-guided scaffolding to GSC_CCare_1.0...".-If possible, the authors should clarify the taxonomic relationship between the reference individual in this work and the reference individual for the previous version of the genome (ref. 26). Is it the same NCBI taxid?-There is a mention to "lateral terminal repeats" at the "Genome annotation" section (page 7). I think it is a typo and it should read "long terminal repeats".-In the same section, at page 9, reference 73 refers to StringTie, not gffread. In addition, it is not clear how "in-frame stop codons were removed". A simple way to unambiguously explain this would be to provide the options that were used, as with other programs.-I would revise the use of "coverage" versus "depth". For instance, the expression "...a coverage of 9.2(...)X" would be more precise as "...a sequencing depth of 9.2(...)X". Coverage should be a fraction or a percentage. However, this is only a piece of advice, as there is no strong consensus at the moment.-The interpretation of methylation patterns is always difficult. In my opinion, the manuscript should discuss several limitations about the results:*First, using blood as the starting tissue is convenient but not ideal, as many methylation patterns are tissue-specific. The authors may want to add a reference to preliminary evidence that some methylation changes in blood cells are related to TSD (Bock et al., Mol Ecol. 2022; 31:5487-5505).*Second, the work examines broad patterns of methylation (all promoters, all coding sequences,...). While this may be interesting for descriptive purposes, it may also drown significant signals. The manuscript should mention this limitation.*Figure 2B shows methylation per gene. If the aim is to compare both kinds of sequencing, there should be at least one comparison of methylation per CpG, which might even be cathegorial or downsampled.-The origin of the duplication of EP300 seems outside the scope of the manuscript. Nevertheless, given that the question is posed, the authors may want to perform a simple phylogenetic analysis of the sequences. Even the basic analysis of the annotated copies plus an outgroup is likely to give a robust answer to this question.-For the benefit of non-specialists, the manuscript might include a brief mention of how microchromosomes allow a larger number of combinations of variants without chromosome recombination.-Some expressions may be

edited for clarity and precission. Examples are "which should be verified whether they are true" (page 17) and "microchromosomes have greater methylation potential and realised levels...".

Level of Interest

Please indicate how interesting you found the manuscript: Choose an item.

Quality of Written English

Please indicate the quality of language in the manuscript: Choose an item.

Declaration of Competing Interests

Please complete a declaration of competing interests, considering the following questions:

- Have you in the past five years received reimbursements, fees, funding, or salary from an organisation that may in any way gain or lose financially from the publication of this manuscript, either now or in the future?
- Do you hold any stocks or shares in an organisation that may in any way gain or lose financially from the publication of this manuscript, either now or in the future?
- Do you hold or are you currently applying for any patents relating to the content of the manuscript?
- Have you received reimbursements, fees, funding, or salary from an organization that holds or has applied for patents relating to the content of the manuscript?
- Do you have any other financial competing interests?
- Do you have any non-financial competing interests in relation to this paper?

If you can answer no to all of the above, write 'I declare that I have no competing interests' below. If your reply is yes to any, please give details below.

I declare that I have no competing interests

I agree to the open peer review policy of the journal. I understand that my name will be included on my report to the authors and, if the manuscript is accepted for publication, my named report including any attachments I upload will be posted on the website along with the authors' responses. I agree for my report to be made available under an Open Access Creative Commons CC-BY license (http://creativecommons.org/licenses/by/4.0/). I understand that any comments which I do not wish to be included in my named report can be included as confidential comments to the editors, which will not be published.

Choose an item.

To further support our reviewers, we have joined with Publons, where you can gain additional credit to further highlight your hard work (see: https://publons.com/journal/530/gigascience). On publication of this paper, your review will be automatically added to Publons, you can then choose whether or not to claim your Publons credit. I understand this statement.

Yes Choose an item.
