# Supplementary material for: Chromosome-level genome assembly and methylome profile yield insights for the conservation of endangered loggerhead sea turtles
Source: Gigascience. 2025 Jun 6;14:giaf054. doi: 10.1093/gigascience/giaf054 (PMC12143204; doi:10.1093/gigascience/giaf054)
Supplement: giaf054_Reviewer_3_Report_original_submission — F. Gözde Çilingir -- 10/12/2024 [file giaf054_reviewer_3_report_original_submission.pdf]

Reviewer Report

Title: Chromosome-level genome assembly and methylome profile yield insights for the conservation of endangered loggerhead sea turtles

Version: Original Submission **Date:** 10/12/2024

Reviewer name: F. GÃ¶zde Ã‡ilingir

Reviewer Comments to Author:

In this study, the authors generated a high-quality chromosome-level genome assembly and methylome for the loggerhead sea turtle (Caretta caretta) using a combination of Oxford Nanopore Technology (ONT) and Illumina sequencing. They also examined population size fluctuations, identified microchromosomes as key areas for monitoring genetic diversity and epigenetic flexibility, and focused on genes linked to temperature-dependent sex determination (TSD), with additional datasets from 10 individuals using whole-genome bisulfite sequencing (WGBS).The study consists of three key parts: 1) genome sequencing and assembly, 2) benchmarking ONT methylation calls with WGBS, and 3) epigenetic patterning of TSD-linked genes, which was contextualized for future studies. The first part certainly includes relatively novel genomic resources that will provide valuable tools for conservation and population genomics. It's encouraging to see the use of DNA modification detection via ONT, with a comprehensive analysis of 5mC and 5hmC methylomes alongside genomesâ€”especially for chelonians, a group that is underrepresented among available vertebrate genomes. Benchmarking ONT methylation calls with WGBS is also relevant for the field (though some clarifications on the experimental design are necessary). However, I have several concerns regarding the biological rationale of certain study design choices and the conclusions drawn by the authors regarding the TSD-linked genes' methylation patterns.Overall, this study provides valuable genomic resources for loggerhead sea turtles. However, some of the biological assumptions and study design choices regarding the methylation patterning require further clarification and a more robust discussion to ensure that the conclusions drawn can be supported by the data produced.Detailed comments to the authorsABSTRACTThe abstract states: "Isolating 191 TSD-linked genes, we further built the largest network of functional associations and methylation patterns for sea turtles to date."Throughout the manuscript, this number changes. Please double-check and ensure consistency in the number of TSD-linked genes reported.BACKGROUNDI suggest using the phrase "a skew toward female-biased sex ratios" instead of "feminisation" throughout the text for a clearer and more neutral description of the biological phenomenon. For example, the third sentence of the second paragraph could be revised as:"As multiple theoretical studies have predicted a significant skew toward female-biased sex ratios and subsequent population collapse by 2100 in response to future climate scenarios."METHODSPage 5, DNA extraction, sequencing, and quality control - first paragraph:ONT kit chemistry numbers and flow cell types can be confusing for readers. Could you also clarify that the SQK-LSK109 kit used is associated with R9.4.1 flow cells, indicating the sequencing error profile of the technology?Regarding the Phred score >Q8 cutoff: Q8 corresponds to a sequencing error rate of ~15-16%. Could you clarify the reasoning behind choosing this cutoff? Citing similar studies that have used this threshold would add support to your decision.Page 8: I couldn't find the de novo

assembled transcriptomes in the ENA or GigaDB repositories. Are these data publicly available? If so, it would be beneficial to provide the location.Page 9, ONT methylation call and validation with WGBS:There's a discrepancy between the retained CpGs: you mention "26,449,075 CpGs" in one place and later report different numbers in the results section. Please clarify these numbers and ensure consistency.It would be helpful to include a table summarizing key metrics of the ONT methylation call, such as mean/median CpG site coverage, similar to Table S3.Page 9, second paragraph: You mention "Ten nesting loggerheads." Please specify that these are ten adult loggerhead females for clarity. Additionally, correct the table references: Table S3 should be Table S2, Table S4 should be Table S3, etc.RESULTS AND DISCUSSIONGenome AssemblyFigure 1B: While Table 1 effectively illustrates the differences in contiguity levels, Figure 1B doesn't add much due to the difficulty in distinguishing closely aligned lines. If you retain the figure, I suggest using more contrastive colors to improve readability.Genome Annotation: I agree that the lack of a pre-determined training parameter set for chelonians within the BRAKER pipeline leads to relatively incomplete gene model predictions. However, lifting over gene models from other sea turtle genomes and combining them with predictions (again using TSEBRA) would likely improve the overall completeness of the annotations.Methylation Call and ValidationYou state, "To verify our ONT methylation call, we compared calls with ten loggerhead methylomes re-sequenced via WGBS." Does this mean you generated an ONT methylome from a single individual and compared it to the average methylation levels from ten different individuals obtained with WGBS? If so, this may not be an ideal benchmarking strategy. Generating both ONT and WGBS data for all individuals would provide a more robust comparison. Clarifying this design would help the reader understand the validation process better. Additionally, consider citing relevant benchmarking studies.In the last paragraph of this section, you highlight ONT as a robust alternative to WGBS but then use WGBS for the TSD-linked gene analysis. This appears somewhat contradictory. It might be useful to explain why WGBS was favored in this part of the analysis.Genome Properties: Figures 3C-F were difficult to read to me (low resolution), and they don't seem directly related to Figures 3A and 3B. I suggest separating these figure groups for better clarity. Additionally, it would be helpful to report or visualize the repeat content of both micro and macro chromosomes. Long-read sequencing assemblies are particularly effective at resolving repeat-rich regions, and microchromosomes are often repeat-rich. Highlighting this aspect would demonstrate the added value of long-read sequencing for assembling reference genomes of organisms like sea turtles.TSD-linked genes: methylation patternsTesting methylation differences between TSD-linked and non-TSD-linked genes focusing on specific regulatory regions is potentially informative, but the biological rationale for expecting consistent differences between these two groups is unclear. TSD-linked genes are involved in dynamic, environmentally responsive processes, whereas non-TSD-linked single-copy orthologues (as used in the study) typically represent essential, evolutionarily conserved functions with more stable methylation patterns. The use of single-copy orthologues as a control set is problematic because these genes could serve fundamentally different roles. A more relevant comparison would be between TSD-linked genes and other genes involved in similarly dynamic, environmentally responsive pathways.Additionally, all methylation data come from adult female blood (N=10, all from the same beach), which may not be the most appropriate approach for studying TSD, a process that primarily occurs during embryonic development, when temperature cues influence sex determination. Methylation patterns in adults may no longer reflect the active regulatory processes that control TSD during embryogenesis. In other words, adult methylation patterns

could be influenced by factors such as reproductive status or aging, and may not reflect the regulation of TSD-linked genes during key developmental stages. These limitations/points should be addressed.CONCLUSIONSThe manuscript would benefit from a discussion of how biological context (such as developmental stage) affects the interpretation of methylation patterns in this study.It is also worth mentioning that both ONT and WGBS require substantial amounts of input DNA, and blood samples from reptiles are ideal because of their nucleated red blood cells-this could be acknowledged as a practical advantage somewhere in the text.SUPPLEMENTARY INFOCould you explain what "DMS" refers to in Text S3? This term isn't defined in the manuscript.There are two Figure S7, please change the last one to Figure S8.SUPPORTING DATAThe FTP server data look good, but I couldn't find the de novo transcriptomes. Some files have long, confusing namesâ€”adding a README file in each directory would help clarify the contents.Important note: It would be helpful to include line numbers in the manuscript to facilitate direct and effective feedback.

Level of Interest

Please indicate how interesting you found the manuscript: Choose an item.

Quality of Written English

Please indicate the quality of language in the manuscript: Choose an item.

Declaration of Competing Interests

Please complete a declaration of competing interests, considering the following questions:

- Have you in the past five years received reimbursements, fees, funding, or salary from an organisation that may in any way gain or lose financially from the publication of this manuscript, either now or in the future?
- Do you hold any stocks or shares in an organisation that may in any way gain or lose financially from the publication of this manuscript, either now or in the future?
- Do you hold or are you currently applying for any patents relating to the content of the manuscript?
- Have you received reimbursements, fees, funding, or salary from an organization that holds or has applied for patents relating to the content of the manuscript?
- Do you have any other financial competing interests?
- Do you have any non-financial competing interests in relation to this paper?

If you can answer no to all of the above, write 'I declare that I have no competing interests' below. If your reply is yes to any, please give details below.

I declare that I have no competing interests.

I agree to the open peer review policy of the journal. I understand that my name will be included on my report to the authors and, if the manuscript is accepted for publication, my named report including any attachments I upload will be posted on the website along with the authors' responses. I agree for my report to be made available under an Open Access Creative Commons CC-BY license (http://creativecommons.org/licenses/by/4.0/). I understand that any comments which I do not wish to be included in my named report can be included as confidential comments to the editors, which will not be published.

Choose an item.

To further support our reviewers, we have joined with Publons, where you can gain additional credit to further highlight your hard work (see: https://publons.com/journal/530/gigascience). On publication of this paper, your review will be automatically added to Publons, you can then choose whether or not to claim your Publons credit. I understand this statement.

Yes Choose an item.
